# Supplementary material for: Electrocatalytic continuous flow chlorinations with iodine(I/III) mediators
Source: Nat Commun. 2024 Jul 27;15:6329. doi: 10.1038/s41467-024-50643-z (PMC11283512; doi:10.1038/s41467-024-50643-z)
Supplement: Supplementary file 1 — Supplementary Information [file 41467_2024_50643_MOESM1_ESM.pdf]

<u>Supplementary Information</u>

Electrocatalytic Continuous Flow Chlorinations with Iodine(I/III) Mediators

Contents

1. General Information 3
1.1. Reagents, solvents and experimental conditions 3
1.2. Analytical techniques 3
1.3. Compound purification 4
1.4. Electrochemical setup 4
2. Experimental Details 5
2.1. Chloride source evaluation for the generation of $PhICl_2$ 5
2.2. Synthesis of $ArICl_2$ 6
2.3. Mechanistic study 10
2.4. Ex-cell dichlorination of alkene with *in situ* generated $ArICl_2$ 17
2.5. Optimisation for alkene dichlorination with catalytic 4-iodotoluene 17
2.6. Synthesis and characterisation data for starting materials 19
2.7. General procedure (GP3) for the dichlorination of alkenes 27
2.8. General procedure (GP4) for dichlorination of alkenes and monochlorination of 1,3-dicarbonyl compounds in non-electrolysis conditions 27
2.9. Characterization data for the products 28
3. HPLC traces 44
4. NMR Spectra 47
5. References 99

1. General Information

1.1. Reagents, solvents and experimental conditions

All reagents were purchased from Alfa Aesar, Sigma-Aldrich, Fluorochem, Acros Organics, Fisher Scientific and used without further purification, except otherwise stated. Dry solvents such THF and acetonitrile were obtained after passing these previously degassed solvents through activated alumina columns (Mbraun, SPS-800). Dry CH_2Cl_2, were obtained by distillation over CaH_2 under nitrogen atmosphere. Reactions involving air and moisture sensitive reagents were carried out in oven-dried glassware under an atmosphere of argon using standard Schlenk technique. Reaction temperatures are referred to the temperature of the heating medium, unless otherwise stated.

1.2. Analytical techniques

NMR-spectra were recorded on Bruker DPX 300, 400 or 500 spectrometers. All spectral data was acquired at 295 K. Deuterated solvents for NMR analysis were purchased from Sigma Aldrich. ^{1}H and ^{13}C chemicals shifts (δ) are quoted in parts per million (ppm) against tetramethylsilane (TMS, δ = 0.00 ppm) and were internally referenced to residual $CHCl_3$ (7.26 ppm for ^{1}H, 77.16 ppm for ^{13}C) or DMSO (2.50 ppm for ^{1}H, 39.52 ppm for ^{13}C). ^{19}F chemicals shifts (δ) are quoted in parts per million (ppm) and were calibrated using absolute referencing to the ^{1}H NMR spectrum. Coupling constants (*J*) are reported in Hertz (Hz) to the nearest 0.1 Hz. The following abbreviations (or combinations thereof) were used to explain multiplicities: s = singlet, d = doublet, t = triplet, q = quartet, quintet, p = pentet, m = multiplet.

High-resolution mass spectra (HRMS) were obtained by the MS service of the Cardiff University on a Water LCR Premier XE-TOF. Ions were generated by the Atmospheric Pressure Ionisation Techniques (APCI), Electrospray (ESI) and Electron Ionisation (EI).

GC-MS spectra were recorded on a Perkin Elmer Clarus 680 GC fitted with a Perkin Elmer Elite-1 column 100% dimethyl polysiloxane (30 m × 0.25 mm internal diameter) and a Perkin Elmer Clarus SQ 8 C mass spectrometer.

Gas chromatography with flame ionisation detector (GC-FID) was performed on an Agilent 7890A GC system fitted with a Restek Rt-bDEXsm column (30 m × 0.32 mm internal diameter).

The cyclic voltammogram studies were performed in an Orygalys OGF500 Potentiostat / Galvanostat with OGFPWR power supply. Working electrode: glassy carbon electrode tip, counter electrode: Pt wire; reference electrode: Ag/AgCl in saturated KCl solution; solvent: HFIP/MeCN; scan rate, v = 100 mV/s; c = 5 mM; supporting electrolyte: Bu_4NClO_4.

Thin layer chromatography was carried out on Merck silica gel 60 F_{254} (0.20 mm) pre-coated aluminum sheets and were visualised using UV light (254 nm) and stained with basic aqueous potassium permanganate or ethanolic phosphomolybdic acid solution.

1.3. Compound purification

Flash chromatography was carried out using silica gel (Acros Organics, 0.035-0.070 mm, 60 Å) under a light positive pressure of argon, eluting with the specified solvent system as mentioned. Solvents for chromatographic purification (petroleum ether, CH_2Cl_2 and EtOAc) were purchased from commercial sources and used directly.

1.4. Electrochemical setup

Flow electrochemical experiments were carried out with an Ion electrochemical reactor from Vapourtec Ltd. In this setup, working and counter electrodes (5 cm × 5 cm) were separated by a 0.5 mm thick FEP spacer resulting in a reactor volume of 600 μL and exposed electrode surface area of 12 cm^2. Rigid graphite (99.95% purity) and Platinum foil (99.95% purity) were procured from Goodfellow and glassy carbon electrode was obtained from Vapourtec. KR Analytical Ltd Fusion 100 Touch syringe pumps were used to pump the reagent solution through the assembled undivided flow electrochemical reactor and was collected in suitable volumetric flask. Aim-TTi Digital Bench Power Supply (280 W, 2 Output, 0 → 35V 0 → 4A) was used for electrolysis under constant current conditions (Supplementary Figure 1).

a **b**

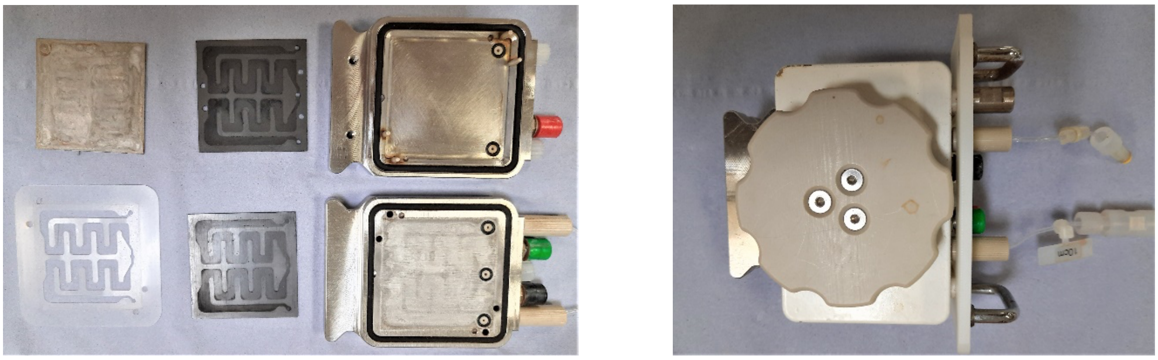

Supplementary Figure 1. The Vapourtec Ion Electrochemical Flow Reactor. a Disassembled components. **b** Assembled reactor.

Batch electrochemical experiments were carried out with an Electrasyn 2.0 device with 5 mL or 10 mL Electrasyn vials. Glassy carbon and graphite (5 cm × 0.8 cm × 0.2 cm) electrodes were obtained from IKA. Platinum foil (5 cm × 0.5 cm) was wrapped around a piece of PTFE (Polytetrafluoroethylene) block (5 cm × 1.0 cm × 0.2 cm) to prepare the platinum electrode.

2. Experimental Details

2.1. Chloride source evaluation for the generation of $PhICl_2$

For the chloride source evaluation, first the hexafluoroisopropoxide ligated I(III) reagent in hexafluoroisopropanol (HFIP) solution was synthesised by following our previous report[1]. Then different chloride sources were evaluated for the generation of $PhICl_2$ by chloride ligand exchange.

A solution of iodobenzene (0.1 M) in HFIP, containing Bu_4NBF_4 (0.005 M), was pumped into the Vapourtec Ion Electrochemical flow reactor (reactor volume = 0.6 mL, spacer 0.5 mm) containing a glassy carbon electrode (effective surface area for electrolysis: A = 12 cm^2) as the anode and a platinum electrode as the cathode by using a syringe pump (0.05 mL/min). A constant current of 24 mA (3 F) was applied for the electrolysis purpose. After reaching a steady state (24 min, 2 reactor vol.), the solution was collected for 24 min in a glass vial. Due to the instability of the electrogenerated I(III) compound, a 0.2 mL aliquot was diluted with 0.3 mL of $CDCl_3$ and the conversion of phenyl iodide to I(III) compound was determined by integration of the aromatic peaks in the ^1H NMR spectrum obtained. Next, a chloride source and a Teflon coated magnetic stirrer was added in this solution and the solution was stirred at room temperature for 6 h. Next, 0.5 mL of dichloromethane was added to this solution and stirred for 15 min to make a homogeneous solution. A 0.2 mL of aliquot was taken from this solution and was diluted with 0.3 mL of $CDCl_3$. The conversion of hexafluoroisopropoxide ligated I(III) reagent into $ArICl_2$ was determined by integration of the aromatic peaks in the ^1H NMR spectrum.

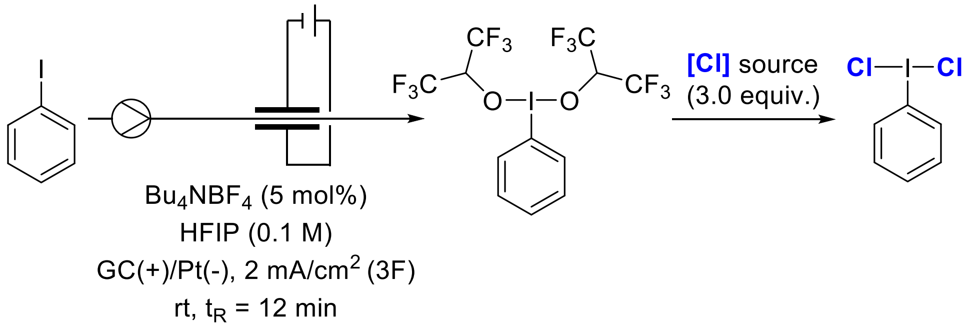

Supplementary Table 1. Variation of chloride source.

Entry	Chloride source	Yield (%)
1	CsCl	18
2	LiCl	11
3	TMSCl	58
4	HCl in MeOH (3 M)	52
5	Et_4NCl	38

2.2. Synthesis of $ArICl_2$

Dichloro(phenyl)-λ[3]-iodane (3a)

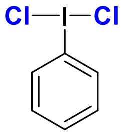

Following the above-mentioned procedure addition of TMSCl led to a yellow precipitate. The residue was filtered and washed with *n*-pentane to provide **3a** as a pale-yellow solid (15 mg, 0.055 mmol, 58%). NMR data is identical with the literature[1].

Dichloro(*p*-tolyl)-λ[3]-iodane (3b)

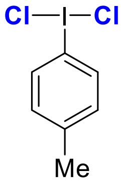

Following the above procedure with 4-iodotoluene, addition of TMSCl led to a yellow precipitate. The residue was filtered and washed with *n*-pentane to provide **3b** as a yellow solid (18 mg, 0.063 mmol, 63%). NMR data is identical with the literature[2].

Compound **3b** has also been prepared starting from 4-iodotoluene (1.444 g, 5 mmol) using a reported procedure[3] with $NaClO_2$ as oxidant in conc. HCl. Compound **3b** was obtained in 78% yield (1.12 g, 3.9 mmol).

Dichloro(4-fluorophenyl)-λ[3]-iodane (3c)

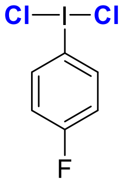

Following the above procedure with 4-fluoroiodobenzene, addition of TMSCl led to an orange solution. Due to the instability of **3c** under reduced pressure, a 0.2 mL of aliquot was used for ^{1}H NMR yield and the formation of the compound was confirmed by comparing the ^{1}H NMR shift with respect to **1c**.

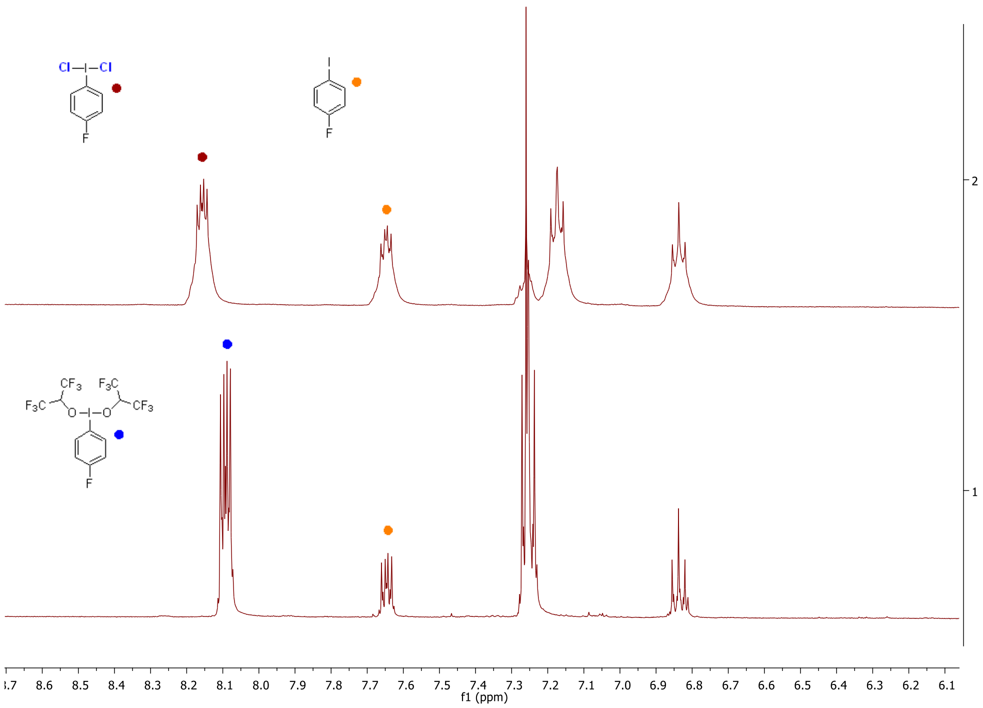

^{1}H NMR (500 MHz, $CDCl_3$)

Dichloro(4-chlorophenyl)-λ^3-iodane (3d)

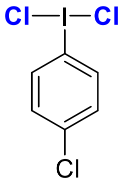

Following the above procedure with 4-chloroiodobenzene, addition of TMSCl led to a yellow solution. Due to the instability of **3d** under reduced pressure, a 0.2 mL of aliquot was used for ^{1}H NMR yield and the formation of the compound was confirmed by comparing the ^{1}H NMR shift with respect to **3d**.

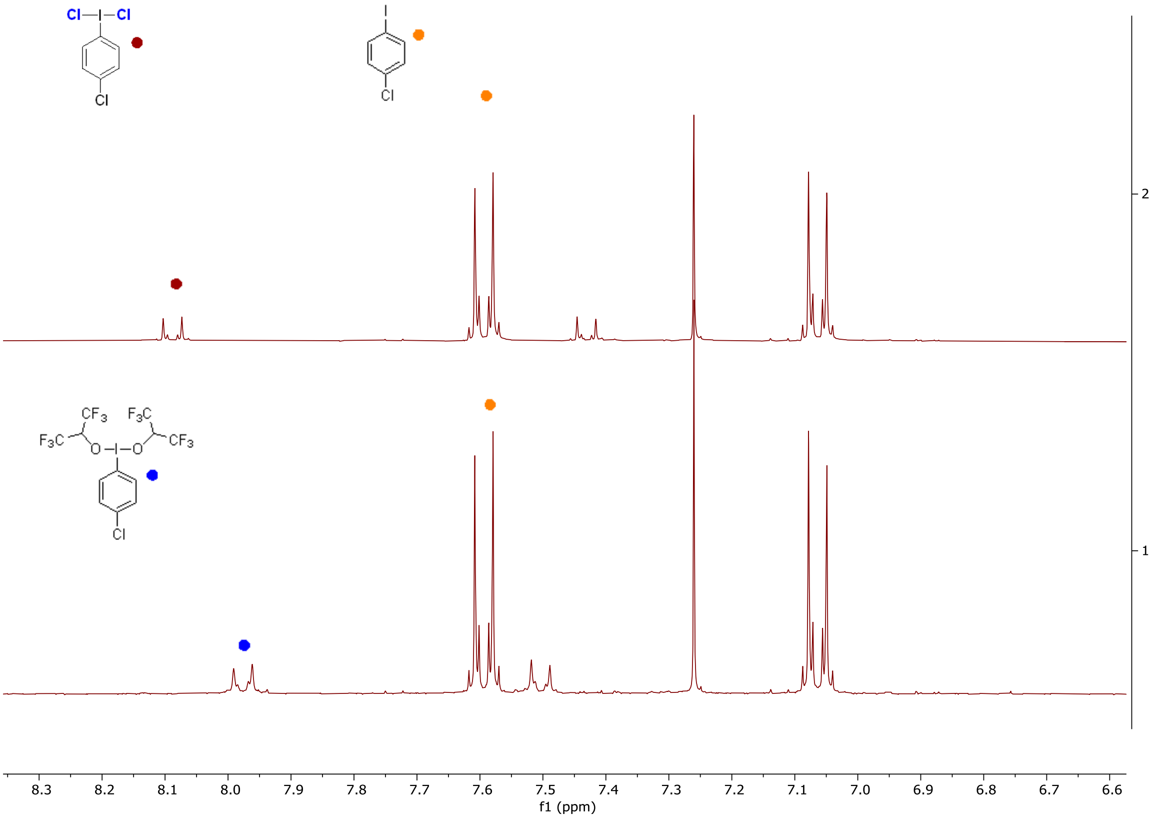

^{1}H NMR (300 MHz, $CDCl_3$)

Dichloro(3-(trifluoromethyl)phenyl)-λ³-iodane (3e)

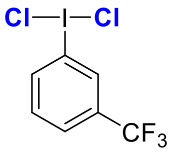

Following the above procedure with 3-trifluoromethyliodobenzene, addition of TMSCl led to a yellowish solution. Due to the instability of **3e** under reduced pressure, a 0.2 mL of aliquot was used for ^{1}H NMR yield and the formation of the compound was confirmed by comparing the ^{1}H NMR shift with respect to **3e**.

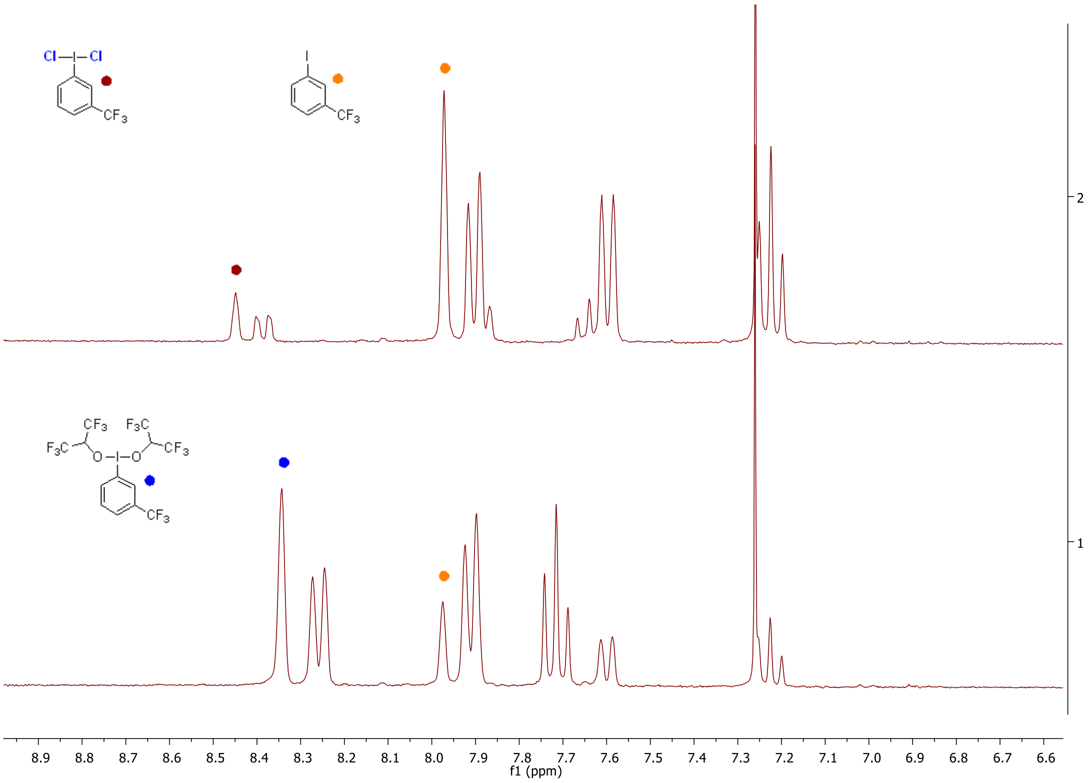

^{1}H NMR (300 MHz, $CDCl_3$)

(3,5-Bis(trifluoromethyl)phenyl)dichloro-λ³-iodane (3f)

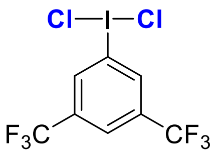

Following the above procedure with 3,5-bis(trifluoromethyl)-iodobenzene and after the addition of TMSCl no trace of **3f** was found in the ^{1}H NMR comparison with respect to **1f**.

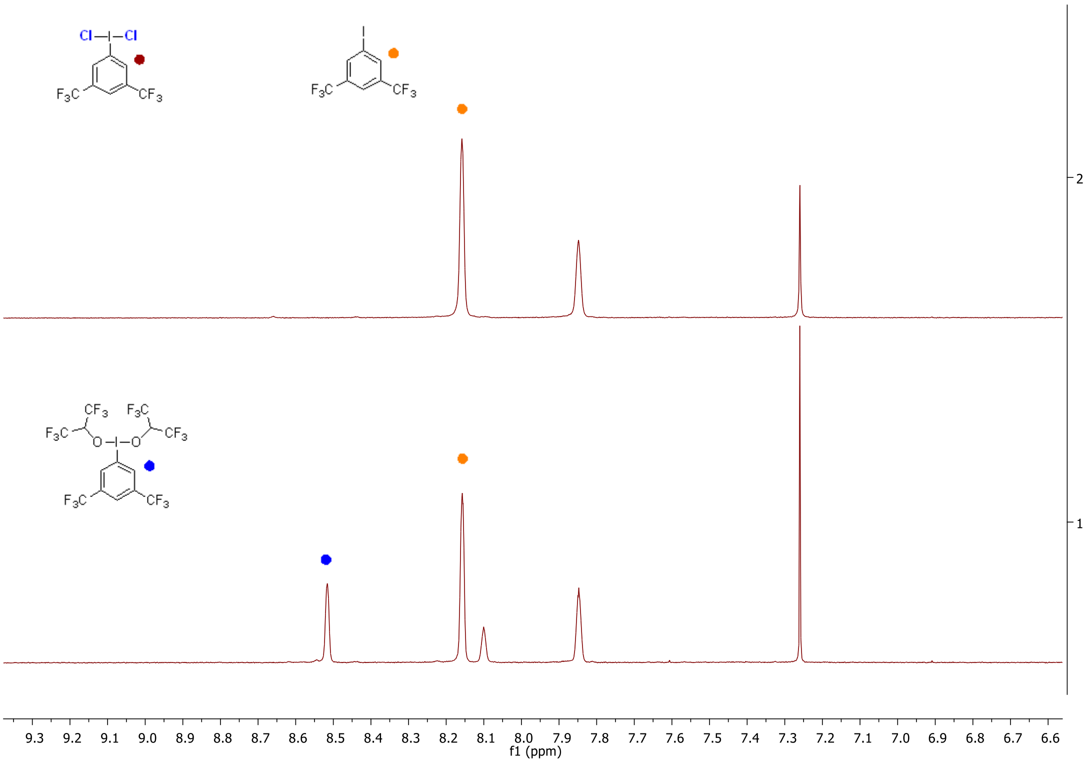

^{1}H NMR (300 MHz, $CDCl_3$)

2.3. Mechanistic study

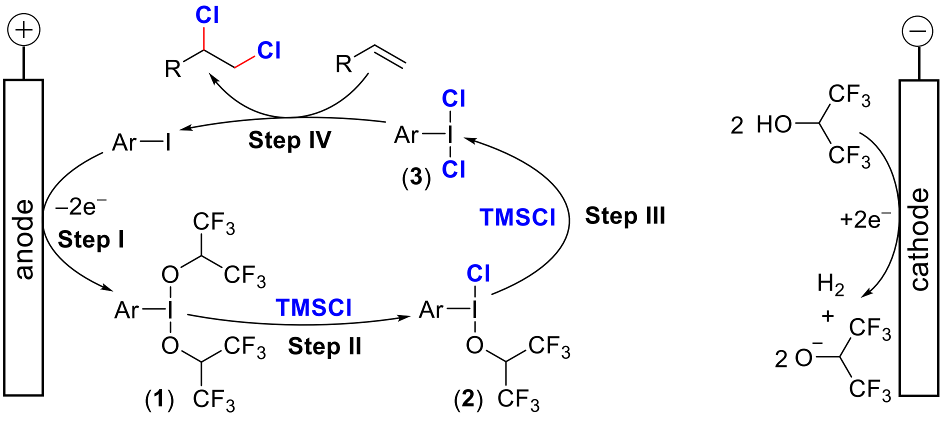

Supplementary Figure 2. Proposed mechanism.

Mechanism: Step I

Formation of **1** was reported in identical conditions in HFIP solution[4].

Mechanism: Step II and Step III (NMR titration for intermediate trapping)

Each of four oven dried 5 mL dram vials was charged with a magnetic stir-bar and an aliquot of freshly prepared **1** (0.02 mmol, 100 µL of 0.2 M solution) in HFIP. In this vials, 0 equiv., 0.5 equiv. (1.5 µL, 0.01 mmol), 1.0 equiv. (3.0 µL, 0.02 mmol) and 2.0 equiv. (6.0 µL, 0.04 mmol) of TMSCl were added respectively and stirred at room temperature for 10 minutes. Then 400 µL of $CDCl_3$ was added in each of these vials and ^1H and ^{19}F NMR were recorded. Similarly with freshly prepared $PhICl_2$ (0.02 mmol) 100 µL HFIP and 400 µL of $CDCl_3$ were added and ^1H and ^{19}F NMR were also recorded for comparison.

In-situ formation of **2a** was observed from both ^1H and ^{19}F NMR spectra. Additionally, fate of TMS group was confirmed from the formation of TMS–$OCH(CF_3)_2$ as observed in the ^{19}F NMR spectra.

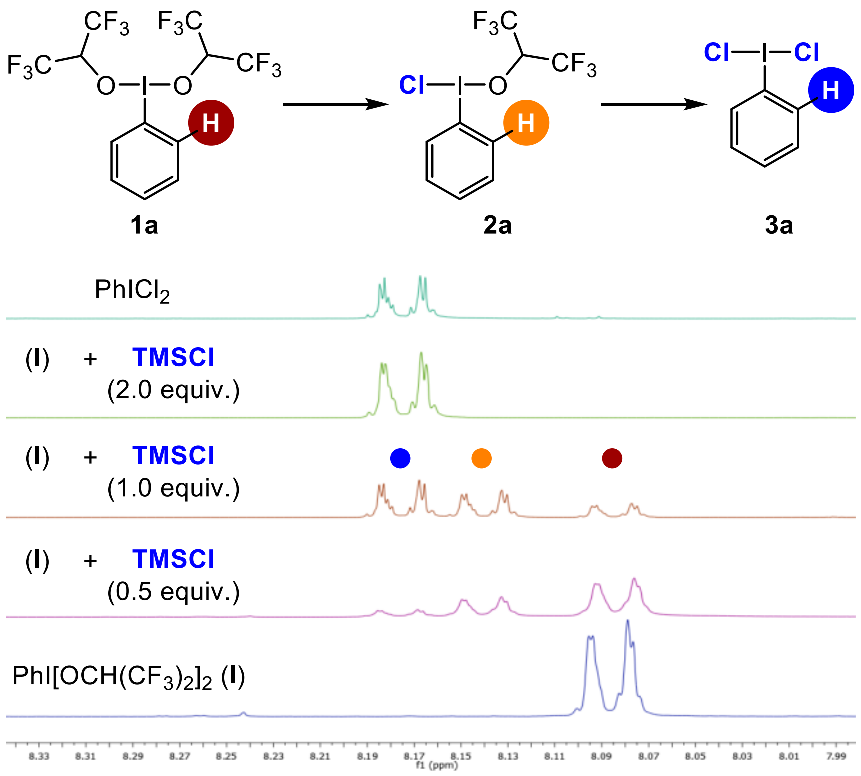

Supplementary Figure 3. **^1H NMR study for stepwise chloride exchange (^1H NMR, 500 MHz, $CDCl_3$).**

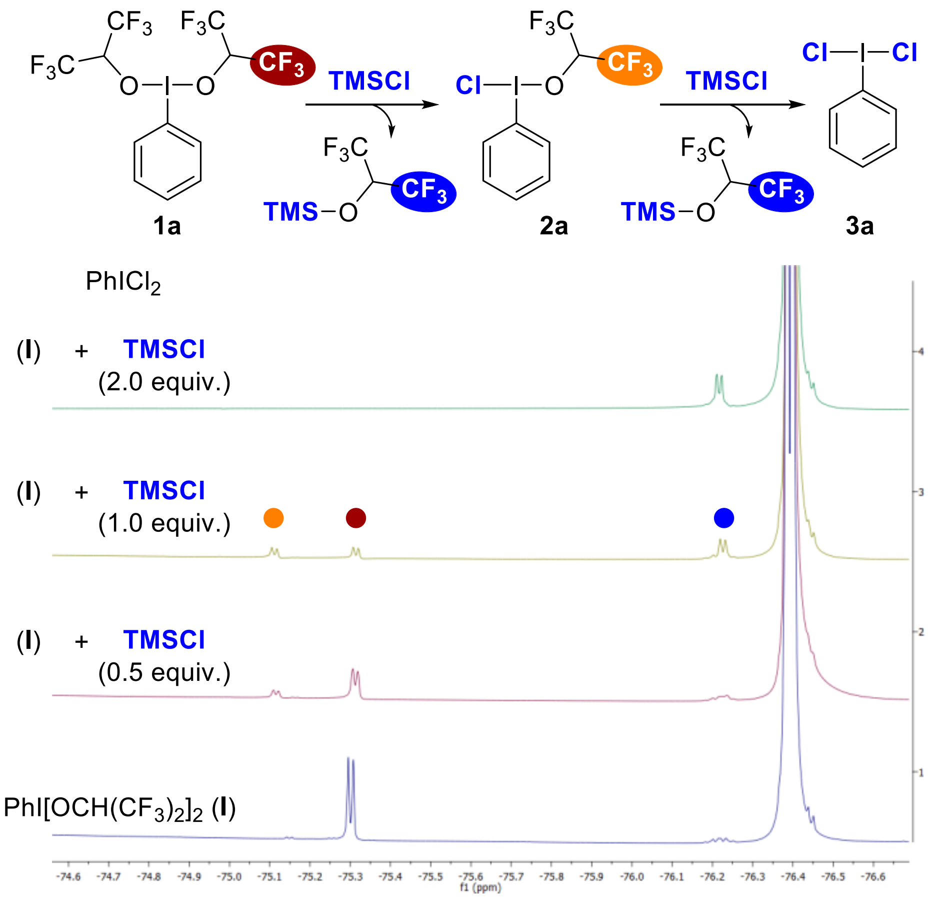

Supplementary Figure 4. ^{19}F NMR study for stepwise chloride exchange (^{19}F NMR, 470 MHz, $CDCl_3$).

Irreversibility between Step II and Step III

Despite our efforts, a reverse reaction from step III to step II was not observed. The ligand exchange was not observed even with hexafluoroisopropoxide ion. Which further confirms that though **2** is formed in our reaction conditions, but it is most likely not an active intermediate responsible for chlorination reactions.

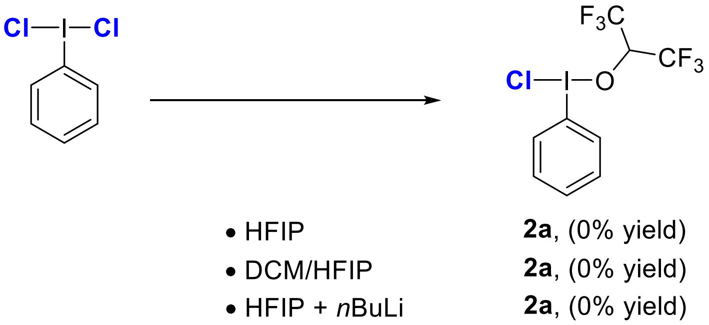

Supplementary Figure 5. Irreversible nature of chloride ligand exchange.

Control reaction to rule out Cl_2 mediated dichlorination reaction.

The reaction mixture was electrolysed without adding any alkene acceptor or aryl iodide catalyst under otherwise standard conditions. Then but-3-en-1-yl benzoate was

added into the reaction in an ex-cell fashion and the reaction mixture was stirred for 6 hours. Apart from the unreacted alkene, no trace of the 1,2-dichloro addition product was detected from GC/MS analysis. This ex-cell reaction proved that Cl_2 did not accumulate during the reaction.

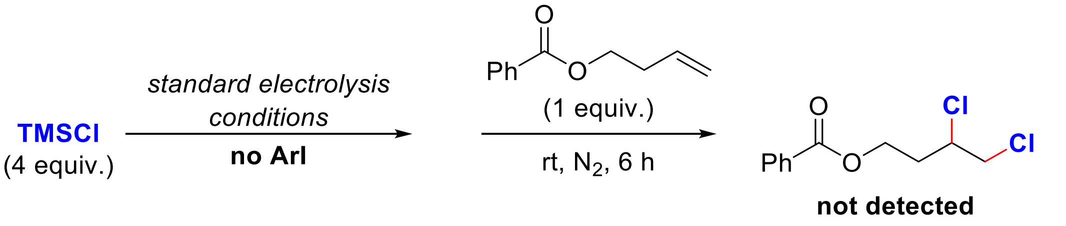

Attempted flow electrolysis for cyclisation of 4,5-dichloropentanoic acid

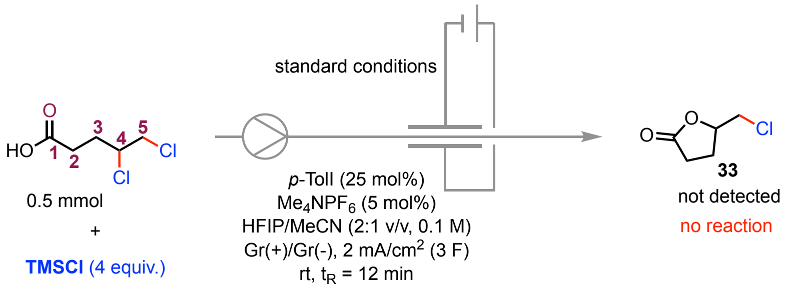

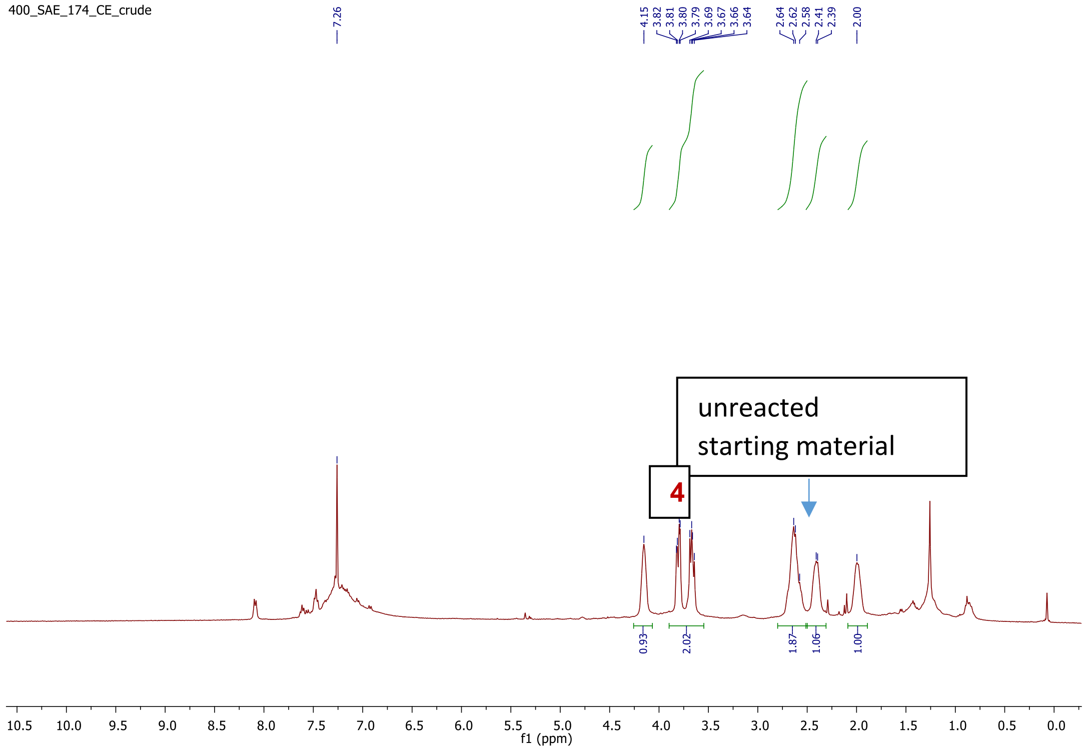

^1H NMR (400 MHz, $CDCl_3$)

This experimental result suggests that chlorocyclisation is not undergoing via dichlorination pathway. This is consistent with the mechanisms reported for hypervalent iodine mediated halocyclisation reactions[5].

Radical trapping in the monochlorination of 1,3-dicarbonyl compounds

All experiments were conducted according to **GP4**. Yields were determined by ^{1}H NMR analysis of the crude reaction mixtures.

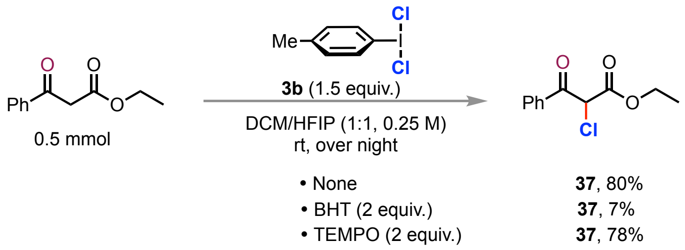

Supplementary Figure 6. Radical trapping for the monochlorination of 1,3-dicarbonyl compounds.

Uncatalyzed electrochemical continuous flow chlorination reactions with electron rich substrates

All these experiments (**i**, **ii** and **iii**) were carried out following **GP3** but without catalyst.

i.

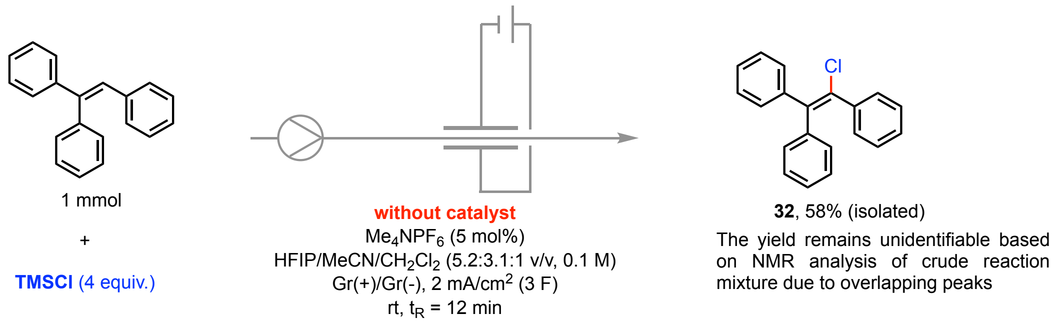

ii.

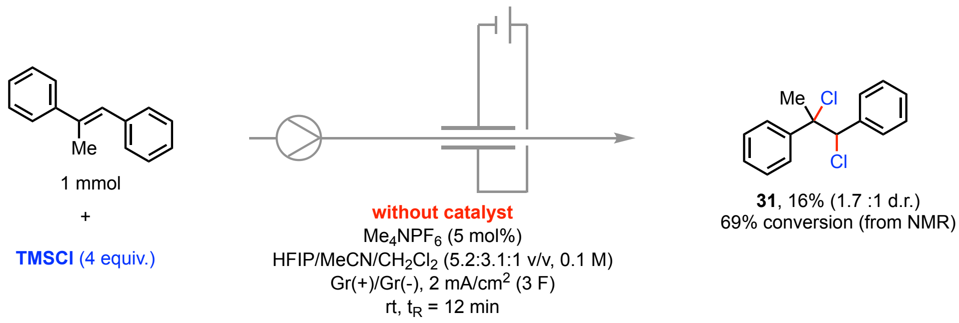

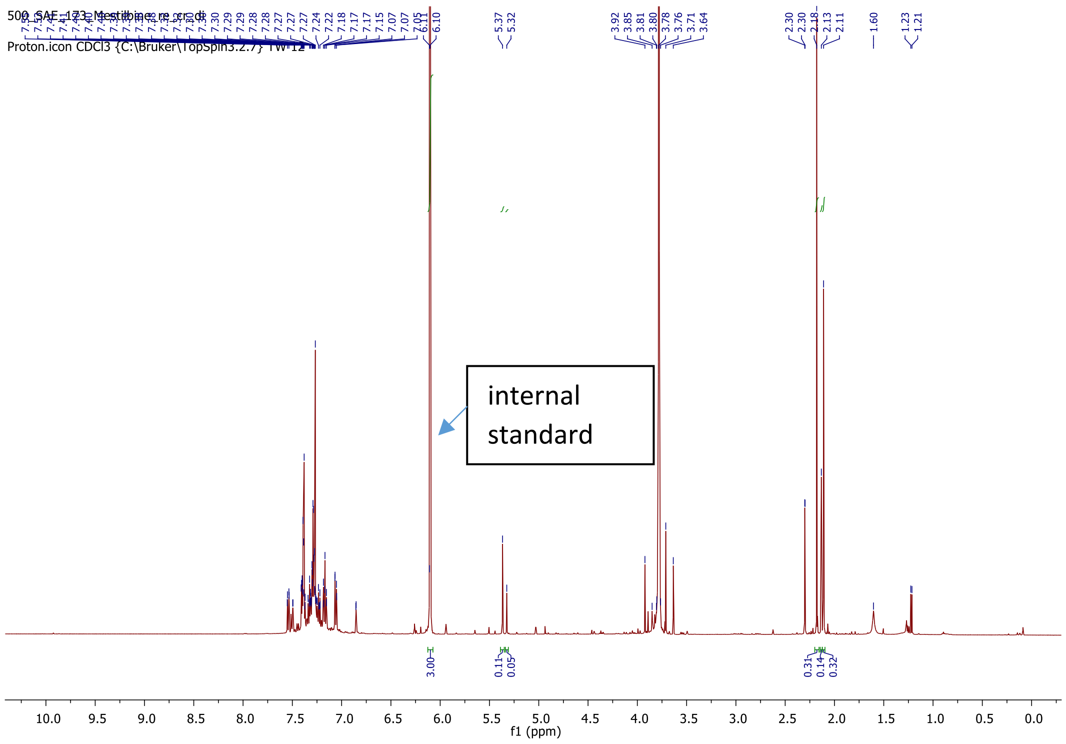

^{1}H NMR (500 MHz, $CDCl_3$)

iii.

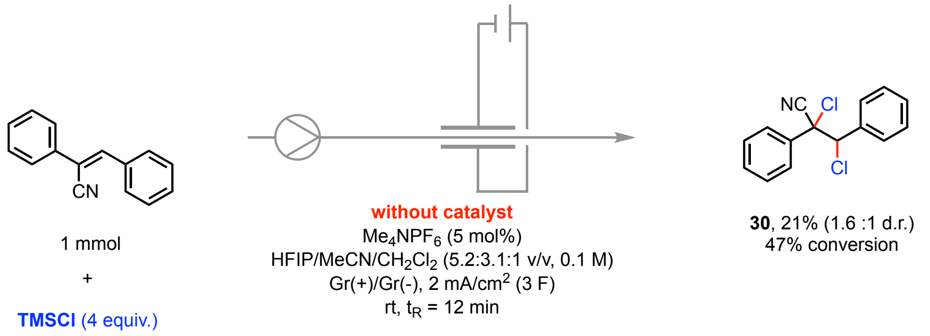

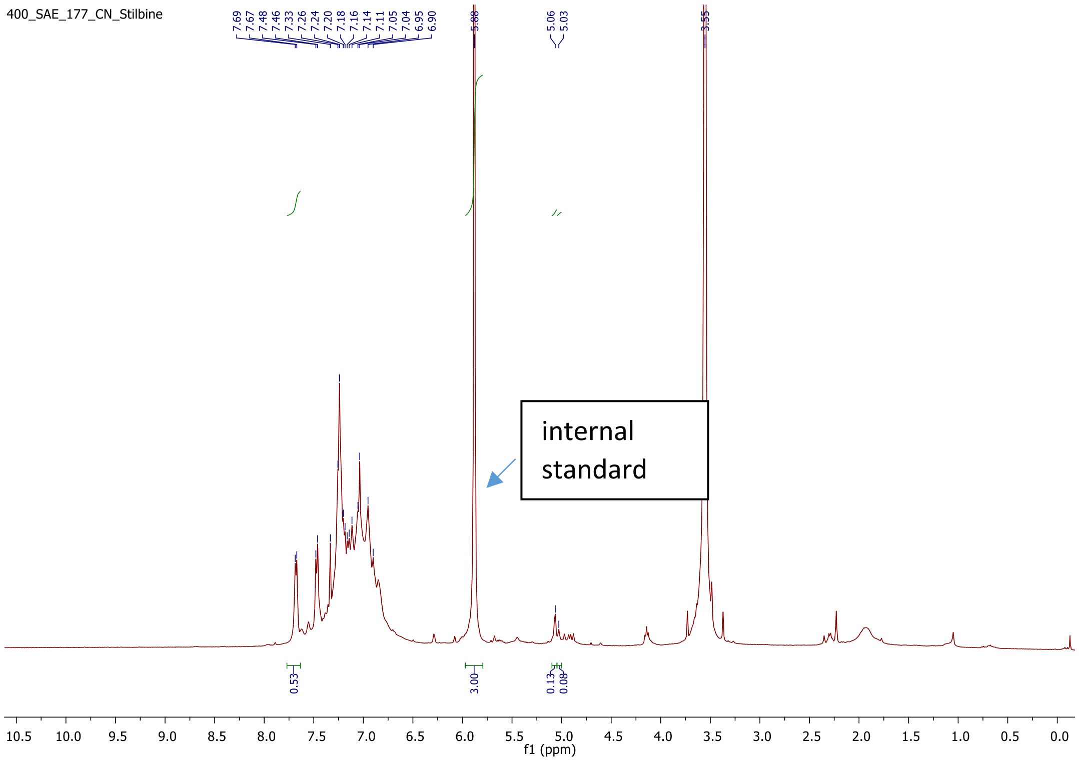

^1H NMR (400 MHz, $CDCl_3$)

Cyclic Voltammetry Investigation

While the onset potential of TMSCl oxidation was slightly lower compared to 4-iodotoluene, but the rate of oxidation was found to be rather slow compared to 4-iodotoluene oxidation. A lower onset potential for 4-iodotoluene oxidation in the presence of TMSCl could be explained by the facile oxidative generation of the hypervalent iodine intermediate (left graph).

a

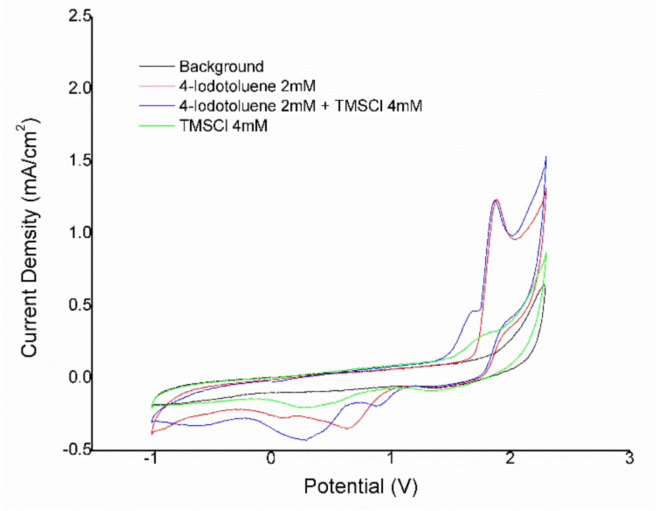

b

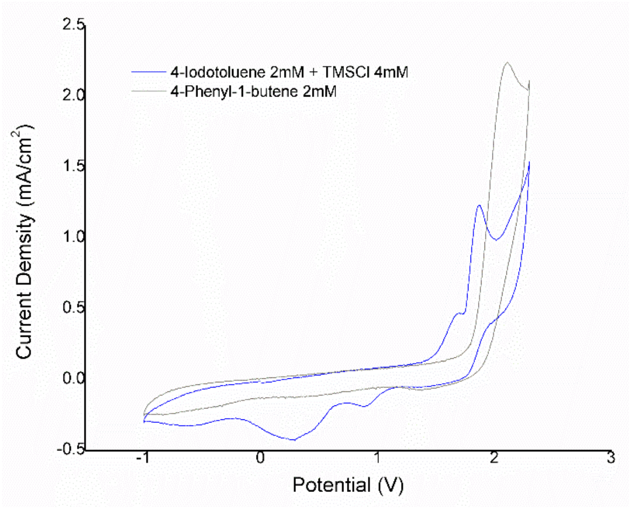

Supplementary Figure 7. a CV of 4-iodotoluene, TMSCl and their combination. **b** CV of 4-iodotoluene with TMSCl and alkene.

2.4. Ex-cell dichlorination of alkene with *in situ* generated $ArICl_2$

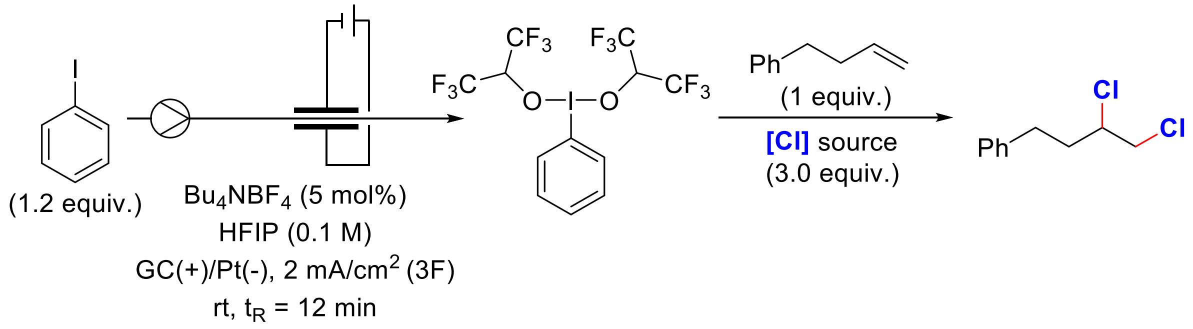

Supplementary Table 2. Variation of chloride source for dichlorination with stoichiometric $ArICl_2$.

Entry	Chloride source	Yield (%)
1	TMSCl	56
2	KCl	-
3	$MgCl_2$	61
4	LiCl	52
5	Et_4NCl	30
6	EtOCOCl	-

2.5. Optimisation for alkene dichlorination with catalytic 4-iodotoluene

Chloride salt optimisation

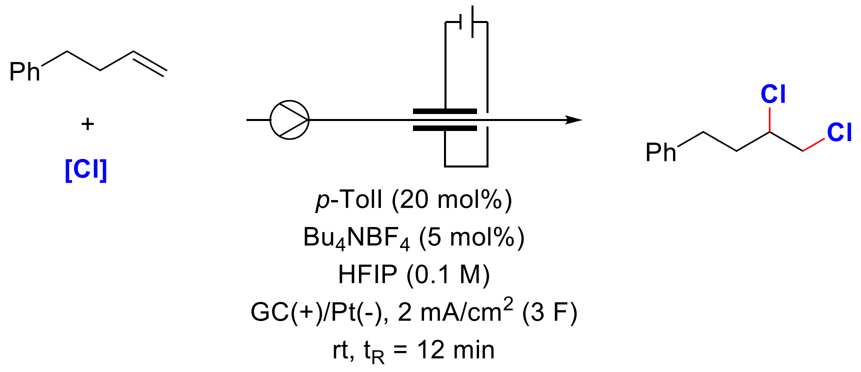

Supplementary Table 3. Variation of chloride source.

Entry	Chloride source (3 equiv.)	Yield (%)
1	$MgCl_2$	-
2	TMSCl	34 (6)[a]
3	LiCl	16
4	Et_4NCl	-

5	TMSCl (4 equiv.)	46

[a] Uncorrected yield of the side product arising from the nucleophilic attack of HFIP.

Solvent optimisation

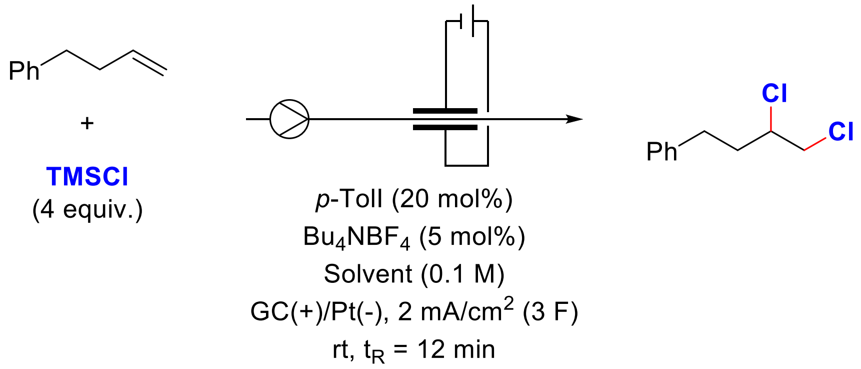

Supplementary Table 4. Screening of solvent.

Entry	Solvent	Yield (%)
1	HFIP	46
2	TFE	29
3	HFIP/MeCN (3:1)	47
4	HFIP/MeCN (2:1)	49
5	HFIP/MeCN (1:1)	29

Electrolyte optimisation

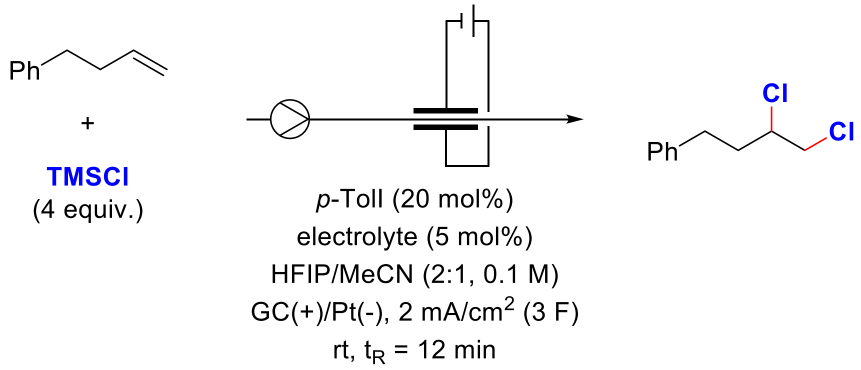

Supplementary Table 5. Screening of electrolyte.

Entry	Electrolyte	Yield (%)
1	Bu_4NBF_4	49
2	$LiClO_4$	38
3	Me_4NPF_6	71
4	-	41

Catalyst loading optimisation

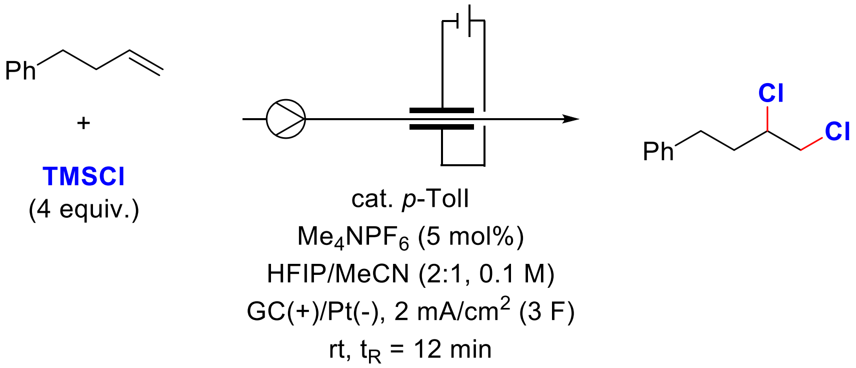

Supplementary Table 6. Amount of 4-iodotoluene.

Entry	4-TolI (mol%)	Yield (%)
1	20	71
2	25	78
3	30	81

Screening of electrode materials

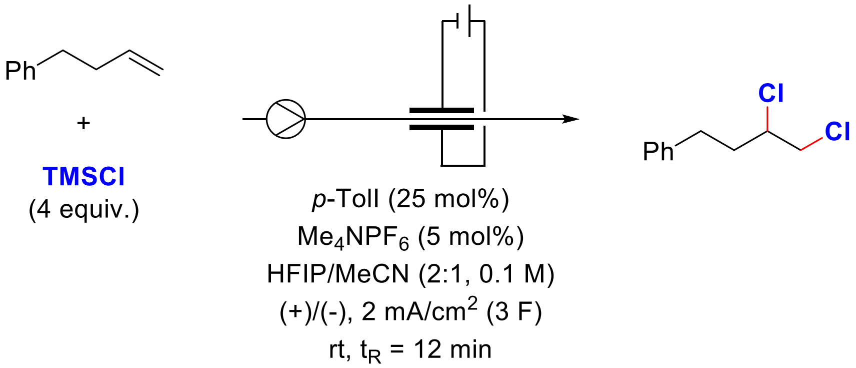

Supplementary Table 7. Screening of electrode materials.

Entry	Anode	Cathode	Yield (%)
1	Glassy carbon (GC)	Platinum (Pt)	78
2	Graphite (Gr)	Platinum (Pt)	75
3	Graphite (Gr)	Graphite (Gr)	82

2.6. Synthesis and characterisation data for starting materials

General procedure (GP1) for the preparation of esters

Carboxylic acid esters were prepared following previously reported procedure[6]. To a solution of alcohol (5.0 mmol) and carboxylic acid (5.0 mmol) in dry CH_2Cl_2 (25 mL), DMAP (10 mol%, 0.5 mmol) and EDCI•HCl (12.5 mmol) was added. The mixture was stirred at room temperature under inert atmosphere until the reaction was complete as observed from TLC monitoring. The mixture was diluted with distilled water (50 mL)

and the CH_2Cl_2 layer was separated, dried over anhydrous $MgSO_4$ and concentrated. The crude mixture was purified by flash column chromatography.

General procedure (GP2) for the preparation of allyl ethers

The procedure is analogous to a reported literature procedure[7]. To a solution of the alcohol (1 equiv.) in allyl bromide (1 equiv.) was added KOH (1.9 equiv.), and tetrabutylammonium bisulfate (20 mol%). The mixture was stirred at room temperature until the reaction was shown to be complete by TLC analysis. Water (20 mL) was then added, and the aqueous layer was extracted with diethyl ether (3 × 20 mL). The combined organic layers were washed once with water (30 mL), and once with brine (30 mL) before being dried over $MgSO_4$, filtered, and concentrated in vacuo to afford the crude product. The crude product was purified by silica gel flash column chromatography.

Pent-4-en-1-yl benzoate (S7):

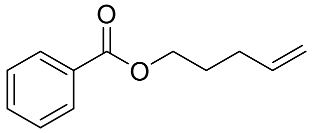

Synthesised by following **GP1** using benzoic acid (610 mg, 5 mmol) and 4-penten-1-ol (516 µL, 5 mmol). The crude mixture was purified by column chromatography to obtain the pure ester as colorless liquid (656 mg, 69%).

R_f (hexane:EtOAc 19:1) = 0.4.

^{1}H NMR (500 MHz, $CDCl_3$): δ 8.08 – 8.03 (m, 2H), 7.59 – 7.53 (m, 1H), 7.47 – 7.41 (m, 2H), 5.86 (ddt, *J* = 16.9, 10.2, 6.6 Hz, 1H), 5.12 – 4.98 (m, 2H), 4.35 (t, *J* = 6.6 Hz, 2H), 2.26 – 2.18 (m, 2H), 1.94 – 1.84 (m, 2H).

^{13}C NMR (126 MHz, $CDCl_3$): δ 166.7, 137.6, 133.0, 130.6, 129.7, 128.5, 115.5, 77.2, 64.5, 30.3, 28.1.

HRMS (ESI): m/z calculated for [$C_{12}H_{15}O_2^+$] [M+H$^+$]: 191.1067, measured: 191.1066.

But-3-en-1-yl benzoate (S8):

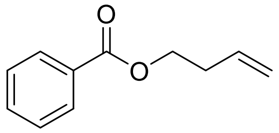

Synthesised by following **GP1** using benzoic acid (610 mg, 5 mmol) and 3-buten-1-ol (430 µL, 5 mmol). The crude mixture was purified by column chromatography to obtain the pure ester as colorless liquid (582 mg, 66%).

R_f (hexane:EtOAc 19:1) = 0.4.

^{1}H NMR (500 MHz, $CDCl_3$): δ 8.07 – 8.01 (m, 2H), 7.58 – 7.52 (m, 1H), 7.47 – 7.41 (m, 2H), 5.88 (ddt, *J* = 17.0, 10.3, 6.7 Hz, 1H), 5.18 (ddd, *J* = 17.0, 3.3, 1.4 Hz, 1H), 5.14 – 5.09 (m, 1H), 4.38 (t, *J* = 6.7 Hz, 2H), 2.53 (qt, *J* = 6.7, 1.4 Hz, 2H).

^{13}C NMR (101 MHz, $CDCl_3$): δ 166.6, 134.2, 133.0, 130.4, 129.7, 128.4, 117.4, 64.1, 33.3, 27.0.

HRMS (ESI): m/z calculated for [$C_{11}H_{13}O_2^+$] [M+H$^+$]: 177.0910, measured: 177.0914.

2-Cyanoethyl pent-4-enoate (S9):

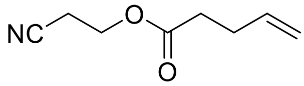

Synthesised by following **GP1** using 4-pentenoic acid (510 µL, 5 mmol) and 3-hydroxypropionitrile (342 µL, 5 mmol). The crude mixture was purified by column chromatography to obtain the pure ester as colorless liquid (551 mg, 72%).

R*f* (hexane:EtOAc 4:1) = 0.6.

^{1}H NMR (300 MHz, $CDCl_3$): δ 5.80 (ddt, *J* = 16.3, 10.2, 6.1 Hz, 1H), 5.12 – 4.94 (m, 2H), 4.26 (t, *J* = 6.3 Hz, 2H), 2.68 (t, *J* = 6.3 Hz, 2H), 2.51 – 2.28 (m, 4H).

^{13}C NMR (75 MHz, $CDCl_3$): δ 172.5, 136.3, 116.9, 115.9, 58.7, 33.2, 28.7, 18.1.

HRMS (ESI): m/z calculated for [$C_8H_{12}NO_2^+$] [M+H$^+$]: 154.0863, measured: 154.0856.

Benzyl pent-4-enoate (S10)[8]**:**

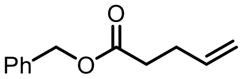

Pent-4-enoic acid (1.0 g, 5 mmol), benzyl bromide(1.4 mL, 12 mmol), anhydrous potassium carbonate (6.9 g, 50 mmol),and tetrabutylammonium iodide (250 mg) were combined in anhydrous acetone (20 mL) and stirred overnight at room temperature. The reaction mixture was filtered, and the solvent removed. The residue was taken up in EtOAc, washed (1N HCl, saturated $NaHCO_3$, saturated NaCl) and dried ($MgSO_4$). Purification by flash chromatography (1:30 CH_2Cl_2/hexane then 1:9 CH_2Cl_2/hexane) gave compound benzyl 4-pentenoate **S10** as a colorless liquid (1.63 g, 86 %).

R*f* (hexane:CH_2Cl_2 9:1) = 0.4.

^{1}H NMR (400 MHz, $CDCl_3$): δ 7.41 – 7.30 (m, 5H), 5.83 (ddt, *J* = 16.3, 10.2, 6.2 Hz, 1H), 5.14 (s, 2H), 5.07 (dd, *J* = 17.2, 1.6 Hz, 1H), 5.01 (dd, *J* = 10.3, 1.5 Hz, 1H), 2.51 – 2.45 (m, 2H), 2.45 – 2.37 (m, 2H).

^{13}C NMR (101 MHz, $CDCl_3$): δ 173.0, 136.7, 136.1, 128.7, 128.3, 115.7, 66.3, 33.7, 29.0.

GCMS: m/z calculated for [$C_{12}H_{14}O_2$]: 190.09883, measured: 190.0987

1-((Allyloxy)methyl)-2-bromobenzene (S13):

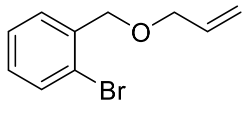

This compound was prepared by following general procedure **GP2** for a previous publication from our group. Detailed experimental procedure and the characterisation data are reported there[9].

But-3-en-1-yl 2-iodobenzoate (S14):

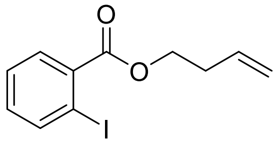

Synthesised by following **GP1** using 2-iodobenzoic acid (1.24 g, 5 mmol) and 3-buten-1-ol (429 µL, 5 mmol). The crude mixture was purified by column chromatography to obtain the pure ester as colorless oil (1.18 g, 78%).

$\mathbf{R}_f$ (hexane:CH_2Cl_2 1:1) = 0.8.

^{1}H NMR (300 MHz, $CDCl_3$): δ 7.98 (dd, *J* = 7.9, 0.9 Hz, 1H), 7.78 (dd, *J* = 7.8, 1.7 Hz, 1H), 7.39 (td, *J* = 7.7, 1.1 Hz, 1H), 7.13 (td, *J* = 7.8, 1.7 Hz, 1H), 5.87 (ddt, *J* = 17.0, 10.2, 6.7 Hz, 1H), 5.24 – 5.06 (m, 2H), 4.39 (t, *J* = 6.7 Hz, 2H), 2.54 (qt, *J* = 6.7, 1.3 Hz, 2H).

^{13}C NMR (75 MHz, $CDCl_3$): δ 166.6, 141.4, 135.4, 134.0, 132.7, 131.0, 128.0, 117.6, 94.2, 64.8, 33.1.

HRMS (ESI): m/z calculated for [$C_{11}H_{12}O_2{}^{127}I^+$] [M+H$^+$]: 302.9882, measured: 302.9892.

Hex-5-en-1-yl but-2-ynoate (S15):

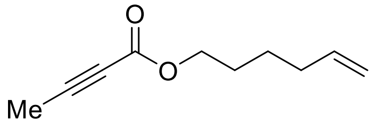

Synthesised by following **GP1** using 2-butynoic acid (420 mg, 5 mmol) and 5-hexen-1-ol (600 µL, 5 mmol). The crude mixture was purified by column chromatography to obtain the pure ester as colorless oil (631 mg, 76%).

$\mathbf{R}_f$ (hexane:CH_2Cl_2 3:1) = 0.4.

^{1}H NMR (300 MHz, $CDCl_3$): δ 5.77 (ddt, *J* = 16.9, 10.2, 6.7 Hz, 1H), 5.09 – 4.87 (m, 2H), 4.14 (t, *J* = 6.6 Hz, 2H), 2.07 (td, *J* = 7.2, 1.2 Hz, 2H), 1.97 (s, 3H), 1.67 (dq, *J* = 8.5, 6.8 Hz, 2H), 1.53 – 1.38 (m, 2H).

^{13}C NMR (75 MHz, $CDCl_3$): δ 154.0, 138.3, 115.0, 85.5, 72.6, 65.8, 33.3, 27.9, 25.2, 3.9.

HRMS (ESI): m/z calculated for [$C_{10}H_{15}O_2^+$] [M+H$^+$]: 167.1067, measured: 167.1071.

(Pent-4-en-1-ylsulfonyl)benzene (S16):

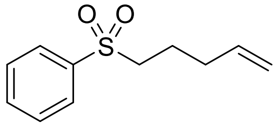

Following a reported literature procedure[9], to a stirred solution of $NaSO_2Ph$ (985 mg, 6 mmol) in DMF (5 mL) at room temperature were added TBAI (185 mg, 0.5 mmol) and 5-bromo-1-pentene (592 µL, 5 mmol), and the reaction mixture was heated to 60 °C. After 5 h, the reaction was quenched with sat. aq NaCl (30 mL) and extracted with EtOAc (3 × 20 mL). The combined organic layer was washed with sat. aq NaCl (3 × 10 mL). The dried ($MgSO_4$) extract was concentrated in vacuo and purified by flash chromatography to give **S16** as a colorless oil (925 mg, 88%).

R_f (hexane:EtOAc 4:1) = 0.5.

^{1}H NMR (300 MHz, $CDCl_3$): δ 7.95 – 7.84 (m, 2H), 7.70 – 7.60 (m, 1H), 7.59 – 7.48 (m, 2H), 5.75 – 5.56 (m, 1H), 5.03 – 4.92 (m, 2H), 3.13 – 3.01 (m, 2H), 2.11 (q, *J* = 7.1 Hz, 2H), 1.87 – 1.74 (m, 2H).

^{13}C NMR (75 MHz, $CDCl_3$): δ 139.3, 136.4, 133.8, 129.4, 128.1, 116.6, 55.6, 32.1, 21.9.

HRMS (ESI): m/z calculated for [$C_{11}H_{15}O_2{}^{32}S^+$] [M+H$^+$]: 211.0787, measured: 211.0787.

Hex-5-en-1-yl diphenyl phosphate (S17):

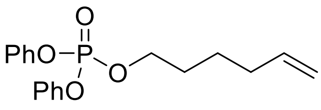

This compound was prepared according to a reported procedure[2]. 5-Hexen-1-ol (1.2 mL, 10.0 mmol) and NEt_3 (2.78 mL, 20.0 mmol) were diluted in dry tetrahydrofuran (25 mL) and the resulting solution was cooled to 0°C. Diphenyl phosphoryl chloride (3.1 mL, 15.0 mmol) was added dropwise into the reaction mixture which was allowed to warm to ambient temperature and was stirred for 24 h. The reaction mixture was diluted with ethyl acetate and water, and the aqueous layer was extracted with ethyl acetate (3 × 20 mL). The combined organic layers were washed with water and brine, dried over $MgSO_4$, filtered and evaporated under vacuo. The crude mixture was purified by column chromatography to afford **S17** as a light yellow oil (2.36 g, 71%).

R_f (hexane:CH_2Cl_2 1:1) = 0.4.

^{1}H NMR (300 MHz, $CDCl_3$): δ 7.34 (dd, *J* = 8.4, 7.5 Hz, 4H), 7.25 – 7.14 (m, 6H), 5.75 (ddt, *J* = 16.9, 10.2, 6.7 Hz, 1H), 5.05 – 4.90 (m, 2H), 4.26 (dd, *J* = 13.9, 6.5 Hz, 2H), 2.05 (dd, *J* = 14.2, 7.2 Hz, 2H), 1.81 – 1.62 (m, 2H), 1.56 – 1.38 (m, 2H).

^{13}C NMR (75 MHz, $CDCl_3$): δ 150.8, 150.7, 138.2, 129.9, 125.4, 120.2, 120.2, 115.1, 69.4, 69.3, 33.2, 29.7, 29.6, 24.7.

HRMS (ESI): m/z calculated for [$C_{18}H_{22}O_4P^+$] [M+H$^+$]: 333.1256, measured: 333.1256.

But-3-en-1-yl pyrazine-2-carboxylate (S18):

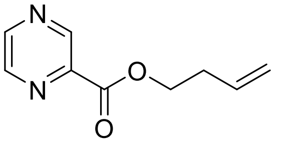

Synthesised by following **GP1** using 2-pyrazine carboxylic acid (621 mg, 5 mmol) and 3-buten-1-ol (429 µL, 5 mmol). The crude mixture was purified by column chromatography to obtain the pure ester as white gum (499 mg, 56%).

R_f (hexane:EtOAc 1:1) = 0.8.

^{1}H NMR (300 MHz, $CDCl_3$): δ 9.23 (d, *J* = 0.9 Hz, 1H), 8.69 (dd, *J* = 8.8, 1.8 Hz, 2H), 5.80 (ddt, *J* = 17.0, 10.2, 6.7 Hz, 1H), 5.18 – 5.00 (m, 2H), 4.44 (t, *J* = 6.8 Hz, 2H), 2.52 (q, *J* = 6.8 Hz, 2H).

^{13}C NMR (75 MHz, CDCl$_3$): δ 163.8, 147.6, 146.3, 144.5, 143.5, 133.4, 117.8, 65.2, 33.0.

HRMS (ESI): m/z calculated for [$C_9H_{11}N_2O_2^+$] [M+H$^+$]: 179.0821, measured: 179.0821.

2-((allyloxy)methyl)pyridine (S19):

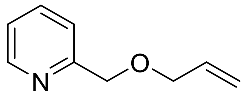

This compound was prepared by following general procedure **GP2** for a previous publication from our group. Detailed experimental procedure and the characterisation data are reported there[9].

But-3-en-1-yl furan-2-carboxylate (S20):

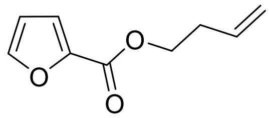

Synthesised by following **GP1** using 2-furoic acid (561 mg, 5 mmol) and 3-buten-1-ol (429 µL, 5 mmol). The crude mixture was purified by column chromatography to obtain the pure ester as colorless oil (507 mg, 61%).

R*f* (hexane:EtOAc 4:1) = 0.8.

^{1}H NMR (300 MHz, CDCl$_3$): δ 7.61 – 7.51 (m, 1H), 7.15 (dd, *J* = 3.5, 0.6 Hz, 1H), 6.49 (dd, *J* = 3.5, 1.7 Hz, 1H), 5.83 (ddt, *J* = 17.0, 10.2, 6.7 Hz, 1H), 5.23 – 5.02 (m, 2H), 4.34 (t, *J* = 6.8 Hz, 2H), 2.49 (qd, *J* = 6.8, 1.2 Hz, 2H).

^{13}C NMR (75 MHz, CDCl$_3$): δ 158.8, 146.4, 144.8, 133.8, 118.0, 117.6, 111.9, 64.0, 33.2.

HRMS (ESI): m/z calculated for [$C_9H_{11}O_3^+$] [M+H$^+$]: 167.0708, measured: 167.0709.

(1*S*,2*R*,4*S*)-2-(Allyloxy)-1,3,3-trimethylbicyclo[2.2.1]heptane (S21):

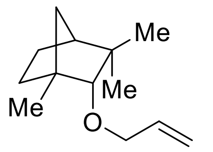

To a solution of (+)-fenchol (771 mg, 5 mmol) in dry THF, NaH (60% in mineral oil, 500 mg, 12.5 mmol) was added portion wise at 0 °C under inert atmosphere and stirred for 15 min. Then, allyl bromide (864 µL, 10 mmol) was dropwise while maintaining the temperature at 0 °C. The mixture was stirred at room temperature until the reaction was shown to be complete by TLC analysis. Water (20 mL) was then added, and the aqueous layer was extracted with diethyl ether (3 × 20 mL). The combined organic layers were washed once with 1 N HCl (30 mL), and once with brine (30 mL) before being dried over $MgSO_4$, filtered, and concentrated in vacuo to afford the crude product. The crude product was purified by silica gel flash column chromatography to provide **S21** as colorless liquid (592 mg, 61%).

R*f* (hexane:CH_2Cl_2 3:1) = 0.8.

^{1}H NMR (400 MHz, CDCl$_3$): δ 5.95 – 5.81 (m, 1H), 5.26 (ddd, *J* = 17.2, 3.6, 1.8 Hz, 1H), 5.17 – 5.07 (m, 1H), 4.10 – 3.99 (m, 1H), 3.95 – 3.84 (m, 1H), 2.94 (d, *J* = 1.8 Hz,

1H), 1.78 – 1.64 (m, 2H), 1.64 – 1.60 (m, 1H), 1.46 – 1.33 (m, 2H), 1.08 (s, 3H), 1.08 – 1.05 (m, 1H), 1.02 (s, 3H), 1.00 – 0.94 (m, 1H), 0.91 (s, 3H).

^{13}C NMR (75 MHz, $CDCl_3$): δ 135.9, 116.0, 92.4, 72.7, 49.2, 48.9, 41.6, 39.5, 31.8, 26.3, 26.1, 20.9, 20.2.

HRMS (ESI): m/z calculated for [$C_{13}H_{23}O^+$] [$M+H^+$]: 195.1743, measured: 195.1741.

(*E*)-Hex-4-en-1-yl-4-methylbenzenesulfonate (S26):

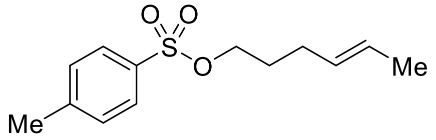

To a cooled solution (ice-water bath) of (*E*)-4-hexen-1-ol (584 µL, 5 mmol) and *p*-toluenesulfonylchloride (1.048 g, 5.5 mmol) in dry CH_2Cl_2 (25 mL), DMAP (61 mg, 0.5 mmol) and triethylamine (1.4 mL, 10 mmol) were added under inert atmosphere. The mixture was stirred at room temperature until the reaction was complete as observed from TLC monitoring. The mixture was diluted with distilled water (50 mL) and the CH_2Cl_2 layer was separated, dried over anhydrous $MgSO_4$ and concentrated. Purification by flash column chromatography to provided **S26** as colorless liquid (865 mg, 68%).

R_f (hexane:EtOAc 4:1) = 0.8.

^{1}H NMR (300 MHz, $CDCl_3$): δ 7.78 (d, *J* = 8.3 Hz, 2H), 7.34 (d, *J* = 8.1 Hz, 2H), 5.42 – 5.16 (m, 2H), 4.00 (t, *J* = 6.4 Hz, 2H), 2.44 (s, 3H), 1.99 (dt, *J* = 13.1, 4.5 Hz, 2H), 1.73 – 1.63 (m, 2H), 1.56 (dd, *J* = 7.7, 6.9 Hz, 3H).

^{13}C NMR (75 MHz, $CDCl_3$): δ 144.8, 133.2, 129.9, 129.1, 128.0, 126.6, 70.0, 28.6, 28.3, 21.7, 18.0.

HRMS (ESI): m/z calculated for [$C_{13}H_{19}O_3{}^{32}S^+$] [$M+H^+$]: 255.1049, measured: 255.1050.

(*Z*)-Hex-4-en-1-yl 4-methylbenzenesulfonate (S27):

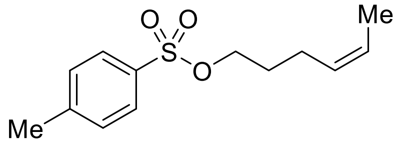

To a cooled solution (ice-water bath) of (*Z*)-4-hexen-1-ol (584 µL, 5 mmol) and *p*-toluenesulfonylchloride (1.048 g, 5.5 mmol) in dry CH_2Cl_2 (25 mL), DMAP (61 mg, 0.5 mmol) and triethylamine (1.4 mL, 10 mmol) were added under inert atmosphere. The mixture was stirred at room temperature until the reaction was complete as observed from TLC monitoring. The mixture was diluted with distilled water (50 mL) and the CH_2Cl_2 layer was separated, dried over anhydrous $MgSO_4$ and concentrated. The crude mixture was purified by flash column chromatography to give **S27** as colorless liquid (839 mg, 66%).

R_f (hexane:EtOAc 8:1) = 0.3.

^{1}H NMR (300 MHz, $CDCl_3$): δ 7.77 (d, *J* = 8.3 Hz, 2H), 7.33 (d, *J* = 8.0 Hz, 2H), 5.56 – 5.34 (m, 1H), 5.34 – 5.12 (m, 1H), 4.01 (t, *J* = 6.4 Hz, 2H), 2.43 (s, 3H), 2.14 – 2.00 (m, 2H), 1.68 (dq, *J* = 13.5, 6.6 Hz, 2H), 1.58 – 1.48 (m, 3H).

^{13}C NMR (75 MHz, $CDCl_3$): δ 144.8, 133.2, 129.9, 128.3, 127.9, 125.6, 70.1, 28.7, 22.7, 21.7, 12.8.

HRMS (ESI): m/z calculated for [$C_{13}H_{19}O_3{}^{32}S^+$] [$M+H^+$]: 255.1049, measured: 255.1055.

(*E*)-Hex-4-en-1-yl 4-chlorobenzoate (S28):

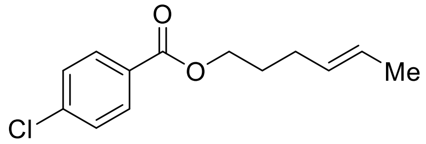

Synthesised by following **GP1** using 4-chlorobenzoic acid (783 mg, 5 mmol) and *trans*-4-hexen-1-ol (584 µL, 5 mmol). The crude mixture was purified by column chromatography to obtain the pure ester as colorless oil (799 mg, 67%).

R_f (hexane:CH_2Cl_2 1:1) = 0.8.

^{1}H NMR (300 MHz, $CDCl_3$): δ 8.02 – 7.93 (m, 2H), 7.45 – 7.36 (m, 2H), 5.55 – 5.37 (m, 1H), 4.31 (td, *J* = 6.5, 2.4 Hz, 1H), 2.22 – 2.08 (m, 1H), 1.87 – 1.77 (m, 1H), 1.68 – 1.61 (m, 1H).

^{13}C NMR (75 MHz, $CDCl_3$): δ 165.9, 139.4, 131.0, 130.0, 129.0, 128.8, 126.1, 64.8, 29.1, 28.6, 18.0.

HRMS (ESI): m/z calculated for [$C_{13}H_{16}{}^{35}ClO_2{}^+$] [$M+H^+$]: 239.0833, measured: 239.0836.

(*Z*)-Hex-4-en-1-yl 4-chlorobenzoate (S29):

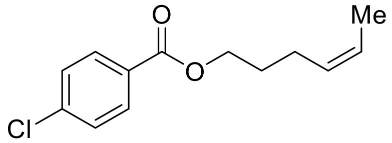

Synthesised by following **GP1** using 4-chlorobenzoic acid (783 mg, 5 mmol) and *cis*-4-hexen-1-ol (584 µL, 5 mmol). The crude mixture was purified by column chromatography to obtain the pure ester as colorless oil (728 mg, 61%).

R_f (hexane:CH_2Cl_2 2:1) = 0.4.

^{1}H NMR (300 MHz, $CDCl_3$): δ 8.03 – 7.91 (m, 2H), 7.50 – 7.33 (m, 2H), 5.62 – 5.29 (m, 2H), 4.31 (t, *J* = 6.6 Hz, 2H), 2.20 (q, *J* = 7.2 Hz, 2H), 1.88 – 1.77 (m, 2H), 1.60 (dd, *J* = 6.3, 1.0 Hz, 3H).

^{13}C NMR (75 MHz, $CDCl_3$): δ 165.9, 139.4, 131.0, 129.1, 129.0, 128.8, 125.2, 64.8, 28.6, 23.4, 12.8.

HRMS (ESI): m/z calculated for [$C_{13}H_{16}{}^{35}ClO_2{}^+$] [$M+H^+$]: 239.0833, measured: 239.0834.

2-Phenylpent-4-enoic acid (S34):

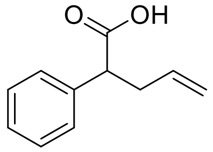

This compound was prepared for a previous publication from our group. Detailed experimental procedure and the characterisation data can be found in that report[9].

2.7. General procedure (GP3) for the dichlorination of alkenes

4-Iodotoluene (55 mg, 0.25 mmol, 0.25 equiv.) and Me_4NPF_6 (11 mg, 0.05 mmol, 0.05 equiv.) were added to an oven-dried vial equipped with a Teflon-coated magnetic stir bar. Dry MeCN (3.1 mL), respective alkene (1.0 mmol, 1.0 equiv.) and TMSCl (507 µL, 4.0 mmol, 4.0 equiv.) were added sequentially and the reaction mixture was pre-stirred until a clear solution was obtained. Dry HFIP (~ 6.2 mL) was added to the reaction mixture to reach 10 mL volume (0.1 M). The mixture was stirred until homogeneous and placed in a 12 ml disposable syringe. The solution was pumped through the electrochemical setup with a fixed flowrate of 0.05 mL/min to give a residence time of 12 minutes in the active part of the reactor, equipped with graphite electrodes separated by 0.6 mm FEP spacer. The reaction mixture was subjected to a constant current electrolysis by applying 24 mA current (current density of 2 mA/cm^2 with electrode surface area of 12 cm^2). This flow rate and concentration delivered 3 F per mole of charge to the reaction mixture. The first 1.5 reactor volume (0.9 mL) was discarded to reach an equilibrium. After which, the reaction output was collected in a vial for 100 minutes (5 mL) and then the reaction was stopped after the collection vial was removed. The power supply was turned off and the reactor was washed by passing MeOH and acetone. A 5 µL aliquot from this crude reaction mixture was analysed by GC/MS. The rest of the reaction mixture was concentrated, and the residue was purified by flash column chromatography to afford the desired product.

2.8. General procedure (GP4) for dichlorination of alkenes and monochlorination of 1,3-dicarbonyl compounds in non-electrolysis conditions

Dichloro(*p*-tolyl)-λ^3-iodane (1.5 equiv.) was added to an oven-dried vial equipped with a teflon-coated magnetic stir bar. To this, dry CH_2Cl_2 or dry CH_2Cl_2 : HFIP (1:1, 2 mL, 0.25 M) was added, followed by the respective alkene or 1,3-dicarbonyl compound (0.5 mmol, 1.0 equiv.) and the trapping reagent (BHT or TEMPO; 1.0 mmol, 2.0 equiv.). The reaction mixture was stirred at ambient temperature for 12 h. The solvent was then evaporated under reduced pressure. The residue was dissolved in 0.6 mL of $CDCl_3$ containing 0.5 mmol of 1,3,5-trimethoxybenzene or 1,2-dibromomethane (as an internal standard), and analysed by ^1H NMR. Subsequently, the sample was evaporated under reduced pressure and purified by column chromatography.

2.9. Characterization data for the products

(3,4-Dichlorobutyl)benzene (5):

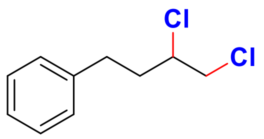

Following **GP3** with but-3-en-1-ylbenzene, 5 mL reaction mixture (0.5 mmol) was collected after flow electrolysis. The volatile solvent was removed *in vacuo* and the residue was purified by column chromatography yielding the product as a colorless oil (81 mg, 80%).

R_f (hexane) = 0.6.

^{1}H NMR (300 MHz, CDCl$_3$): δ 7.16 (dd, *J* = 10.1, 4.4 Hz, 2H), 7.10 – 7.03 (m, 3H), 3.90 – 3.79 (m, 1H), 3.62 (dd, *J* = 11.3, 5.1 Hz, 1H), 3.55 – 3.47 (m, 1H), 2.77 (ddd, *J* = 13.9, 9.1, 5.0 Hz, 1H), 2.61 (ddd, *J* = 13.9, 8.6, 7.6 Hz, 1H), 2.16 (dddd, *J* = 14.3, 9.1, 7.6, 3.3 Hz, 1H), 1.95 – 1.81 (m, 1H).

^{13}C NMR (75 MHz, CDCl$_3$): δ 140.5, 128.7, 128.6, 126.4, 60.4, 48.4, 36.8, 32.1.

HRMS (ESI): m/z calculated for [$C_{10}H_{12}{}^{35}Cl_2^+$] [$M^+$]: 202.0311, measured: 202.0308.

The analytical data are in accordance with reported literature[10].

1,2-Dichlorodecane (6):

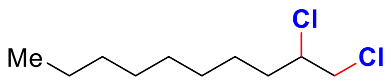

Following **GP3** with 1-decene, 5 mL reaction mixture (0.5 mmol) was collected after flow electrolysis. The volatile solvent was removed *in vacuo* and the residue was purified by column chromatography yielding the product as a colorless liquid (81 mg, 77%).

R_f (hexane) = 0.8.

^{1}H NMR (300 MHz, CDCl$_3$): δ 4.11 – 3.97 (m, 1H), 3.76 (dd, *J* = 11.3, 5.2 Hz, 1H), 3.65 (dd, *J* = 11.3, 7.4 Hz, 1H), 1.98 (dddd, *J* = 14.1, 9.8, 5.6, 3.9 Hz, 1H), 1.79 – 1.62 (m, 1H), 1.61 – 1.47 (m, 1H), 1.47 – 1.38 (m, 1H), 1.37 – 1.21 (m, 10H), 0.88 (t, *J* = 6.7 Hz, 3H).

^{13}C NMR (75 MHz, CDCl$_3$): δ 61.4, 48.4, 35.2, 32.0, 29.5, 29.3, 29.1, 26.0, 22.8, 14.3.

The analytical data are in accordance with previously reported literature[11].

4,5-Dichloropentyl benzoate (7):

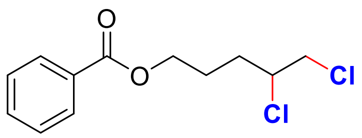

Following **GP3** with **S7**, 5 mL reaction mixture (0.5 mmol) was collected after flow electrolysis. The volatile solvent was removed *in vacuo* and the residue was purified by column chromatography yielding the product as a colorless liquid (93 mg, 71%).

R_f (hexane:CH_2Cl_2 4:1) = 0.3.

^{1}H NMR (300 MHz, $CDCl_3$): δ 8.04 (dd, *J* = 5.2, 3.3 Hz, 2H), 7.63 – 7.51 (m, 1H), 7.51 – 7.38 (m, 2H), 4.42 – 4.32 (m, 2H), 4.18 – 4.05 (m, 1H), 3.79 (dt, *J* = 11.1, 5.6 Hz, 1H), 3.72 – 3.62 (m, 1H), 2.26 – 2.15 (m, 1H), 2.14 – 2.03 (m, 1H), 2.00 – 1.80 (m, 2H).

^{13}C NMR (75 MHz, $CDCl_3$): δ 166.6, 133.1, 130.2, 129.7, 128.5, 64.1, 60.6, 48.1, 31.9, 25.4.

HRMS (ESI): m/z calculated for [$C_{12}H_{14}O_2{}^{35}Cl^+$] [M-Cl$^+$]: 225.0677, measured: 225.0677.

3,4-Dichlorobutyl benzoate (8):

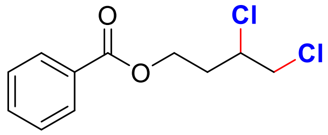

Following **GP3** with **S8**, 5 mL reaction mixture (0.5 mmol) was collected after flow electrolysis. The volatile solvent was removed *in vacuo* and the residue was purified by column chromatography yielding the product as a colorless liquid (95 mg, 77%).

R_f (hexane:CH_2Cl_2 4:1) = 0.3.

^{1}H NMR (300 MHz, $CDCl_3$): δ 8.07 – 8.01 (m, 2H), 7.58 (ddt, *J* = 6.7, 5.1, 1.4 Hz, 1H), 7.48 – 7.41 (m, 2H), 4.64 – 4.44 (m, 2H), 4.33 – 4.21 (m, 1H), 3.91 – 3.80 (m, 1H), 3.79 – 3.69 (m, 1H), 2.63 – 2.45 (m, 1H), 2.22 – 2.07 (m, 1H).

^{13}C NMR (75 MHz, $CDCl_3$): δ 166.4, 133.3, 130.0, 129.7, 128.6, 61.4, 57.6, 48.3, 34.4.

The analytical data are in accordance with reported literature[10].

2-Cyanoethyl 4,5-dichloropentanoate (9):

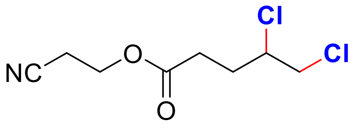

Following **GP3** with **S9**, 5 mL reaction mixture (0.5 mmol) was collected after flow electrolysis. The volatile solvent was removed *in vacuo* and the residue was purified by column chromatography yielding the product as a colorless liquid (86 mg, 79%).

R_f (hexane:EtOAc 4:1) = 0.5.

^{1}H NMR (300 MHz, $CDCl_3$): δ 4.36 – 4.25 (m, 2H), 4.19 – 4.06 (m, 1H), 3.80 (dd, *J* = 11.4, 4.9 Hz, 1H), 3.71 – 3.61 (m, 1H), 2.72 (t, *J* = 6.3 Hz, 2H), 2.68 – 2.51 (m, 2H), 2.40 (dddd, *J* = 15.5, 8.4, 7.1, 3.1 Hz, 1H), 2.08 – 1.91 (m, 1H).

^{13}C NMR (75 MHz, $CDCl_3$): δ 172.0, 116.8, 59.8, 59.0, 48.0, 30.4, 30.2, 18.1.

HRMS (ESI): m/z calculated for [$C_8H_{12}NO_2{}^{35}Cl_2{}^+$] [M+H$^+$]: 224.0240, measured: 224.0234.

Benzyl 4,5-dichloropentanoate (10):

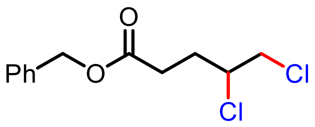

Following **GP3** with benzyl pent-4-enoate, 5 mL reaction mixture (0.5 mmol) was collected after flow electrolysis. The volatile solvent was removed *in vacuo* and the residue was purified by column chromatography yielding the product as a colorless liquid (60 mg, 46%).

R*f* (hexane:CH_2Cl_2 9:1) = 0.3.

^{1}H NMR (500 MHz, $CDCl_3$): δ 7.43 – 7.33 (m, 5H), 5.15 (d, *J* = 1.4 Hz, 2H), 4.13 (dddd, *J* = 9.8, 8.0, 5.0, 3.2 Hz, 1H), 3.78 (dd, *J* = 11.4, 5.0 Hz, 1H), 3.66 (dd, *J* = 11.4, 7.5 Hz, 1H), 2.71 – 2.52 (m, 2H), 2.42 (dddd, *J* = 14.6, 8.4, 7.2, 3.2 Hz, 1H), 2.00 (dddd, *J* = 14.6, 9.8, 8.1, 5.6 Hz, 1H).

^{13}C NMR (126 MHz, $CDCl_3$): δ 172.3, 135.8, 128.7, 128.5, 128.4, 66.6, 60.0, 48.1, 30.7, 30.4.

GCMS: m/z calculated for [$C_{12}H_{14}O_2{}^{35}Cl_2{}^+$] [$M+H^+$]: 260.03654, measured: 260.0364

4,5-Dichloropentanoic acid:

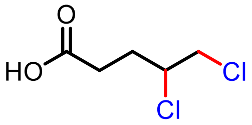

Following literature procedure[12], compound **10** (0.5 mmol, 130 mg) was dissolved in anhydrous CH_2Cl_2 (1 mL) under nitrogen. While stirring, $SnCl_4$ (0.5 mmol, 1 equiv) was added. The reaction vessel was then sealed and heated to 30 °C overnight. The reaction was quenched with HCl (1N, 1 mL) and then extracted with CH_2Cl_2. The combined organic layers were washed with brine and then dried ($MgSO_4$) and the product was purified through column chromatography, yielding the product as a colorless liquid with inseparable impurities (60 mg, 70%). This was further used in mechanistic study for cyclisation reaction under flow electrolysis conditions.

^{1}H NMR (400 MHz, $CDCl_3$): δ 4.22 – 4.10 (m, 1H), 3.81 (dd, *J* = 11.4, 4.9 Hz, 1H), 3.67 (dd, *J* = 11.4, 7.6 Hz, 1H), 2.78 – 2.53 (m, 2H), 2.50 – 2.36 (m, 1H), 2.07 – 1.92 (m, 1H).

^{13}C NMR (126 MHz, $CDCl_3$): δ 178.7, 59.8, 48.1, 30.5, 30.1.

GCMS: Provided EI on fragment minus the chlorines at 99, the molecular ion in any technique could not be observed.

9,10-Dichlorodecan-1-ol (11):

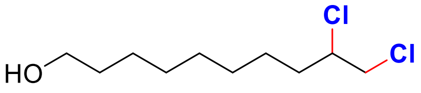

Following **GP3** with 9-decen-1-ol, 5 mL reaction mixture (0.5 mmol) was collected after flow electrolysis. The volatile solvent was removed *in*

vacuo and the residue was purified by column chromatography yielding the product as a colorless oil (35 mg, 31%).

R*f* (CH_2Cl_2) = 0.4.

^{1}H NMR (300 MHz, $CDCl_3$): δ 4.03 (ddd, *J* = 8.9, 6.9, 4.5 Hz, 1H), 3.76 (dd, *J* = 11.3, 5.2 Hz, 1H), 3.69 – 3.59 (m, 3H), 2.05 – 1.92 (m, 1H), 1.78 – 1.64 (m, 1H), 1.63 – 1.50 (m, 4H), 1.33 (s, 8H).

^{13}C NMR (75 MHz, $CDCl_3$): δ 63.2, 61.4, 48.4, 35.2, 32.9, 29.5, 29.4, 29.0, 25.9, 25.8.

HRMS (ESI): m/z calculated for [$C_{10}H_{21}O^{35}Cl_2^+$] [M+H$^+$]: 227.0964, measured: 227.0959.

10,11-Dichloroundecanoic acid (12):

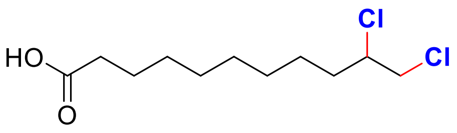

Following **GP3** with undecylenic acid, 5 mL reaction mixture (0.5 mmol) was collected after flow electrolysis. The volatile solvent was removed *in vacuo* and the residue was purified by column chromatography yielding the product as white solid (91 mg, 71%).

R*f* (hexane:EtOAc 3:2) = 0.6.

^{1}H NMR (300 MHz, $CDCl_3$): δ 4.10 – 3.96 (m, 1H), 3.76 (dd, *J* = 11.3, 5.2 Hz, 1H), 3.70 – 3.59 (m, 1H), 2.35 (t, *J* = 7.4 Hz, 2H), 2.05 – 1.88 (m, 1H), 1.81 – 1.50 (m, 5H), 1.32 (s, 8H).

^{13}C NMR (75 MHz, $CDCl_3$): δ 180.4, 61.3, 48.4, 35.2, 34.2, 29.3, 29.2, 29.1, 29.0, 25.9, 24.7.

The analytical data are in accordance with reported literature[2].

1-Bromo-2-((2,3-dichloropropoxy)methyl)benzene (13):

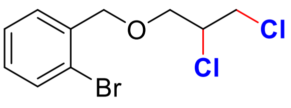

Following **GP3** with **S13**, 5 mL reaction mixture (0.5 mmol) was collected after flow electrolysis. The volatile solvent was removed *in vacuo* and the residue was purified by column chromatography yielding the product as a colorless oil (94 mg, 63%).

R*f* (hexane:CH_2Cl_2 19:1) = 0.3.

^{1}H NMR (300 MHz, $CDCl_3$): δ 7.55 (dt, *J* = 7.4, 3.7 Hz, 1H), 7.48 (dd, *J* = 7.6, 1.3 Hz, 1H), 7.34 (td, *J* = 7.4, 1.0 Hz, 1H), 7.17 (td, *J* = 7.6, 1.7 Hz, 1H), 4.67 (s, 2H), 4.28 – 4.18 (m, 1H), 3.96 – 3.80 (m, 4H).

^{13}C NMR (75 MHz, $CDCl_3$): δ 136.9, 132.8, 129.4, 129.3, 127.6, 122.9, 72.9, 70.9, 58.2, 45.4.

HRMS (ESI): m/z calculated for $[C_{10}H_{11}O^{35}Cl_2^+]$ $[M-Br^+]$: 217.0182, measured: 217.0181.

3,4-Dichlorobutyl 2-iodobenzoate (14):

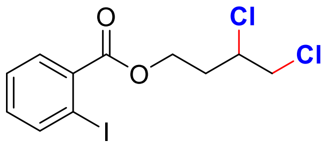

Following **GP3** with **S14**, 5 mL reaction mixture (0.5 mmol) was collected after flow electrolysis. The volatile solvent was removed *in vacuo* and the residue was purified by column chromatography yielding the product as a colorless gum (142 mg, 76%). A control experiment by following **GP1** in the absence of 4-TolI catalyst was also conduct and provided 127 mg (68%) of the same product.

R*f* (hexane:CH_2Cl_2 1:1) = 0.7.

^{1}H NMR (300 MHz, $CDCl_3$): δ 8.04 – 7.95 (m, 1H), 7.78 (dd, *J* = 7.8, 1.6 Hz, 1H), 7.45 – 7.37 (m, 1H), 7.16 (td, *J* = 7.8, 1.6 Hz, 1H), 4.60 (dt, *J* = 10.7, 5.3 Hz, 1H), 4.50 (ddd, *J* = 11.4, 8.8, 4.8 Hz, 1H), 4.38 – 4.26 (m, 1H), 3.85 (dd, *J* = 11.4, 4.8 Hz, 1H), 3.77 – 3.68 (m, 1H), 2.55 (dddd, *J* = 14.7, 8.8, 5.7, 3.3 Hz, 1H), 2.12 (ddt, *J* = 14.7, 9.7, 4.8 Hz, 1H).

^{13}C NMR (75 MHz, $CDCl_3$): δ 166.4, 141.4, 135.2, 132.9, 131.1, 128.1, 94.1, 62.2, 57.5, 48.3, 34.3.

HRMS (ESI): m/z calculated for $[C_{11}H_{12}O_2{}^{35}Cl_2{}^{127}I^+]$ $[M+H^+]$: 372.9259, measured: 372.9251.

The analytical data are in accordance with reported literature[13].

5,6-Dichlorohexyl but-2-ynoate (15):

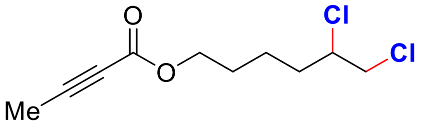

Following **GP3** with **S15**, 5 mL reaction mixture (0.5 mmol) was collected after flow electrolysis. The volatile solvent was removed *in vacuo* and the residue was purified by column chromatography yielding the product as a colorless oil (82 mg, 69%).

R*f* (hexane:CH_2Cl_2 1:1) = 0.4.

^{1}H NMR (300 MHz, $CDCl_3$): δ 4.16 (t, *J* = 6.3 Hz, 2H), 4.09 – 3.95 (m, 1H), 3.75 (dt, *J* = 13.1, 6.5 Hz, 1H), 3.67 – 3.58 (m, 1H), 2.09 – 2.00 (m, 1H), 1.98 (s, 3H), 1.80 – 1.61 (m, 4H), 1.56 – 1.45 (m, 1H).

^{13}C NMR (75 MHz, $CDCl_3$): δ 153.9, 85.8, 72.5, 65.4, 60.8, 48.2, 34.7, 27.9, 22.5, 3.9.

HRMS (ESI): m/z calculated for $[C_{10}H_{15}O_2{}^{35}Cl_2{}^+]$ $[M+H^+]$: 237.0444, measured: 237.0438.

((4,5-Dichloropentyl)sulfonyl)benzene (16):

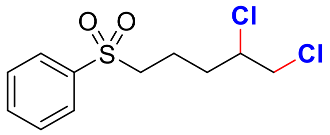

Following **GP3** with **S16**, 5 mL reaction mixture (0.5 mmol) was collected after flow electrolysis. The volatile solvent was removed *in vacuo* and the residue was purified by column chromatography yielding the product as a colorless oil (57 mg, 81%).

R*f* (hexane:EtOAc 4:1) = 0.35.

^{1}H NMR (300 MHz, $CDCl_3$): δ 7.94 – 7.87 (m, 2H), 7.70 – 7.62 (m, 1H), 7.62 – 7.51 (m, 2H), 4.02 – 3.92 (m, 1H), 3.73 (dd, *J* = 11.4, 4.9 Hz, 1H), 3.63 – 3.54 (m, 1H), 3.12 (t, *J* = 7.4 Hz, 2H), 2.16 – 1.96 (m, 2H), 1.91 – 1.71 (m, 2H).

^{13}C NMR (75 MHz, $CDCl_3$): δ 138.9, 134.0, 129.5, 128.1, 60.0, 55.5, 47.8, 33.5, 19.6.

HRMS (ESI): m/z calculated for [$C_{11}H_{15}O_2S^{35}Cl_2^+$] [M+H$^+$]: 281.0170, measured: 281.0167.

5,6-Dichlorohexyl diphenyl phosphate (17):

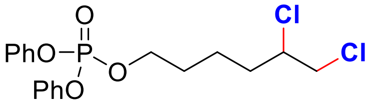

Following **GP3** with **S17**, 5 mL reaction mixture (0.5 mmol) was collected after flow electrolysis. The volatile solvent was removed *in vacuo* and the residue was purified by column chromatography yielding the product as a colorless oil (151 mg, 75%).

R*f* (CH_2Cl_2) = 0.4.

^{1}H NMR (300 MHz, $CDCl_3$): δ 7.41 – 7.29 (m, 4H), 7.26 – 7.13 (m, 6H), 4.33 – 4.21 (m, 2H), 3.97 (tdd, *J* = 8.7, 5.0, 3.6 Hz, 1H), 3.73 (dd, *J* = 11.3, 5.0 Hz, 1H), 3.60 (dd, *J* = 11.3, 7.6 Hz, 1H), 2.06 – 1.91 (m, 1H), 1.81 – 1.58 (m, 4H), 1.55 – 1.40 (m, 1H).

^{13}C NMR (75 MHz, $CDCl_3$): δ 150.7 (d, *J* = 7.2 Hz), 129.9 (d, *J* = 0.4 Hz), 125.5 (d, *J* = 1.2 Hz), 120.2 (d, *J* = 4.9 Hz), 69.0 (d, *J* = 6.4 Hz), 60.8, 48.1, 34.5, 29.7 (d, *J* = 6.8 Hz), 22.0.

HRMS (ESI): m/z calculated for [$C_{18}H_{22}O_4P^{35}Cl_2^+$] [M+H$^+$]: 403.0633, measured: 403.0638.

3,4-Dichlorobutyl pyrazine-2-carboxylate (18):

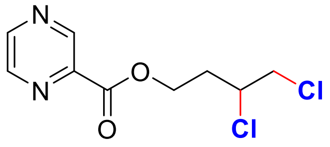

Following **GP3** with **S18**, 5 mL reaction mixture (0.5 mmol) was collected after flow electrolysis. The volatile solvent was removed *in vacuo* and the residue was purified by column chromatography yielding the product as a yellow oil (71 mg, 57%).

R*f* (hexane:EtOAc 2:1) = 0.3.

^{1}H NMR (300 MHz, $CDCl_3$): δ 9.31 (d, *J* = 1.3 Hz, 1H), 8.86 – 8.64 (m, 2H), 4.76 – 4.54 (m, 2H), 4.33 – 4.18 (m, 1H), 3.85 (dd, *J* = 11.4, 4.9 Hz, 1H), 3.79 – 3.69 (m, 1H), 2.58 (dddd, *J* = 14.6, 8.1, 6.3, 3.3 Hz, 1H), 2.20 (qd, *J* = 10.1, 5.2 Hz, 1H).

^{13}C NMR (75 MHz, $CDCl_3$): δ 163.9, 148.0, 146.4, 144.6, 143.3, 62.8, 57.3, 48.2, 34.2.

HRMS (ESI): m/z calculated for [$C_9H_{11}N_2O_2{}^{35}Cl_2{}^+$] [M+H$^+$]: 249.0198, measured: 249.0192.

2-((2,3-Dichloropropoxy)methyl)pyridine (19):

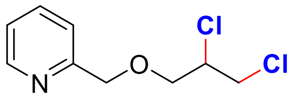

Following **GP3** with **S19**, 5 mL reaction mixture (0.5 mmol) was collected after flow electrolysis. The volatile solvent was removed *in vacuo* and the residue was purified by column chromatography yielding the product as a yellow oil (40 mg, 36%).

R*f* (hexane:EtOAc 1:1) = 0.7.

^{1}H NMR (300 MHz, $CDCl_3$): δ 8.56 (d, *J* = 4.6 Hz, 1H), 7.72 (td, *J* = 7.7, 1.7 Hz, 1H), 7.46 (d, *J* = 7.8 Hz, 1H), 7.21 (dd, *J* = 7.0, 5.4 Hz, 1H), 4.72 (s, 2H), 4.24 (td, *J* = 10.6, 5.3 Hz, 1H), 3.96 – 3.79 (m, 4H).

^{13}C NMR (126 MHz, $CDCl_3$): δ 157.8, 149.2, 137.0, 122.8, 121.6, 74.4, 58.3, 45.3.

HRMS (ESI): m/z calculated for [$C_9H_{12}NO^{35}Cl_2{}^+$] [M+H$^+$]: 220.0296, measured: 220.0291.

3,4-Dichlorobutyl furan-2-carboxylate (20):

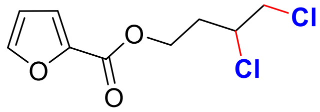

Following **GP3** with **S20**, 5 mL reaction mixture (0.5 mmol) was collected after flow electrolysis. The volatile solvent was removed *in vacuo* and the residue was purified by column chromatography yielding the product as a colorless oil (78 mg, 66%).

R*f* (hexane:CH_2Cl_2 2:1) = 0.3.

^{1}H NMR (300 MHz, $CDCl_3$): δ 7.59 (dd, *J* = 1.7, 0.8 Hz, 1H), 7.19 (dt, *J* = 5.7, 2.8 Hz, 1H), 6.52 (dd, *J* = 3.5, 1.7 Hz, 1H), 4.61 – 4.40 (m, 2H), 4.31 – 4.17 (m, 1H), 3.90 – 3.79 (m, 1H), 3.79 – 3.68 (m, 1H), 2.58 – 2.41 (m, 1H), 2.22 – 2.03 (m, 1H).

^{13}C NMR (75 MHz, $CDCl_3$): δ 158.5, 146.7, 144.4, 118.4, 112.1, 61.4, 57.5, 48.3, 34.3.

HRMS (ESI): m/z calculated for [$C_9H_{11}O_3{}^{35}Cl_2{}^+$] [M+H$^+$]: 237.0080, measured: 237.0073.

(1*S*,2*R*,4*S*)-2-(2,3-Dichloropropoxy)-1,3,3-trimethylbicyclo[2.2.1]heptane (21):

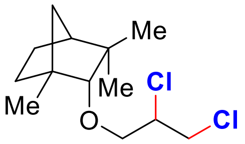

Following **GP3** with **S21**, 5 mL reaction mixture (0.5 mmol) was collected after flow electrolysis. The volatile solvent was removed *in vacuo* and the residue was purified by column chromatography yielding the product as a colorless oil in inseparable 1:1 diastereomeric mixture (87 mg, 66%).

$\mathbf{R}_f$ (hexane:CH_2Cl_2 4:1) = 0.55.

^{1}H NMR (300 MHz, $CDCl_3$): δ 4.17 – 4.06 (m, 1H), 3.96 – 3.87 (m, 1H), 3.86 – 3.80 (m, 1H), 3.77 – 3.59 (m, 2H), 2.93 (s, 1H), 1.72 – 1.62 (m, 3H), 1.46 – 1.33 (m, 2H), 1.11 (s, 3H), 1.09 – 1.05 (m, 1H), 1.03 (s, 3H), 1.00 – 0.93 (m, 1H), 0.90 (s, 3H). The ^{1}H NMR data is provided for both isomers together since the splitting patterns do not differ enough so that they can be reported separately.

^{13}C NMR (75 MHz, $CDCl_3$): δ 94.3, 72.1, 58.8, 49.5, 48.7, 45.6, 41.5, 39.9, 31.7, 26.2, 26.0, 20.8, 20.2 (for one isomer).

94.3, 71.9, 58.8, 49.4, 48.7, 45.6, 41.5, 39.8, 31.7, 26.2, 26.0, 20.7, 20.2 (for another isomer).

HRMS (ESI): m/z calculated for [$C_{13}H_{23}O^{35}Cl_2^+$] [$M+H^+$]: 265.1120, measured: 265.1117.

Methyl-*anti*-2,3-dichloro-3-phenylpropanoate (22):

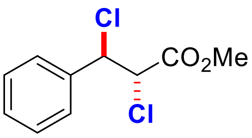

Following **GP3** with methyl cinnamate, 5 mL reaction mixture (0.5 mmol) was collected after flow electrolysis. The volatile solvent was removed *in vacuo* and the residue was purified by column chromatography yielding the product as a white solid (104 mg, 89%).

$\mathbf{R}_f$ (hexane:CH_2Cl_2 17:3) = 0.3.

^{1}H NMR (300 MHz, $CDCl_3$): δ 7.47 – 7.35 (m, 5H), 5.18 (d, J = 10.7 Hz, 1H), 4.62 (d, J = 10.7 Hz, 1H), 3.90 (s, 3H).

^{13}C NMR (75 MHz, $CDCl_3$): δ 168.1, 136.5, 129.6, 129.0, 128.2, 61.2, 58.9, 53.6.

HRMS (ESI): m/z calculated for [$C_{10}H_{10}O_2{}^{35}Cl^+$] [$M-Cl^+$]: 197.0364, measured: 197.0362.

The data is identical with reported literature[14].

(*anti*-2,3-Dichloro-1,3-diphenylpropan-1-one (23):

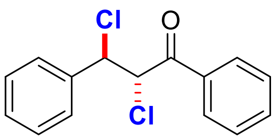

Following **GP3** with (*E*)-1,3-diphenylprop-2-en-1-one, 5 mL reaction mixture (0.5 mmol) was collected after flow electrolysis. The volatile solvent was removed *in vacuo* and the residue was

purified by column chromatography yielding the product as a white solid (117 mg, 84%).

R$_f$ (hexane:CH_2Cl_2 3:1) = 0.5.

^{1}H NMR (300 MHz, $CDCl_3$): δ 8.10 (dd, *J* = 5.3, 3.4 Hz, 2H), 7.67 (ddd, *J* = 6.6, 3.8, 1.2 Hz, 1H), 7.60 – 7.50 (m, 4H), 7.49 – 7.39 (m, 3H), 5.61 – 5.41 (m, 2H).

^{13}C NMR (75 MHz, $CDCl_3$): δ 191.5, 137.2, 134.8, 134.4, 129.5, 129.1, 129.1, 128.9, 128.5, 60.2, 57.1.

HRMS (ESI): m/z calculated for [$C_{15}H_{12}O^{35}Cl^+$] [M-Cl^+]: 243.0571, measured: 243.0574.

The analytical data are in accordance with reported literature[13].

***anti*-2,3-Dichloro-3-(4-fluorophenyl)-1-phenylpropan-1-one (24):**

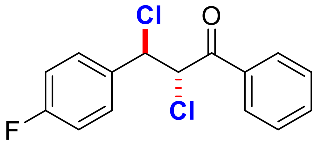

Following **GP3** with (*E*)-1,3-diphenylprop-2-en-1-one, 5 mL reaction mixture (0.5 mmol) was collected after flow electrolysis. The volatile solvent was removed *in vacuo* and the residue was purified by column chromatography yielding the product as a white solid (113 mg, 76%).

R$_f$ (hexane:CH_2Cl_2 17:3) = 0.35.

^{1}H NMR (300 MHz, $CDCl_3$): δ 8.15 – 8.04 (m, 2H), 7.73 – 7.63 (m, 1H), 7.62 – 7.46 (m, 4H), 7.19 – 7.07 (m, 2H), 5.53 – 5.41 (m, 2H).

^{13}C NMR (75 MHz, $CDCl_3$): δ 191.3, 163.2 (d, *J* = 249.0 Hz), 134.7, 134.5, 133.1 (d, *J* = 3.3 Hz), 130.3 (d, *J* = 8.5 Hz), 129.2, 129.1, 116.0 (d, *J* = 21.9 Hz), 59.4, 57.2.

HRMS (ESI): m/z calculated for [$C_{15}H_{11}OF^{35}Cl^+$] [M-Cl^+]: 261.0477, measured: 261.0481.

The analytical data are in accordance with reported literature[15].

1,2-Dichloro-1,2-diphenylethane (25):

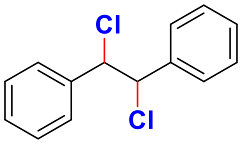

GP3 was followed with *trans*-stilbene using the solvent mixture HFIP/MeCN/DCM (4.2:3.1:2 v/v) to make the alkene soluble. 5 mL reaction mixture (0.5 mmol) was collected after flow electrolysis. The volatile solvent was removed *in vacuo* and the residue was purified by column chromatography yielding the product as a white solid as inseparable 1:1 diastereomeric mixture (85 mg, 68%).

R$_f$ (hexane:CH_2Cl_2 19:1) = 0.45.

1st diastereomer:

^{1}H NMR (300 MHz, $CDCl_3$): δ 7.48 – 7.37 (m, 10H), 5.24 (s, 2H).

^{13}C NMR (75 MHz, $CDCl_3$): δ 138.5, 129.1, 128.7, 128.2, 65.9.

2nd diastereomer:

^{1}H NMR (300 MHz, $CDCl_3$): δ 7.24 – 7.16 (m, 10H), 5.26 (s, 2H).

^{13}C NMR (75 MHz, $CDCl_3$): δ 137.4, 128.8, 128.3, 128.2, 67.8.

HRMS (ESI): m/z calculated for [$C_{14}H_{12}{}^{35}Cl^+$] [M-Cl^+]: 215.0622, measured: 215.0620.

The data is identical with reported literature[13].

4,5-Dichlorohexyl-4-methylbenzenesulfonate (26):

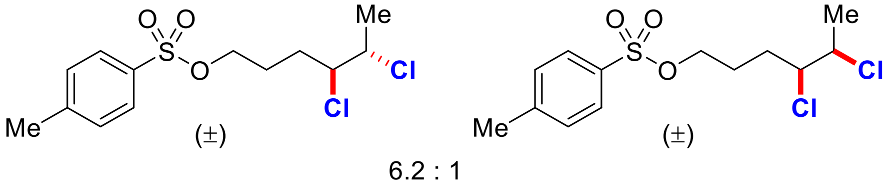

GP3 was followed with **S26** and 5 mL reaction mixture (0.5 mmol) was collected after flow electrolysis. The volatile solvent was removed *in vacuo* and the residue was purified by column chromatography yielding the product as a colorless oil as inseparable 6.2:1 diastereomeric mixture (128 mg, 79%).

R_f (hexane:CH_2Cl_2 1:1) = 0.4.

Major diastereomer:

^{1}H NMR (300 MHz, $CDCl_3$): δ 7.81 – 7.75 (m, 2H), 7.34 (d, J = 8.0 Hz, 2H), 4.15 – 3.96 (m, 3H), 3.92 – 3.71 (m, 1H), 2.44 (s, 3H), 2.08 – 1.90 (m, 2H), 1.79 – 1.67 (m, 2H), 1.57 (d, J = 6.6 Hz, 3H).

^{13}C NMR (75 MHz, $CDCl_3$): δ 145.1, 132.9, 130.0, 128.0, 69.7, 66.3, 60.1, 31.0, 25.8, 22.1, 21.7.

Minor diastereomer:

^{1}H NMR (300 MHz, $CDCl_3$): δ 7.81 – 7.75 (m, 2H), 7.34 (d, J = 8.0 Hz, 2H), 4.15 – 3.96 (m, 3H), 3.92 – 3.71 (m, 1H), 2.44 (s, 3H), 2.08 – 1.90 (m, 2H), 1.79 – 1.67 (m, 2H), 1.52 (d, J = 6.7 Hz, 3H).

HRMS (ESI): m/z calculated for [$C_{13}H_{19}O_3S^{35}Cl_2{}^+$] [M+$H^+$]: 325.0426, measured: 325.0419.

4,5-Dichlorohexyl-4-methylbenzenesulfonate (27):

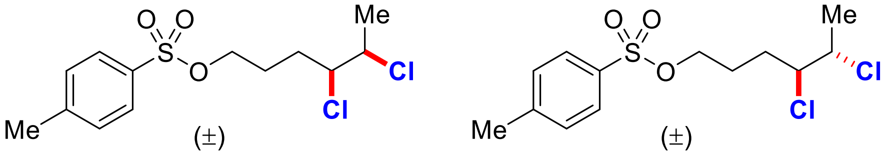

1.7 : 1

GP3 was followed with **S27** and 5 mL reaction mixture (0.5 mmol) was collected after flow electrolysis. The volatile solvent was removed *in vacuo* and the residue was purified by column chromatography yielding the product as a colorless oil as inseparable 1.7:1 diastereomeric mixture (140 mg, 86%).

R*f* (hexane:CH_2Cl_2 1:1) = 0.4.

Major diastereomer:

^{1}H NMR (300 MHz, $CDCl_3$): δ 7.78 (d, *J* = 8.3 Hz, 2H), 7.35 (d, *J* = 8.3 Hz, 2H), 4.18 (qd, *J* = 6.7, 3.0 Hz, 1H), 4.11 – 4.00 (m, 2H), 3.91 (dt, *J* = 7.8, 2.7 Hz, 1H), 2.44 (s, 3H), 2.01 – 1.90 (m, 2H), 1.79 – 1.68 (m, 2H), 1.53 (d, *J* = 6.7 Hz, 3H).

^{13}C NMR (75 MHz, $CDCl_3$): δ 145.1, 133.0, 130.0, 128.0, 69.7, 65.3, 59.9, 30.0, 26.4, 21.7, 20.8.

Minor diastereomer:

^{1}H NMR (300 MHz, $CDCl_3$): δ 7.78 (d, *J* = 8.3 Hz, 2H), 7.35 (d, *J* = 8.3 Hz, 2H), 4.08 – 4.02 (m, 3H), 3.83 (ddd, *J* = 9.2, 6.7, 2.5 Hz, 1H), 2.44 (s, 3H), 2.06 – 1.98 (m, 2H), 1.78 – 1.70 (m, 2H), 1.58 (d, *J* = 6.5 Hz, 3H).

^{13}C NMR (75 MHz, $CDCl_3$): δ 145.1, 133.0, 130.0, 128.0, 69.7, 66.3, 60.1, 31.0, 25.9, 22.1, 21.7.

HRMS (ESI): m/z calculated for [$C_{13}H_{18}O_3S^{35}Cl_2Na^+$] [M+Na$^+$]: 347.0251, measured: 347.0254.

4,5-Dichlorohexyl 4-chlorobenzoate (28):

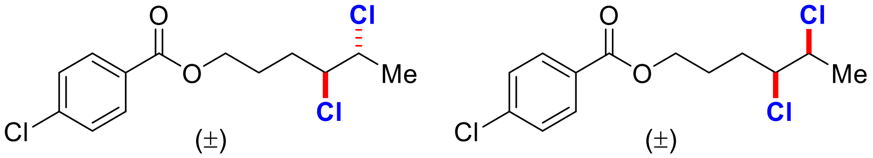

6 : 1

GP3 was followed with **S28** and 5 mL reaction mixture (0.5 mmol) was collected after flow electrolysis. The volatile solvent was removed *in vacuo* and the residue was purified by column chromatography yielding the product as a colorless liquid as inseparable 6:1 diastereomeric mixture (125 mg, 81%).

R*f* (hexane:CH_2Cl_2 2:1) = 0.45.

Major diastereomer:

^{1}H NMR (300 MHz, $CDCl_3$): δ 8.00 – 7.92 (m, 2H), 7.44 – 7.37 (m, 2H), 4.33 (dt, *J* = 6.2, 4.4 Hz, 2H), 4.18 – 4.04 (m, 1H), 4.03 – 3.83 (m, 1H), 2.22 – 2.03 (m, 2H), 1.98 – 1.84 (m, 2H), 1.64 (d, *J* = 6.5 Hz, 3H).

^{13}C NMR (75 MHz, $CDCl_3$): δ 165.8, 139.5, 131.1, 128.9, 128.7, 66.6, 64.4, 60.1, 31.8, 25.6, 22.4.

Minor diastereomer:

^{1}H NMR (300 MHz, $CDCl_3$): δ 8.00 – 7.92 (m, 2H), 7.44 – 7.37 (m, 2H), 4.33 (dt, *J* = 6.2, 4.4 Hz, 2H), 4.18 – 4.04 (m, 1H), 4.03 – 3.83 (m, 1H), 2.22 – 2.03 (m, 2H), 1.98 – 1.84 (m, 2H), 1.59 (d, *J* = 6.7 Hz, 3H).

^{13}C NMR peaks are not reported due to their low intensity of the minor isomer.

HRMS (ESI): m/z calculated for $[C_{13}H_{15}O_2{}^{35}Cl_2{}^{+}]$ [M-Cl^{+}]: 273.0449, measured: 273.0447.

4,5-Dichlorohexyl 4-chlorobenzoate (29):

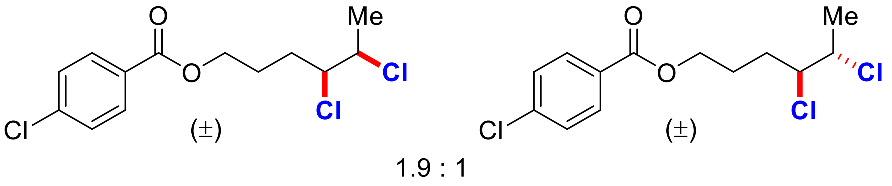

GP3 was followed with **S29** and 5 mL reaction mixture (0.5 mmol) was collected after flow electrolysis. The volatile solvent was removed *in vacuo* and the residue was purified by column chromatography yielding the product as a colorless liquid as inseparable 6:1 diastereomeric mixture (128 mg, 83%).

R_f (hexane:CH_2Cl_2 3:1) = 0.25.

Major diastereomer:

^{1}H NMR (300 MHz, $CDCl_3$): δ 8.00 – 7.92 (m, 2H), 7.45 – 7.37 (m, 2H), 4.40 – 4.32 (m, 2H), 4.31 – 4.21 (m, 1H), 4.10 – 4.04 (m, 1H), 2.21 – 2.04 (m, 2H), 1.96 – 1.80 (m, 2H), 1.58 (d, *J* = 6.4 Hz, 3H).

^{13}C NMR (75 MHz, $CDCl_3$): δ 165.8, 139.6, 131.1, 128.9, 128.7, 65.6, 64.4, 59.9, 30.5, 26.2, 20.7.

Minor diastereomer:

^{1}H NMR (300 MHz, $CDCl_3$): δ 8.00 – 7.92 (m, 2H), 7.45 – 7.37 (m, 2H), 4.40 – 4.32 (m, 2H), 4.10 – 4.04 (m, 1H), 3.97 (ddd, *J* = 11.7, 8.2, 3.5 Hz, 1H), 2.21 – 2.04 (m, 2H), 1.96 – 1.80 (m, 2H), 1.64 (d, *J* = 6.5 Hz, 3H).

^{13}C NMR (75 MHz, $CDCl_3$): δ 165.8, 139.6, 131.1, 128.9, 128.7, 66.6, 64.4, 60.1, 31.8, 25.6, 22.4.

HRMS (ESI): m/z calculated for [$C_{13}H_{16}O_2{}^{35}Cl^+$]: 239.0839, measured: 239.0836.

2,3-Dichloro-2,3-diphenylpropanenitrile (30):

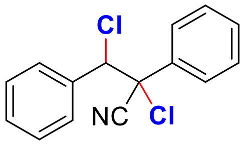

GP3 was followed with α-phenylcinnamonitrile and 5 mL reaction mixture (0.5 mmol) was collected after flow electrolysis. The volatile solvent was removed *in vacuo* and the residue was purified by column chromatography yielding the product as a white solid of inseparable 1:1 diastereomeric mixture (73 mg, 53%).

R*f* (hexane:CH_2Cl_2 9:1) = 0.3.

^{1}H NMR (300 MHz, $CDCl_3$): δ 7.60 – 7.54 (m, 2H), 7.43 (dd, *J* = 7.7, 1.8 Hz, 2H), 7.37 (d, *J* = 2.4 Hz, 2H), 7.34 – 7.24 (m, *J* = 10.5, 6.1, 3.0 Hz, 6H), 7.23 – 7.13 (m, 4H), 7.06 (dd, *J* = 8.5, 1.4 Hz, 2H), 7.01 (dd, *J* = 8.4, 1.4 Hz, 2H), 5.21 – 5.17 (m, *J* = 3.3 Hz, 2H).

^{13}C NMR (75 MHz, $CDCl_3$): δ 135.1, 134.5, 133.9, 133.8, 130.4 130.3, 130.1, 129.7, 129.6, 128.9, 128.6, 128.4, 128.2, 127.2, 127.1, 69.4, 68.0, 67.8, 66.4.

^{13}C NMR data for both the diastereomers are provided together due to the uncertainties in assignment for 1:1 mixture of products.

HRMS (ESI): m/z calculated for [$C_{15}H_{11}N^{35}Cl^+$] [M-Cl^+]: 240.0580, measured: 240.0577.

(1,2-Dichloropropane-1,2-diyl)dibenzene (31):

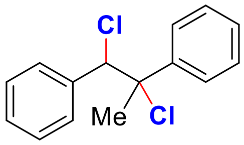

GP3 was followed with *trans*-α-methylstilbene using the solvent mixture HFIP/MeCN/DCM (5.2:3.1:1 v/v) to make the alkene soluble. 5 mL reaction mixture (0.5 mmol) was collected after flow electrolysis. The volatile solvent was removed *in vacuo* and the residue was purified by column chromatography yielding the product as a white solid of inseparable mixture of 1.4:1 diastereomeric mixture (61 mg, 46%).

R*f* (hexane:CH_2Cl_2 19:1) = 0.55.

^{1}H NMR (300 MHz, $CDCl_3$): δ 7.52 – 7.49 (m, 1H), 7.42 – 7.39 (m, 1H), 7.36 – 7.31 (m, 2H), 7.30 – 7.27 (m, 3H), 7.24 – 7.19 (m, 1H), 7.17 – 7.14 (m, 1H), 7.07 – 7.04 (m, 1H), 5.35 (s, 1H), 2.12 (s, 3H).

^{1}H NMR data for both the diastereomers are provided together due to the overlapping splitting of both diastereomers.

^{13}C NMR (75 MHz, $CDCl_3$): δ 141.2, 136.7, 129.5, 128.5, 128.3, 127.9, 127.7, 127.5, 75.9, 72.5, 27.8 (*major diastereomer*).

^{13}C NMR (75 MHz, CDCl$_3$): δ 142.2, 136.9, 129.9, 128.7, 128.3, 128.2, 128.1, 127.3, 74.6, 71.7, 26.9 (*minor diastereomer*).

HRMS (ESI): m/z calculated for [$C_{15}H_{14}{}^{35}Cl^+$] [M-Cl$^+$]: 229.0778, measured: 229.0779.

(2-Chloroethene-1,1,2-triyl)tribenzene (32):

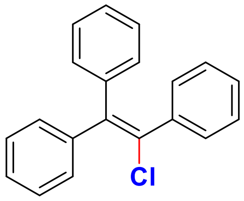

GP3 was followed with triphenylethylene using the solvent mixture HFIP/MeCN/DCM (5.2:3.1:1 v/v) to make the alkene soluble. 5 mL reaction mixture (0.5 mmol) was collected after flow electrolysis. The volatile solvent was removed *in vacuo* and the residue was purified by column chromatography yielding the product as a white solid (107 mg, 74%).

R*f* (hexane:CH$_2$Cl$_2$ 19:1) = 0.6.

^{1}H NMR (300 MHz, CDCl$_3$): δ 7.45 – 7.39 (m, 4H), 7.37 (ddt, *J* = 5.7, 2.6, 1.0 Hz, 3H), 7.25 – 7.19 (m, 3H), 7.16 – 7.11 (m, 3H), 7.04 – 6.99 (m, 2H).

^{13}C NMR (75 MHz, CDCl$_3$): δ 142.0, 141.2, 140.4, 139.5, 130.7, 130.1, 129.9, 128.3, 128.2, 128.1, 128.0, 127.7, 127.7, 127.2.

HRMS (ESI): m/z calculated for [$C_{20}H_{15}{}^{35}Cl^+$] [M$^+$]: 290.0857, measured: 290.0856.

The data is identical with reported literature[16].

5-(Chloromethyl)dihydrofuran-2(3*H*)-one (33):

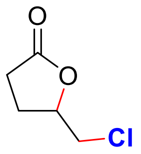

Following **GP3** with 4-pentenoic acid, 5 mL reaction mixture (0.5 mmol) was collected after flow electrolysis. The volatile solvent was removed *in vacuo* and the residue was purified by column chromatography yielding the product as a colorless oil (58 mg, 87%).

R*f* (hexane:CH$_2$Cl$_2$ 1:2) = 0.4.

^{1}H NMR (300 MHz, CDCl$_3$): δ 4.83 – 4.70 (m, 1H), 3.79 – 3.61 (m, 2H), 2.72 – 2.51 (m, 2H), 2.46 – 2.33 (m, 1H), 2.16 (dddd, *J* = 13.1, 10.2, 7.6, 6.3 Hz, 1H).

^{13}C NMR (75 MHz, CDCl$_3$): δ 176.4, 78.2, 46.1, 28.3, 25.1.

Analytical data matches with previous report[17].

5-(Chloromethyl)-3-phenyldihydrofuran-2(3*H*)-one (34):

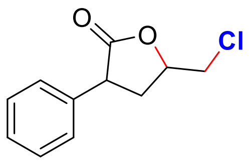

Following **GP3** with **S33**, 5 mL reaction mixture (0.5 mmol) was collected after flow electrolysis. The volatile solvent was removed *in vacuo* and the residue was purified by column chromatography yielding the product as a white gum (76 mg, 72%).

R_f (hexane:CH_2Cl_2 1:3) = 0.45.

^{1}H NMR (300 MHz, $CDCl_3$): δ 7.39 (ddd, *J* = 6.4, 3.6, 2.0 Hz, 2H), 7.35 – 7.28 (m, 3H), 4.75 (qd, *J* = 10.0, 5.0 Hz, 1H), 3.95 (dd, *J* = 12.3, 9.2 Hz, 1H), 3.80 (d, *J* = 4.9 Hz, 2H), 2.84 (ddd, *J* = 13.0, 9.2, 6.0 Hz, 1H), 2.35 (td, *J* = 12.6, 10.0 Hz, 1H).

^{13}C NMR (75 MHz, $CDCl_3$): δ 175.9, 136.2, 129.1, 128.2, 128.0, 76.2, 46.8, 45.3, 35.1.

HRMS (ESI): m/z calculated for [$C_{11}H_{12}O_2{}^{35}Cl^+$] [$M+H^+$]: 211.0526, measured: 211.0520.

2-Chloro-1,3-diphenylpropane-1,3-dione (35):

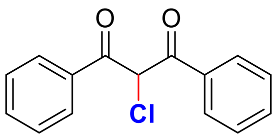

Following **GP3** with 1,3-diphenyl-1,3-propanedione, 5 mL reaction mixture (0.5 mmol) was collected after flow electrolysis. The volatile solvent was removed *in vacuo* and the residue was purified by column chromatography yielding the product as a colorless oil (93 mg, 72%).

R_f (hexane:CH_2Cl_2 2:1) = 0.3.

^{1}H NMR (300 MHz, $CDCl_3$): δ 8.06 – 7.95 (m, 4H), 7.66 – 7.55 (m, 2H), 7.52 – 7.41 (m, 4H), 6.44 (s, 1H).

^{13}C NMR (75 MHz, $CDCl_3$): δ 189.5, 134.5, 133.9, 129.4, 129.1, 62.9.

Analytical data matches with previous report[18].

2-Chloro-2-methyl-1,3-diphenylpropane-1,3-dione (36):

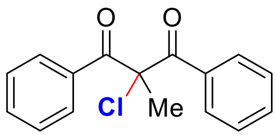

Following **GP3** with 2-methyl-1,3-diphenyl-1,3-propanedione, 5 mL reaction mixture (0.5 mmol) was collected after flow electrolysis. The volatile solvent was removed *in vacuo* and the residue was purified by column chromatography yielding the product as a colorless oil (94 mg, 69%).

R_f (hexane:CH_2Cl_2 9:1) = 0.5.

^{1}H NMR (300 MHz, $CDCl_3$): δ 7.97 – 7.88 (m, 4H), 7.52 – 7.45 (m, 2H), 7.40 – 7.32 (m, 4H), 2.20 (s, 3H).

^{13}C NMR (75 MHz, $CDCl_3$): δ 192.6, 133.7, 133.5, 130.0, 128.7, 75.4, 27.2.

HRMS (ESI): m/z calculated for [$C_{16}H_{14}O_2{}^{35}Cl^+$] [$M+H^+$]: 273.0677, measured: 273.0673.

Analytical data matches with previous report[19].

Ethyl 2-chloro-3-oxo-3-phenylpropanoate (37):

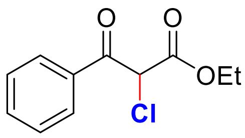

Following **GP3** with ethyl benzoylacetate, 5 mL reaction mixture (0.5 mmol) was collected after flow electrolysis. The volatile solvent was removed *in vacuo* and the residue was purified by column chromatography yielding the product as a colorless liquid (106 mg, 94%).

R*f* (hexane:CH_2Cl_2 1:1) = 0.6.

^{1}H NMR (300 MHz, $CDCl_3$): δ 8.04 – 7.94 (m, 2H), 7.63 (dd, *J* = 10.6, 4.2 Hz, 1H), 7.50 (t, *J* = 7.6 Hz, 2H), 5.62 (s, 1H), 4.28 (q, *J* = 7.1 Hz, 2H), 1.23 (t, *J* = 7.1 Hz, 3H).

^{13}C NMR (75 MHz, $CDCl_3$): δ 188.3, 165.4, 134.5, 133.4, 129.4, 129.0, 63.3, 58.1, 14.0.

HRMS (ESI): m/z calculated for [$C_{11}H_{11}O_3{}^{35}Cl^+$] [$M+H^+$]: 226.0391, measured: 226.0393.

Analytical data matches with previous report[18].

Ethyl 1-chloro-2-oxocyclohexane-1-carboxylate (38):

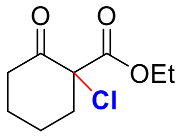

Following **GP3** with ethyl cyclohexanone-2-carboxylate, 5 mL reaction mixture (0.5 mmol) was collected after flow electrolysis. The volatile solvent was removed *in vacuo* and the residue was purified by column chromatography yielding the product as a colorless liquid (87 mg, 85%).

R*f* (hexane:CH_2Cl_2 1:1) = 0.65.

^{1}H NMR (300 MHz, $CDCl_3$): δ 4.27 (q, *J* = 7.1 Hz, 2H), 2.89 – 2.68 (m, 2H), 2.49 – 2.31 (m, 1H), 2.17 – 2.02 (m, 1H), 1.99 – 1.80 (m, 3H), 1.79 – 1.65 (m, 1H), 1.28 (t, *J* = 7.1 Hz, 3H).

^{13}C NMR (75 MHz, $CDCl_3$): δ 199.8, 167.3, 73.6, 63.0, 39.7, 38.9, 26.8, 22.2, 14.0.

HRMS (ESI): m/z calculated for [$C_9H_{14}O_3{}^{35}Cl^+$] [$M+H^+$]: 205.0631, measured: 205.0627.

Analytical data matches with previous report[20].

Ethyl 1-chloro-2-oxocyclopentane-1-carboxylate (39):

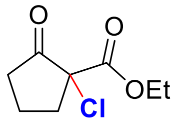

Following **GP3** with ethyl cyclopentanone-2-carboxylate, 5 mL reaction mixture (0.5 mmol) was collected after flow electrolysis. The volatile solvent was removed *in vacuo* and the residue was purified by column chromatography yielding the product as a colorless liquid (77 mg, 81%).

R*f* (hexane:CH_2Cl_2 2:1) = 0.3.

^{1}H NMR (300 MHz, CDCl$_3$): δ 4.26 (q, *J* = 7.1 Hz, 2H), 2.73 (ddd, *J* = 14.2, 9.5, 7.9 Hz, 1H), 2.62 – 2.48 (m, 1H), 2.46 – 2.30 (m, 2H), 2.20 – 2.05 (m, 2H), 1.28 (t, *J* = 7.1 Hz, 3H).

^{13}C NMR (75 MHz, CDCl$_3$): δ 206.3, 167.3, 69.8, 63.2, 38.5, 35.5, 19.2, 14.1.

HRMS (ESI): m/z calculated for [$C_8H_{12}O_3{}^{35}Cl$ $^+$] [M+H$^+$]: 191.0469, measured: 191.0463.

Analytical data matches with the previous report[21].

3. HPLC traces

3,4-Dichlorobutyl furan-2-carboxylate (20)

Following **GP3** with **S20**, 5 mL reaction mixture (0.5 mmol) was collected after flow electrolysis. The volatile solvent was removed *in vacuo* and the residue was purified by column chromatography yielding the product as a colorless oil (80 mg, 66%).

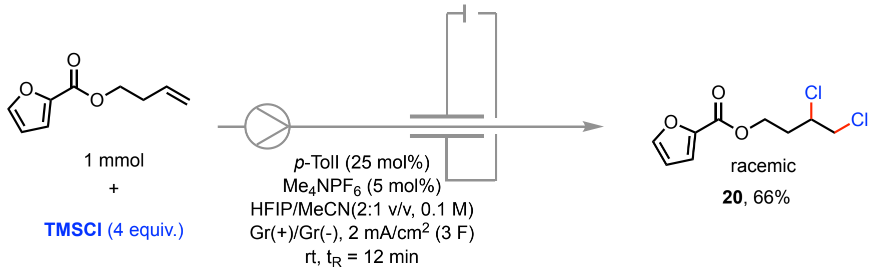

HPLC trace: racemate

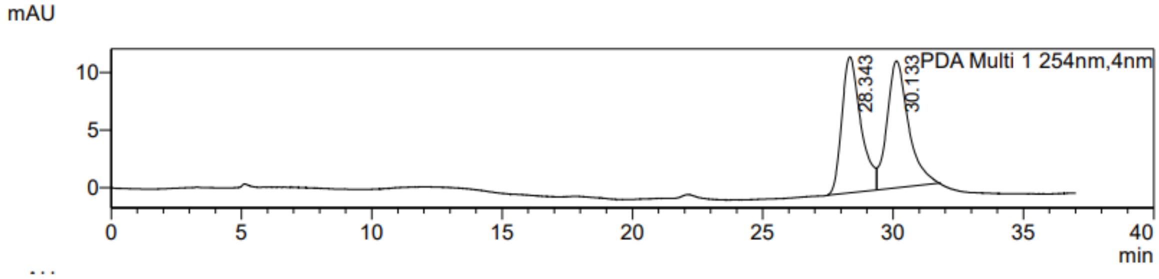

Peak Table
PDA Ch1 254nm

Peak#	Ret. Time	Area%
1	28.343	48.648
2	30.133	51.352
Total		100.000

(2*R*,2'*R*)-2,2'-((2-Iodo-1,3-phenylene)bis(oxy))bis(N,N-diisopropylpropanamide) (chiral iodoarene catalyst 1):

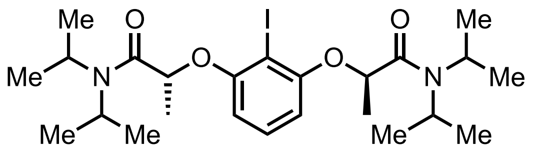

This compound was prepared following a previous publication from our group. Detailed experimental procedure and the characterisation data can be found in literature[22].

Dimesityl 2,2'-((2-iodo-1,3-phenylene)bis(oxy))(2*R*,2'*R*)-dipropionate (chiral iodoarene catalyst 2):

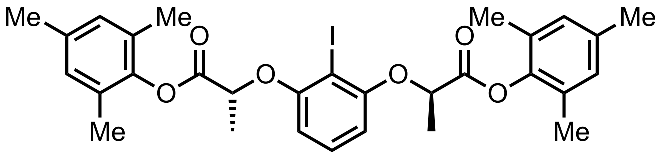

This compound was prepared previously. Detailed experimental procedure and the characterization data can be found in literature[22].

HPLC trace: product **20** after column chromatography following flow electrolysis with **chiral iodoarene catalyst 1**

The e.r. of the purified product was determined by HPLC analysis using a Daicel Chiralcel OD-H (0.46 cm * 25 cm) column and n-hexane : *i*-propanol (99.5 : 0.5, 1.0 mL/min) as the eluent. The detection was at 254 nm. tR = 30.50 min (major enantiomer), tR = 29.59 min (minor enantiomer), e.r.: 54.2:45.8.

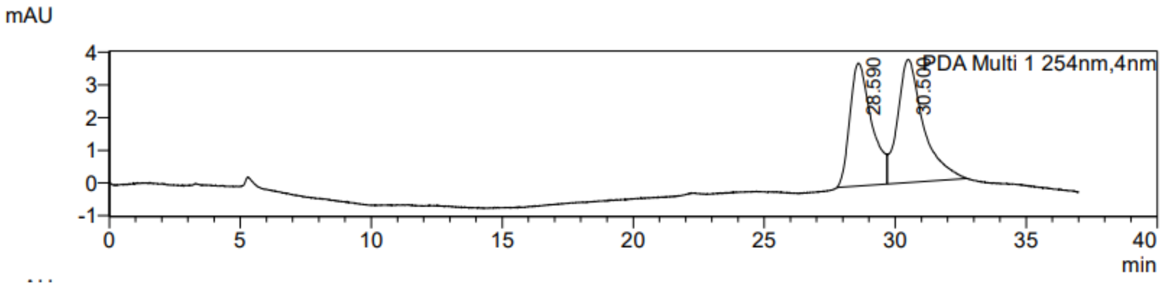

Peak Table

PDA Ch1 254nm

Peak#	Ret. Time	Area%
1	28.590	45.799
2	30.500	54.201
Total		100.000

HPLC trace: product **20** after column chromatography following flow electrolysis with **chiral iodoarene catalyst 2**

The e.r. of the purified product was determined by HPLC analysis using a Daicel Chiralcel OD-H (0.46 cm * 25 cm) column and n-hexane : *i*-propanol (99.5 : 0.5, 1.0 mL/min) as the eluent. The detection was at 254 nm. tR = 29.64 min (major enantiomer), tR = 28.01 min (minor enantiomer), e.r.: 53.7:46.3.

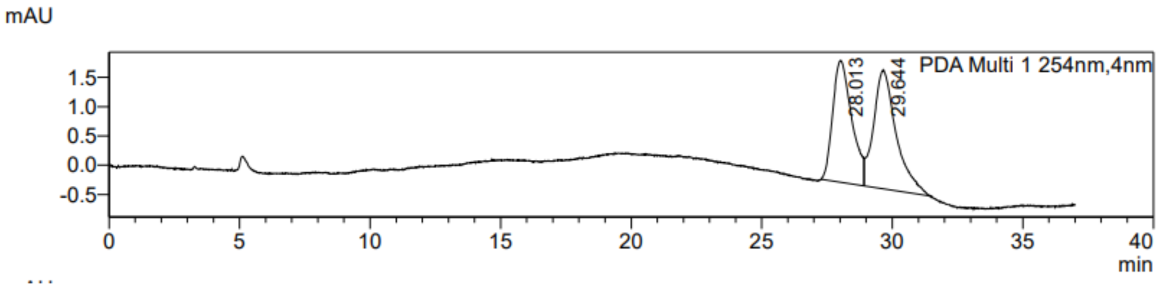

Peak Table

PDA Ch1 254nm

Peak#	Ret. Time	Area%
1	28.013	46.306
2	29.644	53.694
Total		100.000

4. NMR Spectra

Pent-4-en-1-yl benzoate (S7)

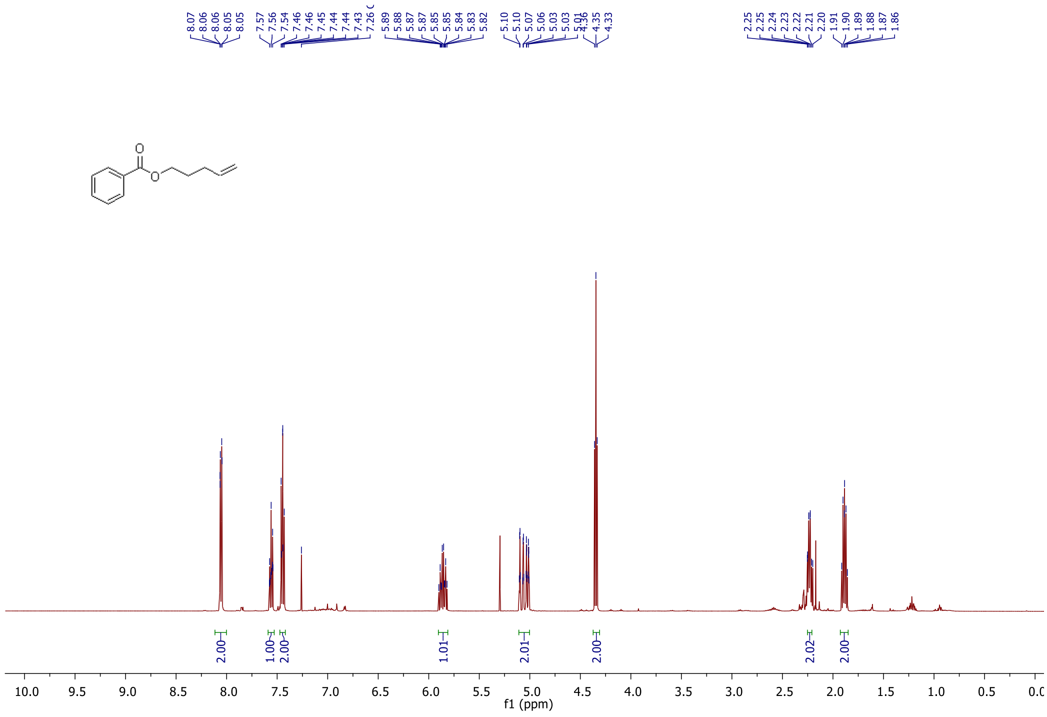

^{1}H NMR (500 MHz, $CDCl_3$)

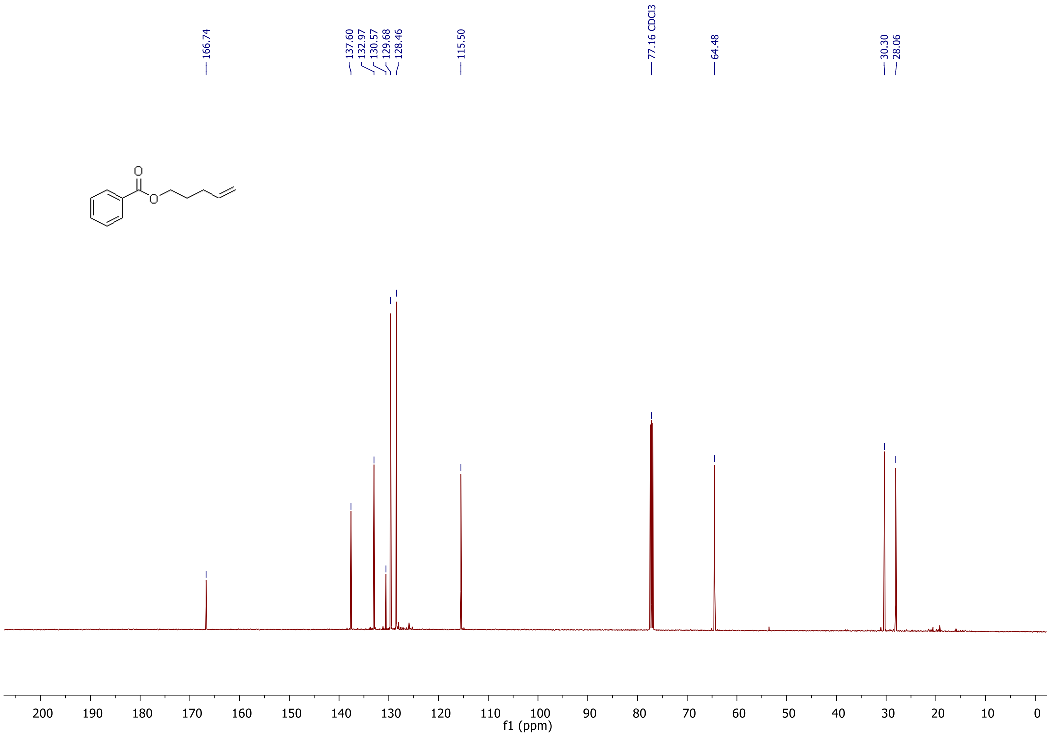

^{13}C NMR (126 MHz, $CDCl_3$)

But-3-en-1-yl benzoate (S8)

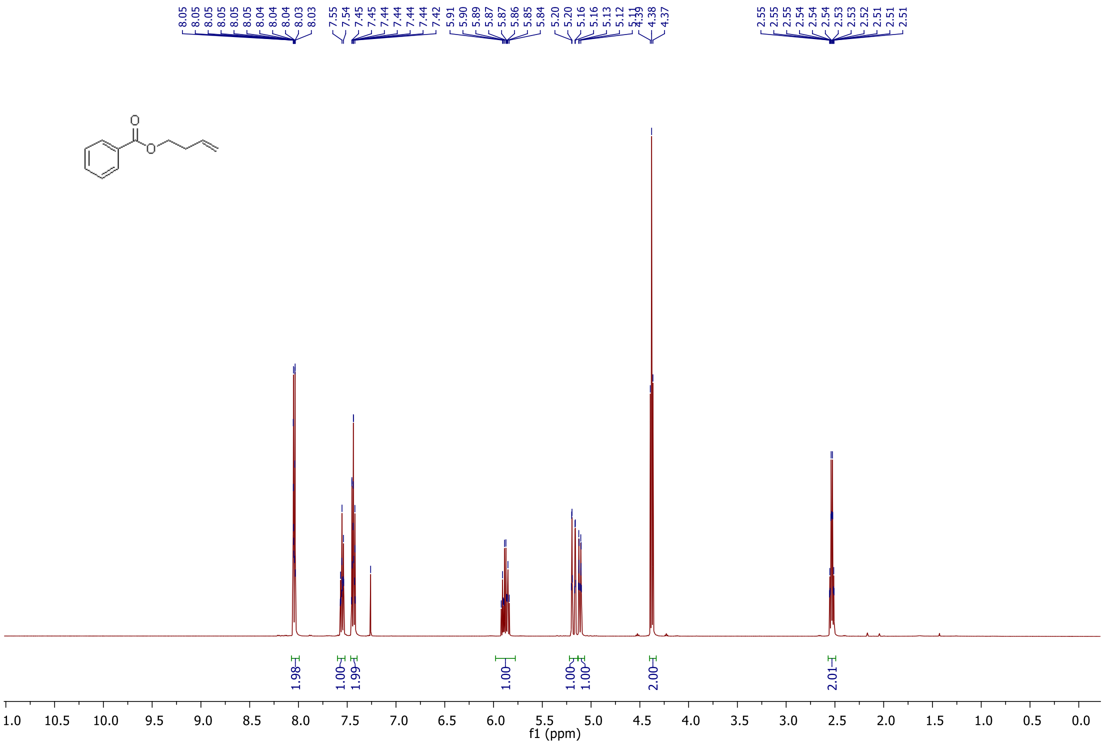

^{1}H NMR (500 MHz, $CDCl_3$)

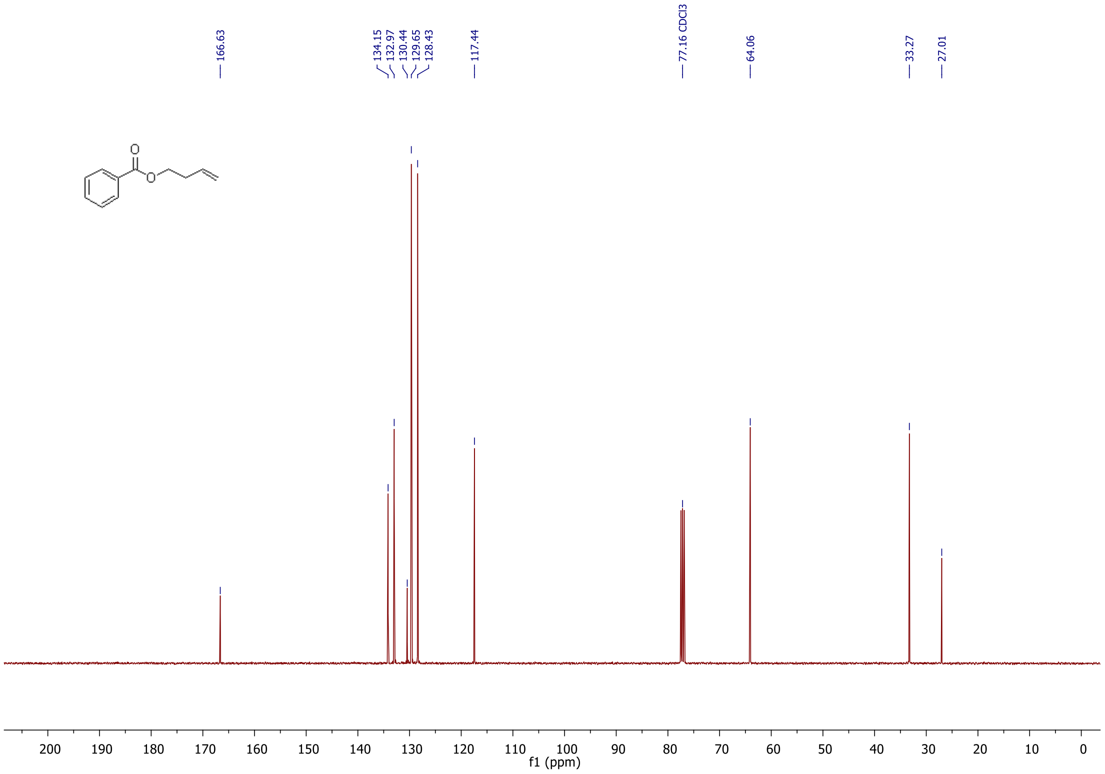

^{13}C NMR (101 MHz, $CDCl_3$)

2-Cyanoethyl pent-4-enoate (S9)

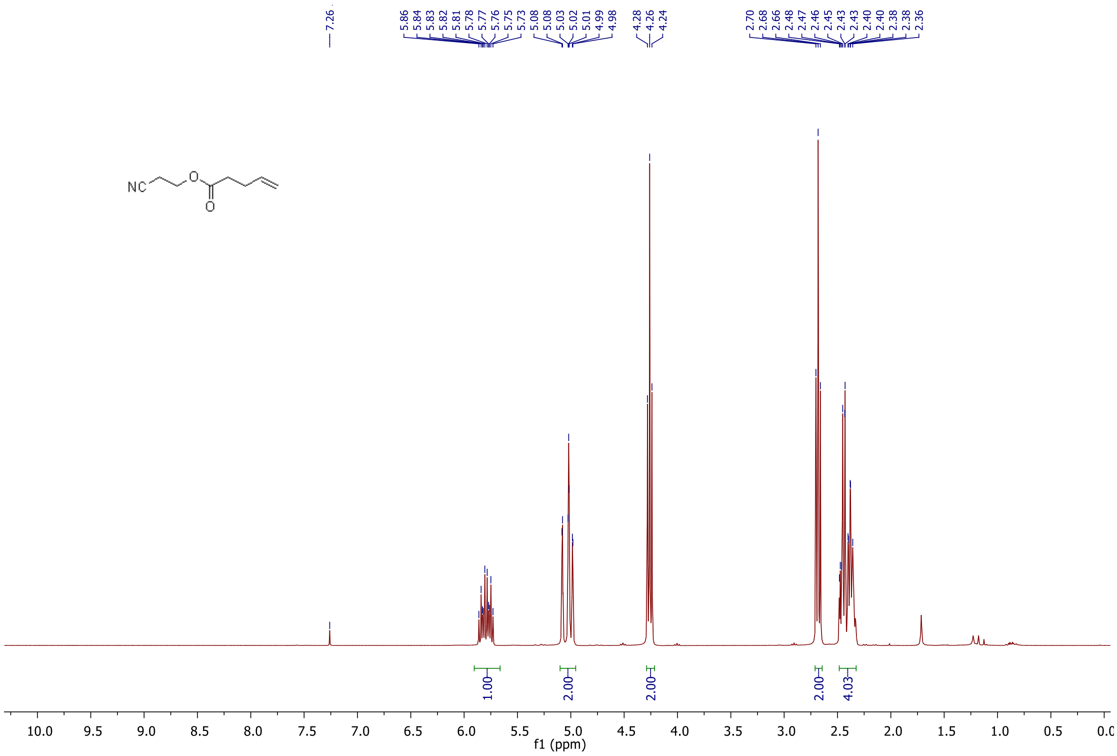

^{1}H NMR (300 MHz, $CDCl_3$)

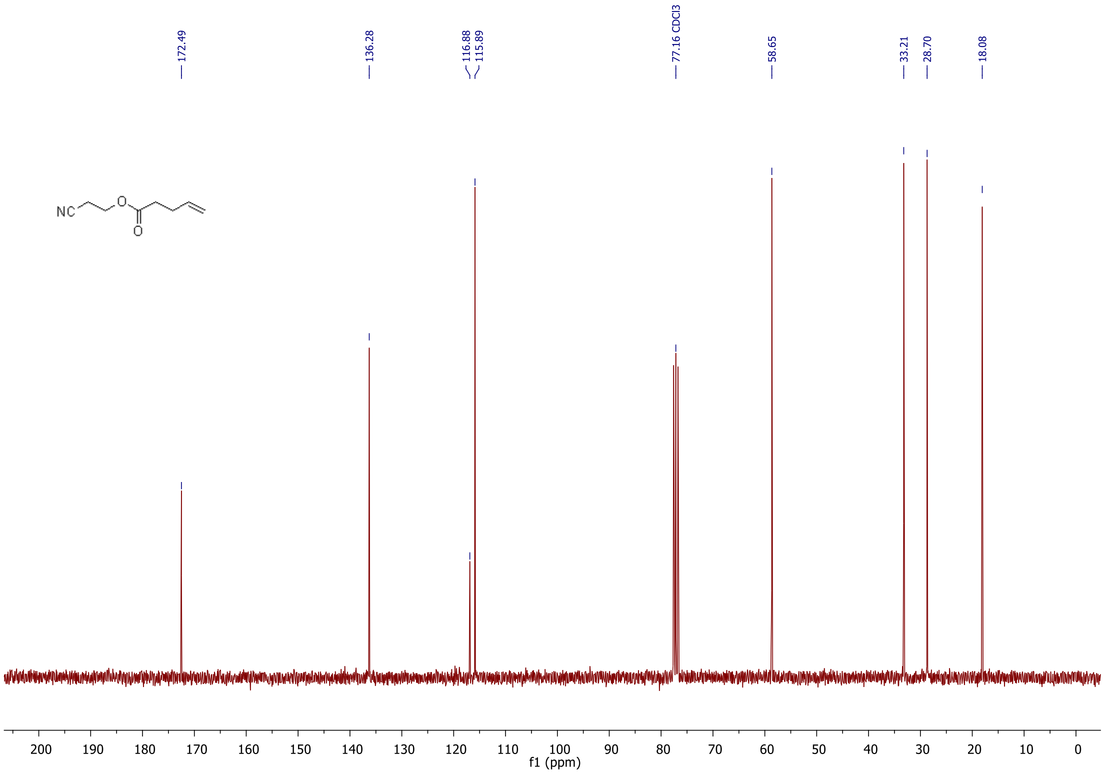

^{13}C NMR (75 MHz, $CDCl_3$)

Benzyl pent-4-enoate (S10)

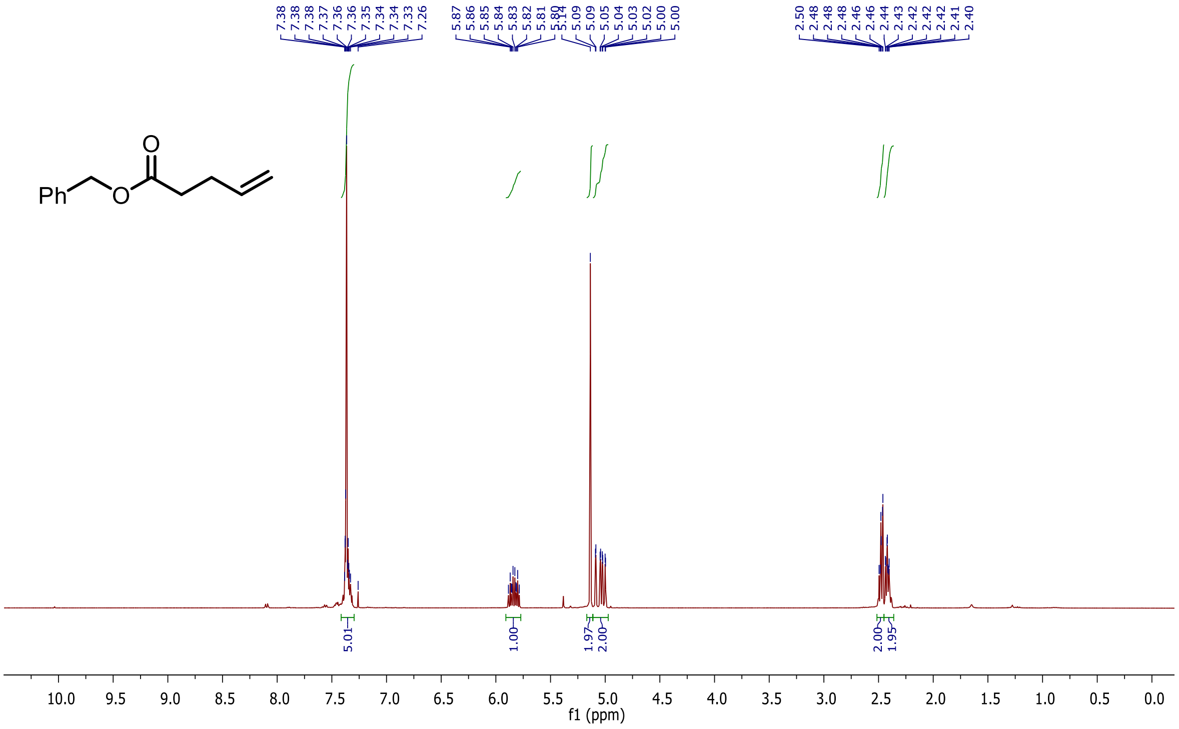

^{1}H NMR (400 MHz, $CDCl_3$)

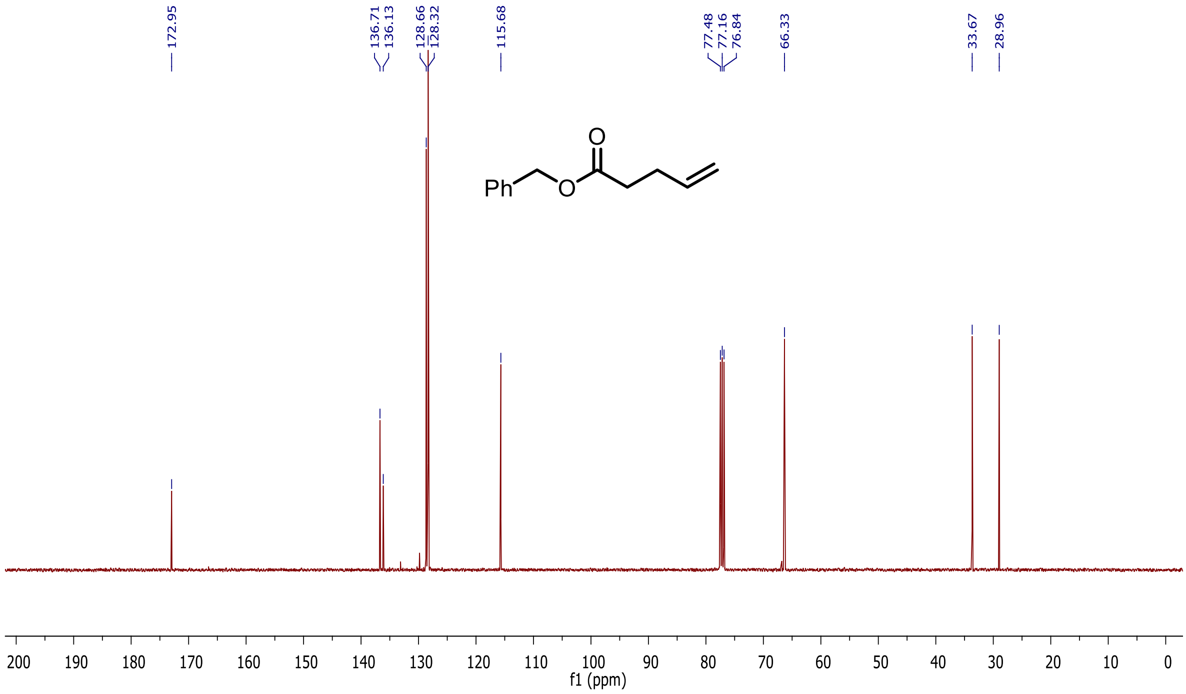

^{13}C NMR (101 MHz, $CDCl_3$)

But-3-en-1-yl 2-iodobenzoate (S14)

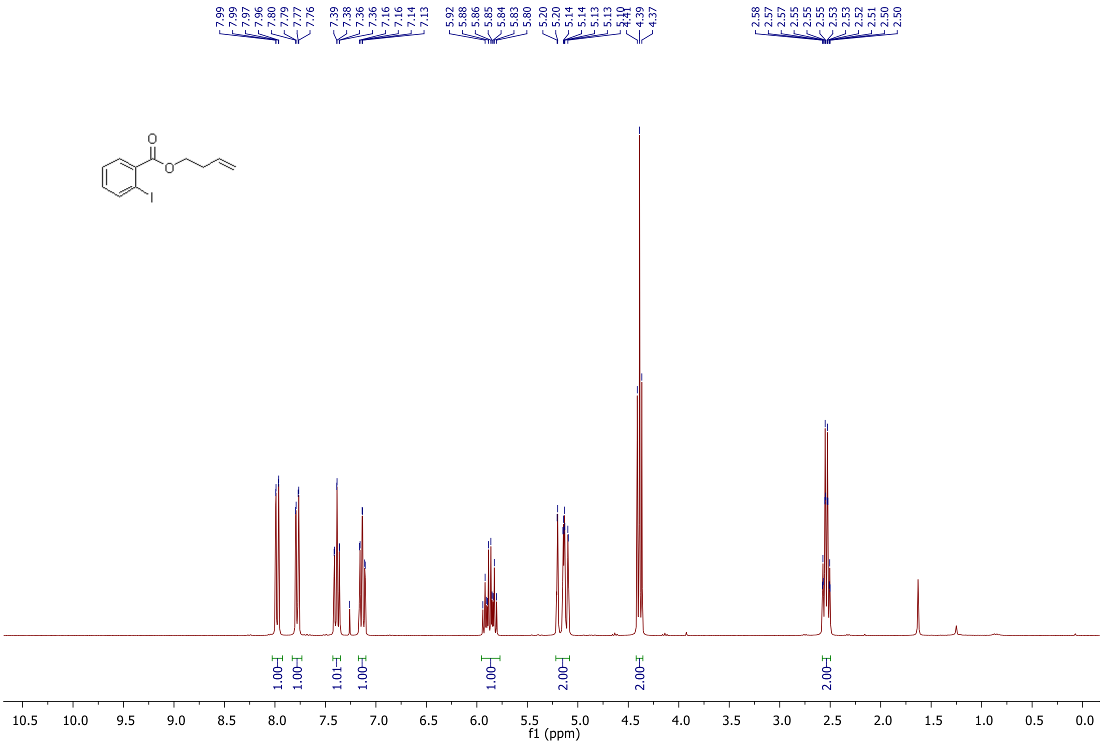

^{1}H NMR (300 MHz, $CDCl_3$)

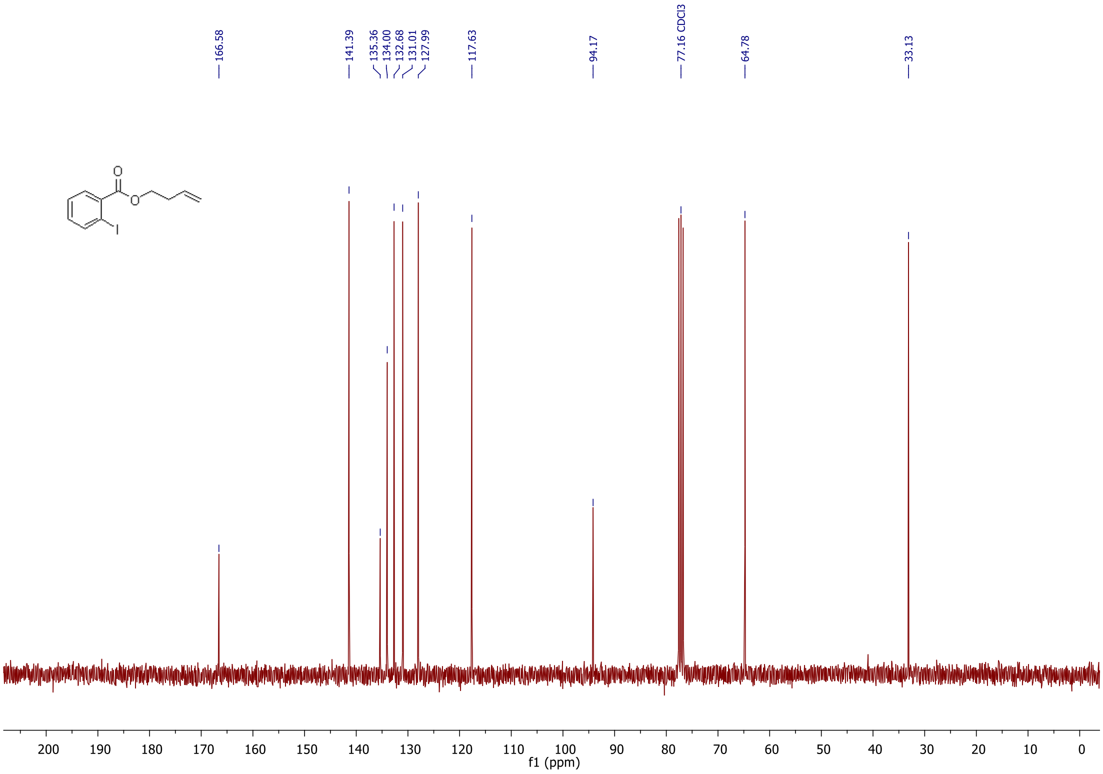

^{13}C NMR (75 MHz, $CDCl_3$)

Hex-5-en-1-yl but-2-ynoate (S15)

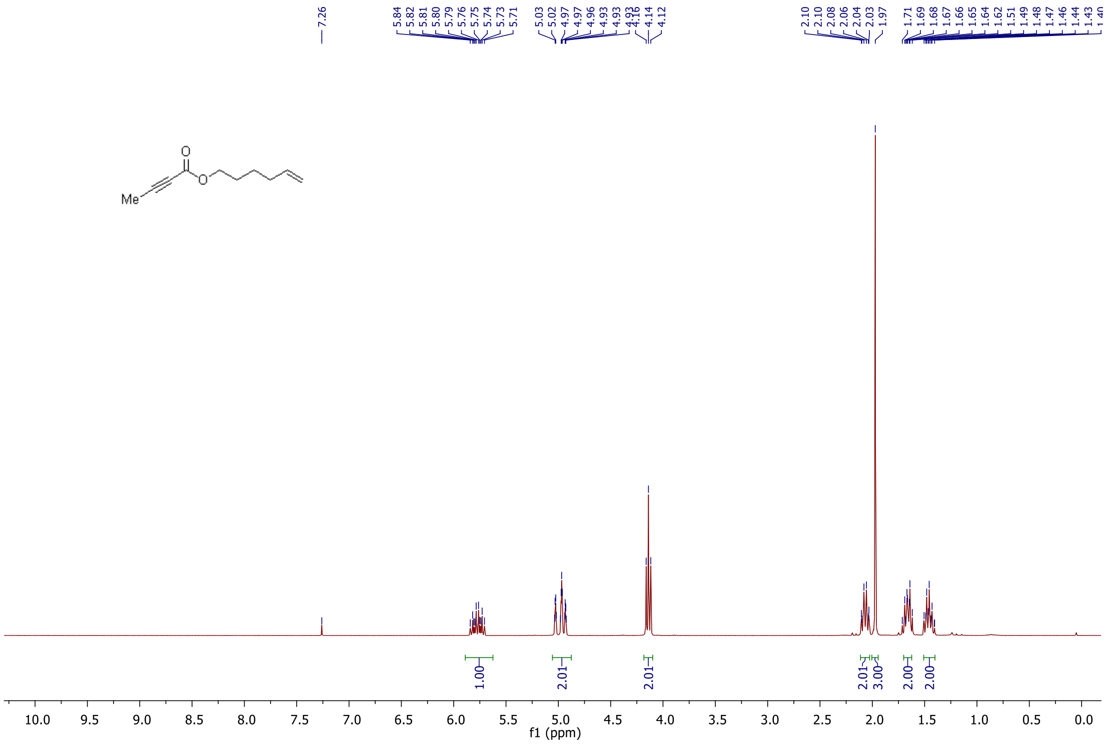

^{1}H NMR (300 MHz, $CDCl_3$)

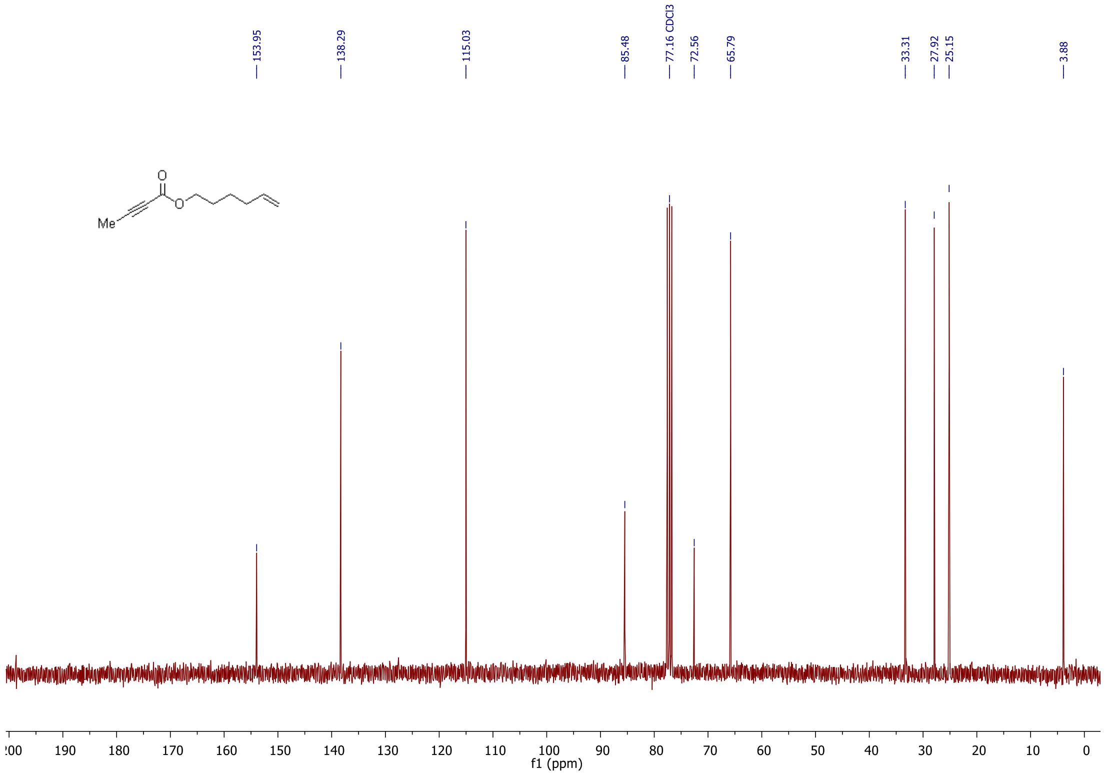

^{13}C NMR (75 MHz, $CDCl_3$)

(Pent-4-en-1-ylsulfonyl)benzene (S16)

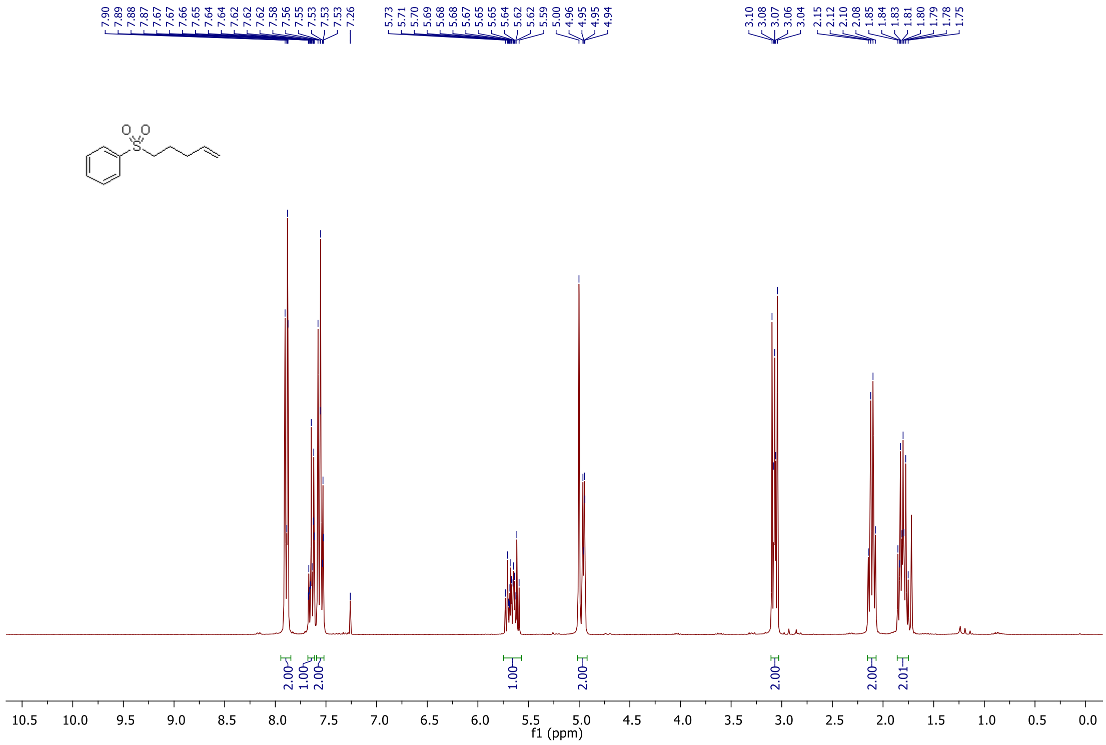

^{1}H NMR (300 MHz, $CDCl_3$)

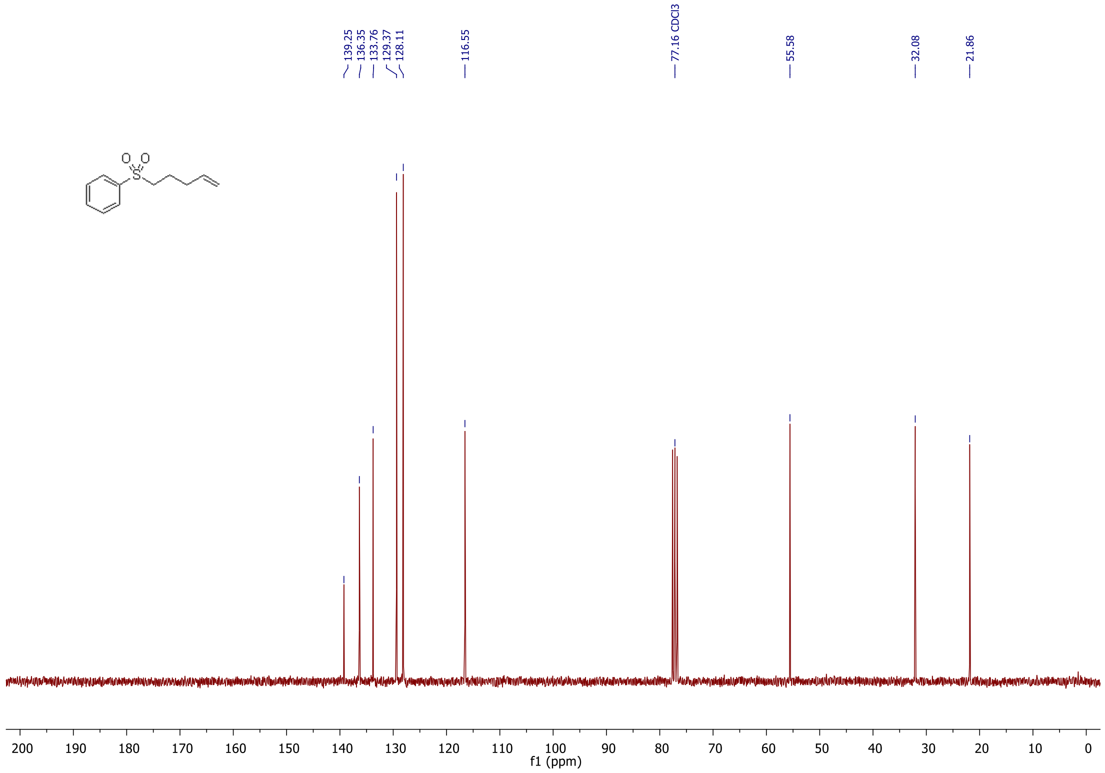

^{13}C NMR (75 MHz, $CDCl_3$)

Hex-5-en-1-yl diphenyl phosphate (S17)

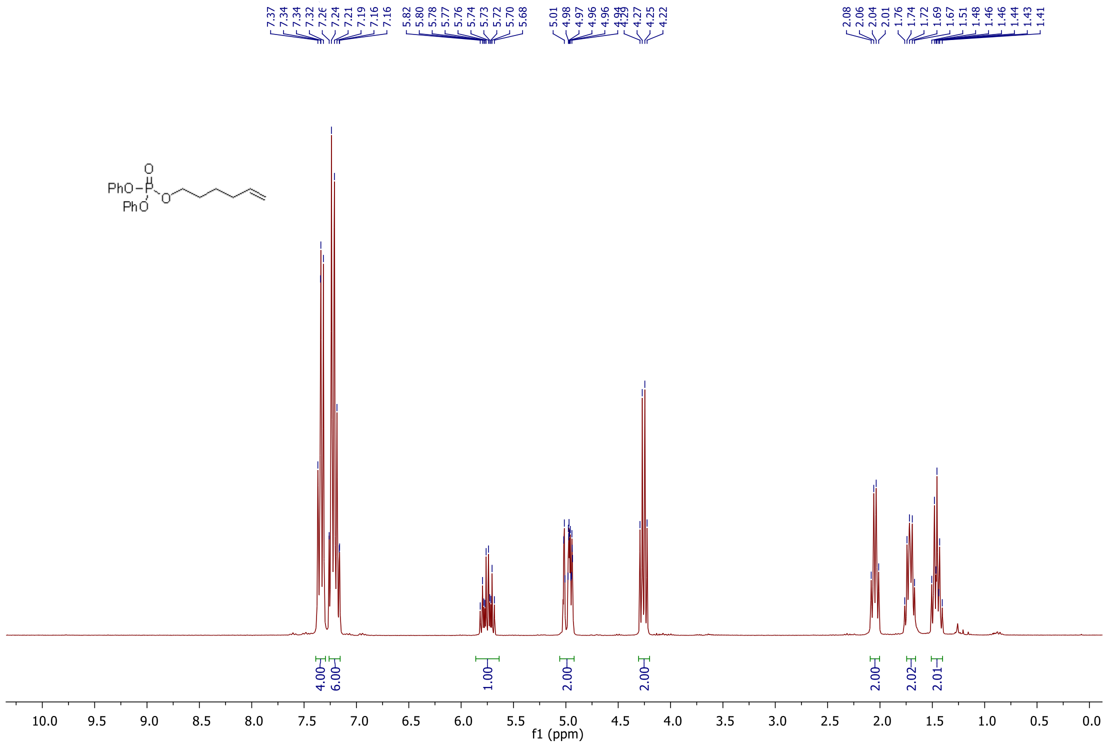

^1H NMR (300 MHz, $CDCl_3$)

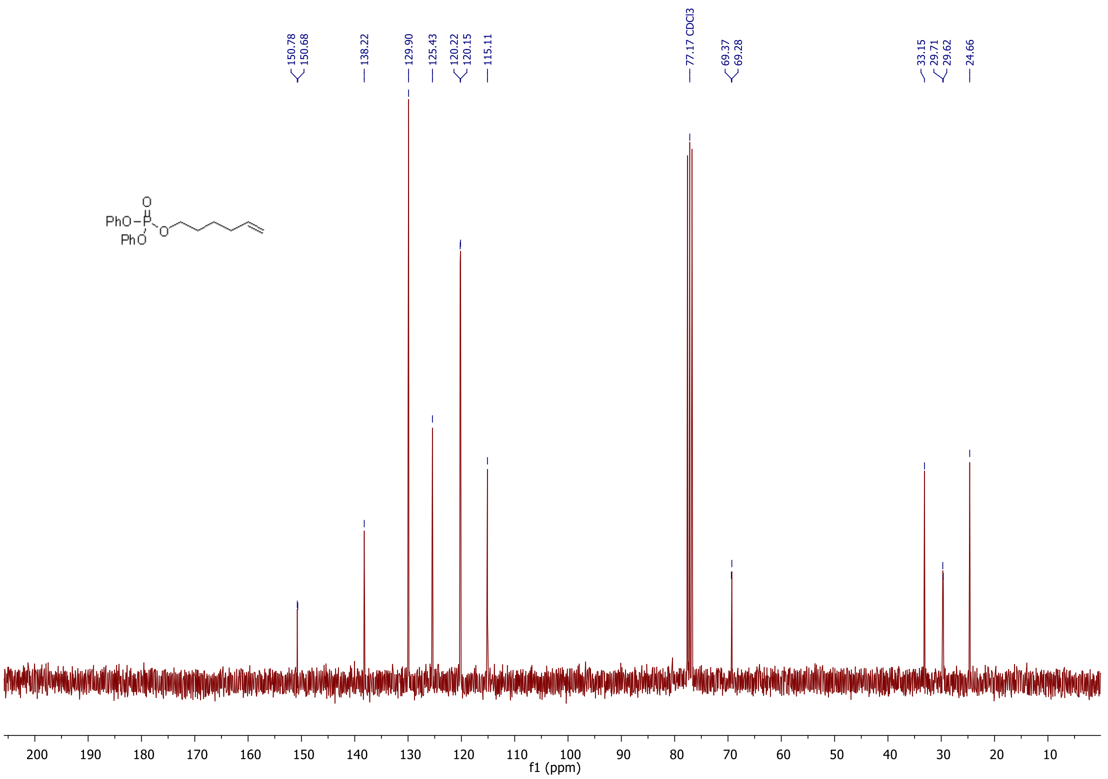

^{13}C NMR (75 MHz, $CDCl_3$)

But-3-en-1-yl pyrazine-2-carboxylate (S18)

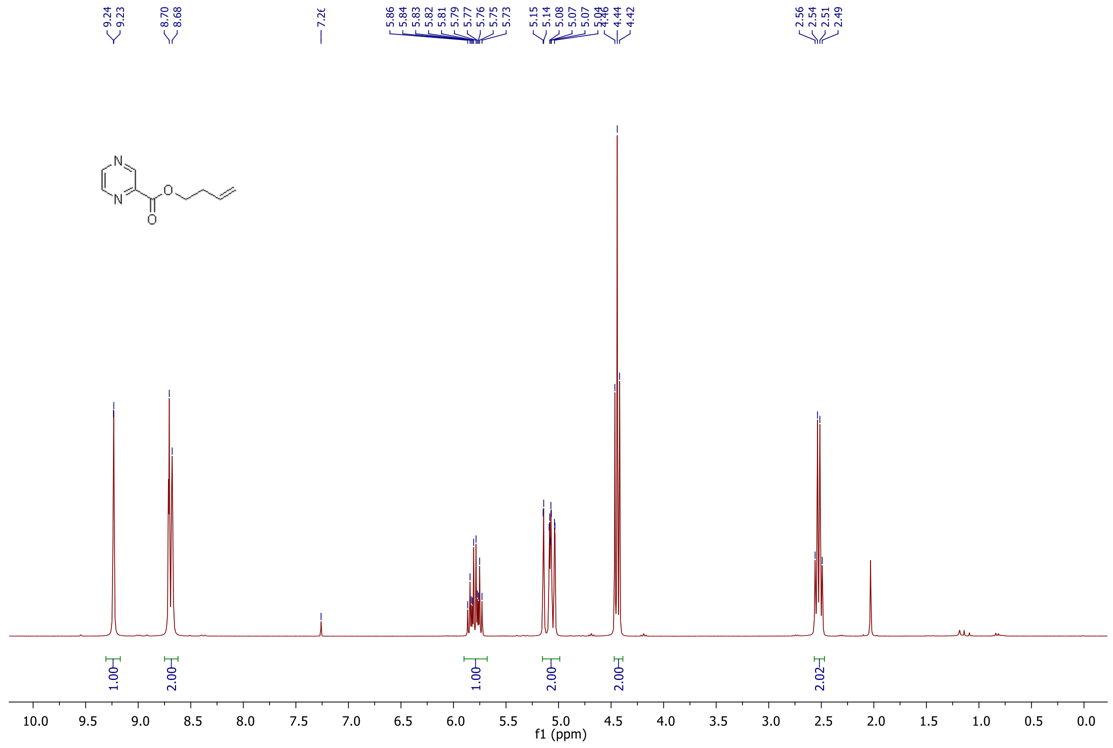

^{1}H NMR (300 MHz, $CDCl_3$)

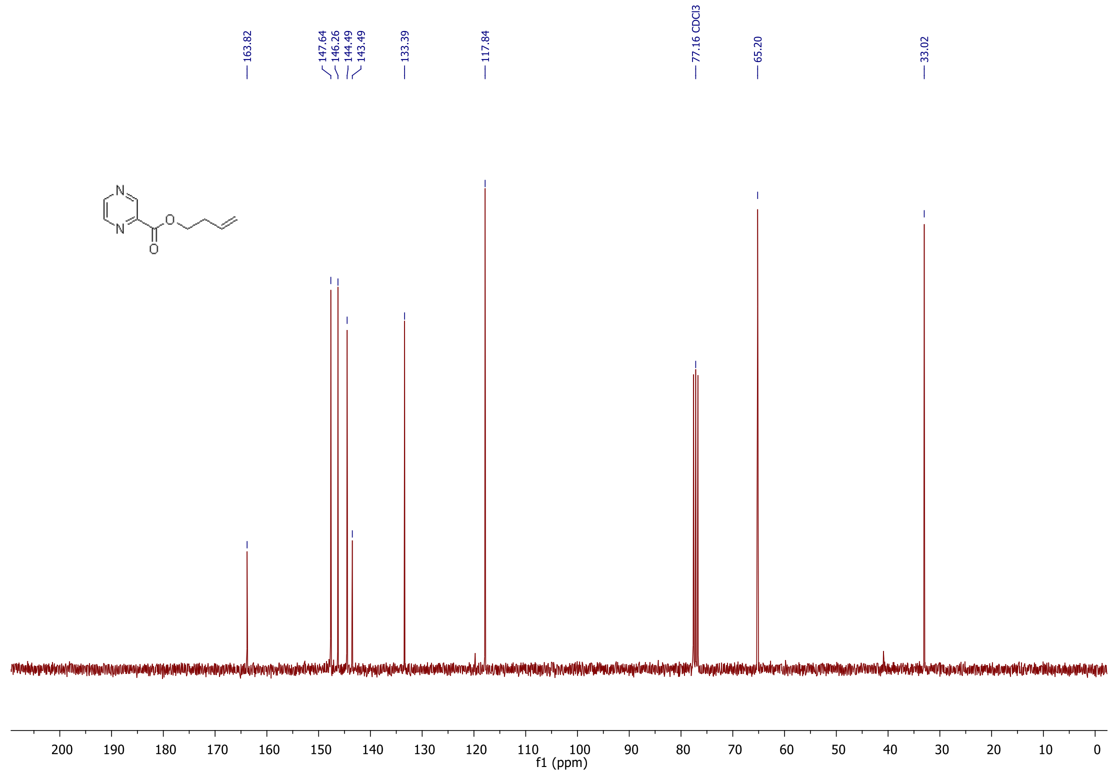

^{13}C NMR (75 MHz, $CDCl_3$)

But-3-en-1-yl furan-2-carboxylate (S20)

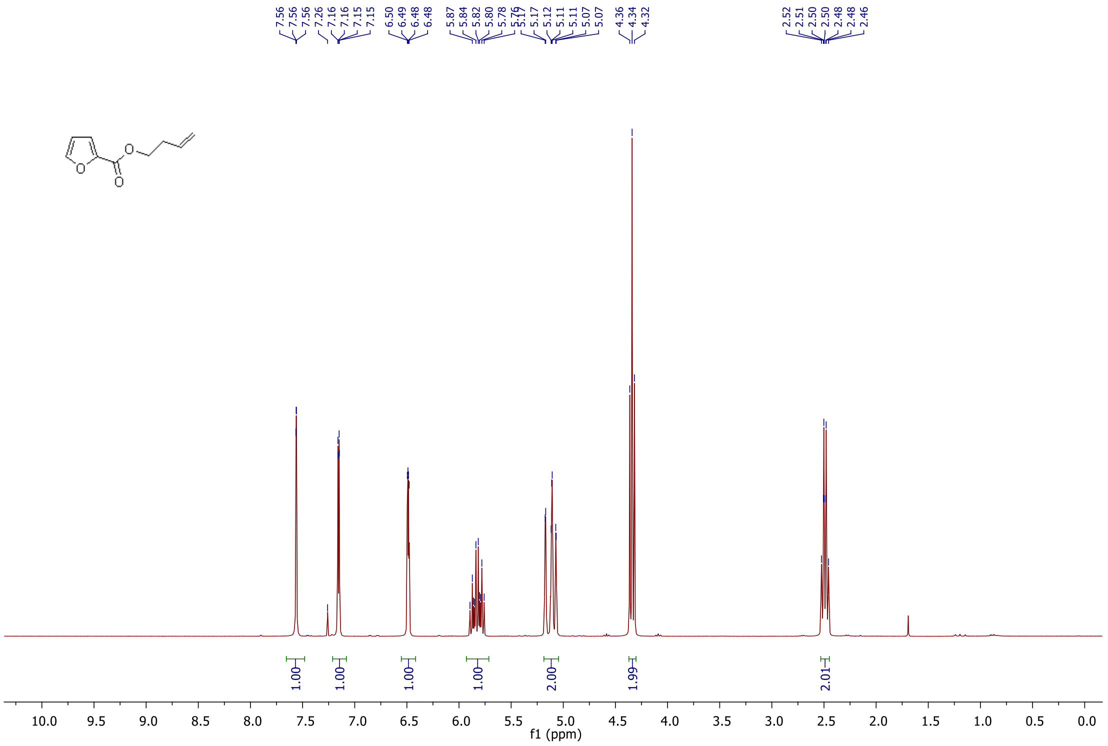

^{1}H NMR (300 MHz, $CDCl_3$)

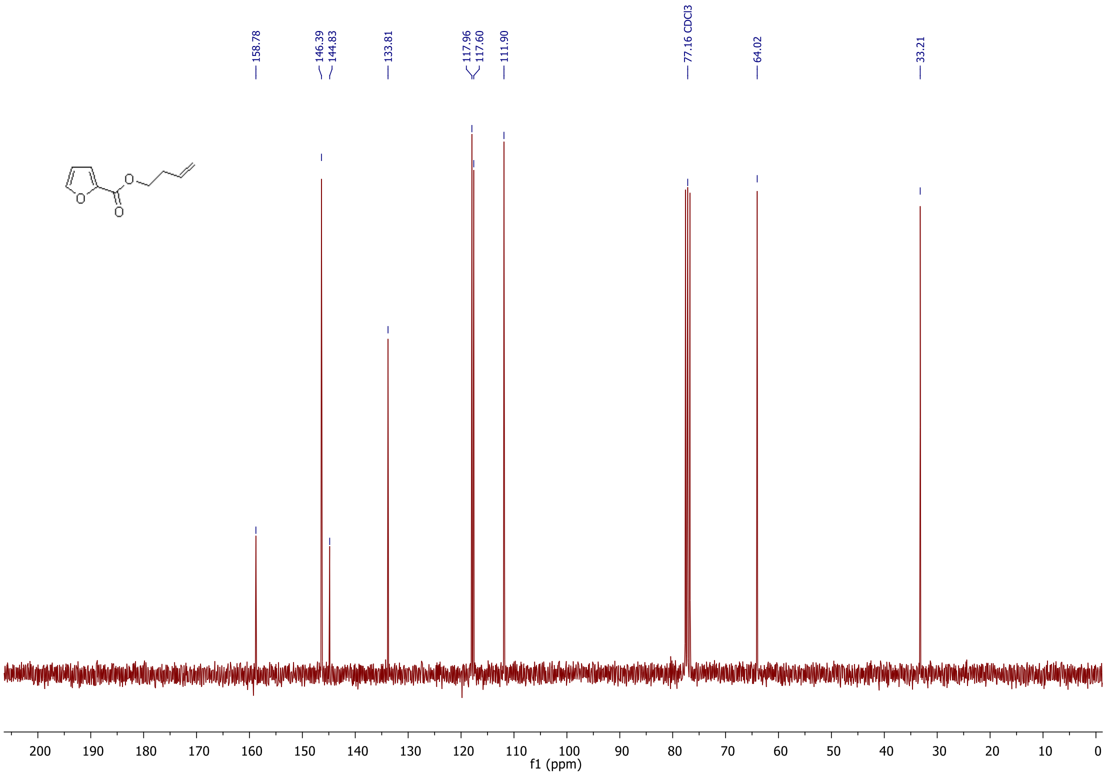

^{13}C NMR (75 MHz, $CDCl_3$)

(1*S*,2*R*,4*S*)-2-(Allyloxy)-1,3,3-trimethylbicyclo[2.2.1]heptane (S21)

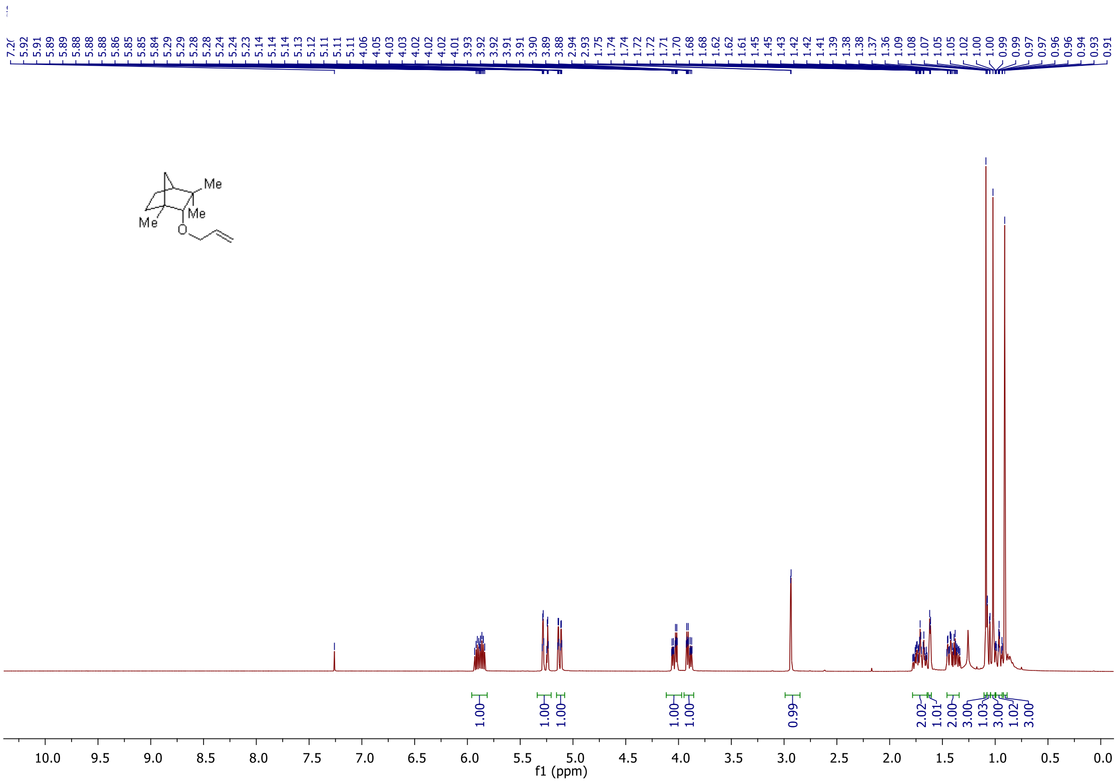

^{1}H NMR (400 MHz, $CDCl_3$)

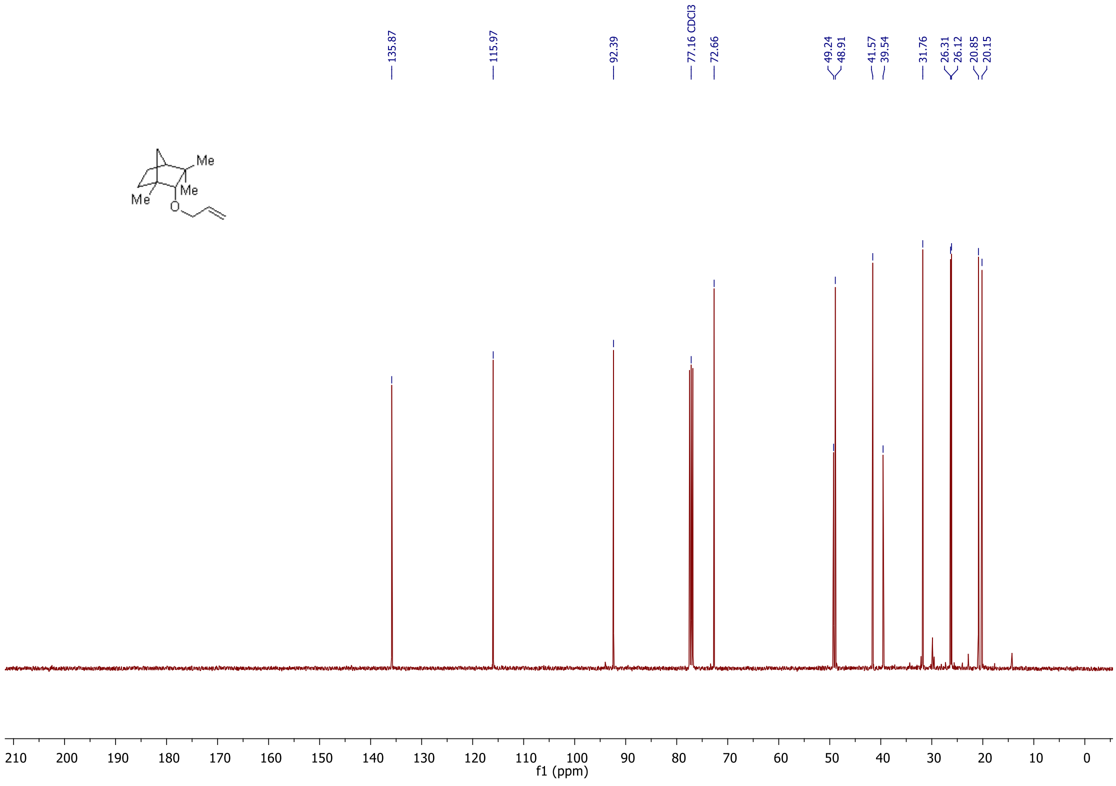

^{13}C NMR (75 MHz, $CDCl_3$)

(*E*)-Hex-4-en-1-yl-4-methylbenzenesulfonate (S26)

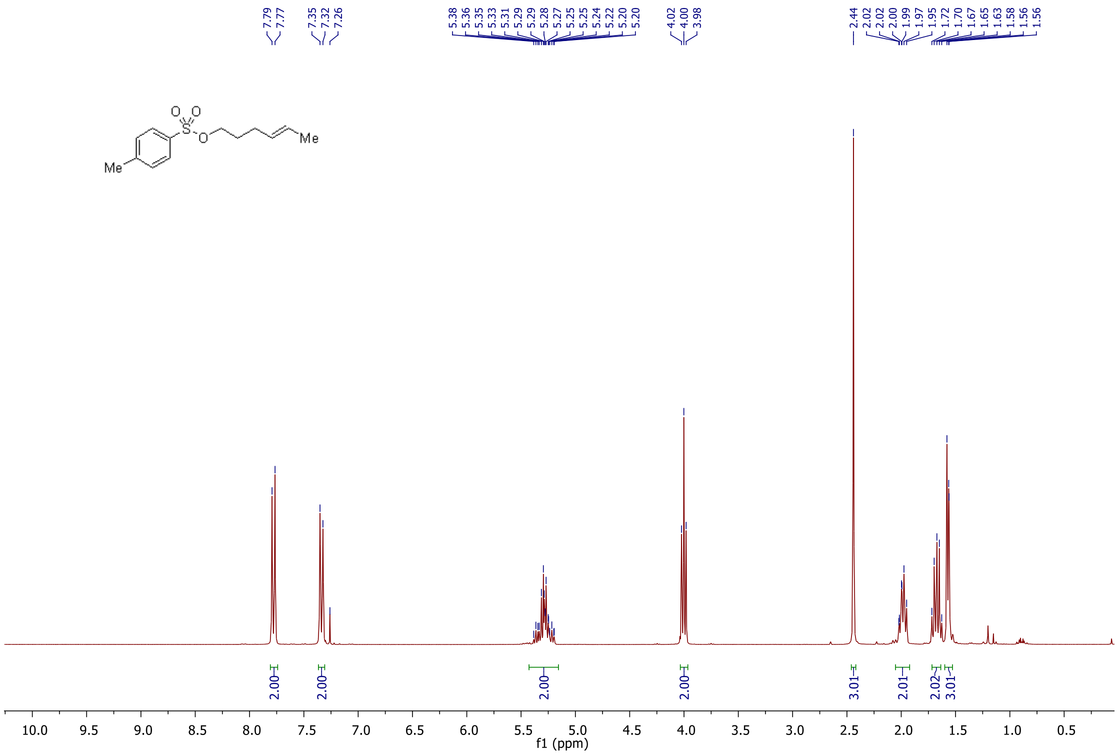

^{1}H NMR (300 MHz, $CDCl_3$)

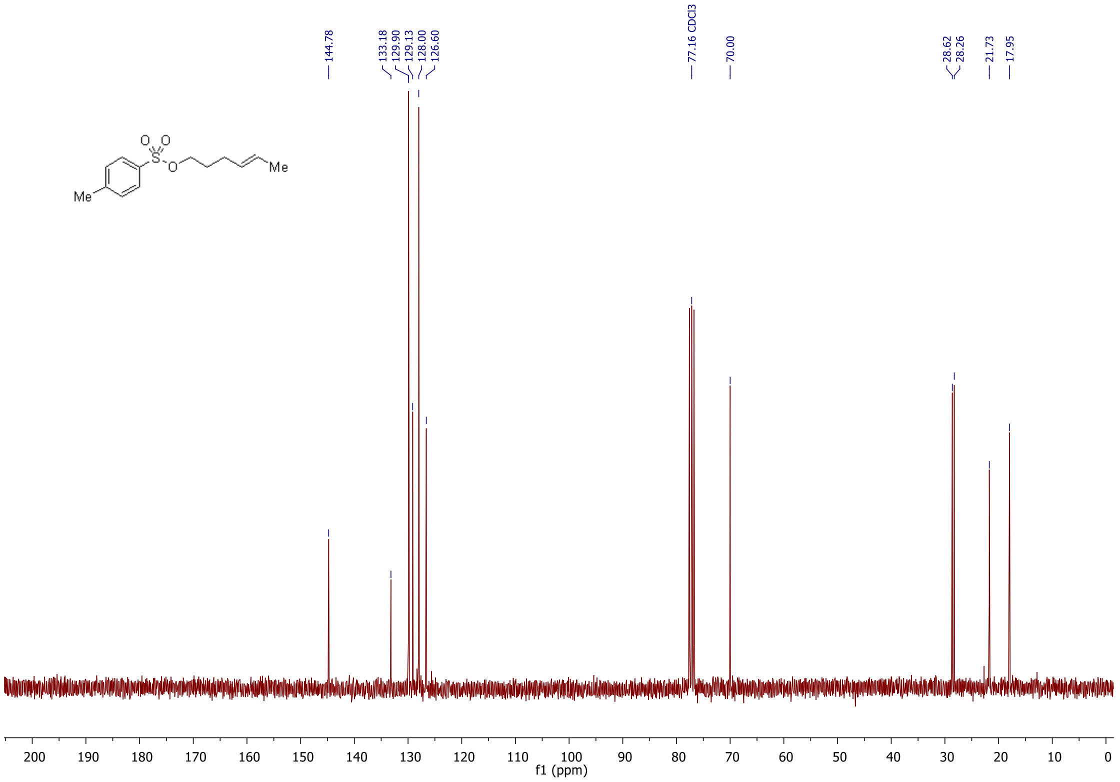

^{13}C NMR (75 MHz, $CDCl_3$)

(*Z*)-Hex-4-en-1-yl-4-methylbenzenesulfonate (S27)

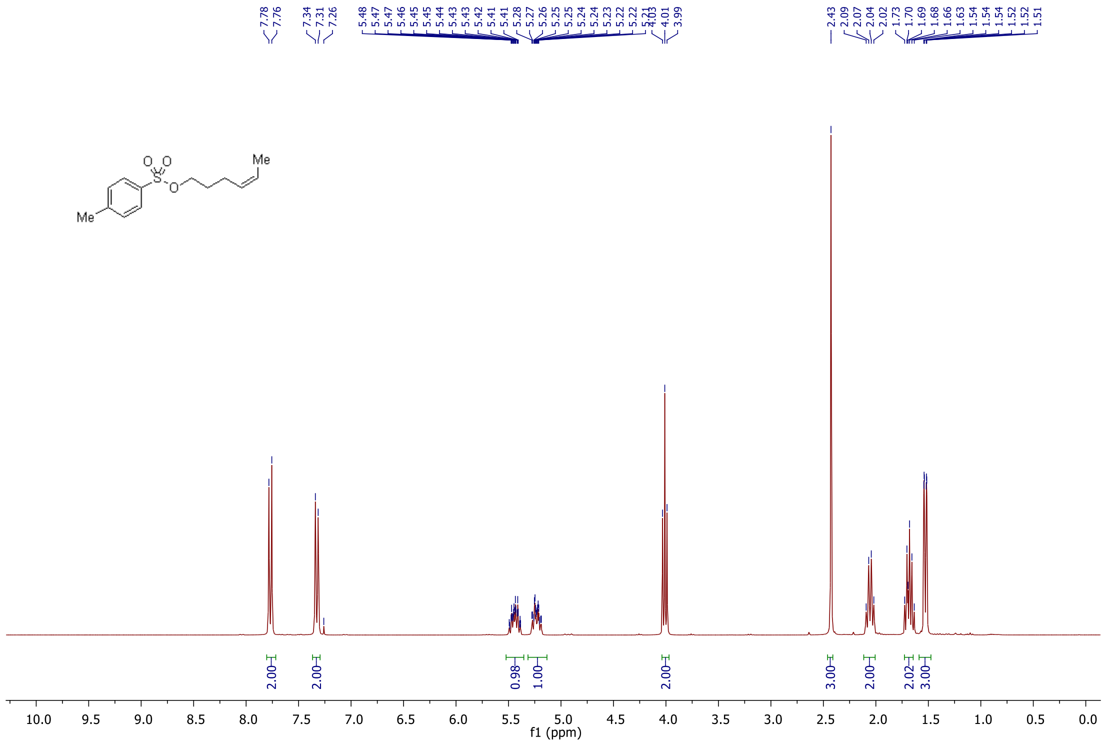

^{1}H NMR (300 MHz, $CDCl_3$)

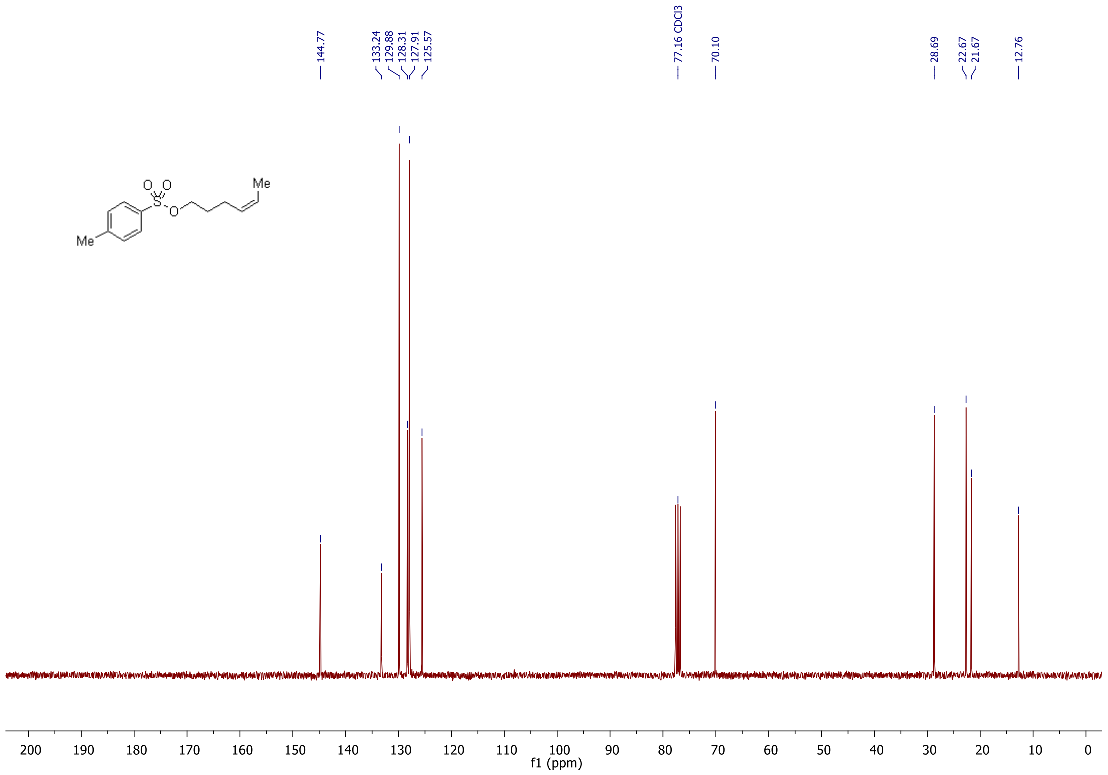

^{13}C NMR (75 MHz, $CDCl_3$)

(*E*)-Hex-4-en-1-yl-4-chlorobenzoate (S28)

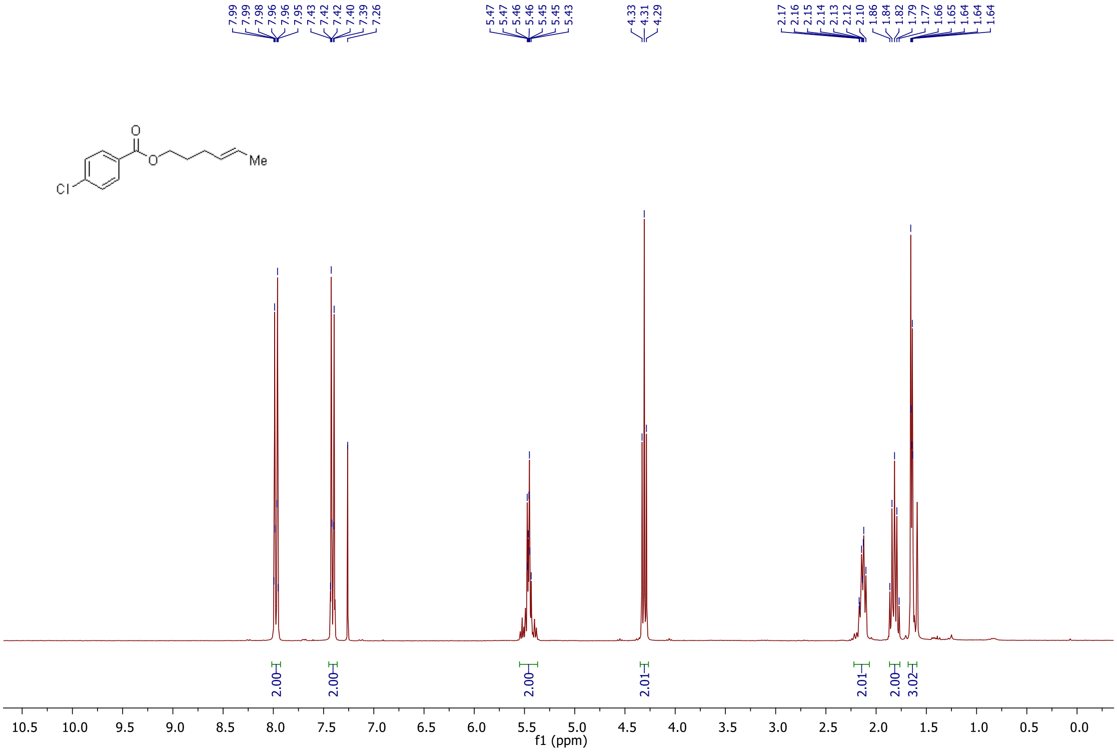

^{1}H NMR (300 MHz, $CDCl_3$)

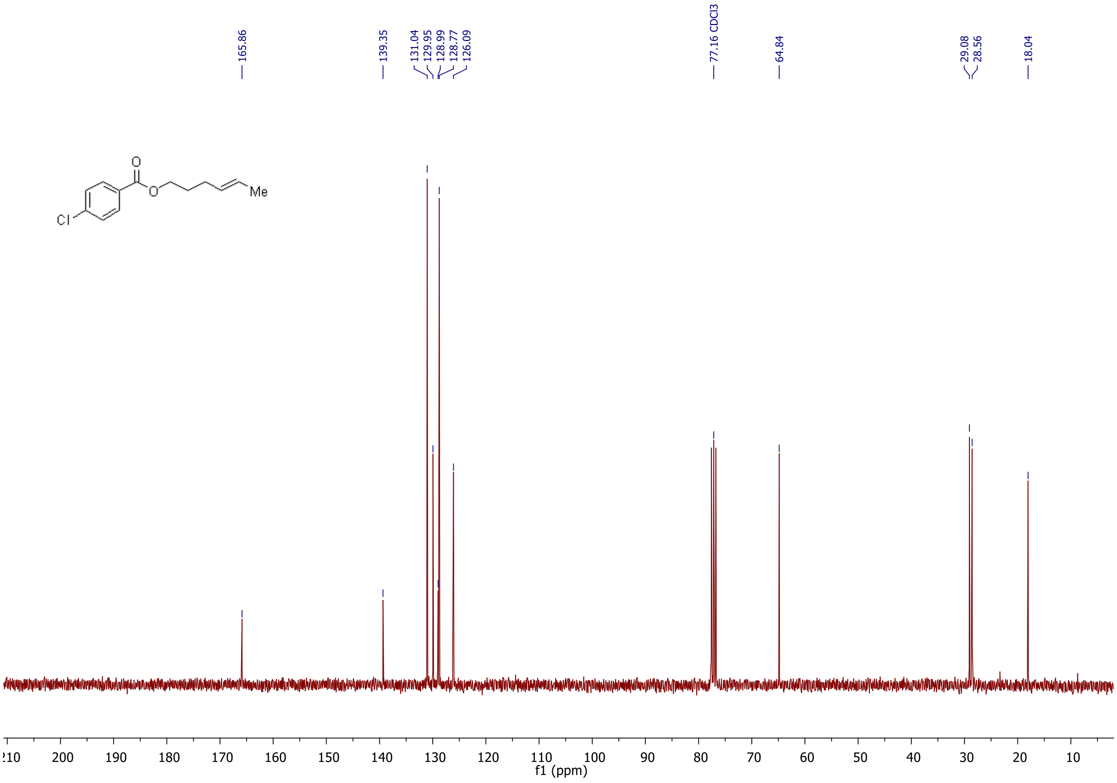

^{13}C NMR (75 MHz, $CDCl_3$)

(*Z*)-Hex-4-en-1-yl-4-chlorobenzoate (S29)

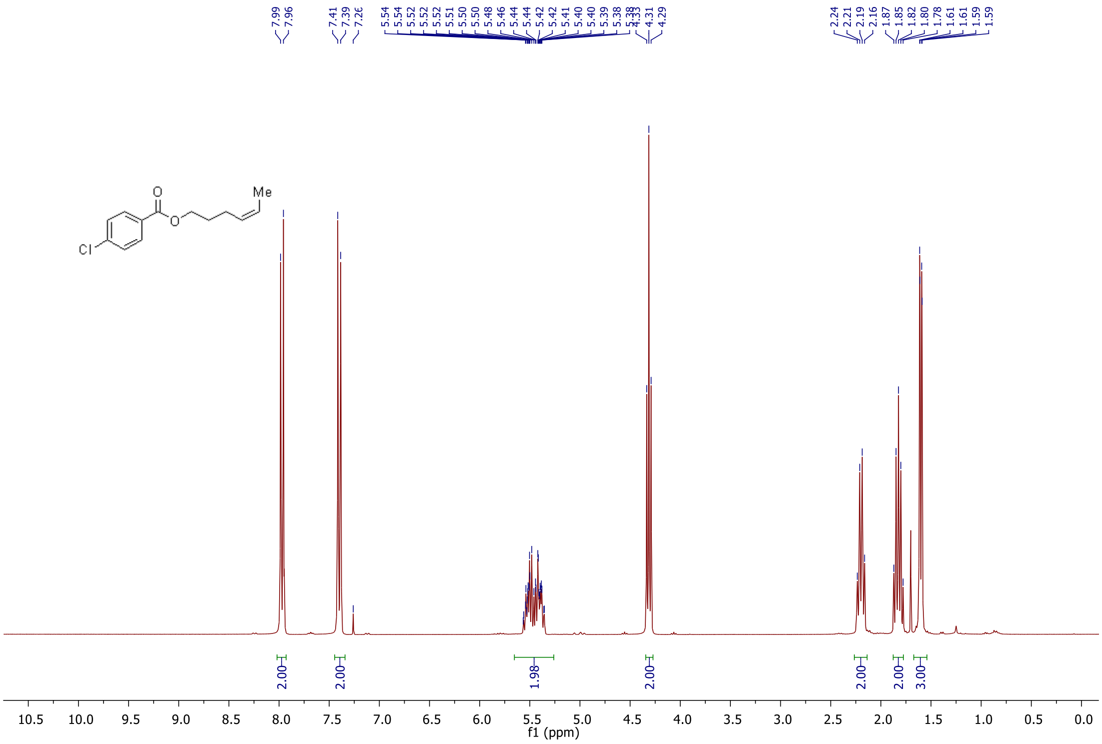

^{1}H NMR (300 MHz, $CDCl_3$)

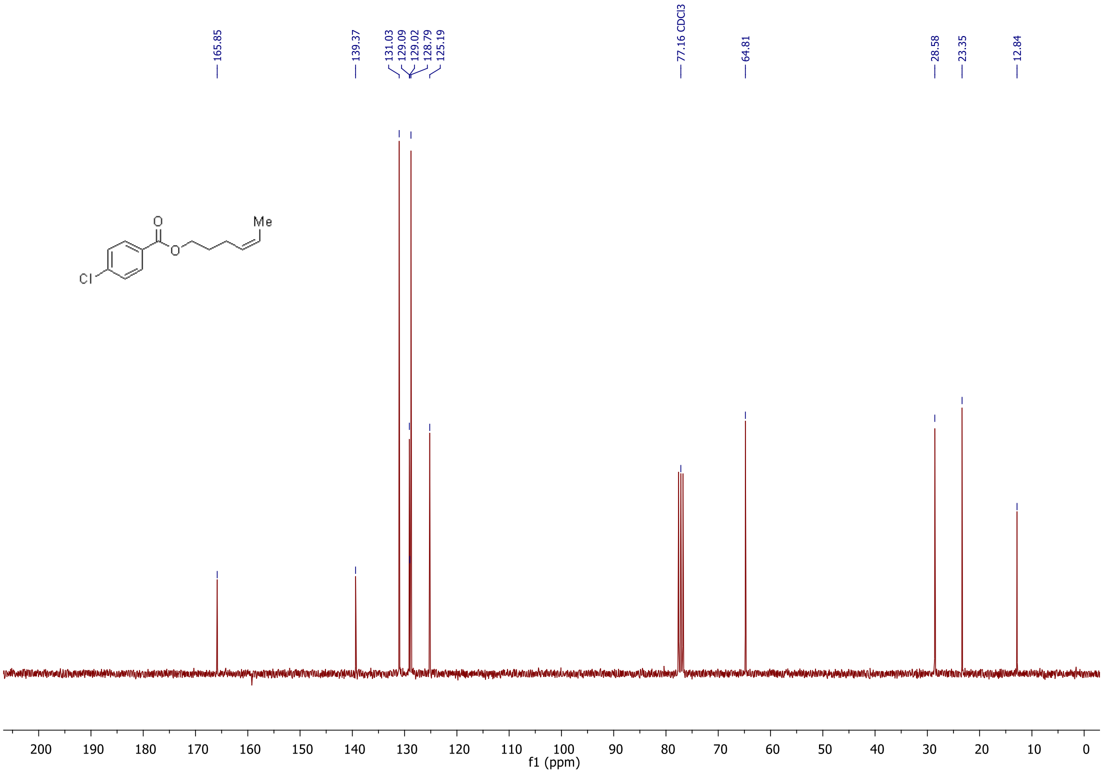

^{13}C NMR (75 MHz, $CDCl_3$)

(3,4-Dichlorobutyl)benzene (5)

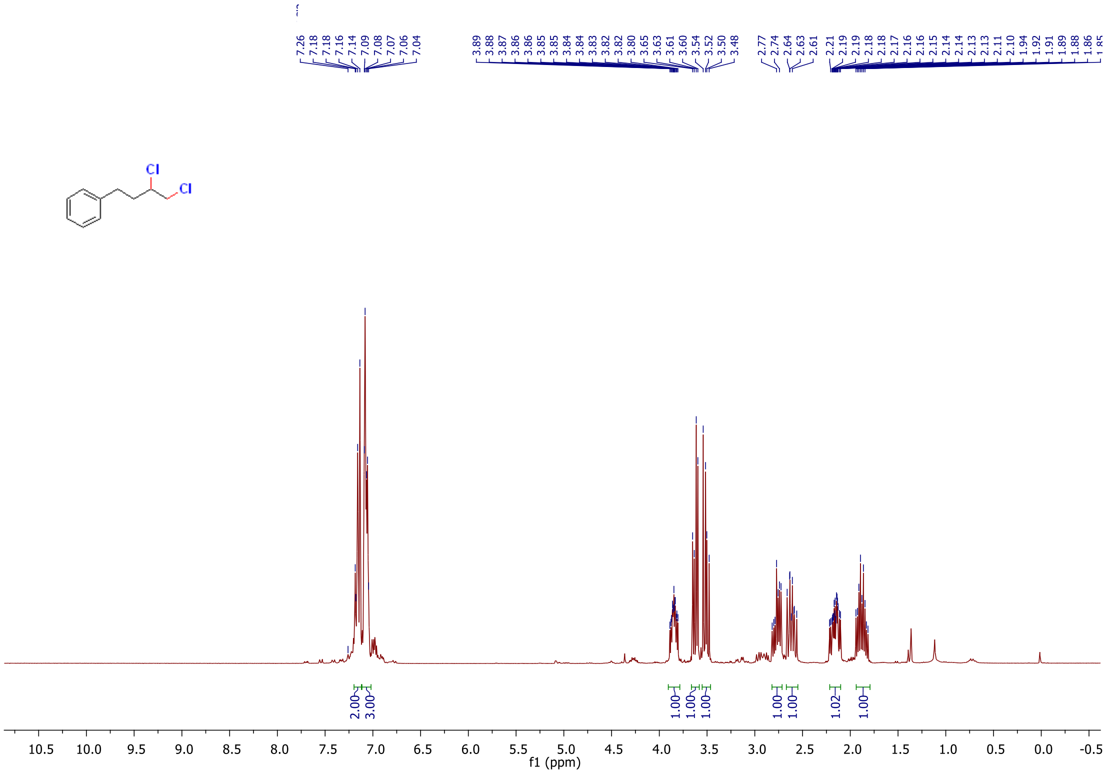

^{1}H NMR (300 MHz, $CDCl_3$)

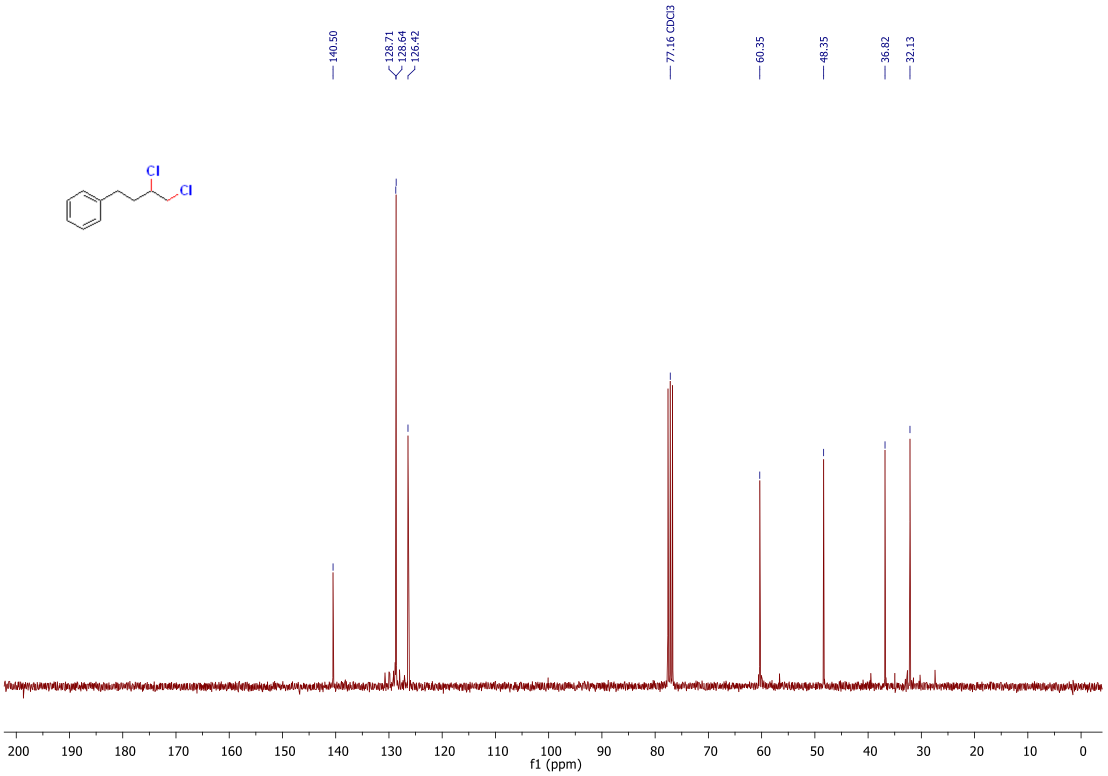

^{13}C NMR (75 MHz, $CDCl_3$)

1,2-Dichlorodecane (6)

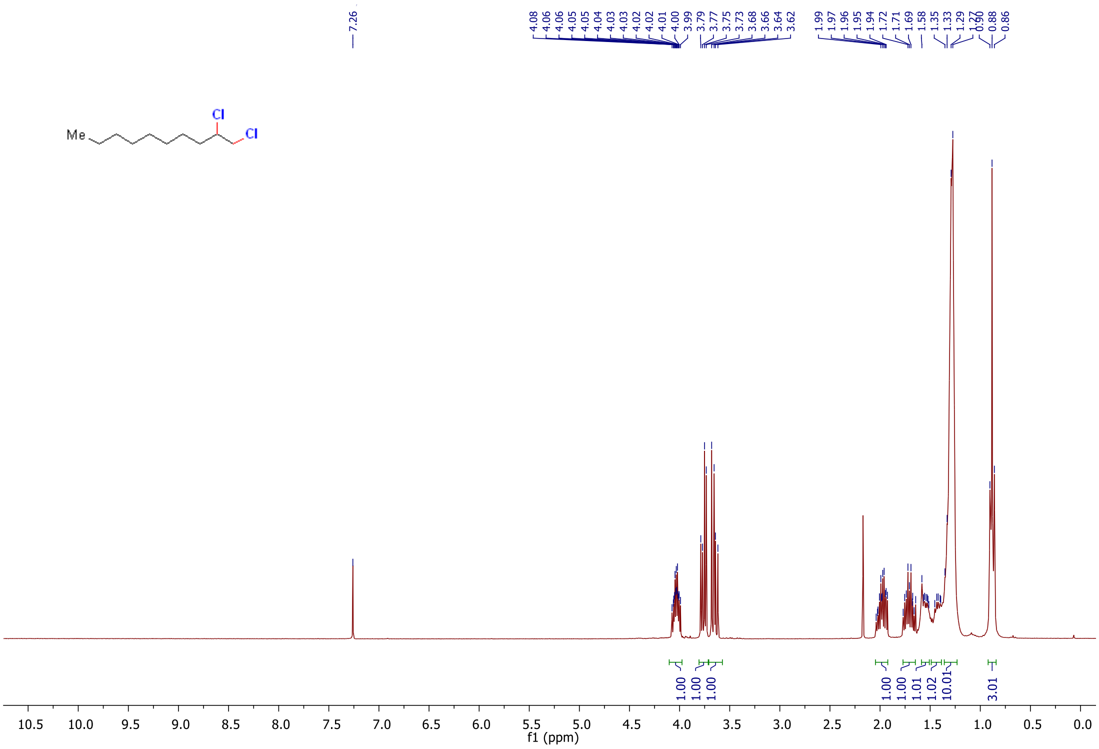

^{1}H NMR (300 MHz, $CDCl_3$)

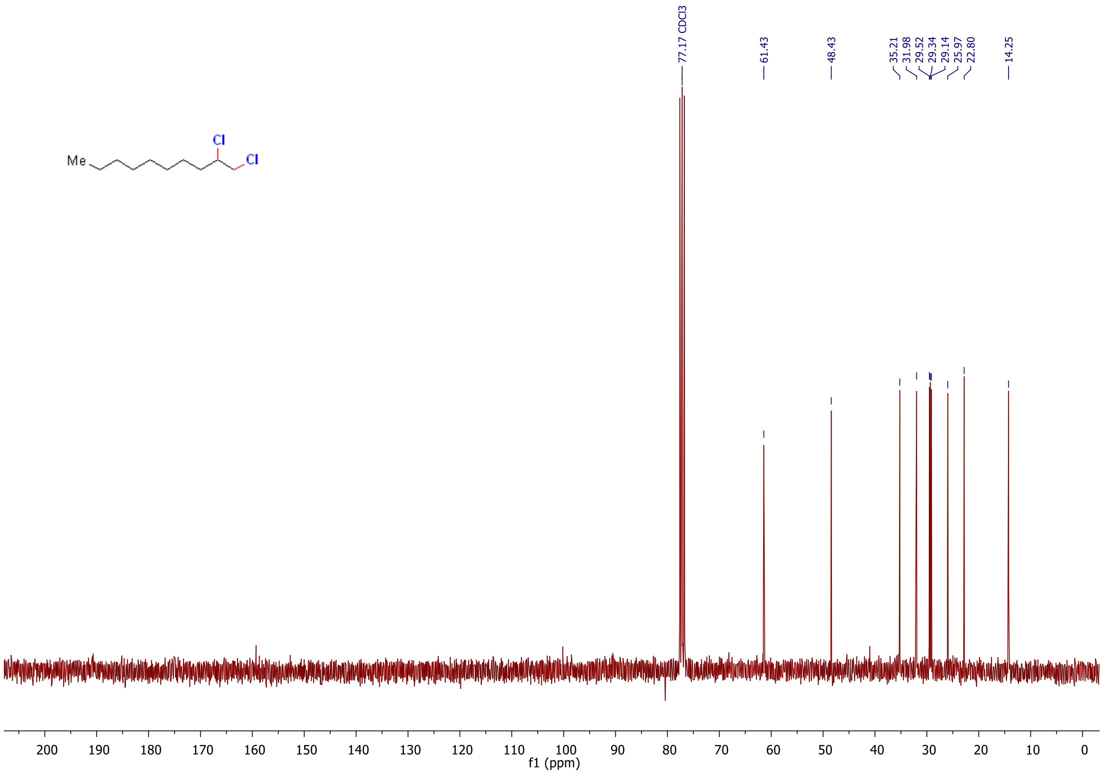

^{13}C NMR (75 MHz, $CDCl_3$)

4,5-Dichloropentyl benzoate (7)

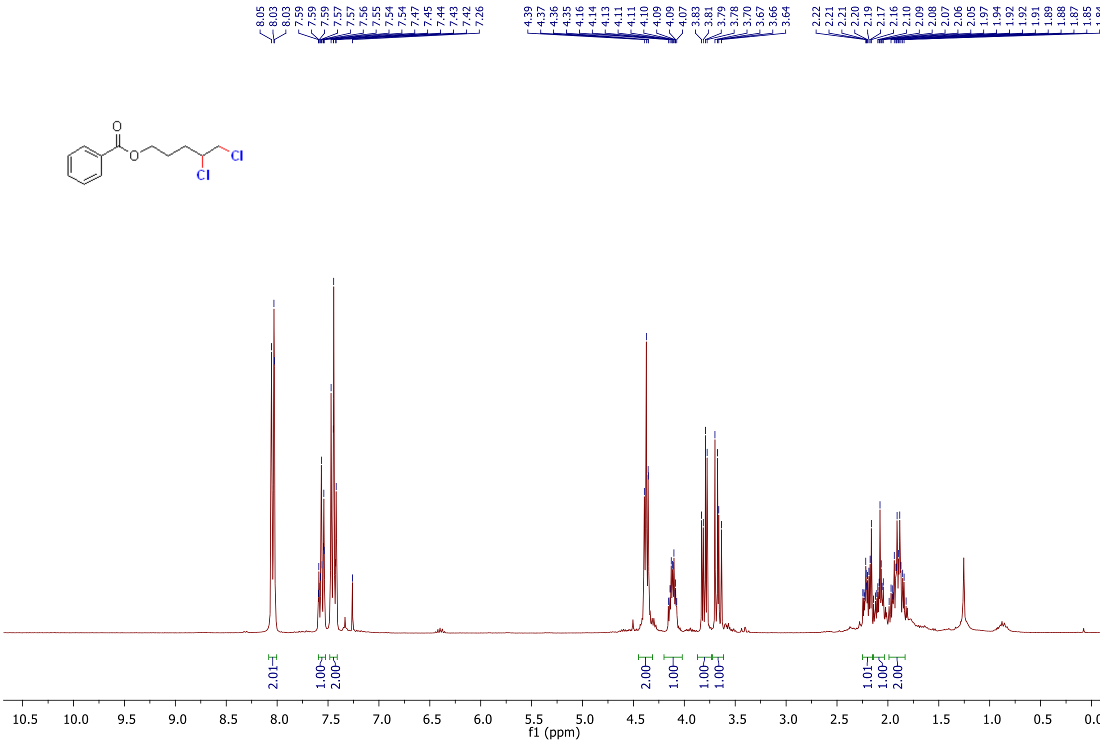

^{1}H NMR (300 MHz, $CDCl_3$)

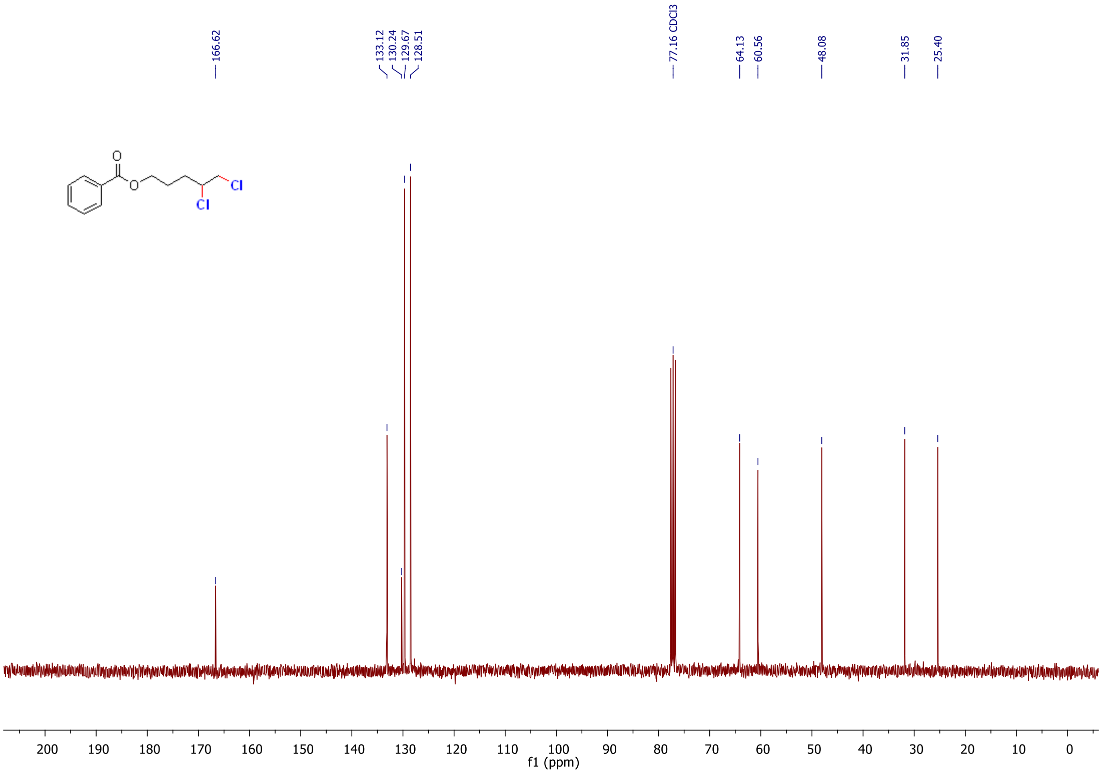

^{13}C NMR (75 MHz, $CDCl_3$)

3,4-Dichlorobutyl benzoate (8)

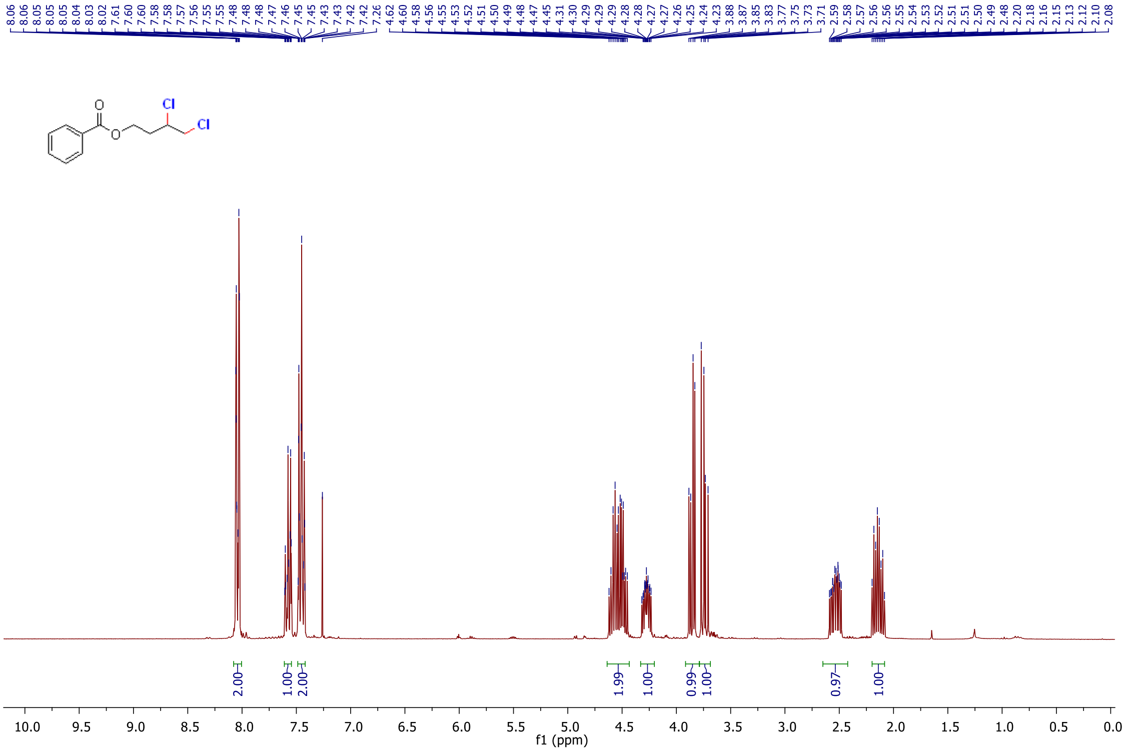

^{1}H NMR (300 MHz, $CDCl_3$)

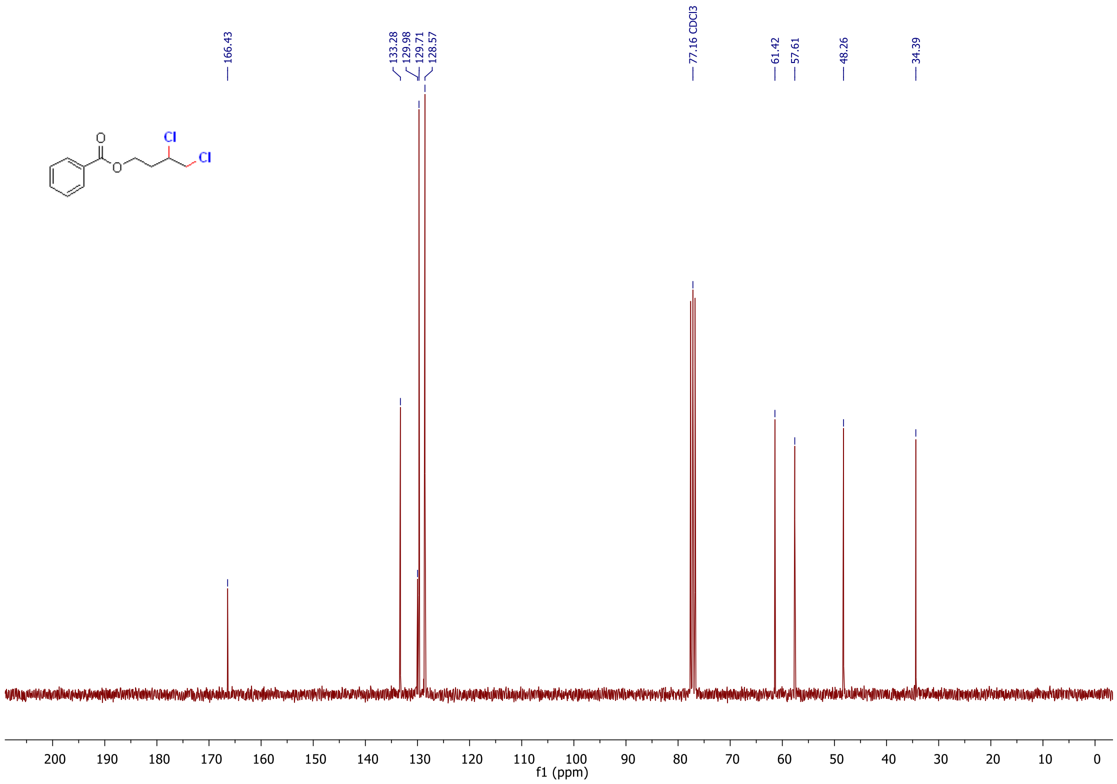

^{13}C NMR (75 MHz, $CDCl_3$)

2-Cyanoethyl 4,5-dichloropentanoate (9)

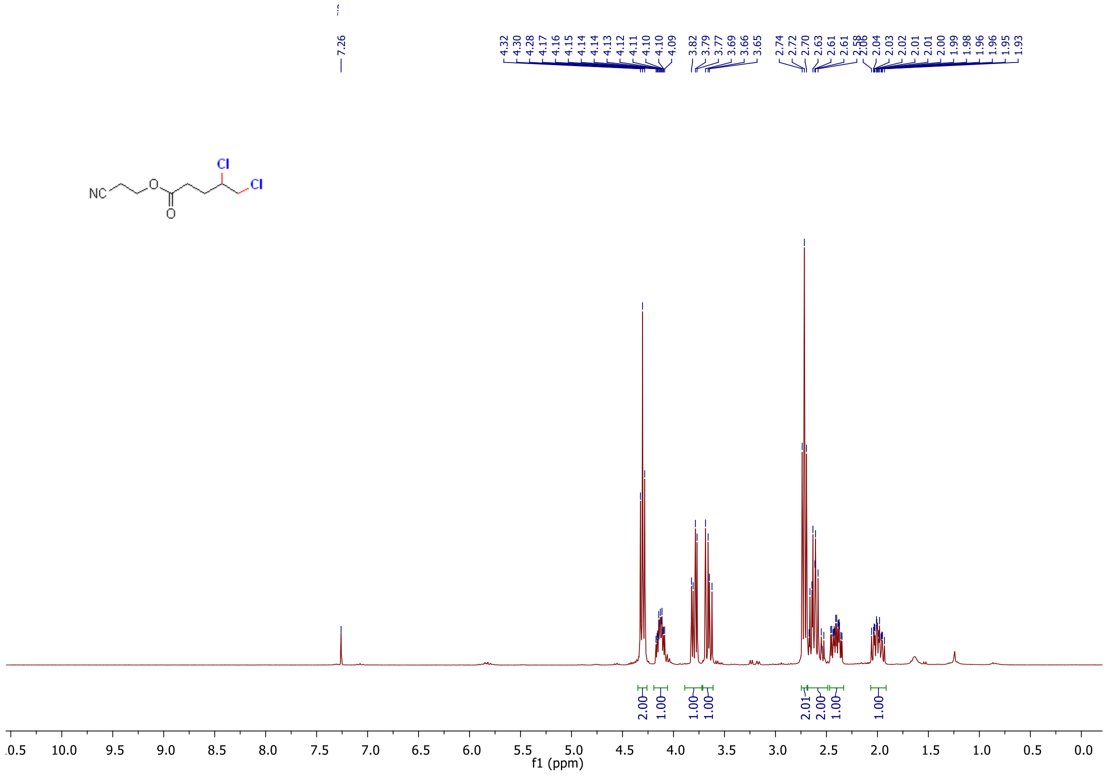

^{1}H NMR (300 MHz, $CDCl_3$)

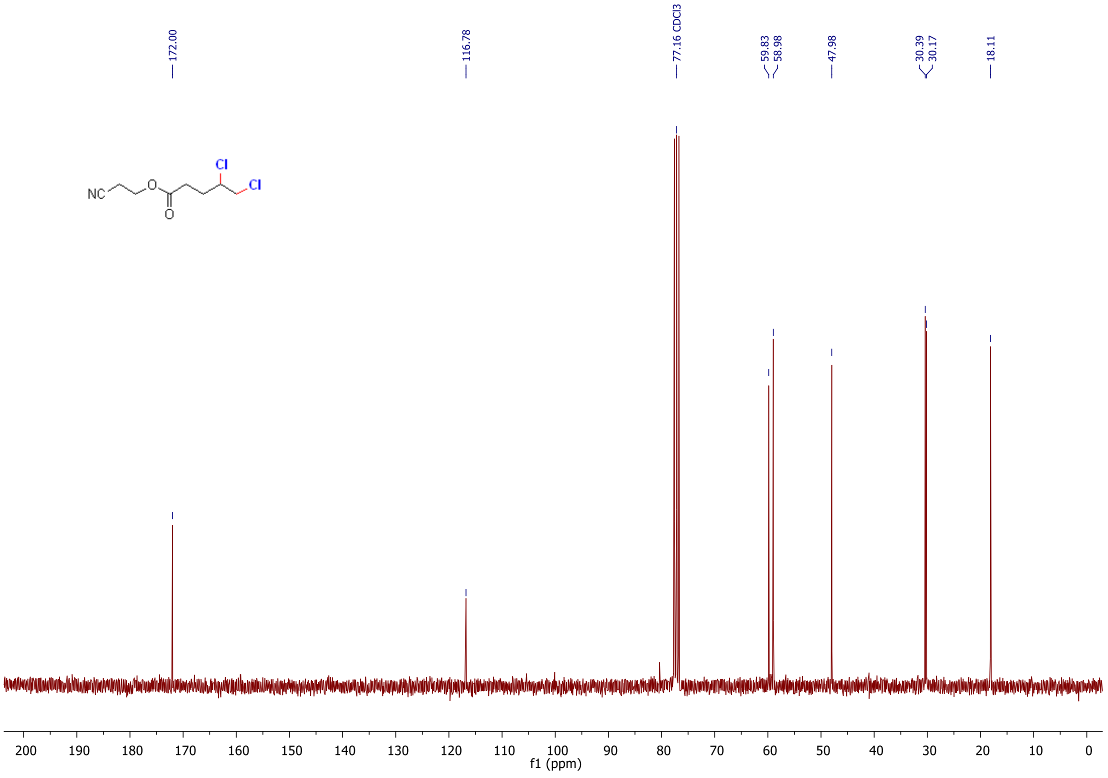

^{13}C NMR (75 MHz, $CDCl_3$)

Benzyl 4,5-dichloropentanoate (10)

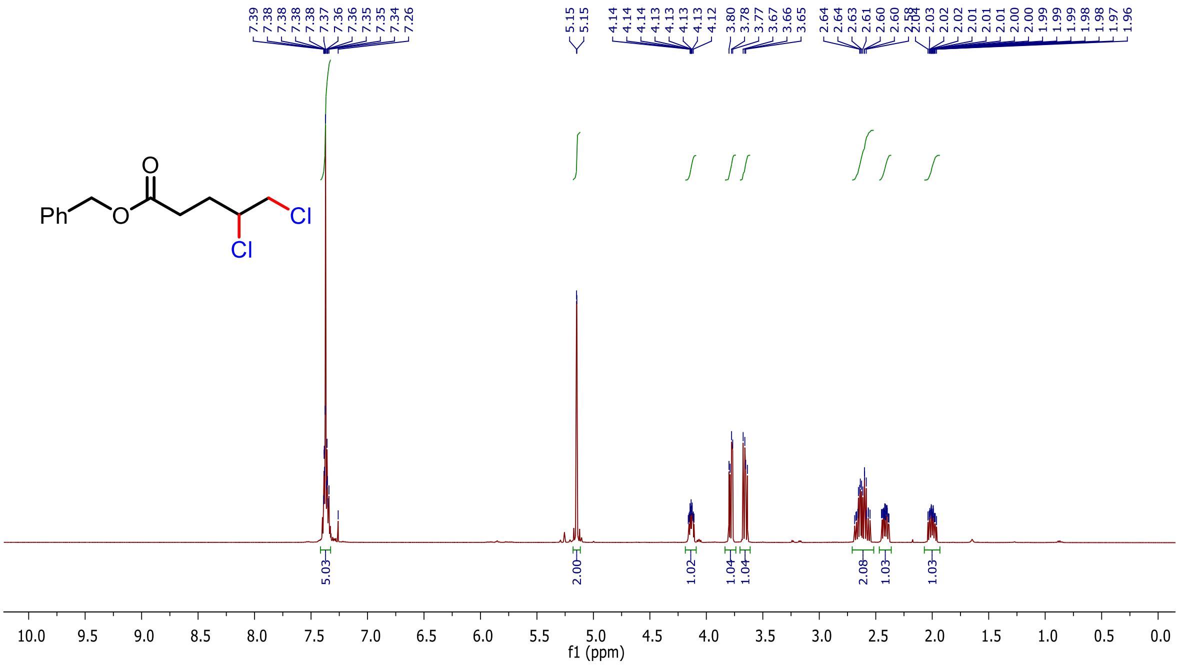

^{1}H NMR (500 MHz, $CDCl_3$)

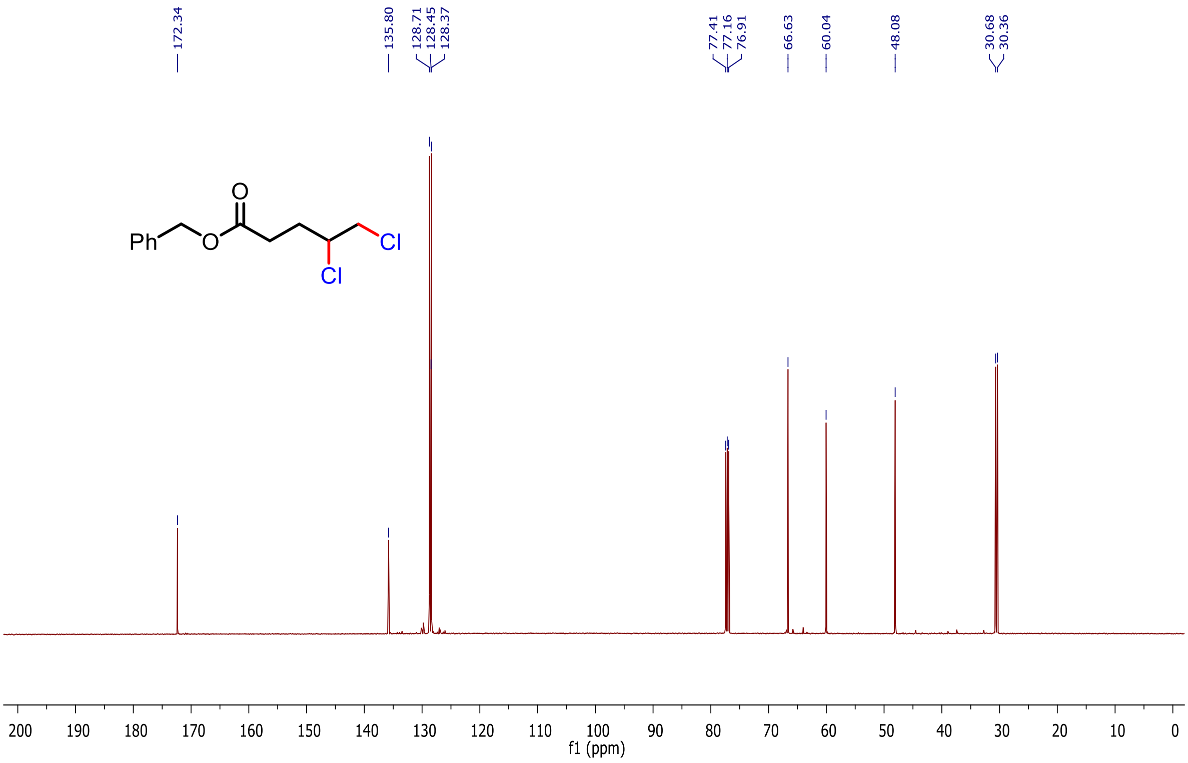

^{13}C NMR (126 MHz, $CDCl_3$)

4,5-Dichloropentanoic acid

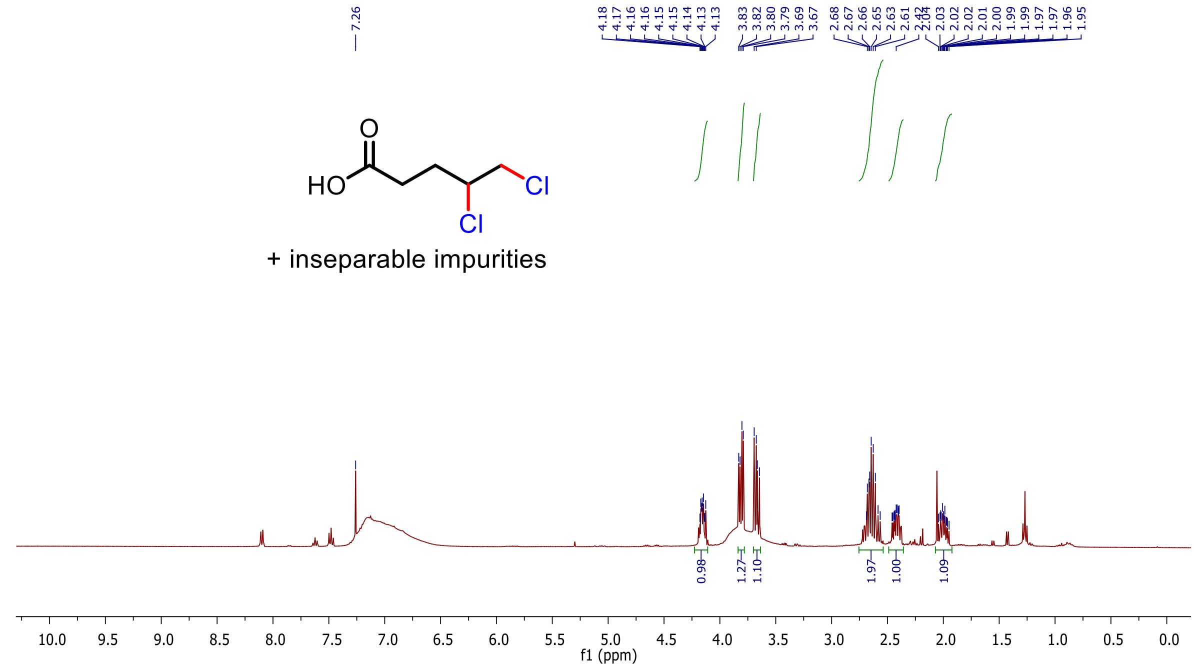

^{1}H NMR (400 MHz, $CDCl_3$)

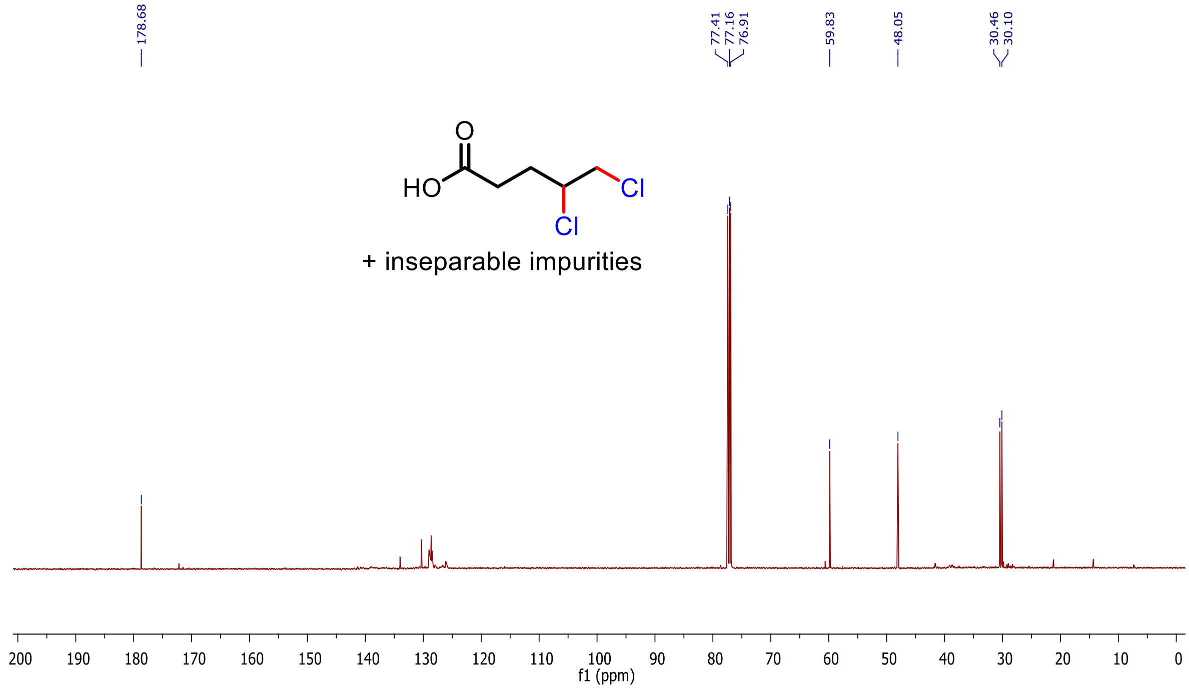

^{13}C NMR (126 MHz, $CDCl_3$)

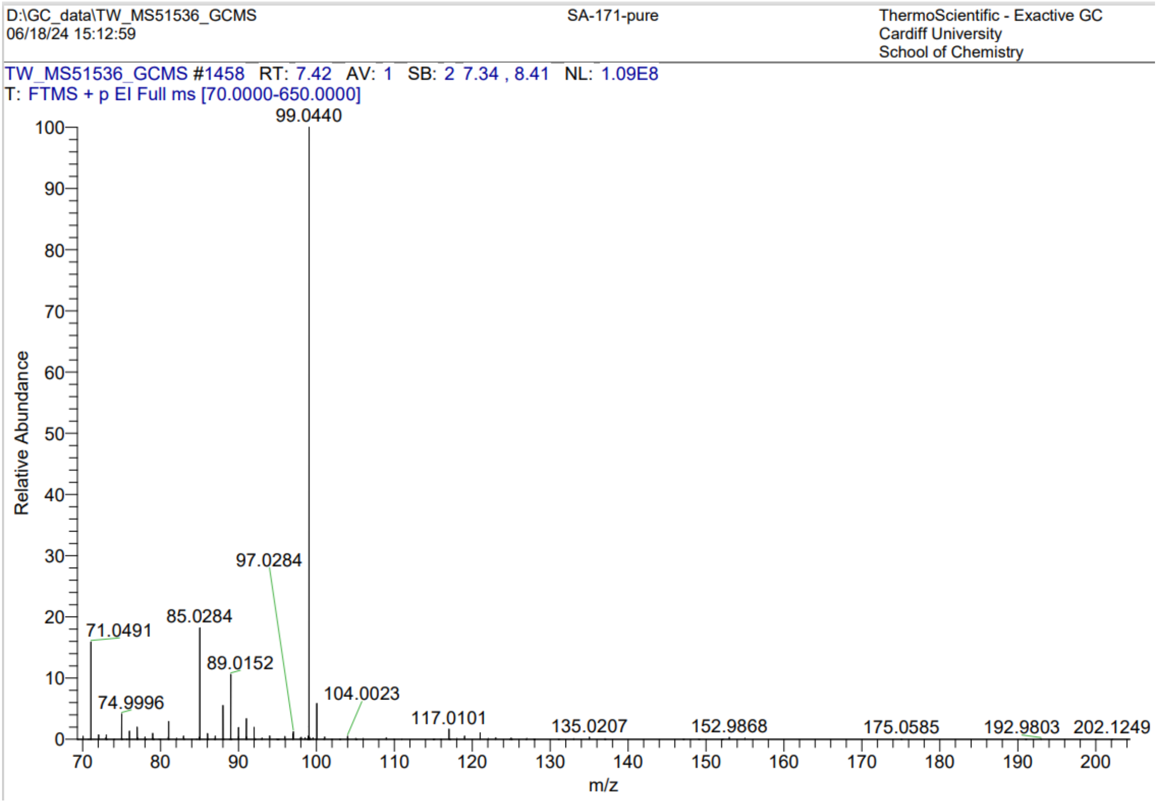

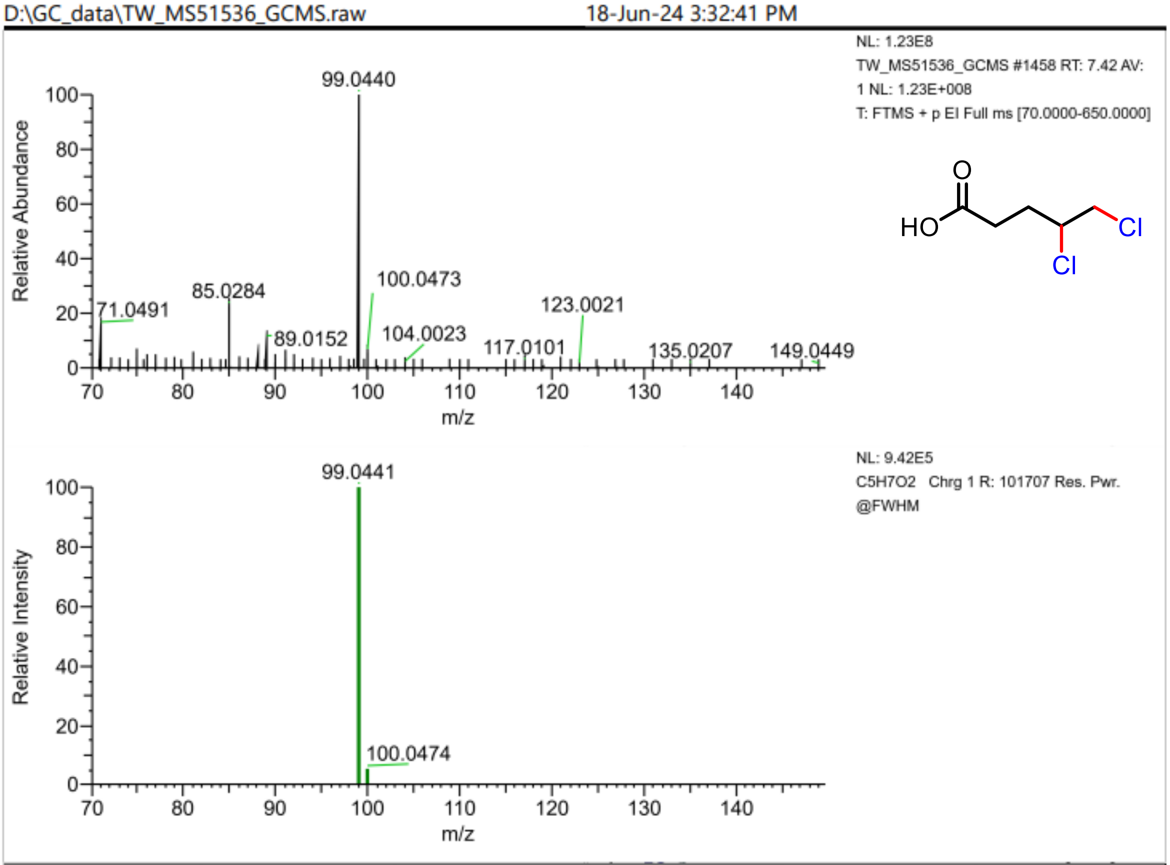

Peak Mass	Display Formula	Delta [ppm]	Theo. mass	Combined Score	MSMS Matched Fr...
99.0440	$C_5H_7O_2$	-0.42	99.04406	97.9411982330823	(Collection)

9,10-Dichlorodecan-1-ol (11)

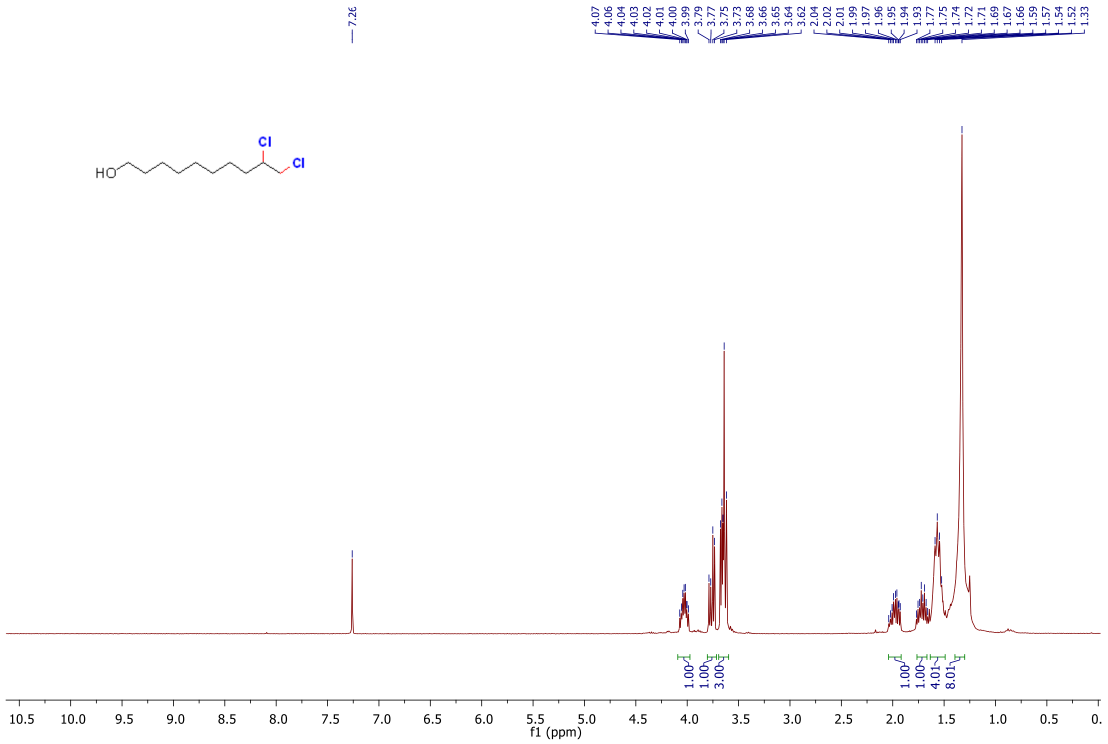

^{1}H NMR (300 MHz, $CDCl_3$)

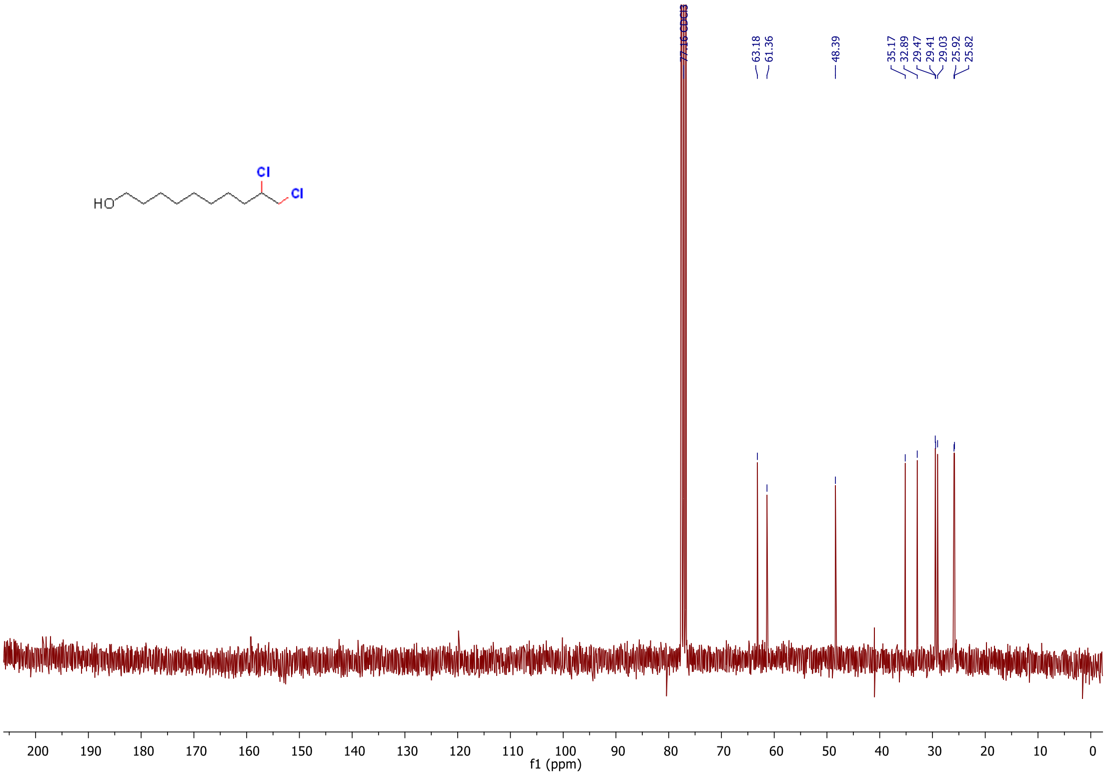

^{13}C NMR (75 MHz, $CDCl_3$)

10,11-Dichloroundecanoic acid (12)

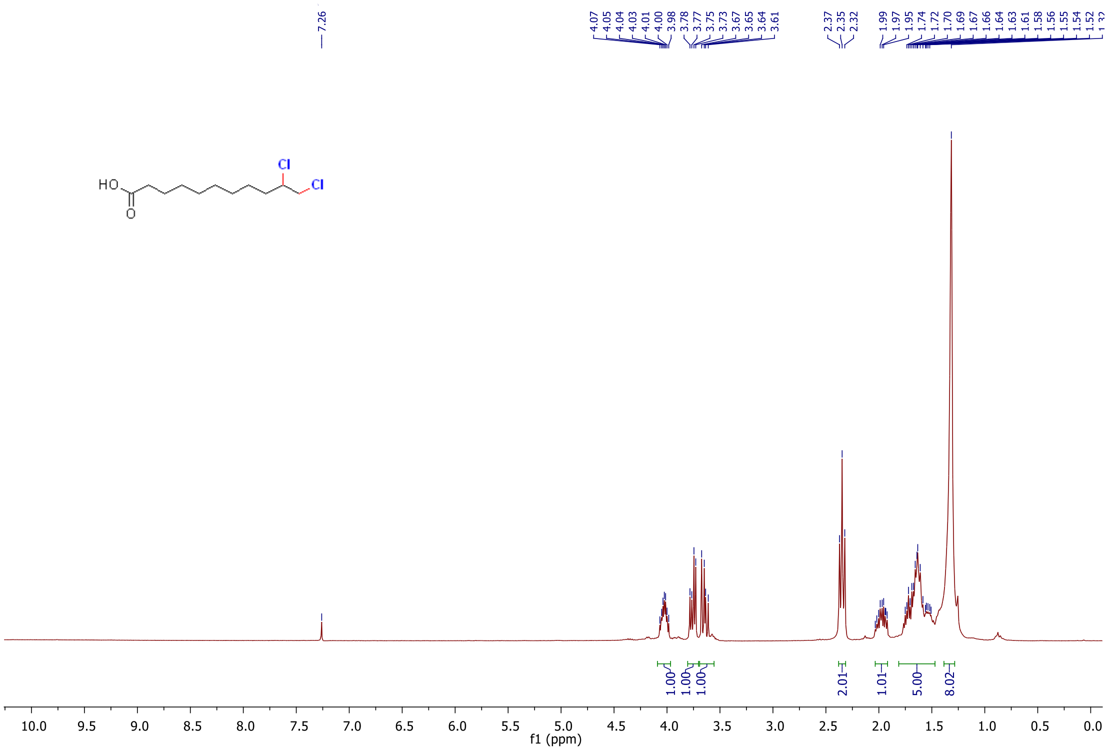

^{1}H NMR (300 MHz, $CDCl_3$)

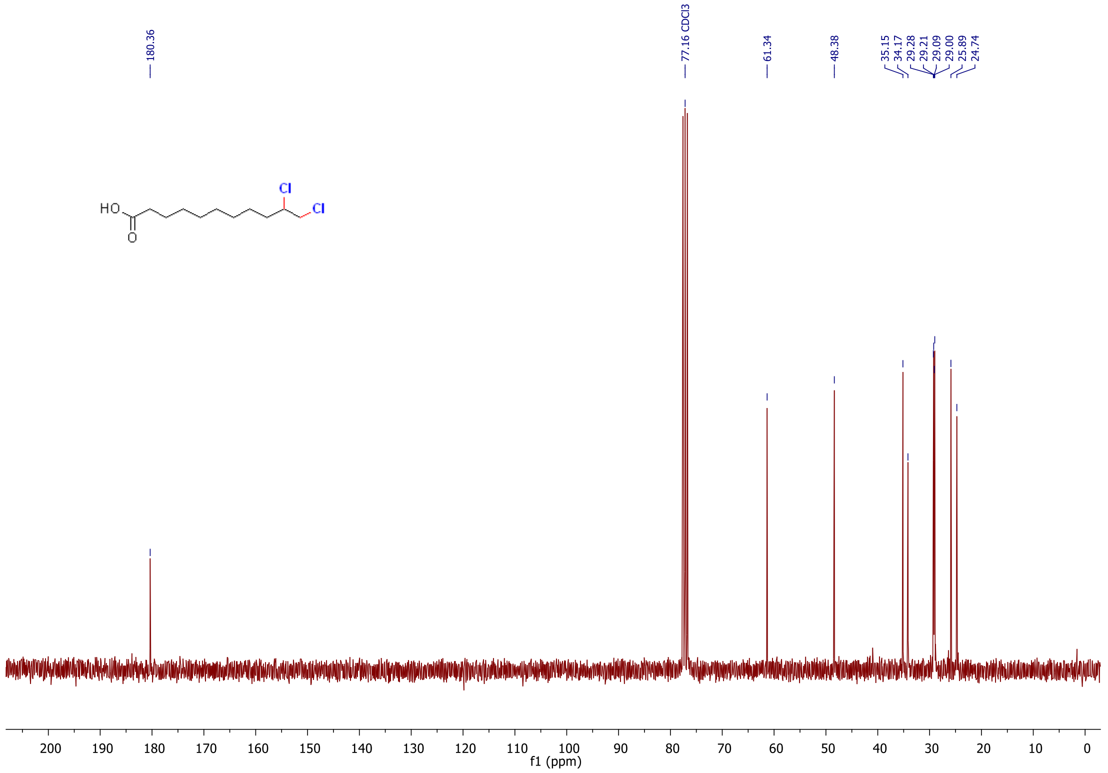

^{13}C NMR (75 MHz, $CDCl_3$)

1-Bromo-2-((2,3-dichloropropoxy)methyl)benzene (13)

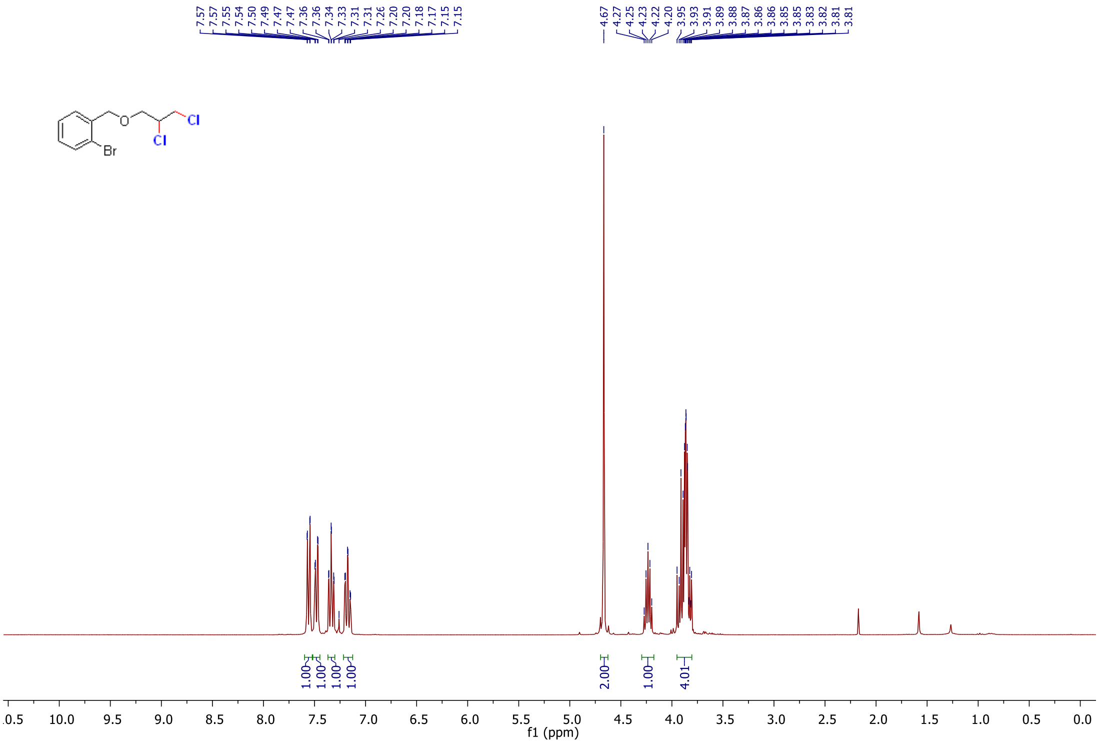

^{1}H NMR (300 MHz, $CDCl_3$)

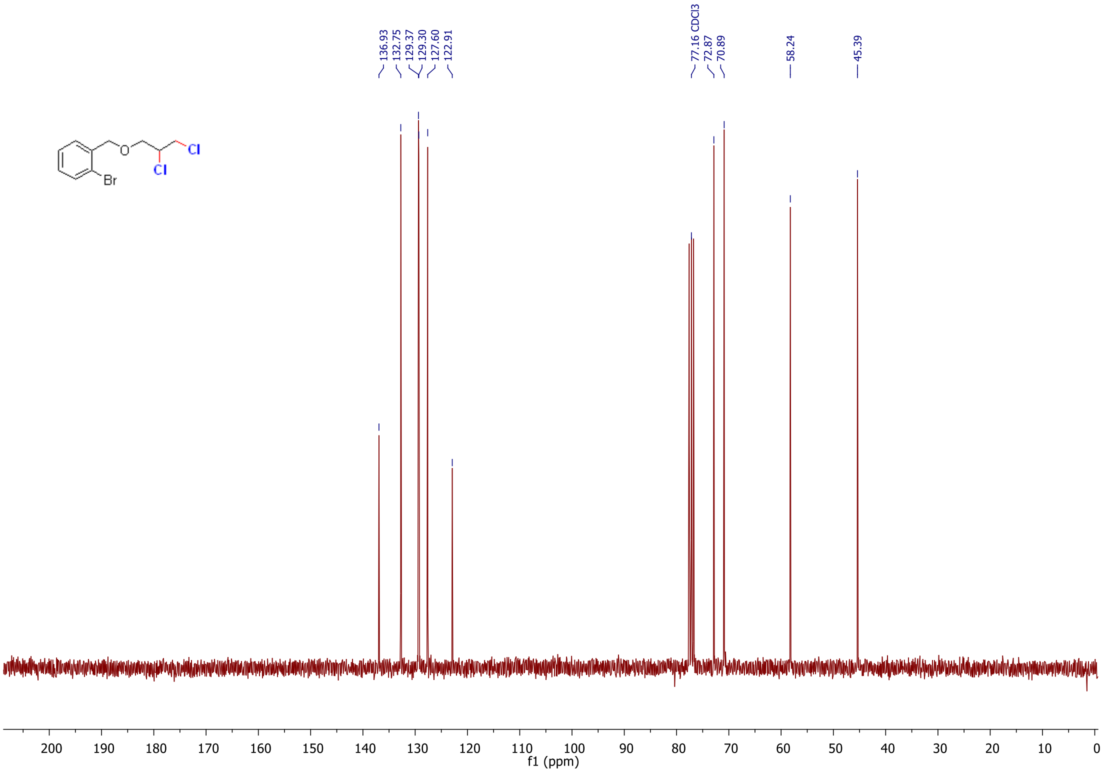

^{13}C NMR (75 MHz, $CDCl_3$)

3,4-Dichlorobutyl 2-iodobenzoate (14)

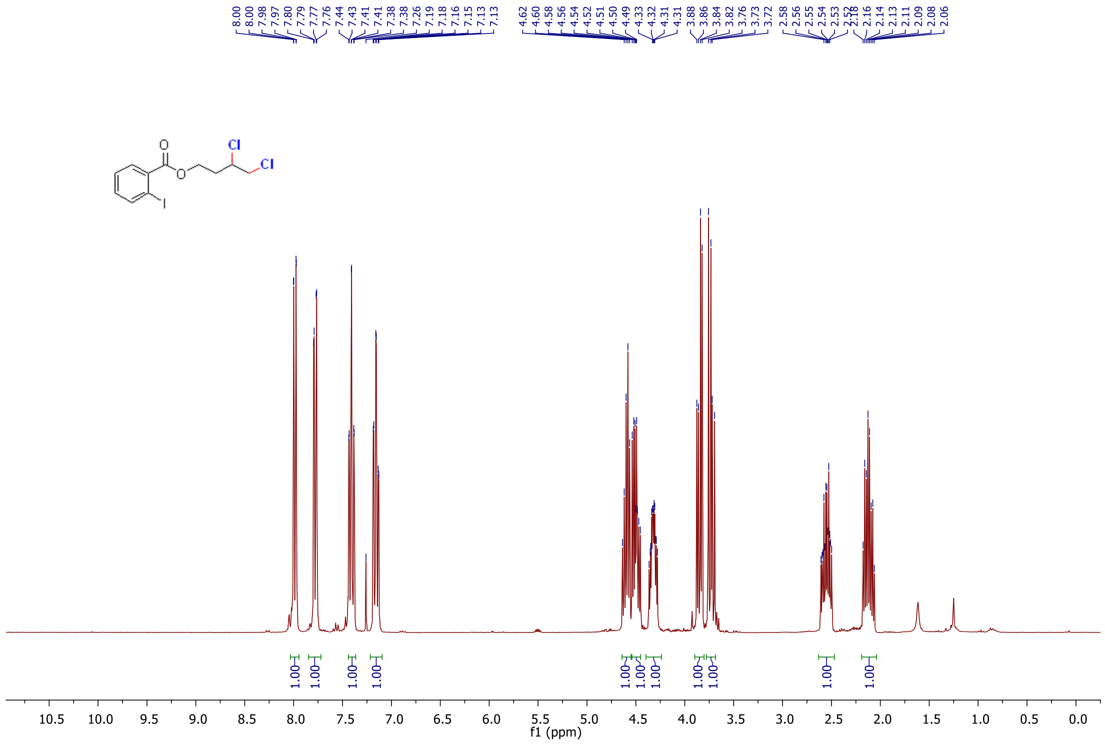

^{1}H NMR (300 MHz, $CDCl_3$)

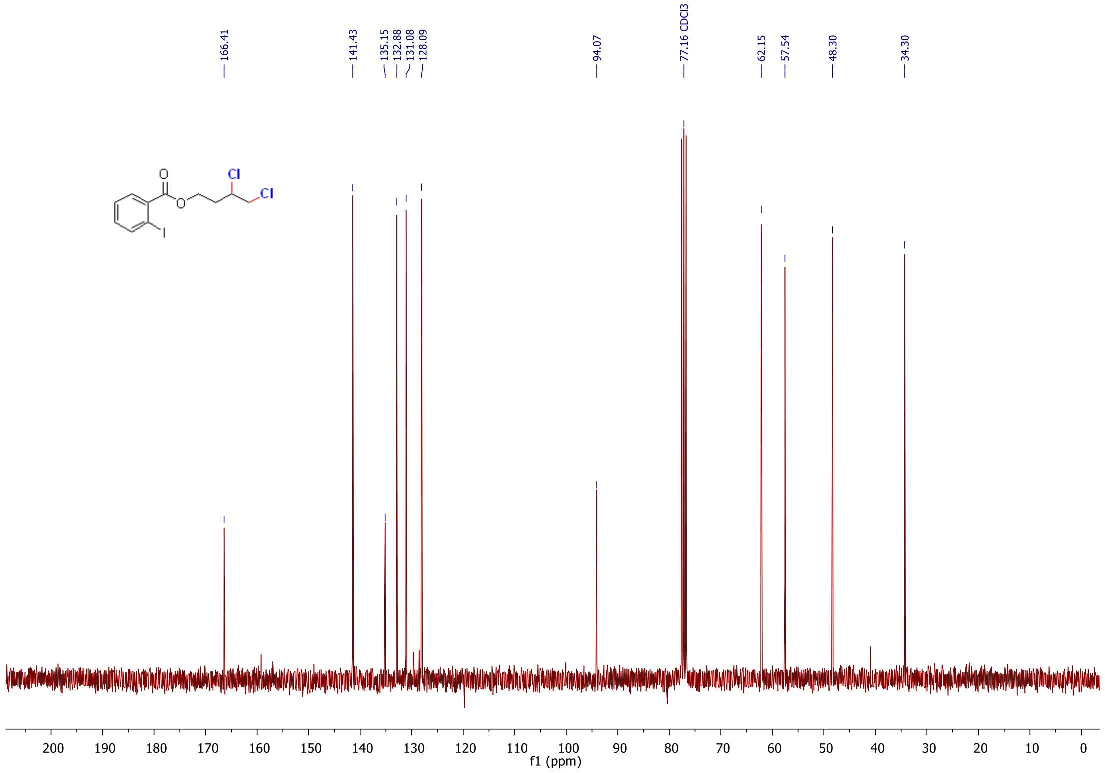

^{13}C NMR (75 MHz, $CDCl_3$)

5,6-Dichlorohexyl but-2-ynoate (15)

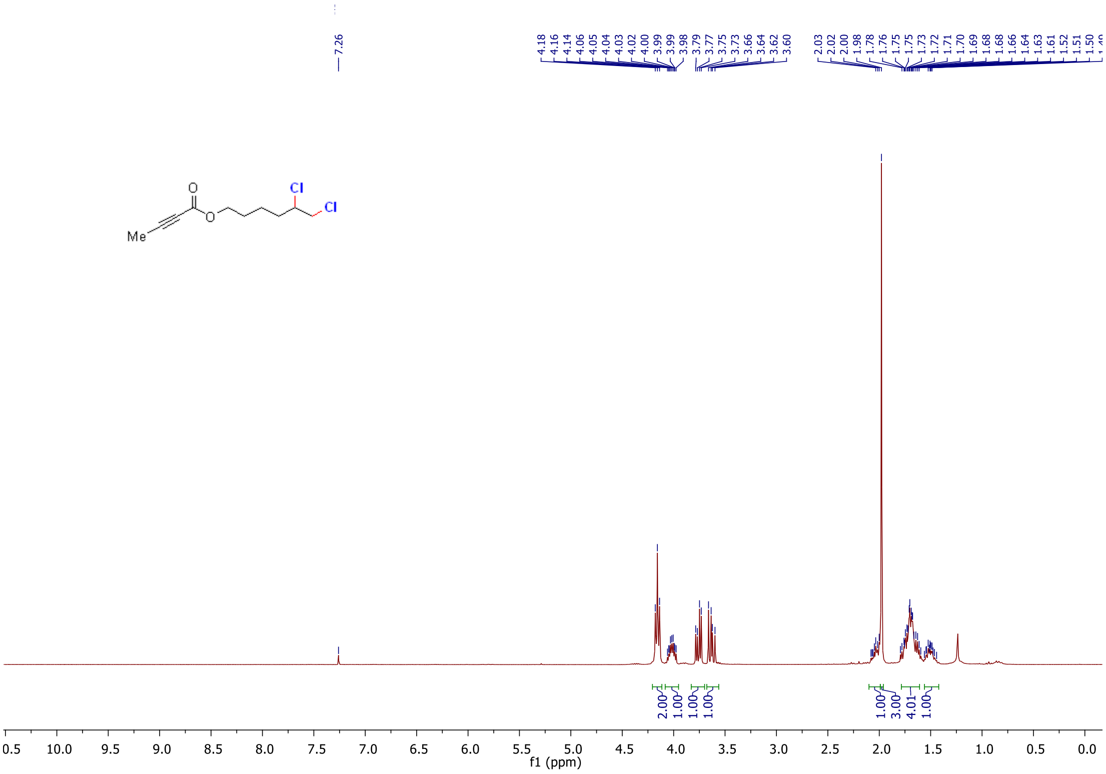

^{1}H NMR (300 MHz, $CDCl_3$)

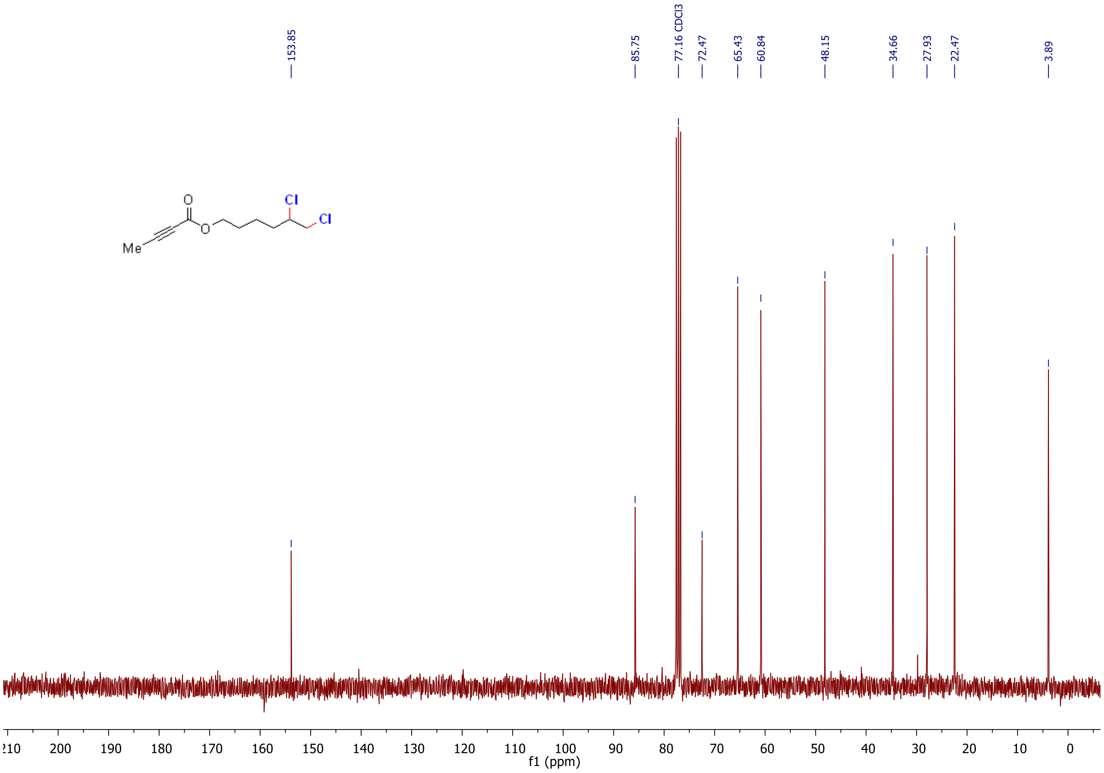

^{13}C NMR (75 MHz, $CDCl_3$)

((4,5-Dichloropentyl)sulfonyl)benzene (16)

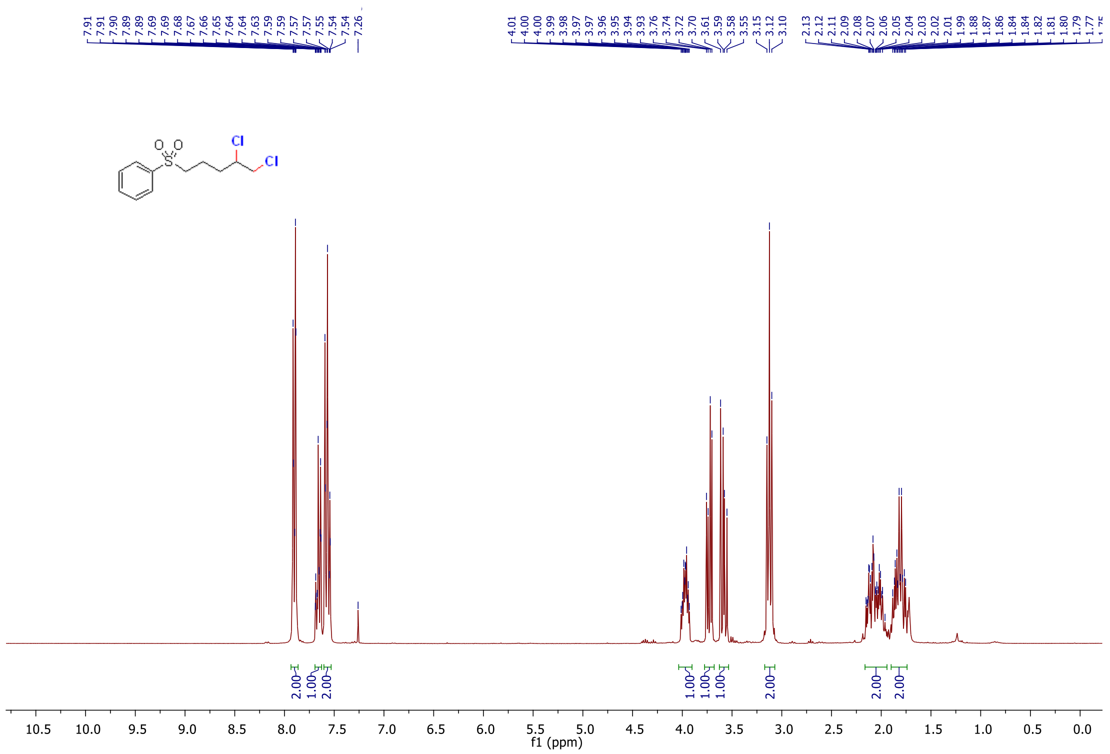

^{1}H NMR (300 MHz, $CDCl_3$)

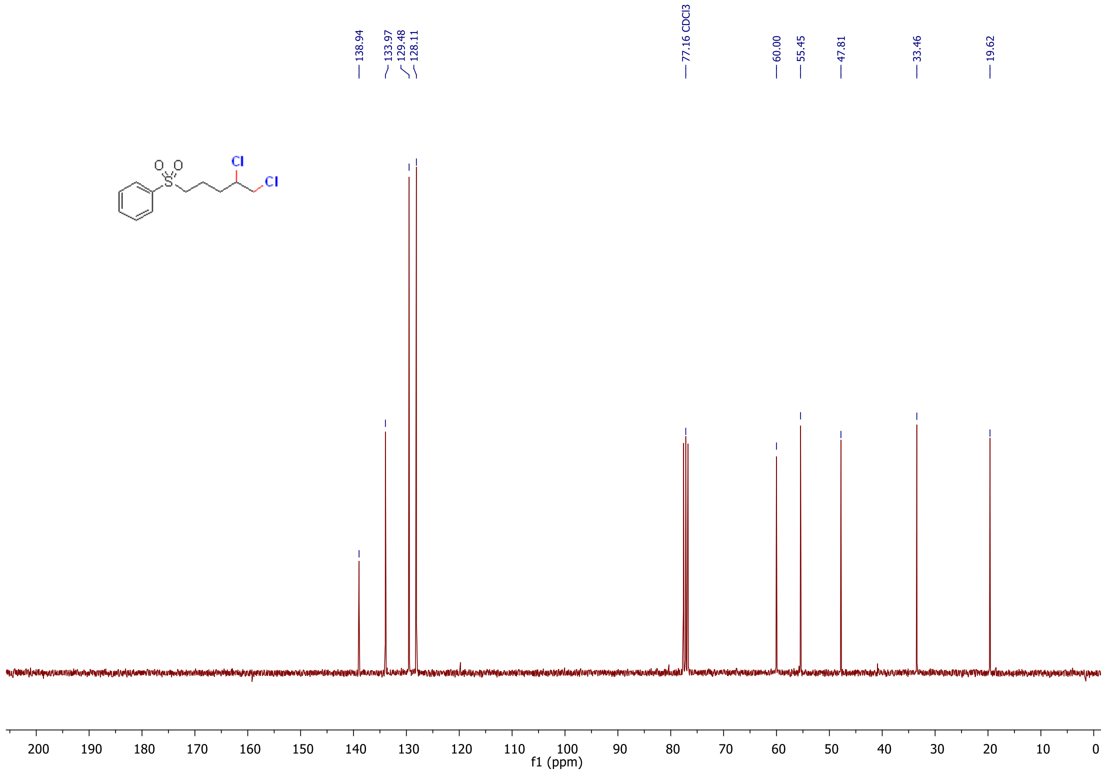

^{13}C NMR (75 MHz, $CDCl_3$)

5,6-Dichlorohexyl diphenyl phosphate (17)

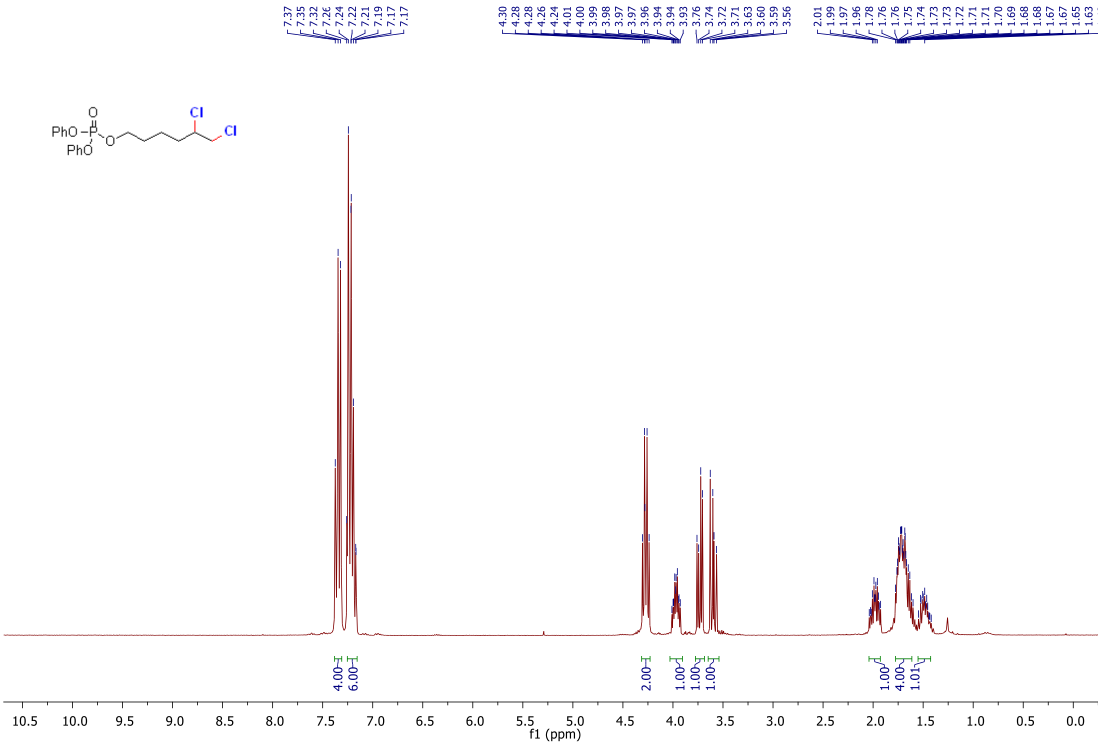

^{1}H NMR (300 MHz, $CDCl_3$)

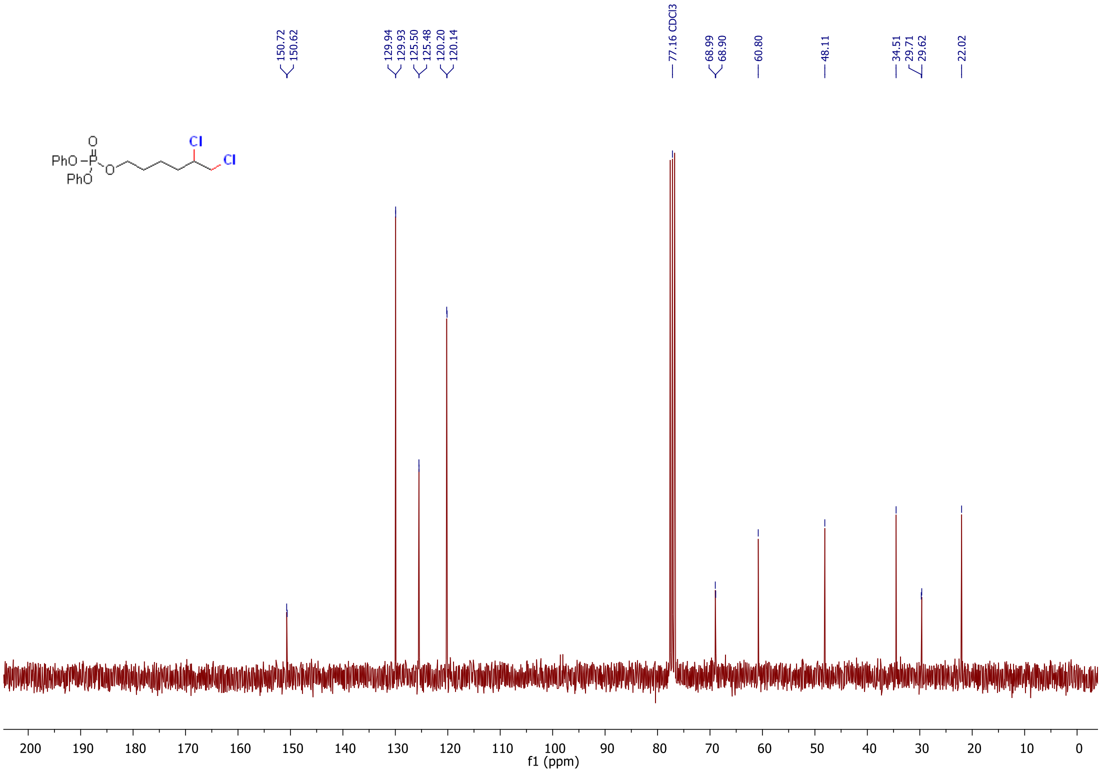

^{13}C NMR (75 MHz, $CDCl_3$)

3,4-Dichlorobutyl pyrazine-2-carboxylate (18)

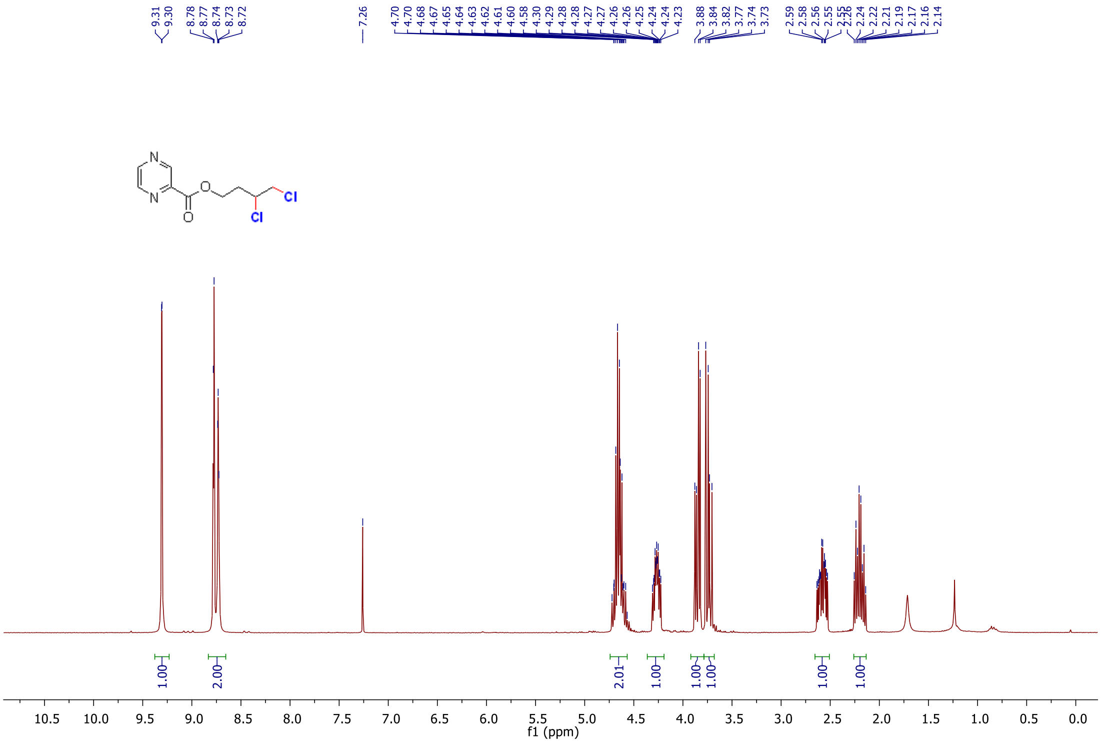

^{1}H NMR (300 MHz, $CDCl_3$)

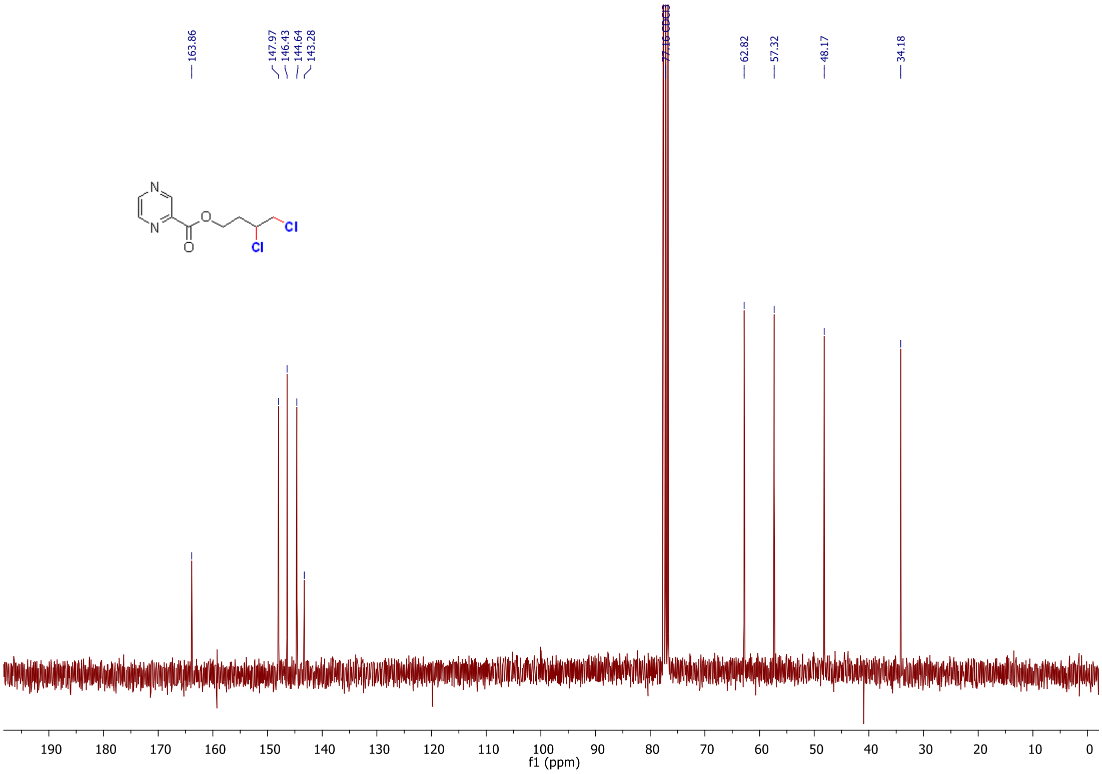

^{13}C NMR (75 MHz, $CDCl_3$)

2-((2,3-Dichloropropoxy)methyl)pyridine (19)

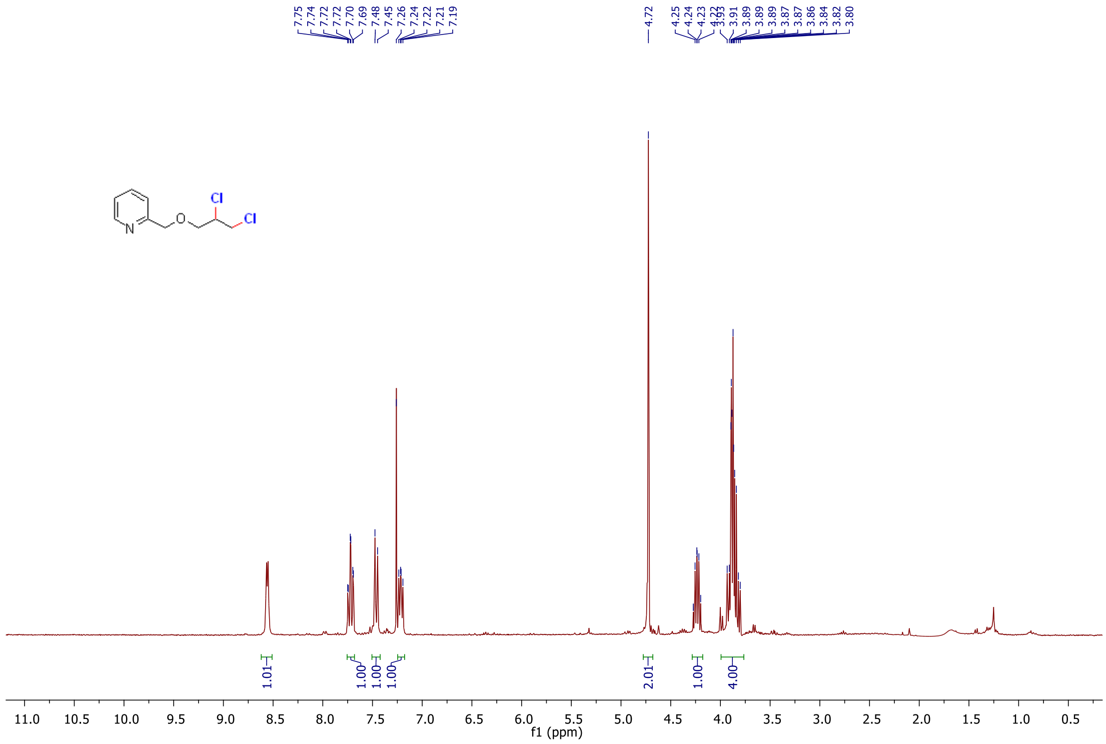

^{1}H NMR (300 MHz, $CDCl_3$)

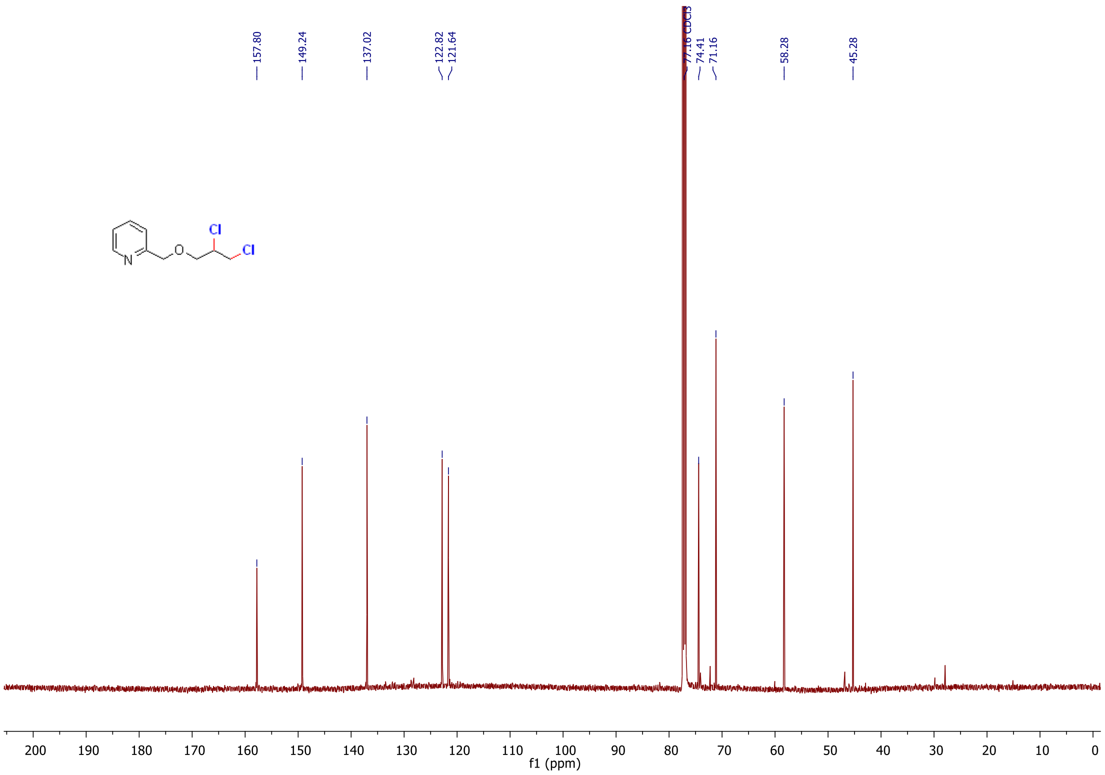

^{13}C NMR (126 MHz, $CDCl_3$)

3,4-Dichlorobutyl furan-2-carboxylate (20)

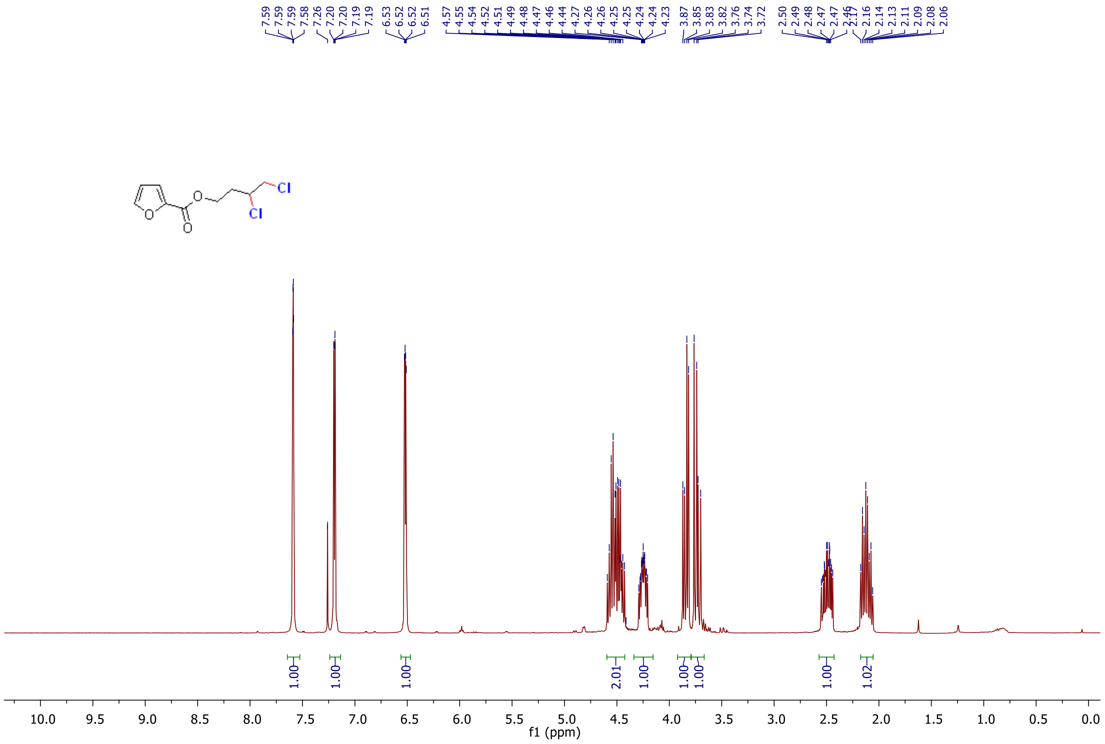

^{1}H NMR (300 MHz, $CDCl_3$)

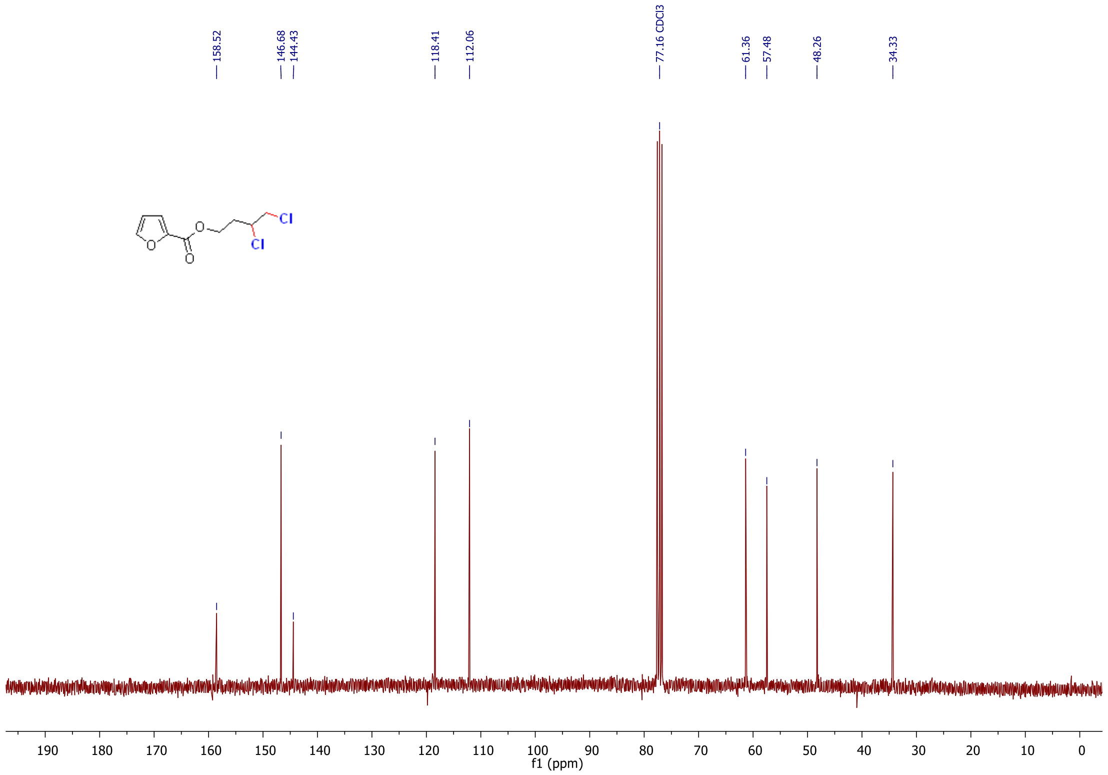

^{13}C NMR (75 MHz, $CDCl_3$)

(1*S*,2*R*,4*S*)-2-(2,3-Dichloropropoxy)-1,3,3-trimethylbicyclo[2.2.1]heptane (21)

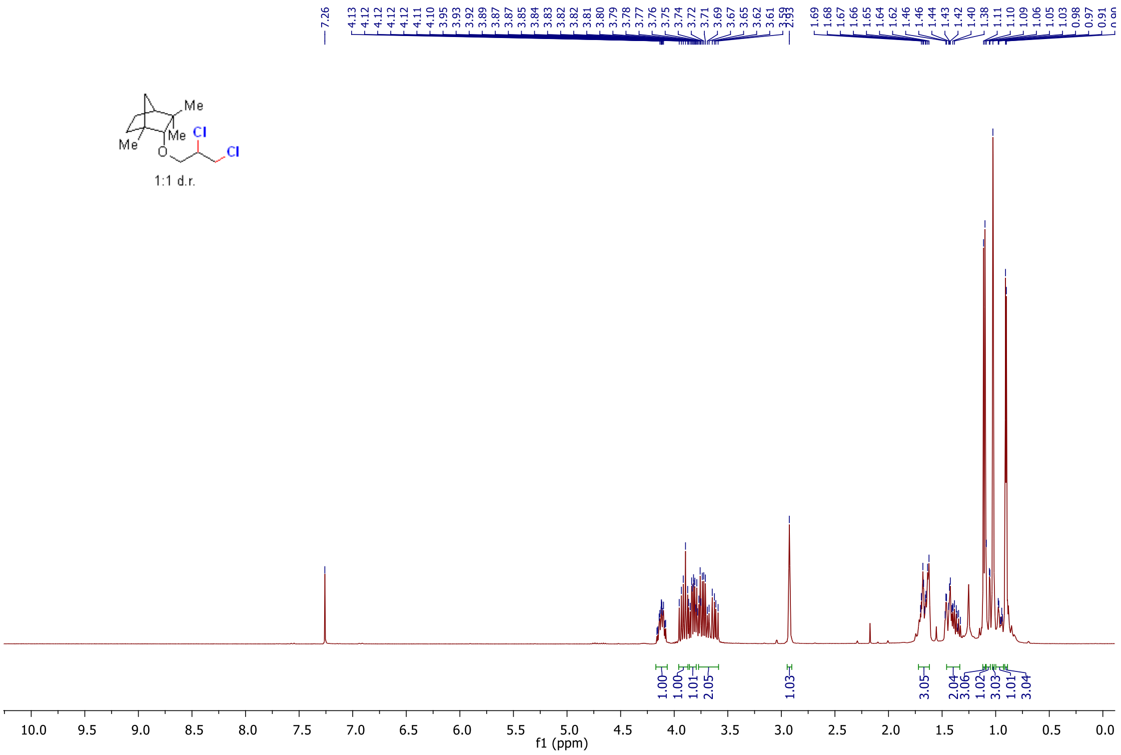

^{1}H NMR (300 MHz, $CDCl_3$)

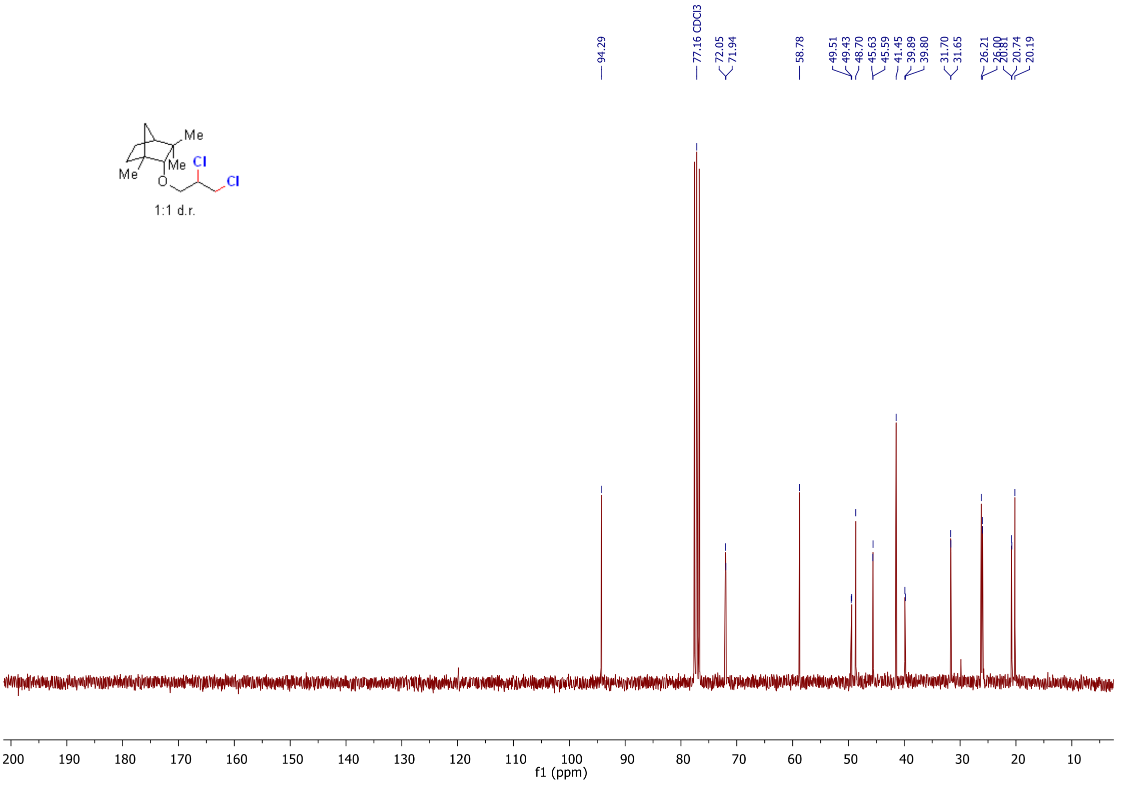

^{13}C NMR (75 MHz, $CDCl_3$)

Methyl-*anti*-2,3-dichloro-3-phenylpropanoate (22)

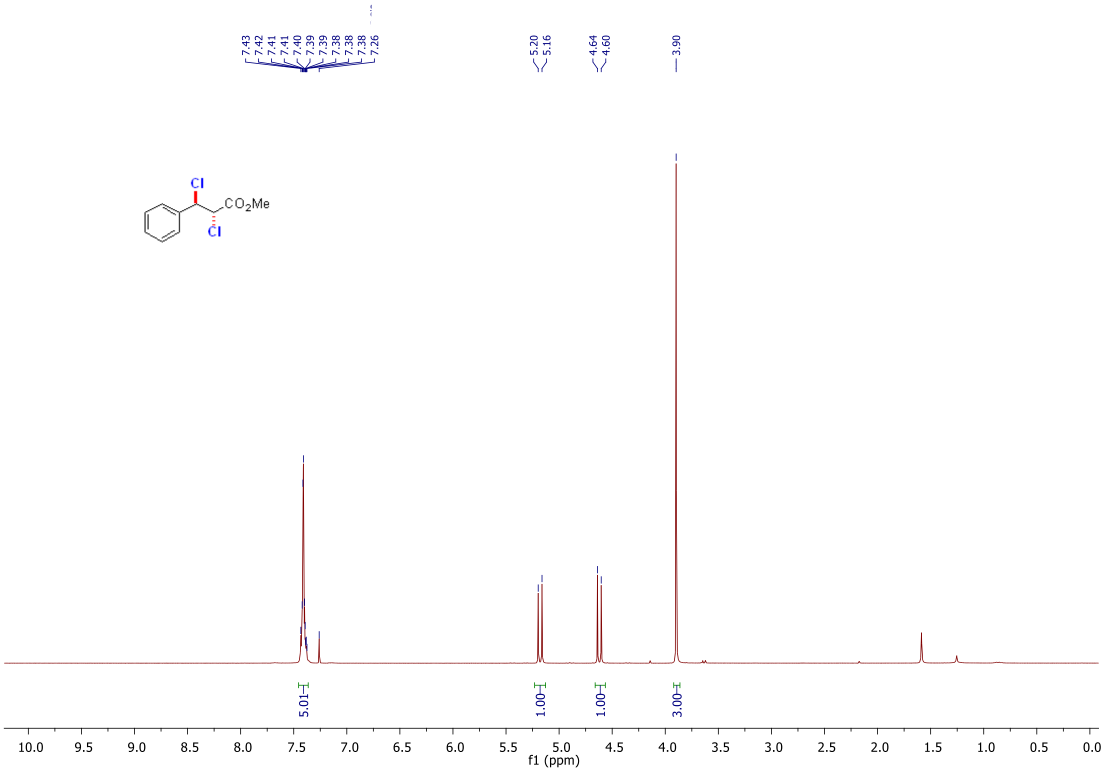

^1H NMR (300 MHz, $CDCl_3$)

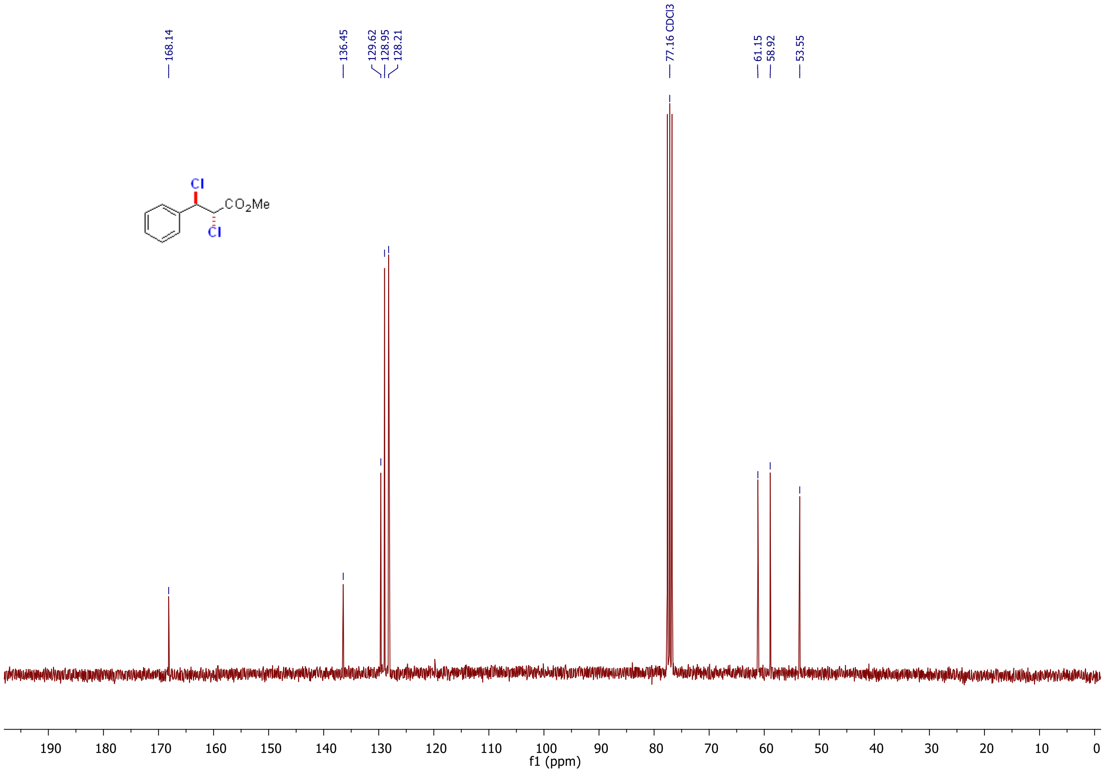

^{13}C NMR (75 MHz, $CDCl_3$)

anti-2,3-Dichloro-1,3-diphenylpropan-1-one (23)

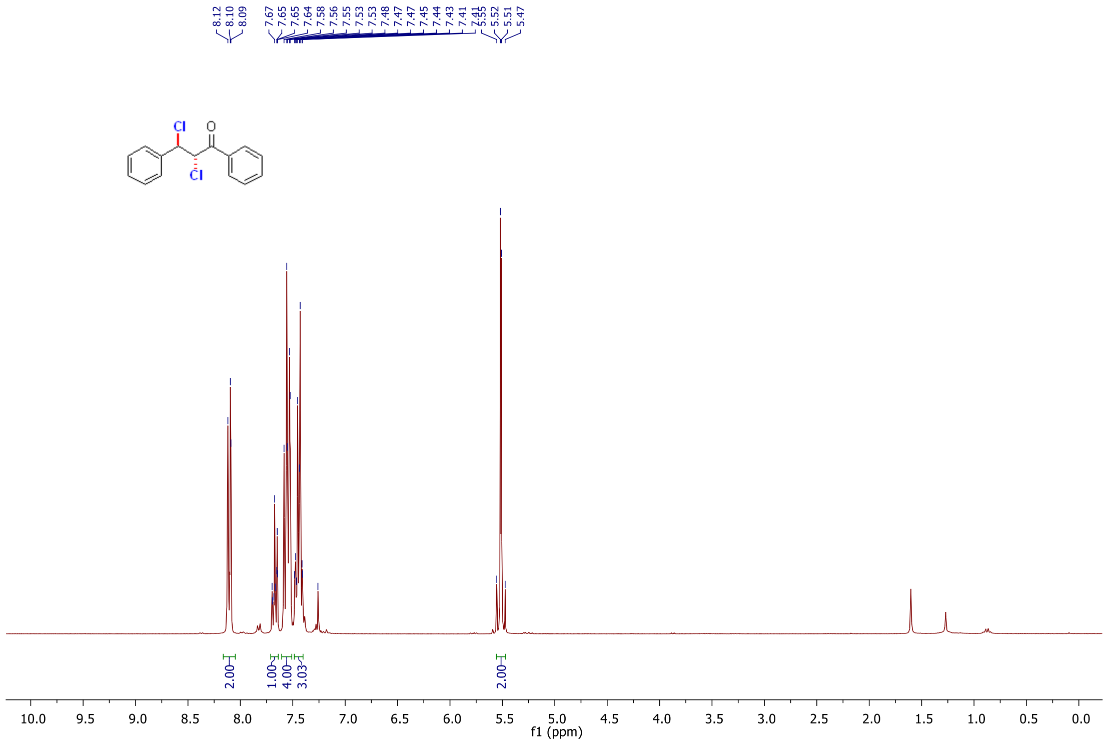

^{1}H NMR (300 MHz, CDCl$_3$)

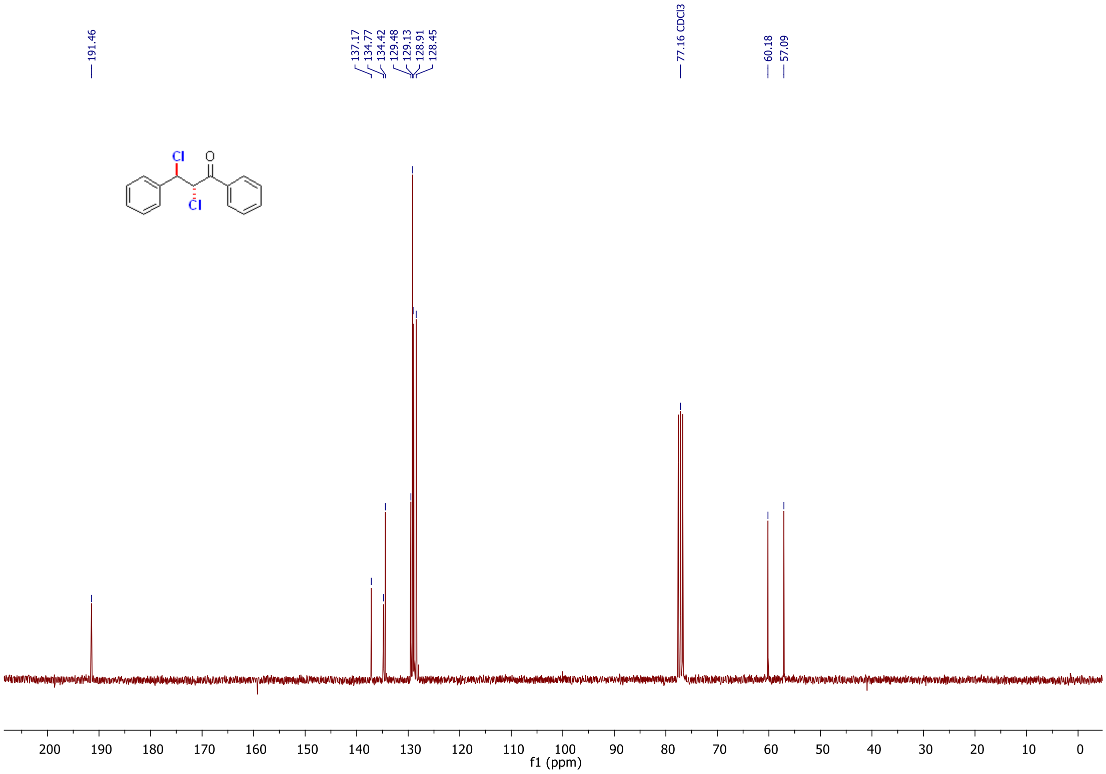

^{13}C NMR (75 MHz, CDCl$_3$)

anti-2,3-Dichloro-3-(4-fluorophenyl)-1-phenylpropan-1-one (24)

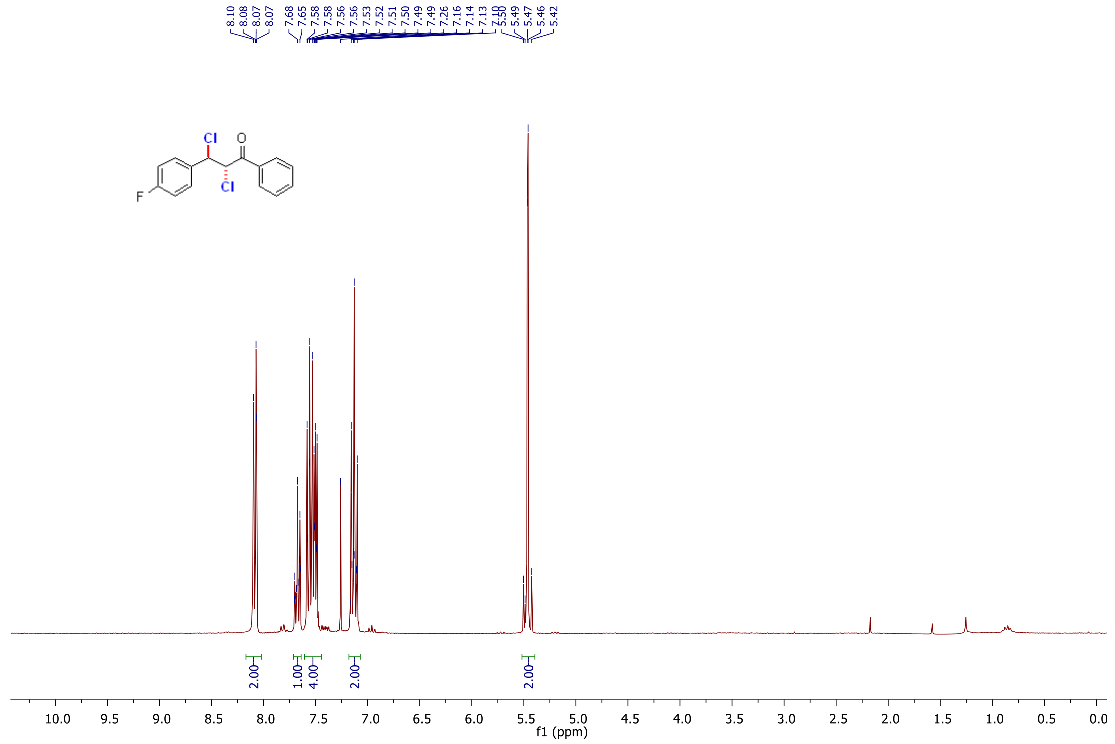

^{1}H NMR (300 MHz, $CDCl_3$)

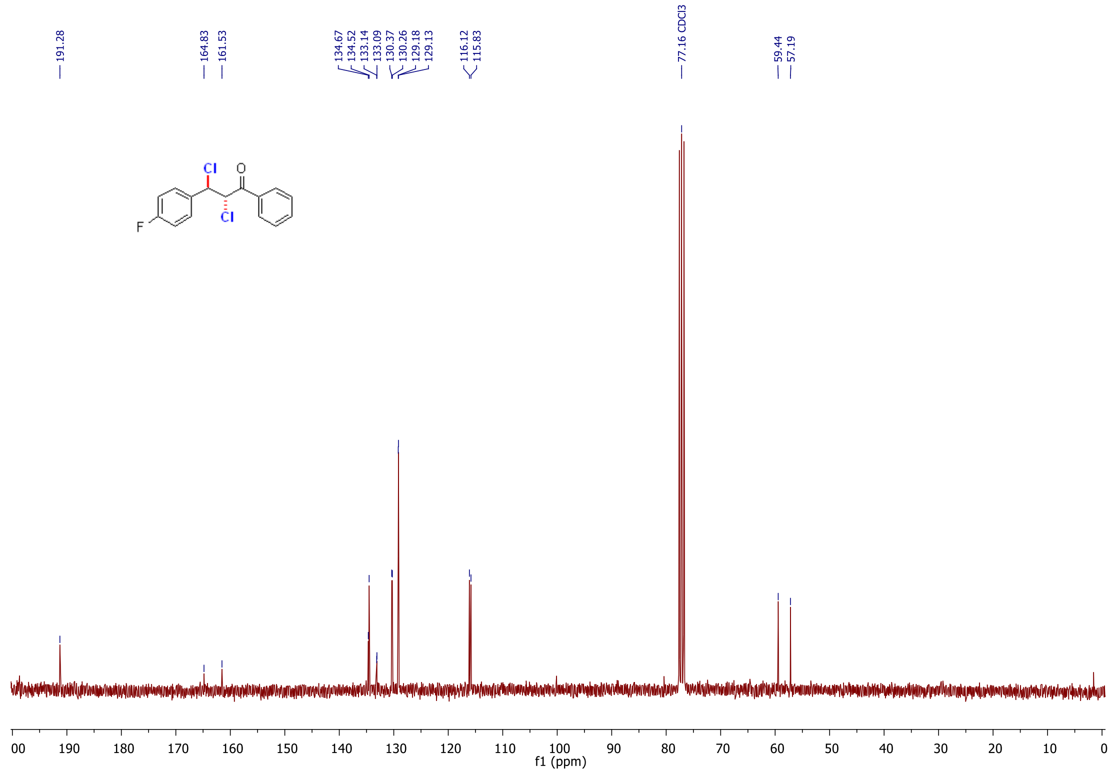

^{13}C NMR (75 MHz, $CDCl_3$)

1,2-Dichloro-1,2-diphenylethane (25)

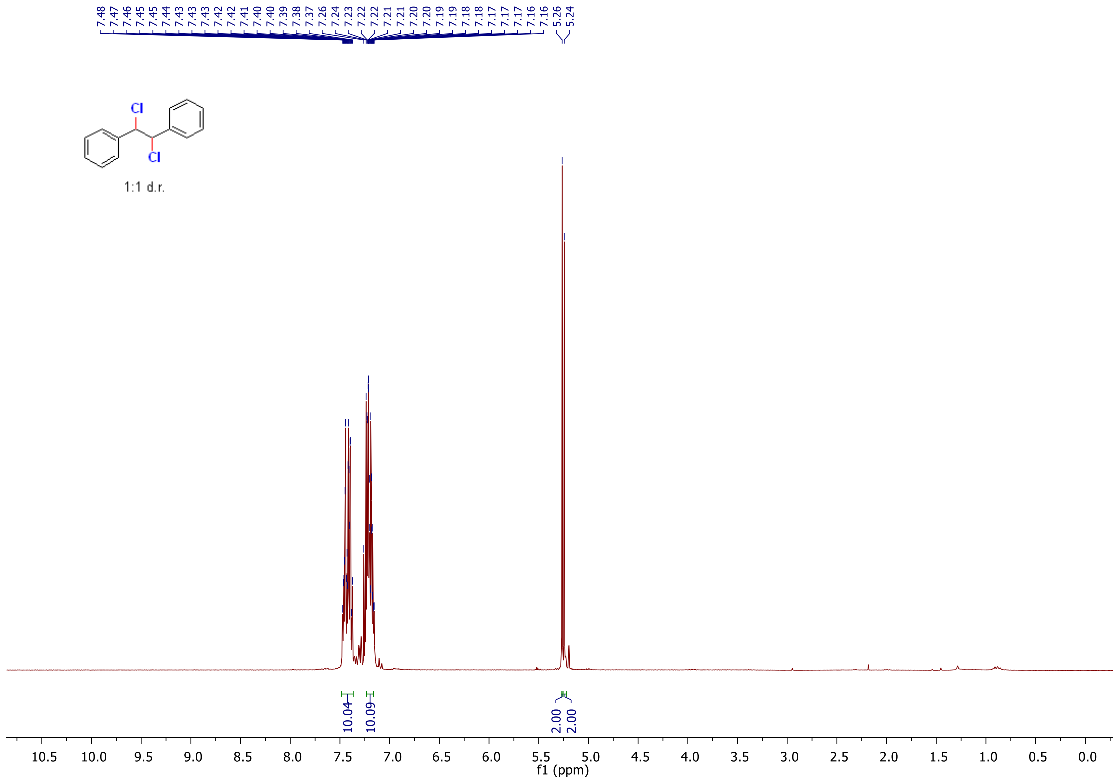

^{1}H NMR (300 MHz, $CDCl_3$)

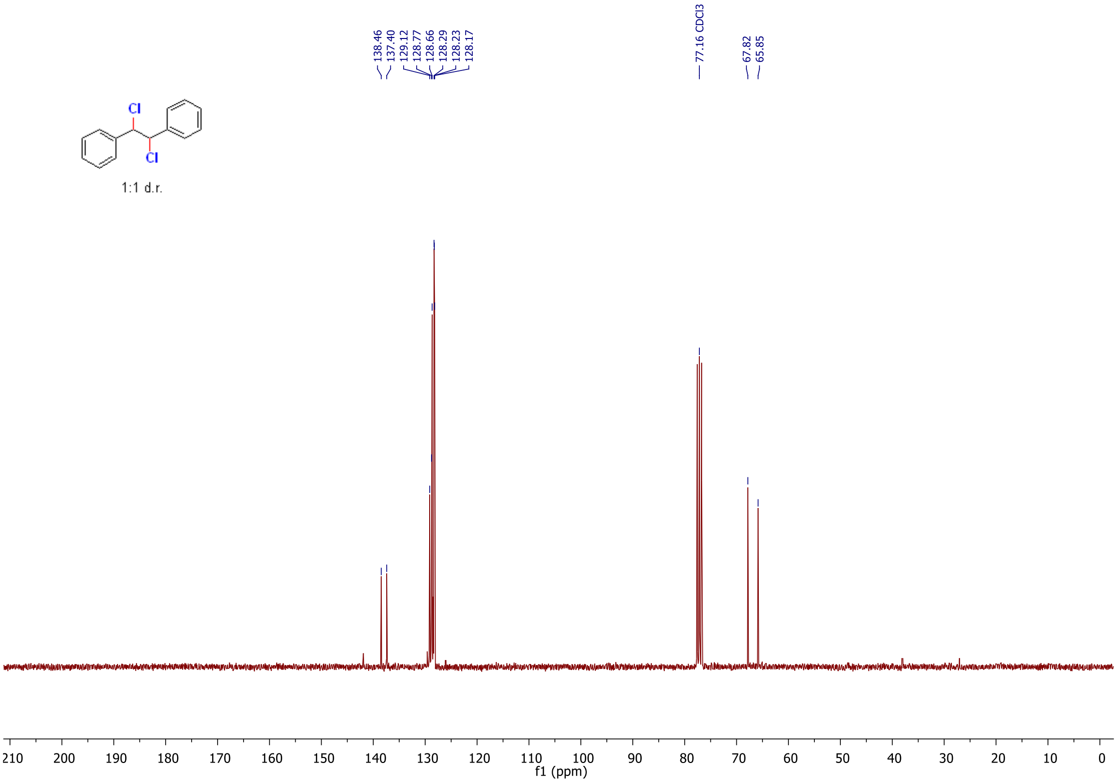

^{13}C NMR (75 MHz, $CDCl_3$)

4,5-Dichlorohexyl 4-methylbenzenesulfonate (26)

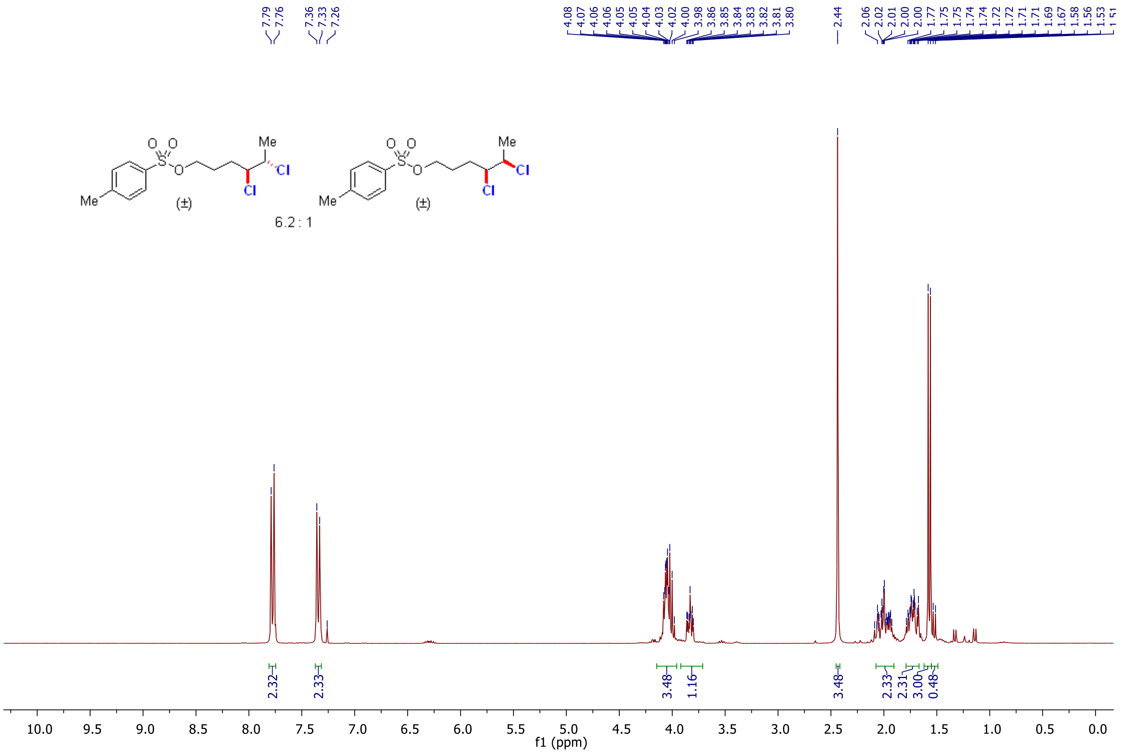

^{1}H NMR (300 MHz, $CDCl_3$)

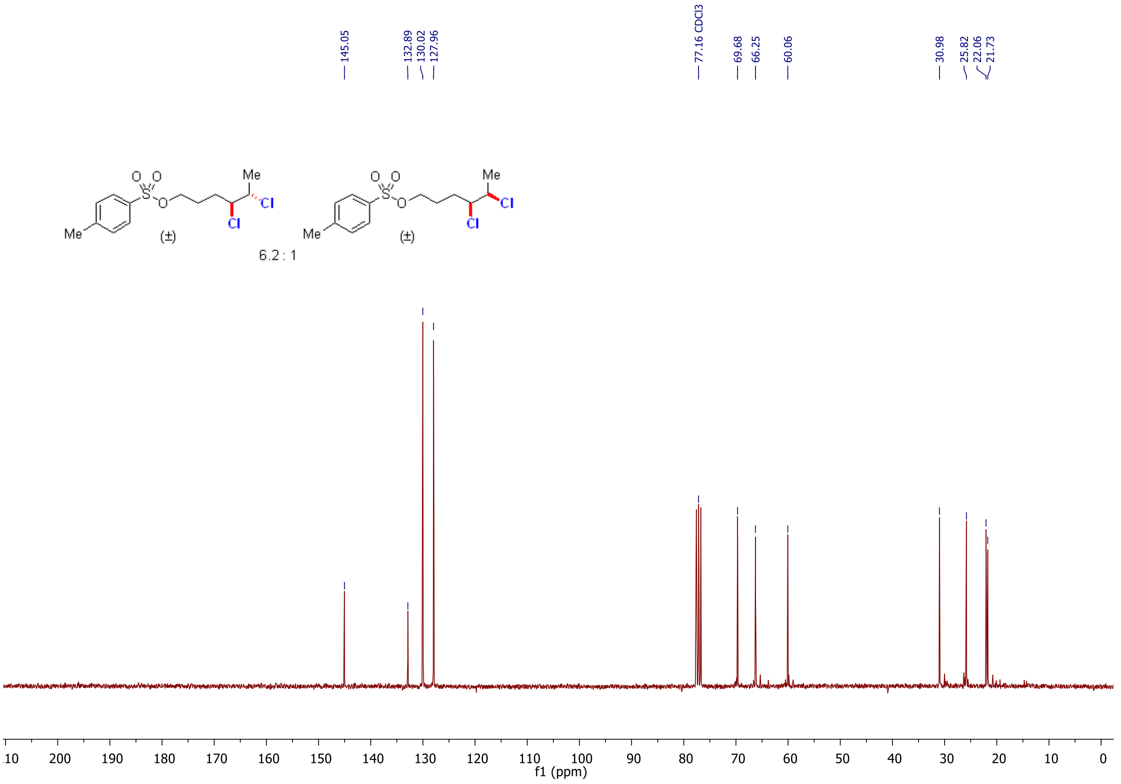

^{13}C NMR (75 MHz, $CDCl_3$)

4,5-Dichlorohexyl-4-methylbenzenesulfonate (27)

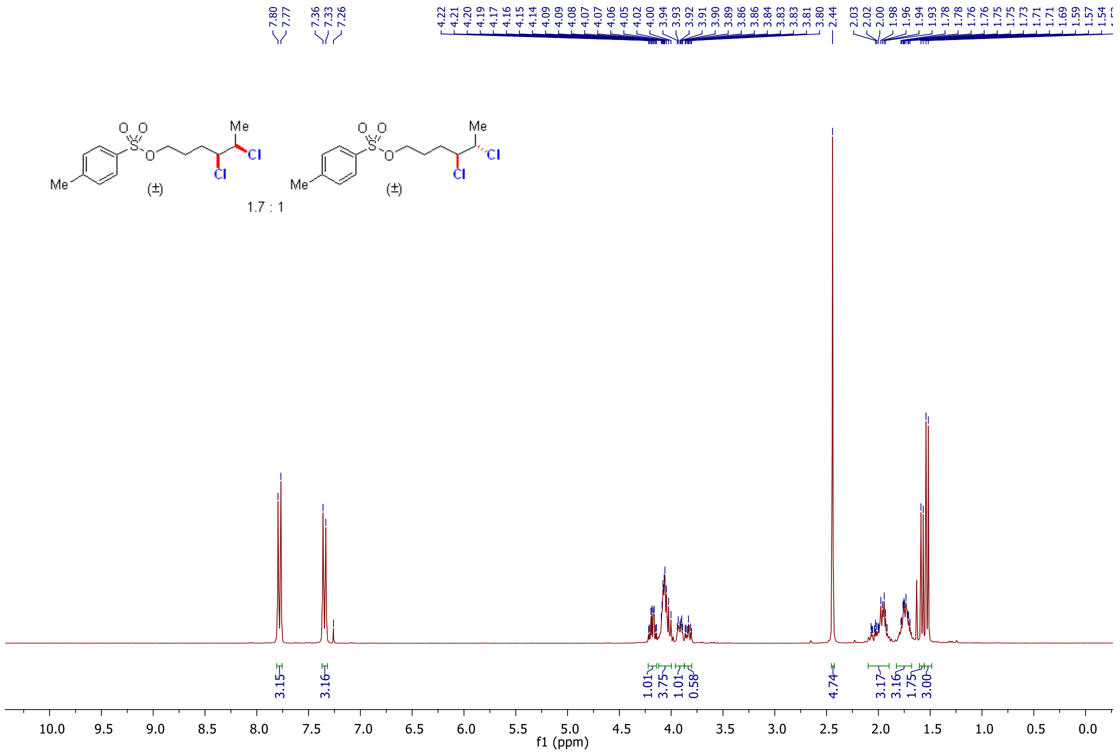

^{1}H NMR (300 MHz, $CDCl_3$)

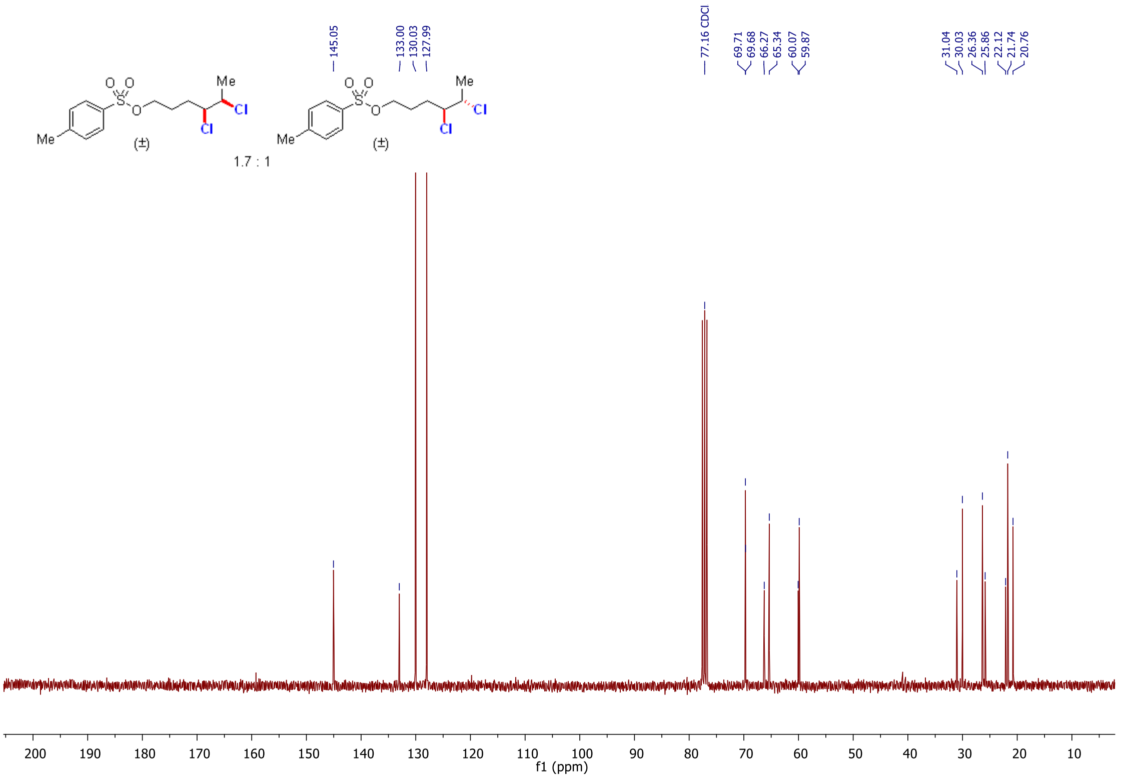

^{13}C NMR (75 MHz, $CDCl_3$)

4,5-Dichlorohexyl 4-chlorobenzoate (28)

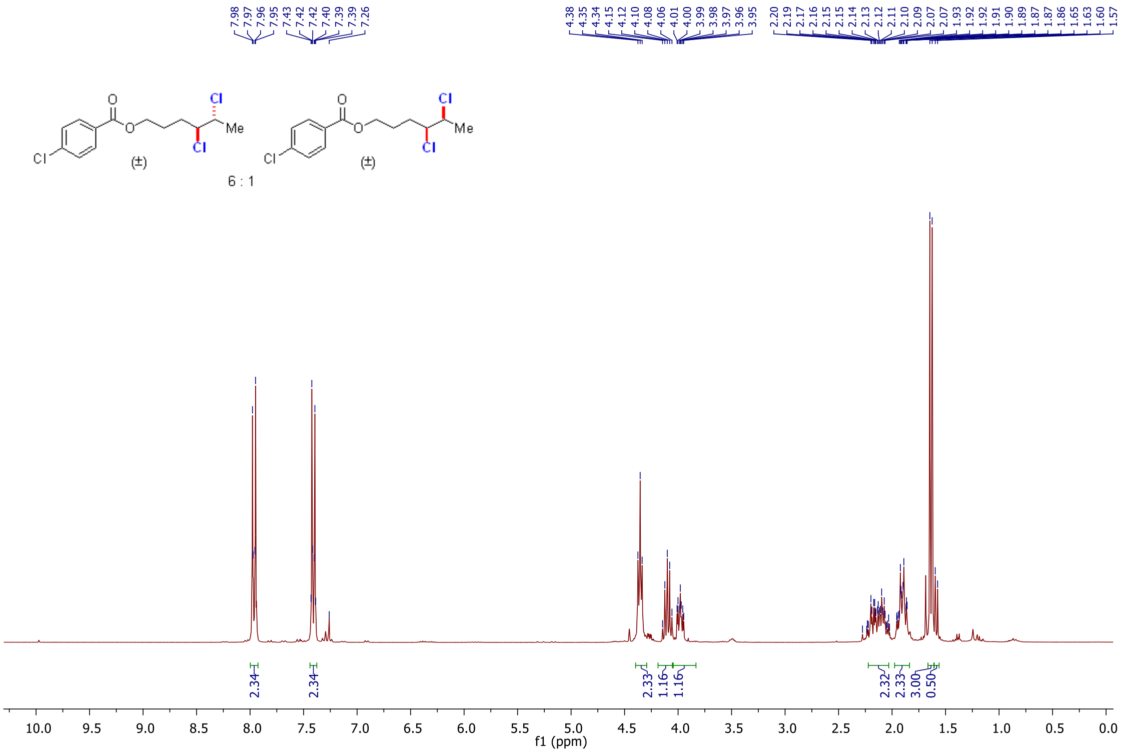

^1H NMR (300 MHz, $CDCl_3$)

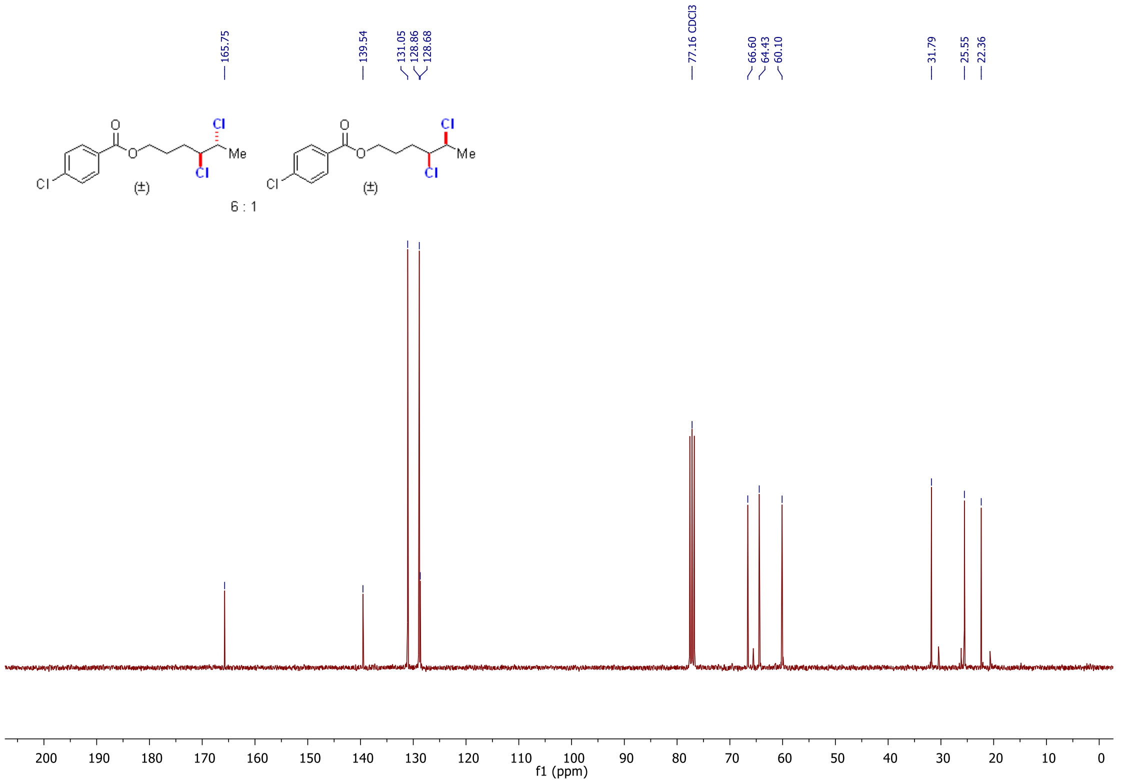

^{13}C NMR (75 MHz, $CDCl_3$)

4,5-Dichlorohexyl 4-chlorobenzoate (29)

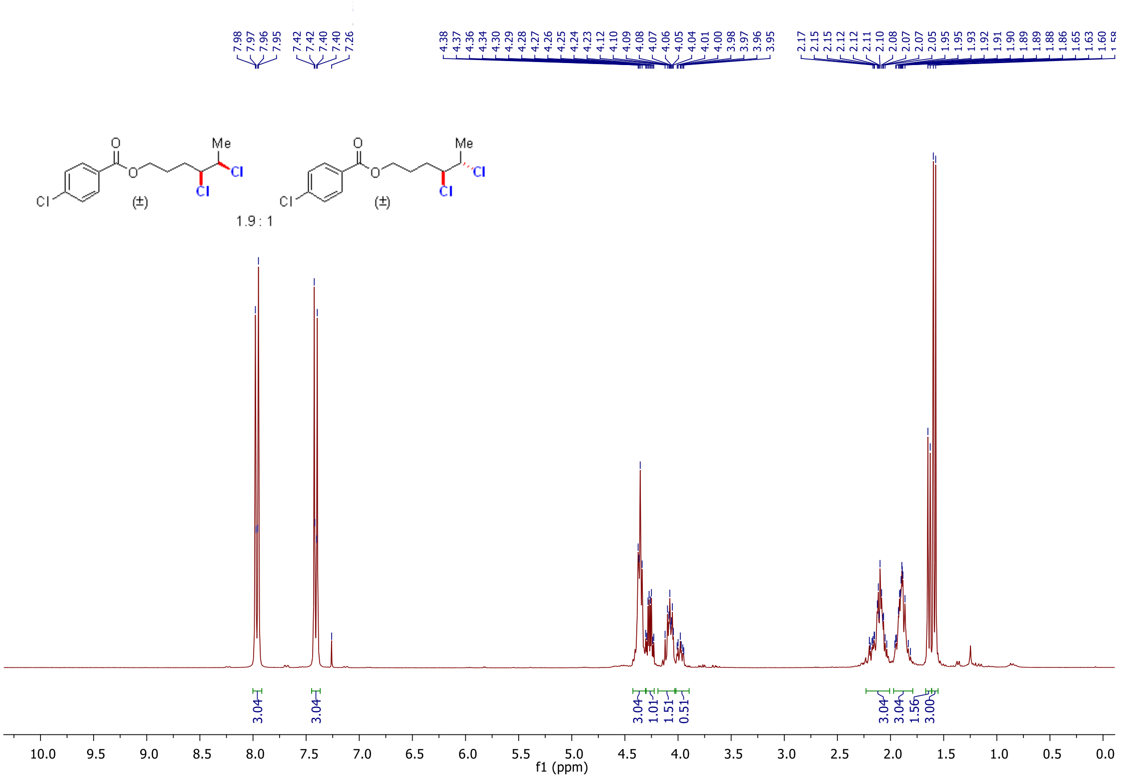

^{1}H NMR (300 MHz, $CDCl_3$)

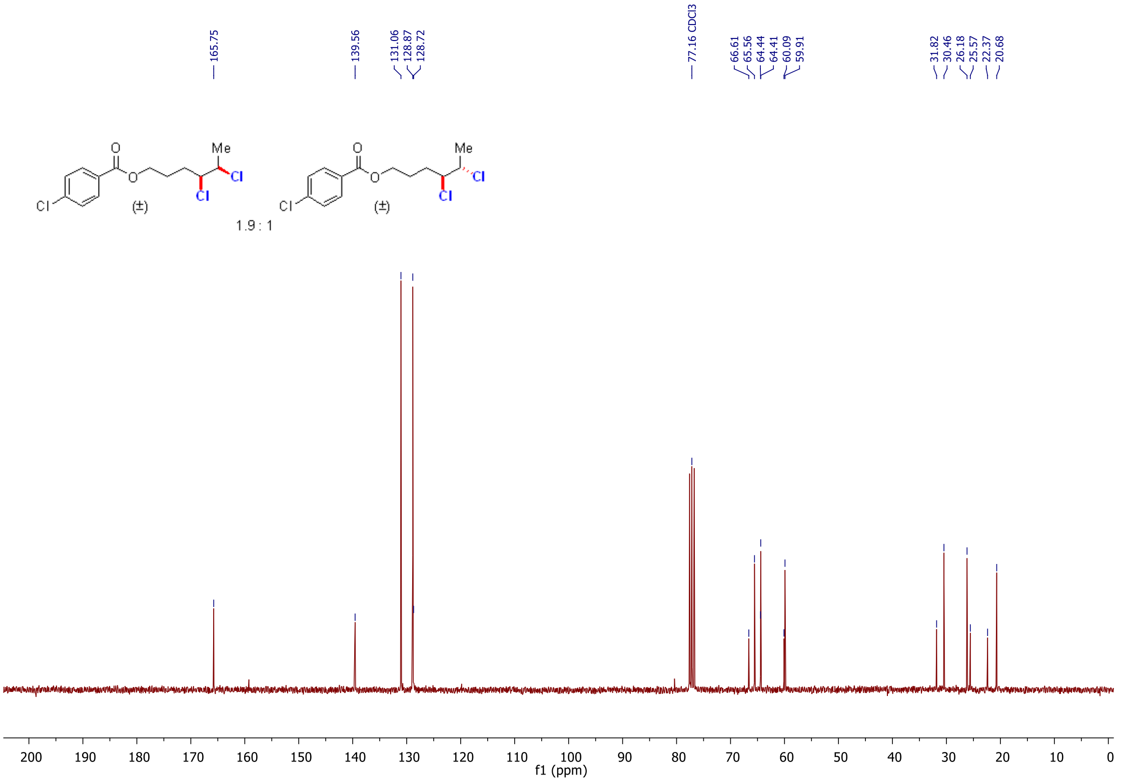

^{13}C NMR (75 MHz, $CDCl_3$)

2,3-Dichloro-2,3-diphenylpropanenitrile (30)

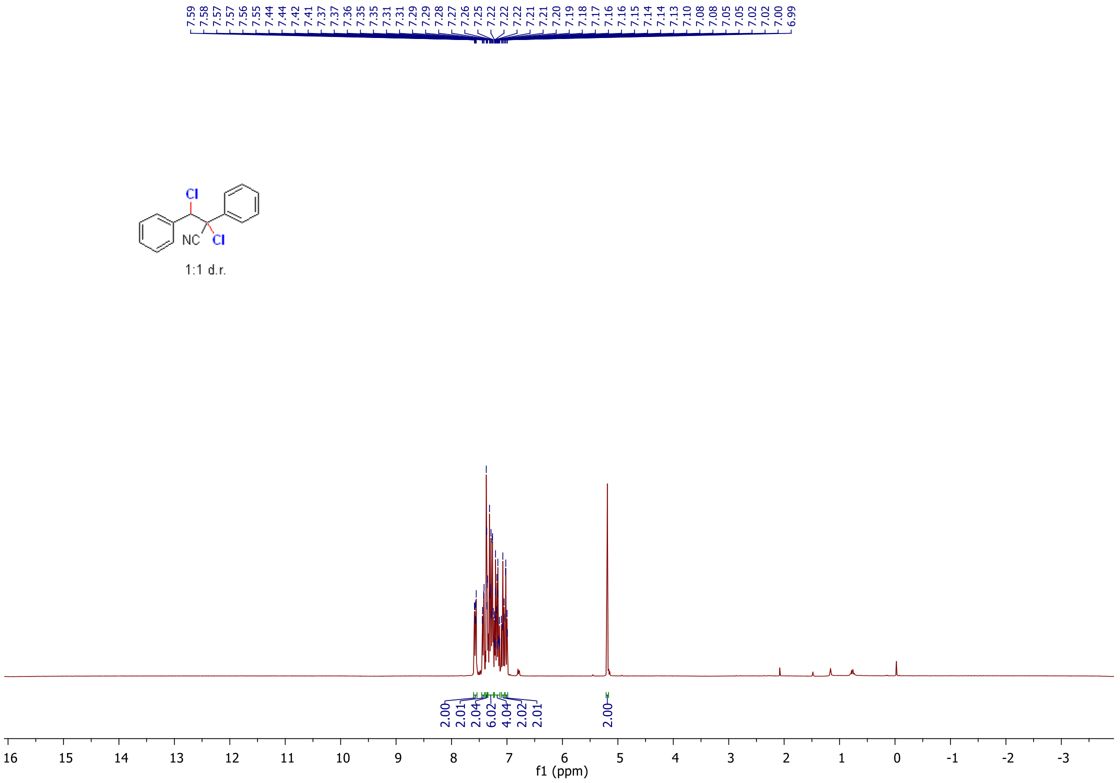

^{1}H NMR (300 MHz, $CDCl_3$)

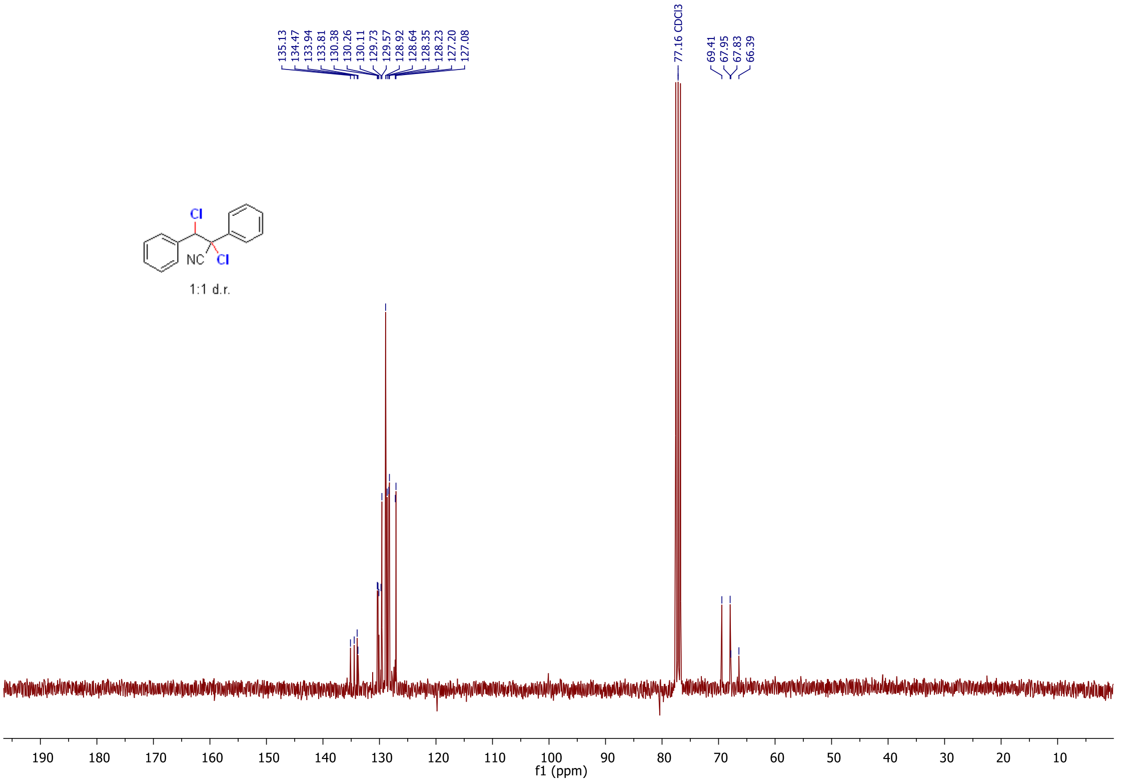

^{13}C NMR (75 MHz, $CDCl_3$)

(1,2-Dichloropropane-1,2-diyl)dibenzene (31)

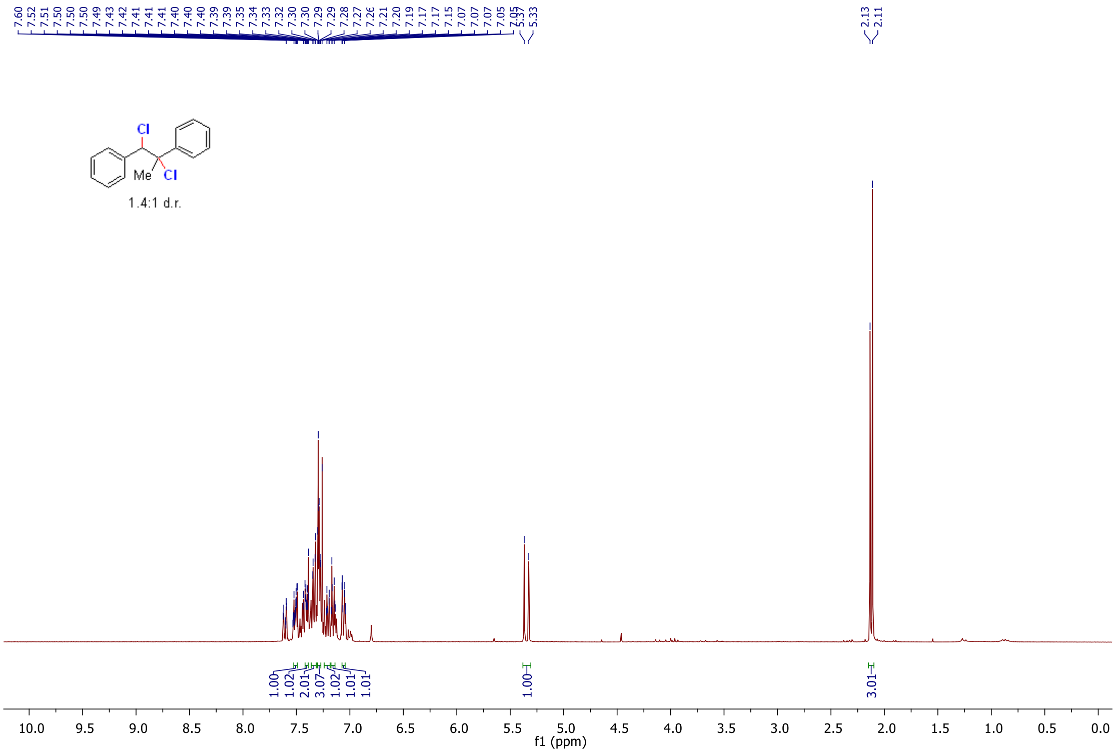

^{1}H NMR (300 MHz, $CDCl_3$)

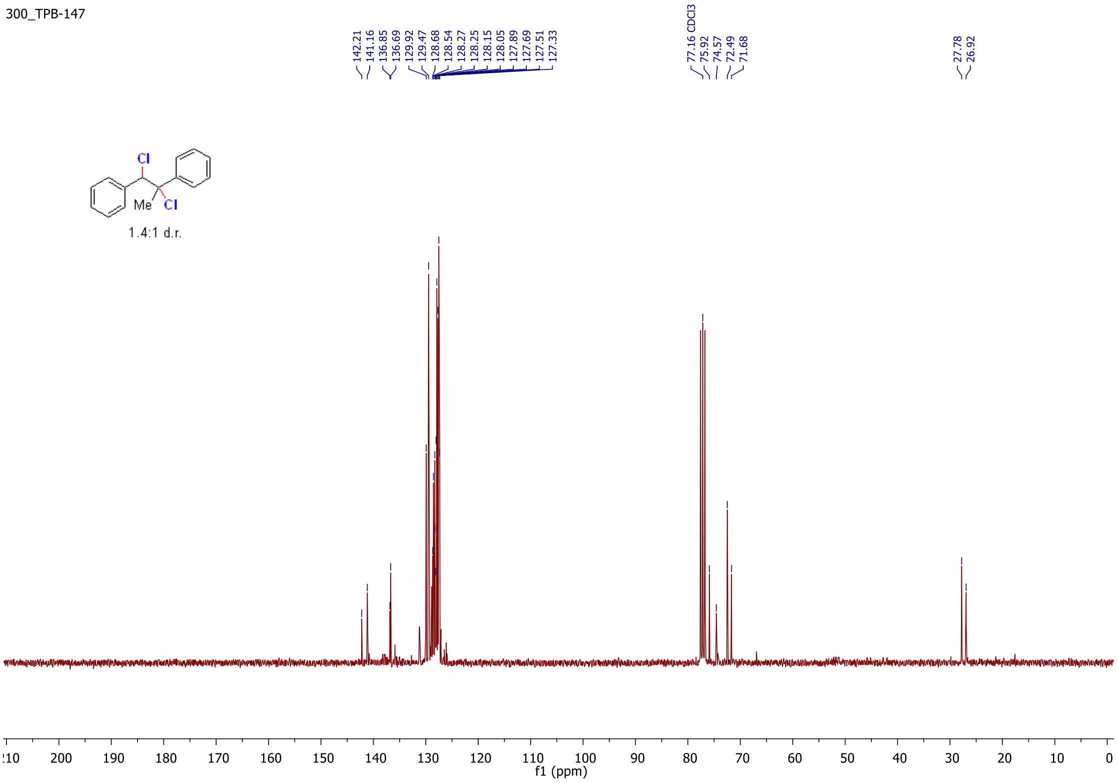

^{13}C NMR (75 MHz, $CDCl_3$)

(2-Chloroethene-1,1,2-triyl)tribenzene (32)

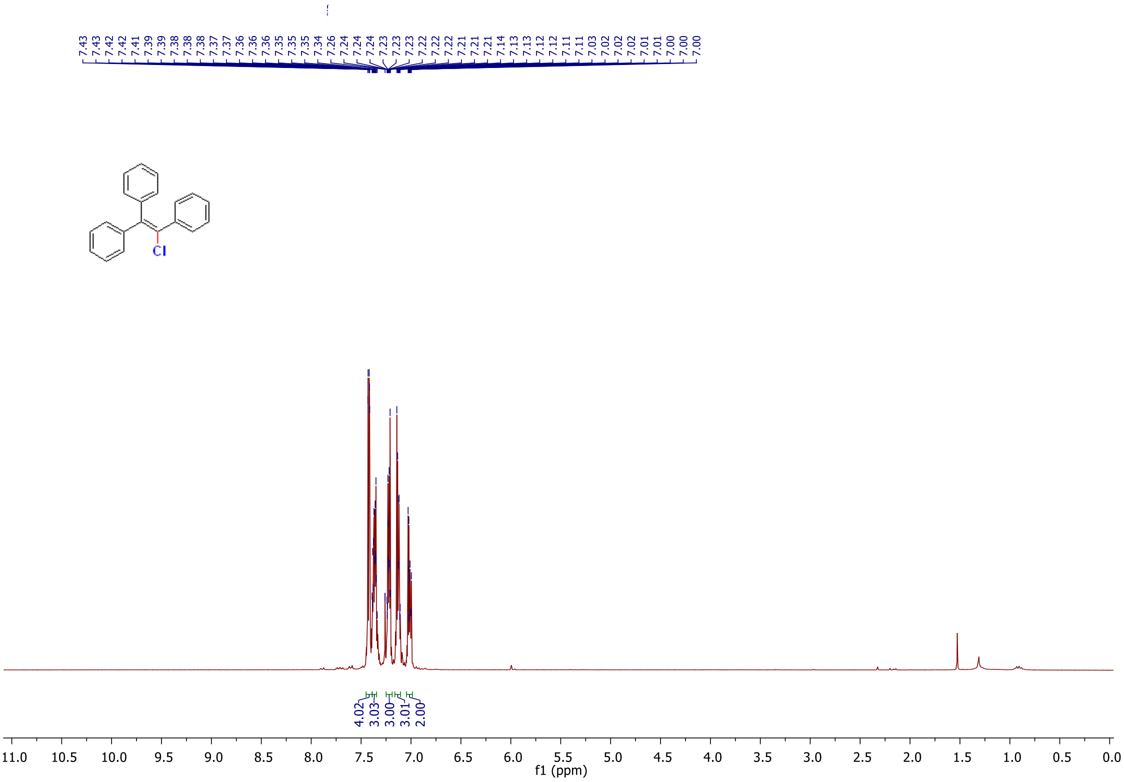

^1H NMR (300 MHz, $CDCl_3$)

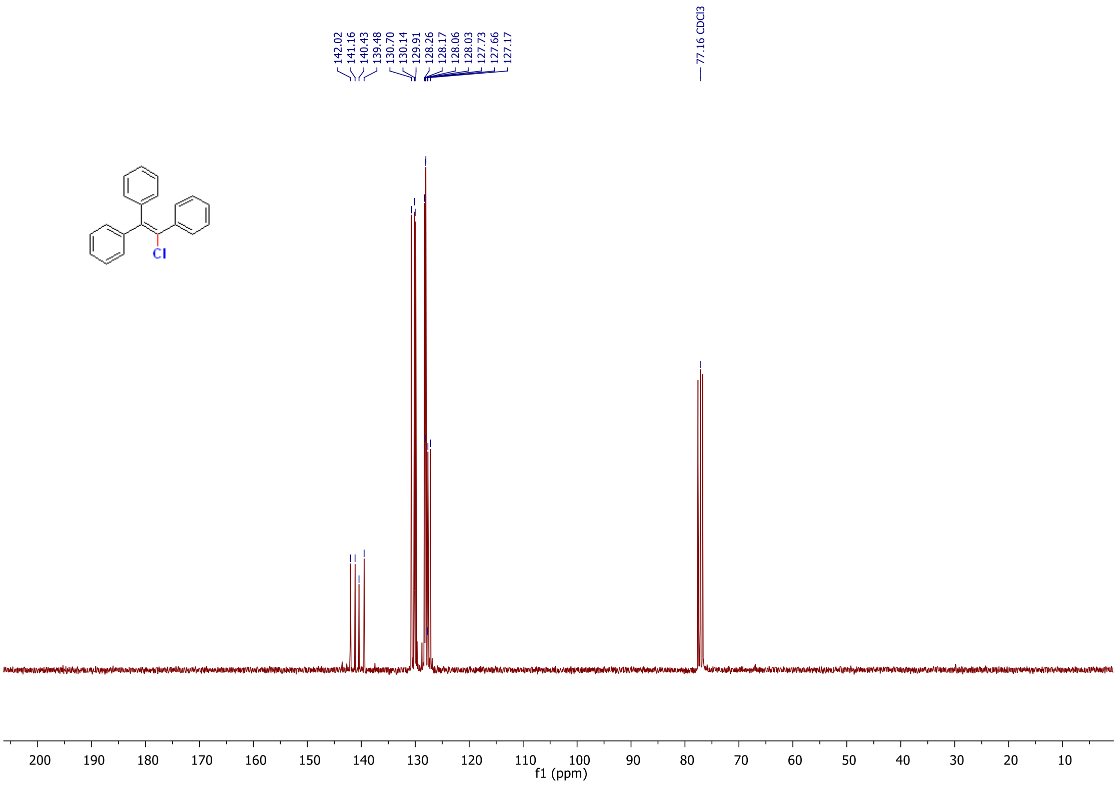

^{13}C NMR (75 MHz, $CDCl_3$)

5-(Chloromethyl)dihydrofuran-2(3*H*)-one (33)

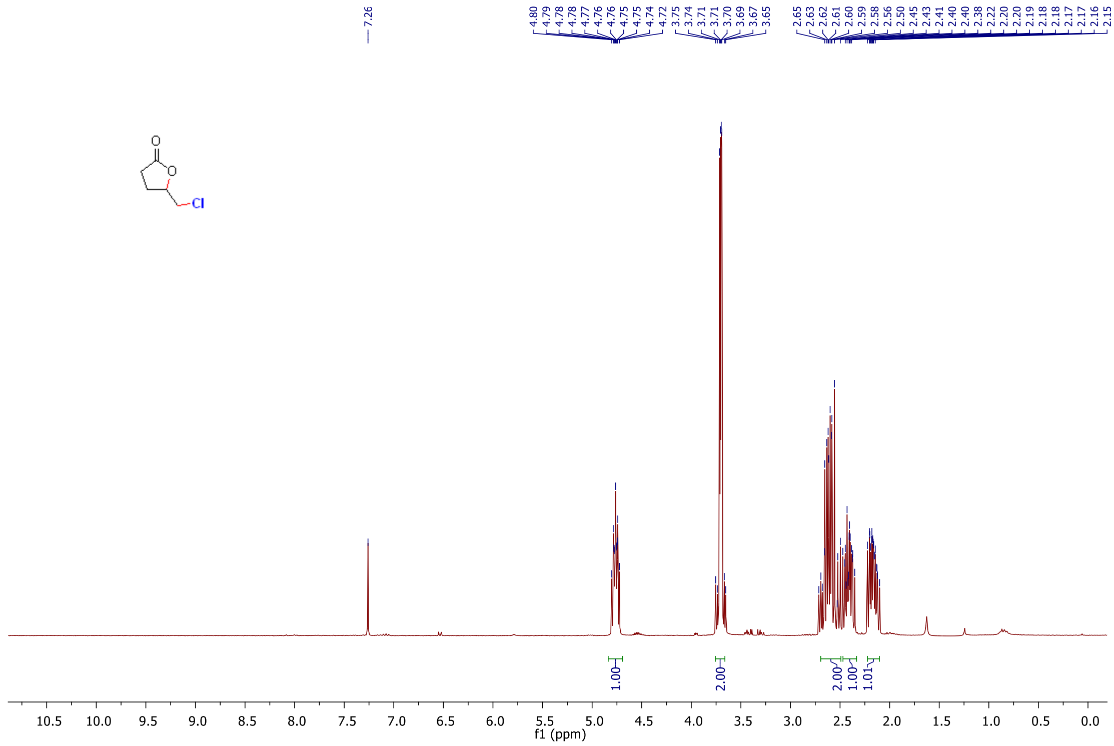

^1H NMR (300 MHz, $CDCl_3$)

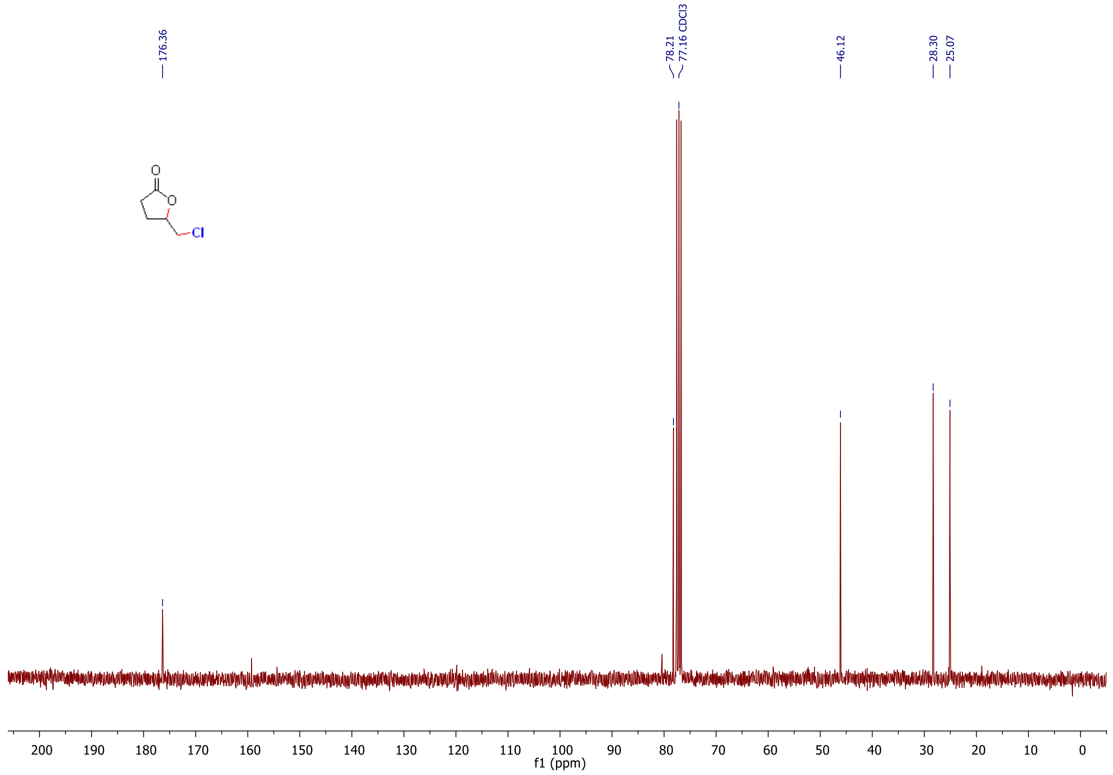

^{13}C NMR (75 MHz, $CDCl_3$)

5-(Chloromethyl)-3-phenyldihydrofuran-2(3*H*)-one (34)

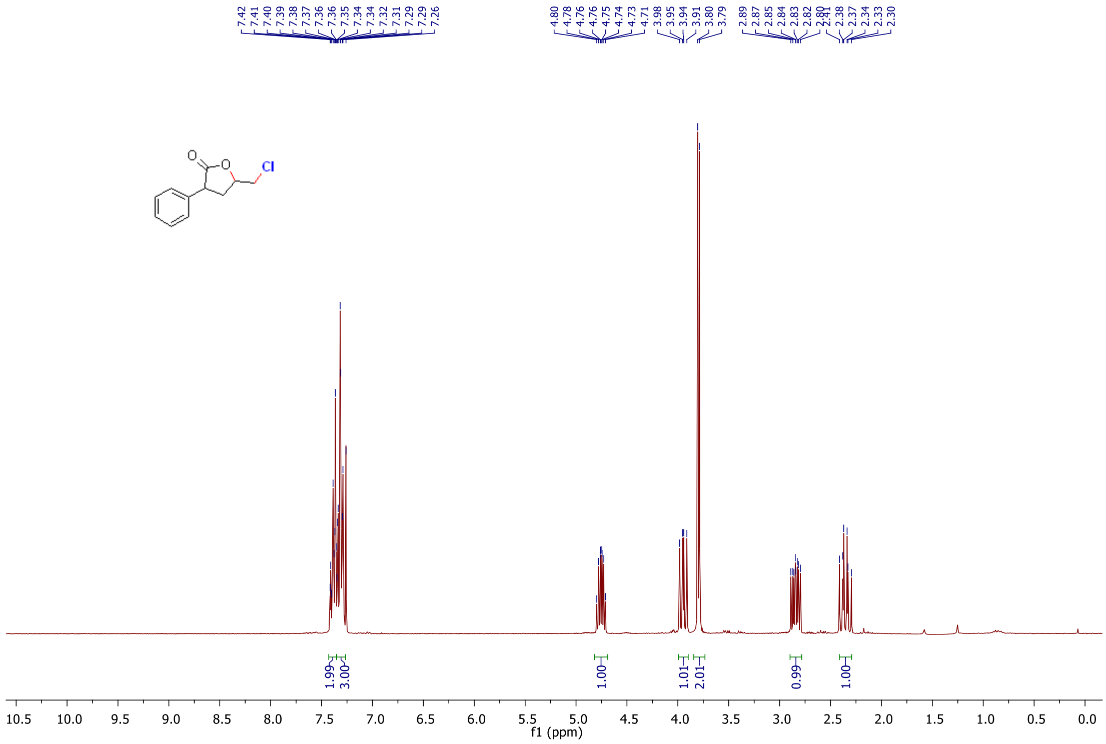

^{1}H NMR (300 MHz, $CDCl_3$)

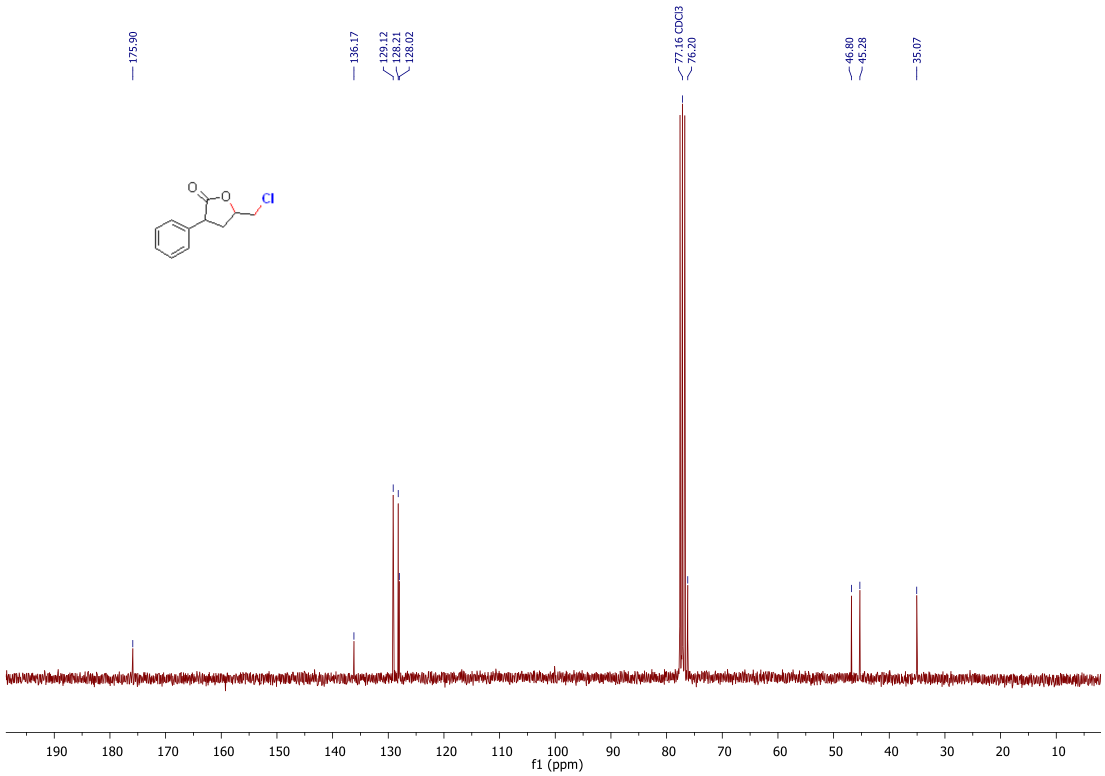

^{13}C NMR (75 MHz, $CDCl_3$)

2-Chloro-1,3-diphenylpropane-1,3-dione (35)

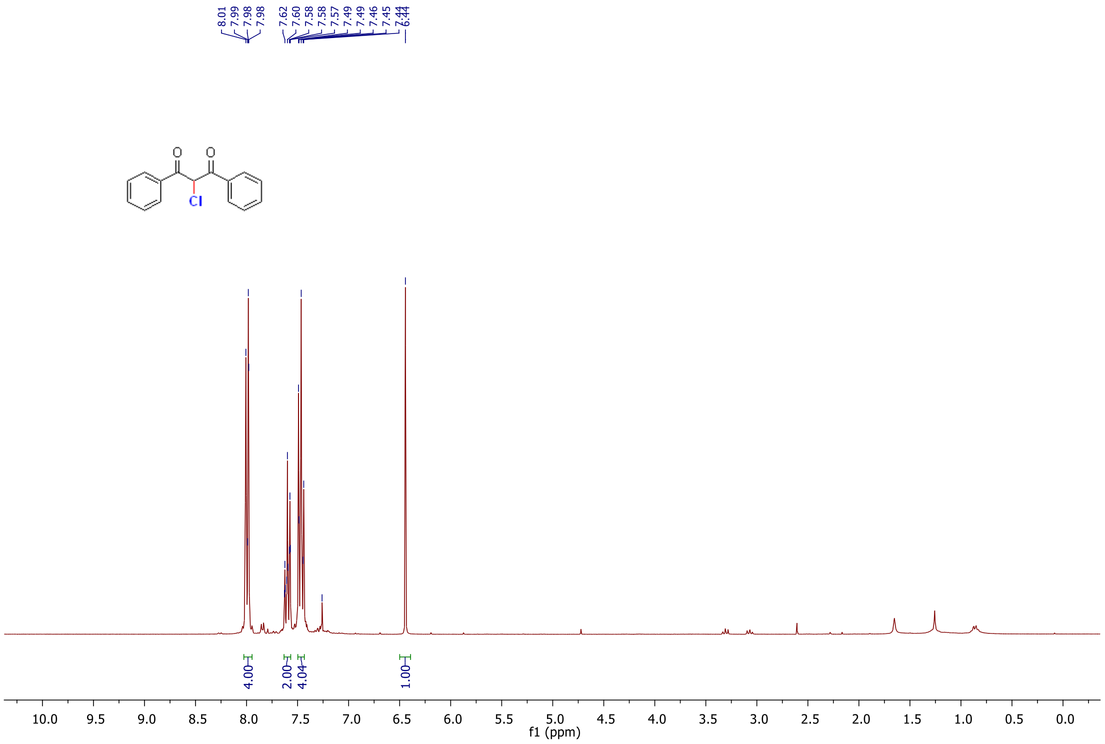

^{1}H NMR (300 MHz, $CDCl_3$)

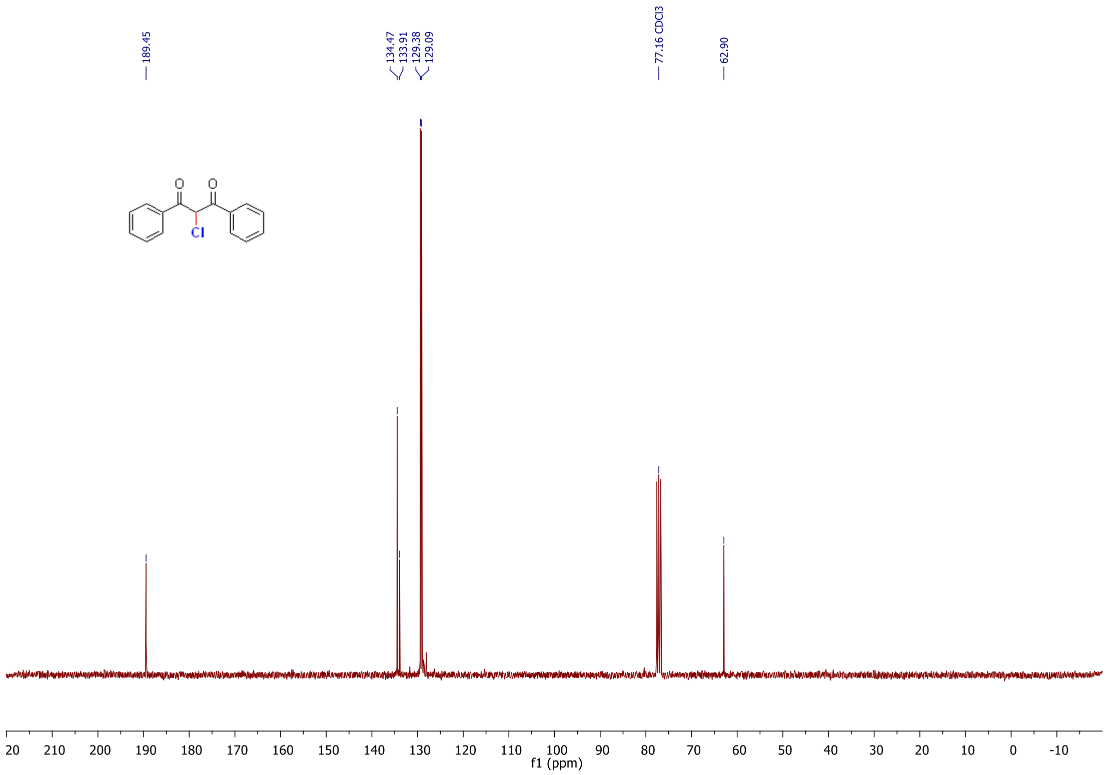

^{13}C NMR (75 MHz, $CDCl_3$)

2-Chloro-2-methyl-1,3-diphenylpropane-1,3-dione (36)

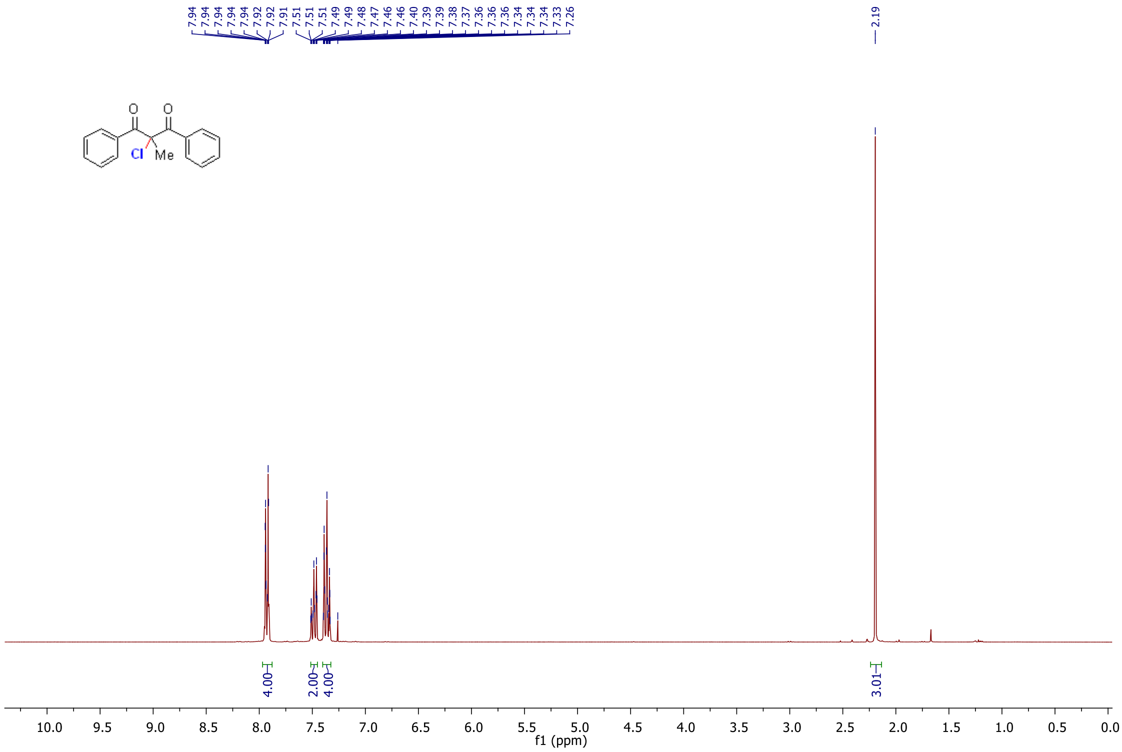

^{1}H NMR (300 MHz, $CDCl_3$)

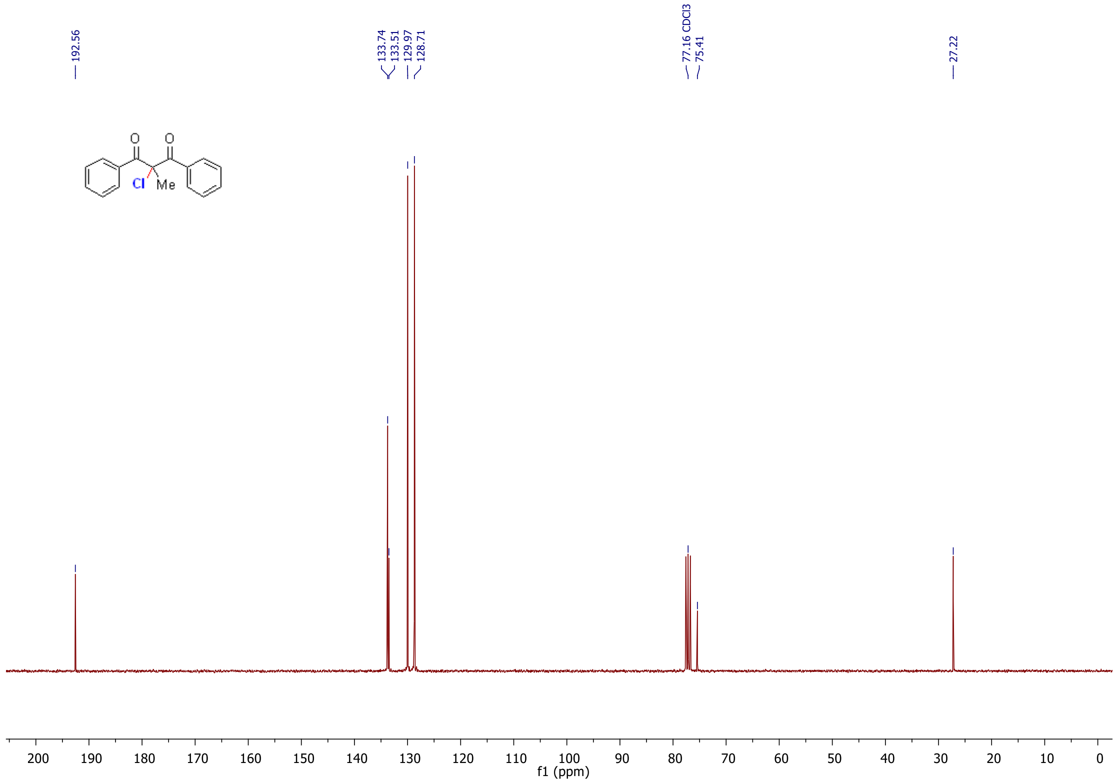

^{13}C NMR (75 MHz, $CDCl_3$)

Ethyl 2-chloro-3-oxo-3-phenylpropanoate (37)

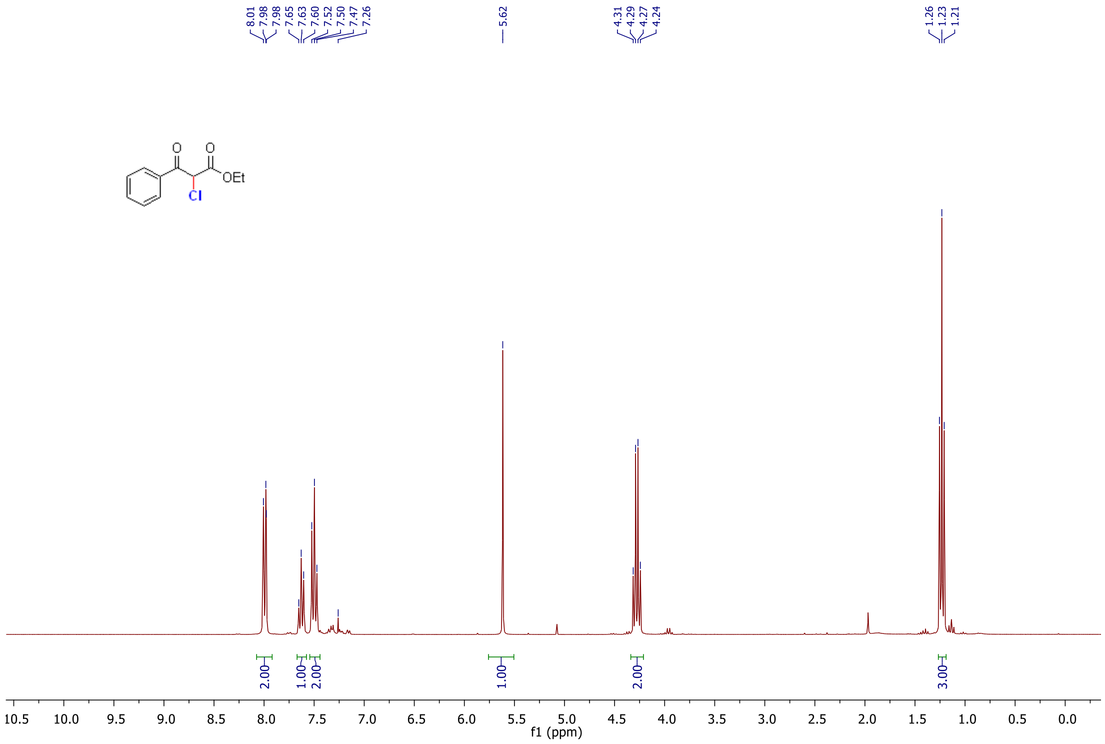

^{1}H NMR (300 MHz, $CDCl_3$)

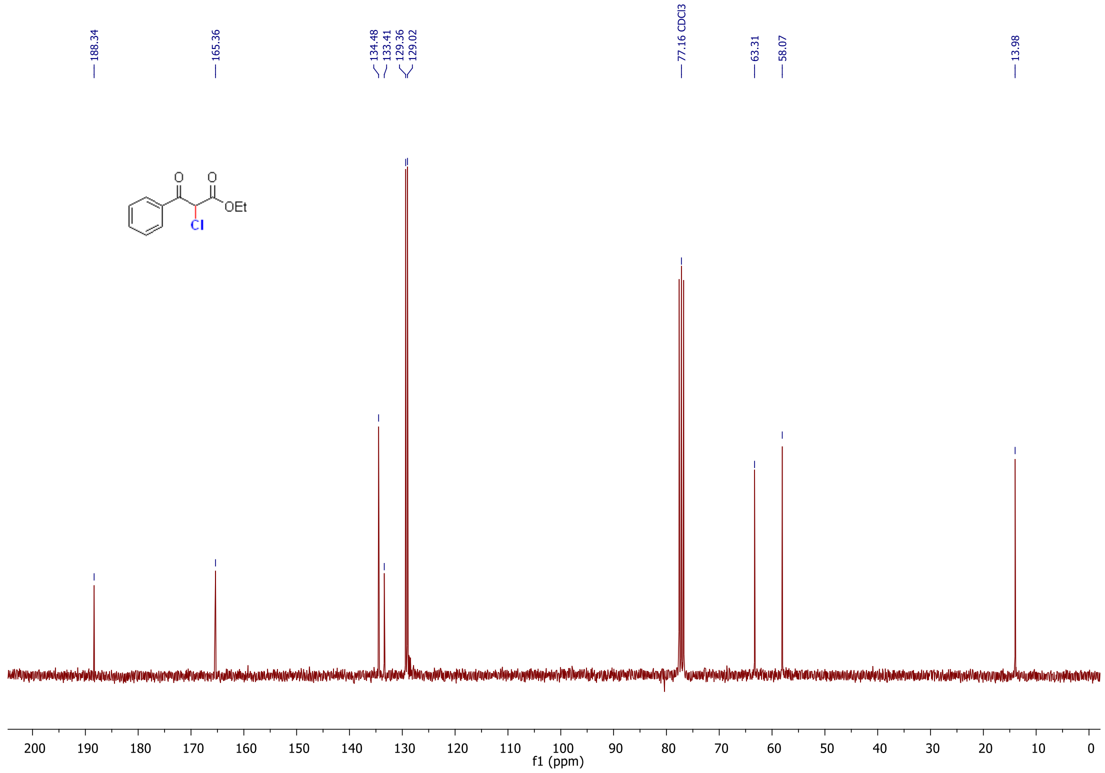

^{13}C NMR (75 MHz, $CDCl_3$)

Ethyl 1-chloro-2-oxocyclohexane-1-carboxylate (38)

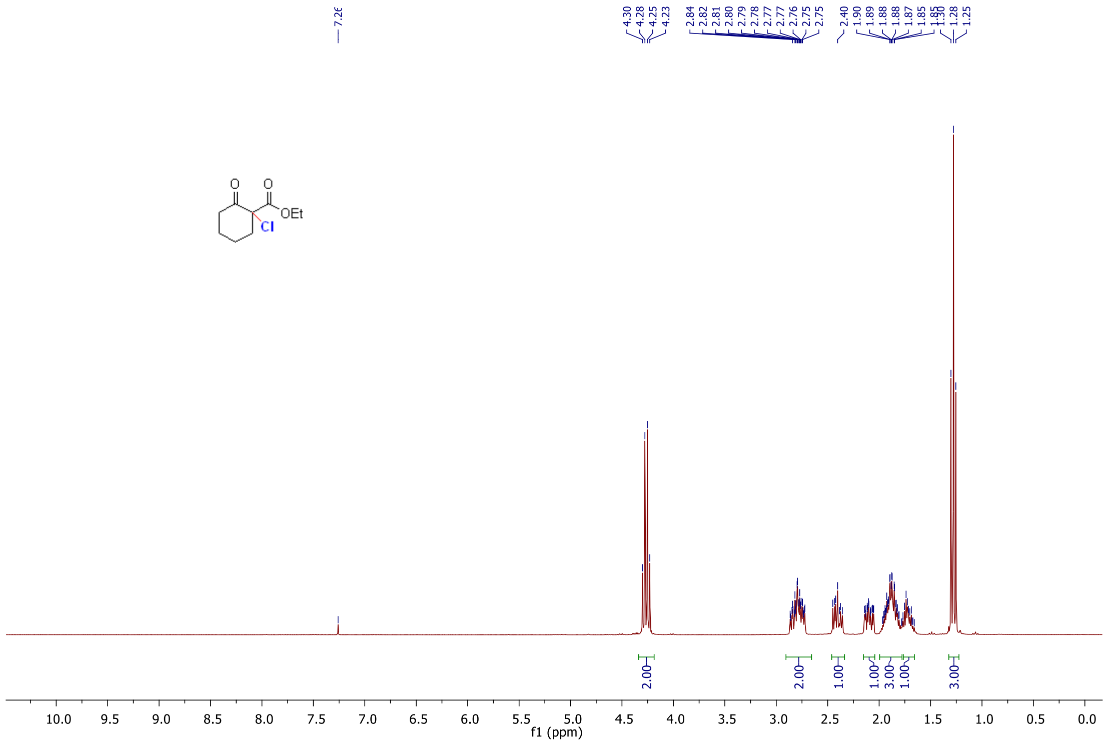

^{1}H NMR (300 MHz, $CDCl_3$)

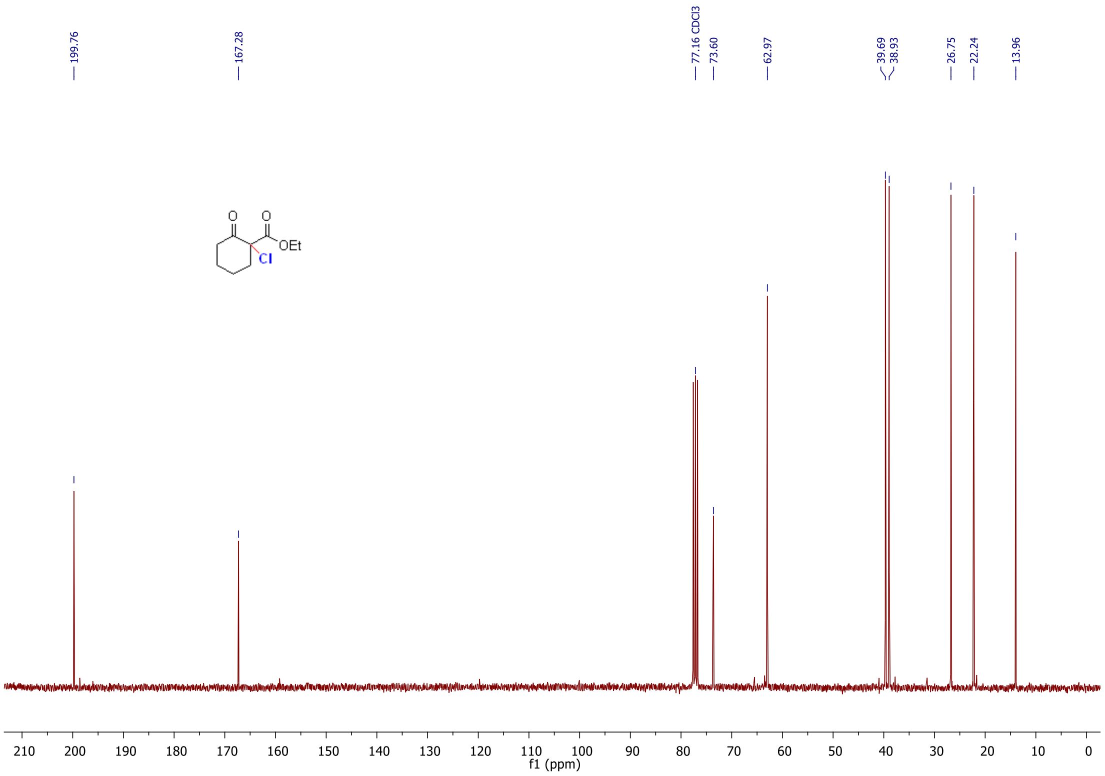

^{13}C NMR (75 MHz, $CDCl_3$)

Ethyl 1-chloro-2-oxocyclopentane-1-carboxylate (39)

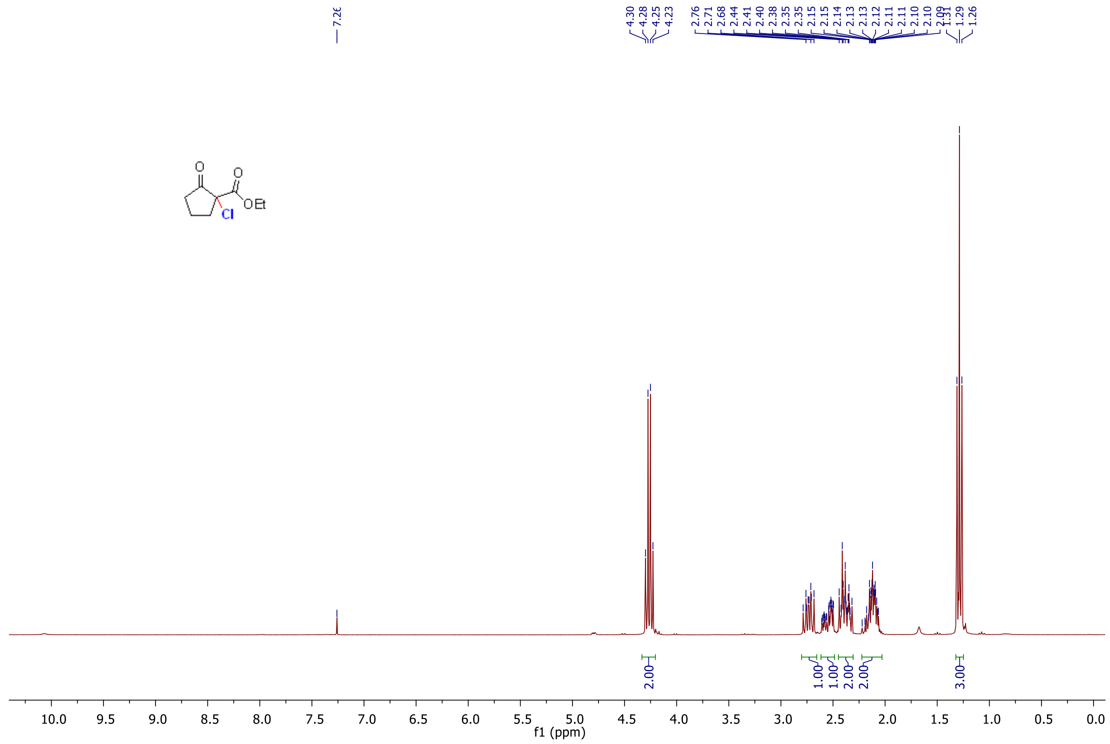

^1H NMR (300 MHz, $CDCl_3$)

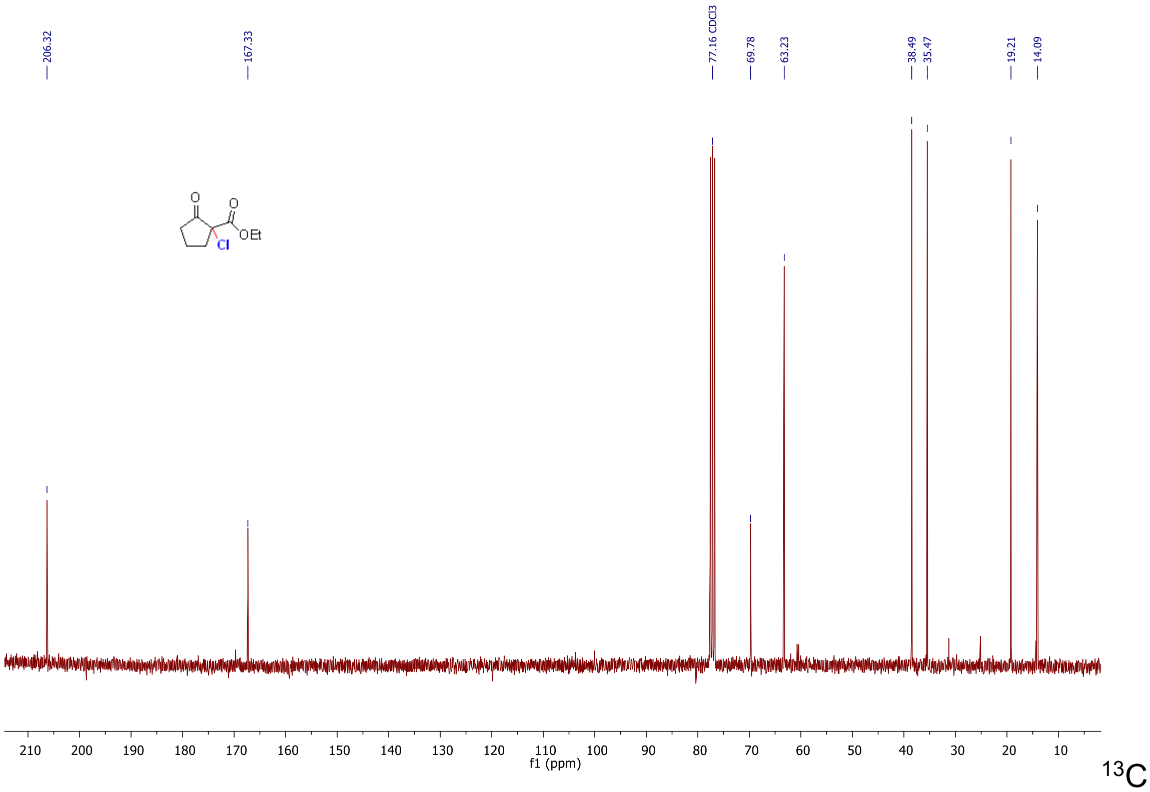

^{13}C NMR (75 MHz, $CDCl_3$)

5. References

1. Powers, D. C., Benitez, D., Tkatchouk, E., Goddard, W. A. & Ritter, T. Bimetallic Reductive Elimination from Dinuclear Pd(III) Complexes. *J. Am. Chem. Soc.* **132**, 14092–14103 (2010).

2. Sarie, J. C., Neufeld, J., Daniliuc, C. G. & Gilmour, R. Catalytic *Vicinal* Dichlorination of Unactivated Alkenes. *ACS Catal.* **9**, 7232–7237 (2019).

3. Zhao, X.-F. & Zhang, C. Iodobenzene Dichloride as a Stoichiometric Oxidant for the Conversion of Alcohols into Carbonyl Compounds; Two Facile Methods for Its Preparation. *Synthesis* 551–557 (2007).

4. Elsherbini, M., Winterson, B., Alharbi, H., Folgueiras-Amador, A. A., Génot, C. & Wirth, T. Continuous-Flow Electrochemical Generator of Hypervalent Iodine Reagents: Synthetic Applications. *Angew. Chem. Int. Ed.* **58**, 9811–9815 (2019).

5. Singh, F. V., Shetgaonkar, S. E., Krishnan, M. & Wirth, T. Progress in organocatalysis with hypervalent iodine catalysts. *Chem. Soc. Rev.* **51**, 8102–8139 (2022).

6. Patra, T., Bellotti, P., Strieth-Kalthoff, F. & Glorius, F. Photosensitized Intermolecular Carboimination of Alkenes through the Persistent Radical Effect. *Angew. Chem. Int. Ed.* **59**, 3172–3177 (2020).

7. Doobary, S., Sedikides, A. T., Caldora, H. P., Poole, D. L. & Lennox, A. J. J. Electrochemical Vicinal Difluorination of Alkenes: Scalable and Amenable to Electron-Rich Substrates. *Angew. Chem. Int. Ed.* **59**, 1155–1160 (2020).

8. Hogg, J. H. Ollmann, I. R., Wetterholm, A., Andberg, M. B., Haeggström, J., Samuelsson, B. & Wong, C.-H. Probing the Activities and Mechanisms of Leukotriene A_4 Hydrolase with Synthetic Inhibitors. *Chem. Eur. J.* **4**, 1698–1713 (1998).

9. Winterson, B., Rennigholtz, T. & Wirth, T. Flow electrochemistry: a safe tool for fluorine chemistry. *Chem. Sci.* **12**, 9053–9059 (2021).

10. Dong, X., Roeckl, J. L., Waldvogel, S. R. & Morandi, B. Merging shuttle reactions and paired electrolysis for reversible vicinal dihalogenations. *Science* **371**, 507–514 (2021).

11. Fu, N., Sauer, G. S. & Lin, S. Electrocatalytic Radical Dichlorination of Alkenes with Nucleophilic Chlorine Sources. *J. Am. Chem. Soc.* **139**, 15548–15553 (2017).

12. Baker, A. E. G., Marchal, E., Lund, K. L. A. R. & Thompson, A. The use of tin(IV) chloride to selectively cleave benzyl esters over benzyl ethers and benzyl amines. *Can. J. Chem.* **92**, 1175–1185 (2014).

13. Lian, P., Long, W., Li, J., Zheng, Y. & Wan, X. Visible-Light-Induced Vicinal Dichlorination of Alkenes through LMCT Excitation of $CuCl_2$. *Angew. Chem. Int. Ed.* **59**, 23603–23608 (2020).

14. Swamy, P., Reddy, M. M., Kumar, M. A., Naresh, M. & Narender, N. Vicinal dichlorination of olefins using NH4Cl and oxone®. *Synthesis* **46**, 251–257 (2014).

15. Brosche, K., Weber, F. G., Westphal, G. & Reimann, E. α-Chlorchalkone. *Z. Chem.* **19**, 96–97 (1979).

16. Xia, J.-D., Deng, G.-B., Zhou, M.-B., Liu, W., Xie, P. & Li, J.-H. Reusable Visible Light Photoredox Catalysts; Catalyzed Benzylic $C(sp^3)$–H Functionalization/ Carbocyclization Reactions. *Synlett* **23**, 2707–2713 (2012).

17. Ding, R., Lan, L., Li, S., Liu, Y., Yang, S., Tian, H. & Sun, B. A Novel Method for the Chlorolactonization of Alkenoic Acids Using Diphenyl Sulfoxide/Oxalyl Chloride. *Synthesis* **50**, 2555–2566 (2018).

18. Tsuchida, K., Kochi, T. & Kakiuchi, F. Copper-Catalyzed Electrochemical Chlorination of 1,3-Dicarbonyl Compounds Using Hydrochloric Acid. *Asian J. Org. Chem.* **2**, 935–937 (2013).

19. Mishra, A. K., Nagarajaiah, H. & Moorthy, J. N. Trihaloisocyanuric Acids as Atom-Economic Reagents for Halogenation of Aromatics and Carbonyl Compounds in the Solid State by Ball Milling. *Eur. J. Org. Chem.* 2733–2738 (2015).

20. Frings, M. & Bolm, C. Enantioselective Halogenation of β-Oxo Esters Catalyzed by a Chiral Sulfoximine–Copper Complex. *Eur. J. Org. Chem.* 4085–4090 (2009).

21. Dousset, M., Le Jeune, K., Cohen, S., Parrain, J.-L. & Chouraqui, G. Tiffeneau–Demjanov Rearrangement by Using α-Chloro-α-diazoacetate: A Direct Access to α-Chlorinated Medium-Size-Ring Ketones via Ring Enlargement. *Synthesis* **48**, 2396–2401 (2016).

22. Hokamp, T. & Wirth, T. Hypervalent Iodine(III)-Catalysed Enantioselective α-Acetoxylation of Ketones. *Chem. Eur. J.* **26**, 10417–10421 (2020).
